# High-throughput screening of 756 chemical contaminants in aquaculture products using liquid chromatography/quadrupole time-of-flight mass spectrometry

**DOI:** 10.1016/j.fochx.2022.100380

**Published:** 2022-06-28

**Authors:** Mingkai Bai, Ruixue Tang, Guorong Li, Wenhai She, Gangjun Chen, Hongmei Shen, Suqin Zhu, Hongwei Zhang, Haohao Wu

**Affiliations:** a College of Food Science and Engineering, Ocean University of China, 5 Yushan Road, Qingdao 266003, China; b Linxia Food Inspection and Testing Center, 8 Renmin Road, Linxia 731100, China; c Yin-chuan Administration for Market Regulation, 205 South Limin Street, Yinchuan 750001, China; d Guangdong Aquatic Resources Industrialization Engineering Technology Research Center, Guangzhou Luxe Seafood Enterprises Ltd., 1 Lushi Road, Guangzhou 510820, China; e Institute of Nutrition and Health, School of Public Health, Qingdao University, 308 Ningxia Road, Qingdao 266021, China; f Technology Center of Qingdao Customs, 83 Xinyue Road, Qingdao 266109, China

**Keywords:** Pesticides, Veterinary drugs, POPs, Marine toxins, Fish, Shellfish, QuEChERS, LC/Q-TOF-HRMS

## Abstract

A high-throughput screening method embracing 756 multiclass chemical contaminants in aquaculture products was developed using modified QuEChERS extraction coupled with liquid chromatography/quadrupole time-of-flight mass spectrometry. A mega-database with retention time/accurate mass data for 524 pesticides, 182 veterinary drugs, 32 persistent organic pollutants and 18 marine toxins was established for compound identification via retrospective library searching. In the four representative matrices (muscle tissues of tilapia and grouper, and edible portions of oyster and scallop), all the database compounds showed acceptable recovery and repeatability with the screening detection limit and limit of quantification below 0.01 mg/kg for >90% of them. The matrix-matched calibration revealed acceptable quantitative property of the method in terms of linear range, linearity, and matrix effect, and fish muscle samples showed stronger matrix effect than shellfish samples. Analysis of 64 real-life samples from aquaculture farms and retail markets evidenced applicability of the proposed method to high-throughput screening scenarios.

## Highlights

• A high-throughput screening method was developed for aquaculture products.
• Modified QuEChERS extraction was used in couple with LC/Q-TOF-HRMS.
• A mega-database was established for 756 multiclass chemical contaminants.
• The method had desirable sensitivity, recovery and repeatability.
• Analysis of real-life samples evidenced applicability of the proposed method.

## Graphical abstract

**Figure:**
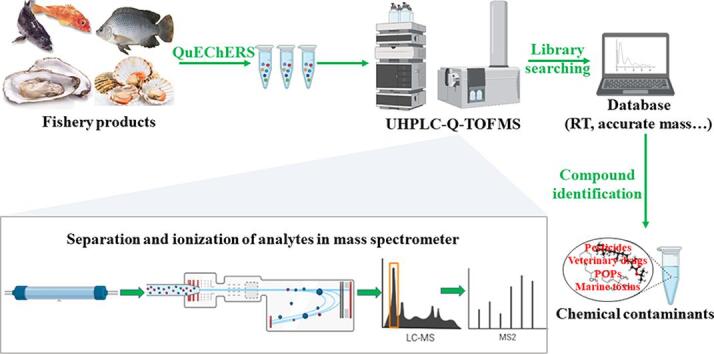

## 1 Introduction

The global demand for fishery products is increasing by virtue of their palatability and nutrition. According to a recent report based on data from Food and Agriculture Organization and The World Bank, global average consumption of fishery products per person rose from 11.5 to 15.1 kg/year in edible weight during the 20-year period from 1998 to 2018, and is expected to increase by over 80% between 2015 and 2050 (Naylor et al., 2021). Since the 1990s, capture fishery production has remained almost unchanged, so to keep production in pace with demand, largescale and high-density aquaculture is becoming common accross the leading aquaculture countries (Wenning, 2020). Modern aquaculture is facing food satety challenges with regard to chemical hazards intentionally introduced or from environmental contamination. On one hand, to avoid losses due to frequent outbreaks of diseases, the use of veterinary drugs is often required in high-density aquaculture to control pathogenic infections. On the other hand, due to enviromental pollution from agriculture and industry, fishery products are inevitably contaminated by pesticides, persistent organic pollutants (POPs), and marine toxins (Lehel, Yaucat-Guendi, Darnay, Palotas, & Laczay, 2021). Consumers have to be aware of chemical contanminants found in aquaculture products that may add up to acceptable residue limits. However, there is a huge aggregate of chemical contanminants (>900 compounds of regulatory concern according to a plethora of regulations worldwide (European Court of, 2019)) falling into the categories of veterinary drugs, pesticides, POPs and marine toxins. Hence, there is the urge for high-throughput screening methods to be developed to monitor the safety of aquaculture products for the interest of public health.

Triple-Quadrupole tandem mass spectrometry (QqQ-MS/MS) is the most common method currently employed in multi-residue analysis. By using a carefully optimized acquisition method in selected reaction monitoring mode, QqQ-MS/MS offers unsurpassed quantitative performance for over one hundred of targeted analytes of regulatory concern in various food matrices, such as 132 veterinary drugs in poultry muscle and 200 pesticides in fruits and vegetables (Cao et al., 2018, Hanot et al., 2015). However, due to inadequate scan speed and low sensitivity in full-scan mode, QqQ-MS/MS can hardly meet the requirements of rapidity and large range for high-throughput non-targeted screening. Recent advances in high resolution mass spectrometers (HRMS) such as Orbitrap and time-of-flight (TOF) mass analyzers map well against the current requirements in non-targeted food safety testing by providing high mass accuracy, resolution, scan speed as well as sensitivity in full-scan mode, and have led to the development of rapid and wide-scope multi-residue screening methods, such as liquid chromatography (LC)/Q-TOF-HRMS screening for over 630 multi-class chemical contanminants in baby food samples (Perez-Ortega et al., 2017). LC-HRMS operating in full scan mode has the ability in principle to record an unlimited number of compounds, so is inherently capable of developing non-targeted screening strategies based on software-assisted library searching against mega-databases of accurate masses and retention times of substances.

In order to detect as many as possible chemical contaminants in food matrices, it is essential to use an efficient and reliable sample preparation method with high recovery and suitable matrix effect for all analytes. The “quick, easy, cheap, effective, rugged, and safe” (QuEChERS) sample preparation procedure was first introduced in 2003 to recover a broad spectrum of pesticides ranging from nonpolar to very polar substances in fruits and vegetables (Anastassiades, Lehotay, Stajnbaher, & Schenck, 2003), and has so far been widely applied in the multi-residue analysis of pesticides, veterinary drugs and other chemical contaminants in a variety of plant and animal foods (Perestrelo et al., 2019). Compared to traditional sample preparation approches (e.g. solid-phase extraction, liquid–liquid extraction, and accelerated solvent extraction), QuEChERS offers the advantages of high analyte recoveries, fast sample treatment, accurate results, little use of solvent, and minimal lab-space and equipment requirements. In order to further improve its technique performance according to the chemical nature of target analytes and matrices, the original QuEChERS method has been submitted to several modifications involving extraction solvent, pH buffering and partitioning salts, clean-up sorbents, and agitation mode (Perestrelo et al., 2019).

To counteract the emerging threat of multi-class chemical contaminants posed to aquaculture products, more powerful analytical strategies are always eagerly needed to conquer the ever increasing complicated challenges. The advanced mass spectrometry in combination with evolution of sample preparation approaches is highly expected to cope with the concerned challenges with performance improvements such as more contaminant coverage, more generic sample preparation procedure, and more competent sensitivity. In this study, a wide-scope screening approach for multiclass chemical contaminants in aquaculture products has been developed using a modified QuEChERS extraction procedure coupled with LC/Q-TOF-HRMS. By using commercially available reference standards, a mega-database of retention times, MS and MS/MS accurate masses of 524 pesticides, 182 veterinary drugs, 32 POPs and 18 marine toxins has been established for the purpose of retrospective library searching. Four important aquaculture species of freshwater and marine aquaculture including tilapia, grouper, oyster and scallop were used as model matrices. The developed method was validated in accordance with the criteria set by European Commission for qualitative and quantitative multi-residue methods (European Commission, 2021, European Commission, 2021; EU Commission, 2020; EU Commission, 2019).

## 2 Materials and methods

### 2.1 Chemicals and reagents

The analytical-grade standards for 524 pesticides, 182 veterinary drugs, 32 POPs, and 18 marine toxins were obtained from Shimadzu Co., Ltd. (Shanghai, China) Alta Scientific Co., Ltd. (Tianjin, China), and National Research Council Canada (Halifax, Canada). HPLC-grade acetonitrile, formic acid and methanol were purchased from Thermo Fisher Scientific Co., Ltd (Shanghai, China). Ultrapure water (resistivity >18 MΩ.cm) was prepared using a Milli-Q Academic system (Merck Millipore, Shanghai, China). Individual stock solutions were prepared at 1 mg/mL in different solvents (mostly acetonitrile, or sometimes methanol) depending upon solubility and stability, and were stored at −80 °C. Intermediate working solutions were prepared for each experiment by appropriate dilution of the stock solutions with acetonitrile. Octadecylsilane (C18) was supplied by ANPEL Laboratory Technologies (Shanghai, China).

### 2.2 Sample preparation

#### 2.2.1 Live sample handling

The live samples of tilapia, grouper, oyster and scallop were acquired from local aquaculture farms and transported to the laboratory in oxygenated water within 1.5 h. Upon arrival, the fish were stunned by delivering a forceful blow to the head using a wood fish bonker followed by exsanguination. White muscle was manually filleted from the fish with skin and bones removed using a knife. Fresh muscle tissues of tilapia and grouper and edible portions of oyster and scallop were chopped into small pieces before being homogenized in a Geno Grinder food processor. The homogenized samples were stored at −20 °C until extraction.

#### 2.2.2 Sample extraction

Sample extraction was carried out according to previous works with some modifications (Casey et al., 2021, Khaled et al., 2019, Mol et al., 2008). A representative portion (2.0 g ± 0.01 g) of homogenized sample was weighed out into a 50-mL polypropylene centrifuge tube and extracted with 8 mL of 0.1% formic acid in acetonitrile for 2 min on vortex mixer. Following centrifugation at 2650 g for 5 min at 4 °C, the full extract was transferred into a 50-mL graduated polypropylene centrifuge tube with its total volume to be about 9.5 mL, taking into account approximate 1.5 mL of water in the 2 g tissue samples. The residue was extracted with 8 mL of 0.1% formic acid in acetonitrile again to recover contaminant remains, and after centrifugation, the second extract (≈8 mL) was combined with the first extract to obtain a total volume of approximate 17.5 mL of the combined extract.

#### 2.2.3 Sample cleanup

For clean-up, 500 mg end-capped octadecylsilane (C18) dispersive sorbent was added into the combined extract. After vortexing for 1 min and subsequent centrifugation at 3820*g* for 10 min at 4 °C, the clean-up supernatant (≈17 mL) was transferred into a glass tube, evaporated to dryness under nitrogen flow, redissolved in 1 mL acetonitrile-H_2_O (v/v 50:50), and filtered through 0.22 μm PTFE syringe filters for matrix-matching or LC/Q-TOF-HRMS analysis.

### 2.3 LC/Q-TOF-HRMS parameters

LC/Q-TOF-HRMS analysis was carried out on an Agilent 1290 Infinity ΙΙ ultra-high performance LC system coupled to an Agilent G6530C Q-TOF mass spectrometer (CA, USA). An ACQUITY BEH C18 column (100 × 2.1 mm, 1.7 μm, Waters, Ireland) was used to conduct the separation at 40 °C with the flow rate at 0.4 mL/min and the injection volume at 5 μL as per the manufacture’s guidelines. Mobile phase A was acetonitrile while mobile phase B was 0.1% formic acid in water. The elution gradient program was 0–0.5 min/5% A, 0.5–3 min/5–15% A, 3–6 min/15–40% A, 6–9 min/40% A, 9–15 min/40–60% A, 15–19 min/60–99% A, and 19–23 min/99% A. Analyte ionization was performed with an electrospray ionization source in the positive ion mode using the following operating parameters: source temperature, 450 °C; capillary voltage, 4000 V; gas temperature, 325 °C; drying gas flow, 11 L/min; nebulizer pressure, 40 psig; sheath gas temperature, 350 °C; sheath gas flow, 11 L/min; fragmentor voltage, 175 V; skimmer voltage, 65 V; octupole RF, 750 V. Nitrogen was used as the collision-induced dissociation gas. LC-MS accurate mass spectra were recorded in full-scan mode across the range *m*/*z* 50–1500 at an acquisition rate of 2 scan per second. The MS/MS data aquisition was operated in the target mode (targeted analysis with fragment ion confirmation) to extract exact mass chromatograms of compounds and fragments with a dwell time of 2 ms. The data were recorded with Agilent MassHunter Data Acquisition software (version B.07.00) and analyzed with Agilent MassHunter Qualitative Analysis software (version B.07.00).

### 2.4 Database development and identification criteria

The single or mixed standard solutions at individual concentrations of 100 ng/mL each were injected in the LC/Q-TOF-HRMS system to collect retention time data, MS and MS/MS accurate masses of target ions. A Microsoft Excel spreadsheet was created containing compound name, molecular formula, retention time, exact mass, collision energy and fragment ions for each analyte, and was converted into comma-separated values format for retrospective library searching by the Agilent MassHunter Data Acquisition software (version B.07.00). The raw data of a sample run can then be automatically matched against the database within a defined tolerance for retention time (±0.1 min) and exact mass (±5 ppm) by the MassHunter software (European Commission, 2021, European Commission, 2021, European Commission, 2019).

### 2.5 Method validation

In this work, the method validation for qualitative and quantitative analysis was carried out in accordance with the European Commission guidance document SANTE/12682/2019 (EU Commission, 2019). The homogenized samples (2 g) were spiked with 20, 40, and 100 μL of standard working solutions at the fortification levels of 10, 20, and 50 μg/kg, respectively. The spiked samples were allowed to remain at 4 °C for at least 8 h to ensure the appropriate distribution of the analytes, followed by the above descriped sample preparation procedure to obtain sample extracts. The recoveries were determined using matrix-matched calibration curves to compensate for matrix effects in LC/Q-TOF-HRMS analysis. The repeatability was expressed as the relative standard deviation (RSD) of 5 replicated measurements. The limit of quantification (LOQ) is defined as the lowest level with sufficient recovery and RSD according to the criteria of SANTE/2020/12830 (European Commission, 2021, European Commission, 2021), i.e., 60–120% recovery range and 30% RSD at a spiking level equal to or lower than 0.01 mg/kg, and 70–120% recovery range and 20% RSD at a spiking level of 0.01–0.1 mg/kg. For screening detection limit (SDL) evaluation, 20 fortified samples at each spiking level were analyzed, and the lowest concentration required to detect an analyte in at least 19 of 20 trials (false-negative rate of 5%) was assigned as SDL.

The solvent-only and matrix-matched standards at the concentrations of 10, 20, 50, 100, 150, and 250 ng/mL were analyzed to determine the linearity of calibration, matrix effect, accuracy and precision. The correlation coefficient (r^2^) of the calibration curve was calculated to evaluate the linearity of calibration. The linear range of target analyte was determined as the largest concentration range with r^2^ higher than 0.90. The matrix effect was evaluated according to the percent difference in the slopes of calibration curves of the matrix-matched standards and the solvent-only standards using the following equation: matrix effect (%) = 100% × (slope_matrix-matched_/slope_solvent-only_ − 1). The applicability of the method on real-life aquaculture products was investigated by analyzing market and farm samples of tilapia, grouper, oyster and scallop.

## 3 Results and discussion

### 3.1 Screening method development and general considerations

It is a complex process to simultaneously detect hundreds of trace amounts of chemicals at the µg kg^−1^ level in complex matrices with approxiamately 5000–20,000 food components that may interfere (Gomez-Ramos, Ferrer, Malato, Aguera, & Fernandez-Alba, 2013), so effective approaches capable of separating and indentifying these chemicals are required for high-throughput non-targeted screening. According to preliminary studies, a dedicated LC elution gradient is needed for efficient separation and detection in multi-residue analysis. Considering that a short chromatographic run (e.g. 5 min) could result in strong analyte-to-analyte interference and matrix effect due to substantial overlapping, a 23-min gradient was finally selected as it provides appropriate separation conditions for 512 pesticides and 160 veterinary drugs according to the China national standards (China National Food Safety Standard, 2016, Ministry of Agriculture and Rural Affairs, 2019). To obtain a well-distributed elution profile of multi-class food contaminants over the gradient, the chromatographic program used was carefully designed in combination of those in the two standards.

**Fig. 1:**
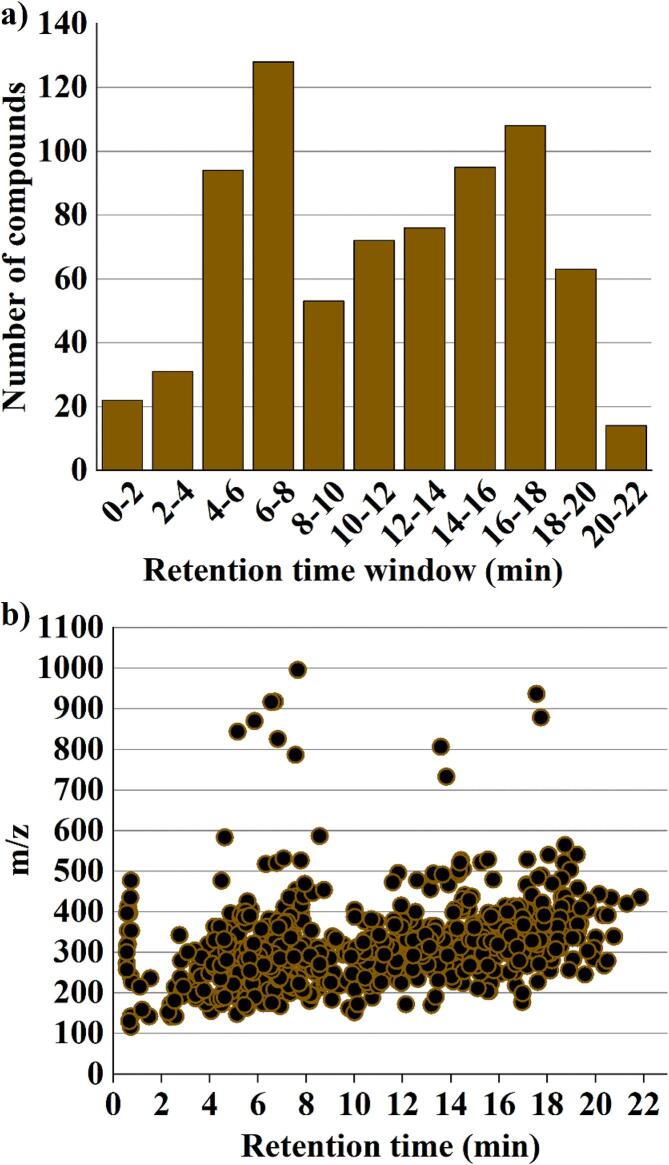
(a) Distribution of the database compounds according to their retention times; (b) The 2D-plot of *m*/*z* versus retention time for the database compounds.

Fig. 1a and 1b display the distributions of number density and *m*/*z* value, respectively, for the 756 analytes through the LC run to provide a glimpse of the task. Within a retention time window of 2 min, there was a maximum analyte number of 128 eluting from the LC column, so that the acquisition rate of 2 scan per second (0.5 s per scan) could fit well with the separation conditions. In addition, there were scatterly distributed *m*/*z* values at similar retention times in Fig. 1b, so that weak analyte-to-analyte interference could be expected to occur. It should be noted that there were some early eluting highly polar compounds amongst the plethora of contaminants tested. According to the criteria set by European Commission, the minimum acceptable retention time for the analyte under LC-MS quantitative analysis is twice the retention time corresponding to the void volume of the column (EU Commission, 2002). Considering the void volume (i.e., 0.4 mL) and void retention time (i.e., 1 min) of the column used in this study, 22 compounds were found not to meet this criterion. These analytes included a pesticide (methamidophos), 8 veterinary drugs (metformin hydrochloride, pirbuterol, 1-aminohydantoin hydrochloride, terbutaline, sulfaguanidine, 4-acetamidophenol, buformin hydrochloride, and procainamide), and 13 marine toxins (tetrodotoxin, *n*-sulfocarbamoylgonyautoxin-2, decarbamovlgonyautoxin-2, decarbamovlgonyautoxin-3, decarbamoylneosaxitoxin, decarbamoylsaxitoxin, gonyautoxin-1, gonyautoxin-4, gonyautoxin-2, gonyautoxin-3, gonyautoxin-6, neosaxitoxin, saxitoxin). The quantitative determination of these contaminants would request the use of other types of chromatographic columns, such as ion-exchange and hydrophilic interaction columns as previously reported for the quantification of marine toxins (Christian & Luckas, 2008).

For sample extraction, we selected an acidified acetonitrile extraction solvent previously designed for simultaneous extraction of various classes of pesticides, mycotoxins, plant toxins, and veterinary drugs in various matrices (Mol, Plaza-Bolanos, Zomer, de Rijk, Stolker, & Mulder, 2008). The acidified acetonitrile extraction has been fully in-house validated for the LC-MS/MS based quantification of >1200 biotoxins, pesticides and veterinary drugs in two compound feed matrices (Steiner et al., 2020). For dispersive solid phase extraction clean-up, we selected the end-capped C18 sorbent designed to improve the qualitative screening performance of LC-MS/MS for >100 analytes in mussle tissues (Geis-Asteggiante, Lehotay, Lightfield, Dutko, Ng, & Bluhm, 2012). The end-capped C18 dispersive sorbent has recently been successfully applied for the LC-MS/MS analysis of muscle from bison, deer, elk, and rabbit to test for 112 veterinary drug residues (Casey, Andersen, Williams, Nickel, & Ayres, 2021).

### 3.2 Establishment of the screening database for multiclass food contaminants

Considering their potential presence in aquaculture water and feed, over 800 chemical contaminants including 524 pesticides, 190 veterinary drugs, 82 POPs, and 18 marine toxins (Tables S1 and S2) were carefully selected based on the different lists of regulatory concern in China, the United States, and the European Union (EU), and their reference standards were commertially obtained to tentatively establish a screening database by using our LC/Q-TOF-HRMS method. Among these compounds, 8 veterinary drugs and 46 POPs (i.e., 24 chlorinated and brominated POPs and 22 polycyclic aromatic hydrocarbons) were not amenable to LC–MS analysis due to relatively poor ionization efficiencies (Table S1), which was in line with previous reports that chlorinated and brominated POPs and polycyclic aromatic hydrocarbons are mostly quantified by gas chromatography coupled to mass spectrometry (Kim et al., 2019, Salihovic et al., 2013). Four POPs including biphenyl, 2-chlorobiphenyl, 4-chlorobiphenyl, and decachlorobiphenyl were only detected in the negative ion mode (Table S1), so were not included in the screening database. Finally, there were 756 food contaminants included in the screening database. Table S2 shows detailed information about elemental composition, retention time, ionization type, theoretical and experimental exact mass *m*/*z* values, quantitative and qualitative fragment ions, and collision energy. Protonated molecules were detected in most cases, and exceptionally for some compounds (∼10%), either sodium or ammonium adducts were identified as the most abundant ion. All compounds included were detected in positive ion mode, so that rapid screening could be achieved by one LC/Q-TOF-HRMS run.

It is generally valid to screen food contaminants by automated matching to retention times and exact mass *m*/*z* values in the database, but when the matched ions are of low abundance, false negatives may occur due to matrix interference. As per the guidance of EU, only diagnostic ions (including the molecular ion, characteristic fragment or product ions) with a relative intensity of >10% in the reference spectrum of the calibration standard, matrix-matched standard or matrix-fortified standards are suitable for LC-MS full-scan screening methods (European Commission, 2021, European Commission, 2021). Therefore, very low-abundant suspected contaminants should be confirmed by matching to characteristic fragment ions in the database. According to the criterion set by European Commission, HRMS confirmation can be achieved by monitoring 2 ions with a mass accuracy of ≤5 ppm (<1 mDa for *m*/*z* <200), preferably including the precursor ion (e.g. molecular ion, protonated molecule or adduct ion) and at least one fragment ion (EU European Commission, 2021, European Commission, 2021). In this study, for confirmation purposes, the first two most abundant fragment ions for each precursor ion were recorded at the carefully optimized collision energy (Table S2).

The contaminant coverage of a HRMS database is definitely crucial in real-life high-throughput screening scenarios for food safety risk assessment. Several studies have established HRMS-based multiresidue screening methods for aquaculture products in recent years, but their screening databases covered a limited number of contaminants ranging from tens to over a hundred (Dasenaki et al., 2015, Turnipseed et al., 2019). This study established a screening database covering up to 756 contaminants falling within the major categories of aquaculture pollutants including pesticides, veterinary drugs, POPs, and marine toxins. Apparently, the mega-database established here could confer a superiority of our screening method over those previously reported in contaminant coverage and screening capacity.

### 3.3 Method performance

**Fig. 2:**
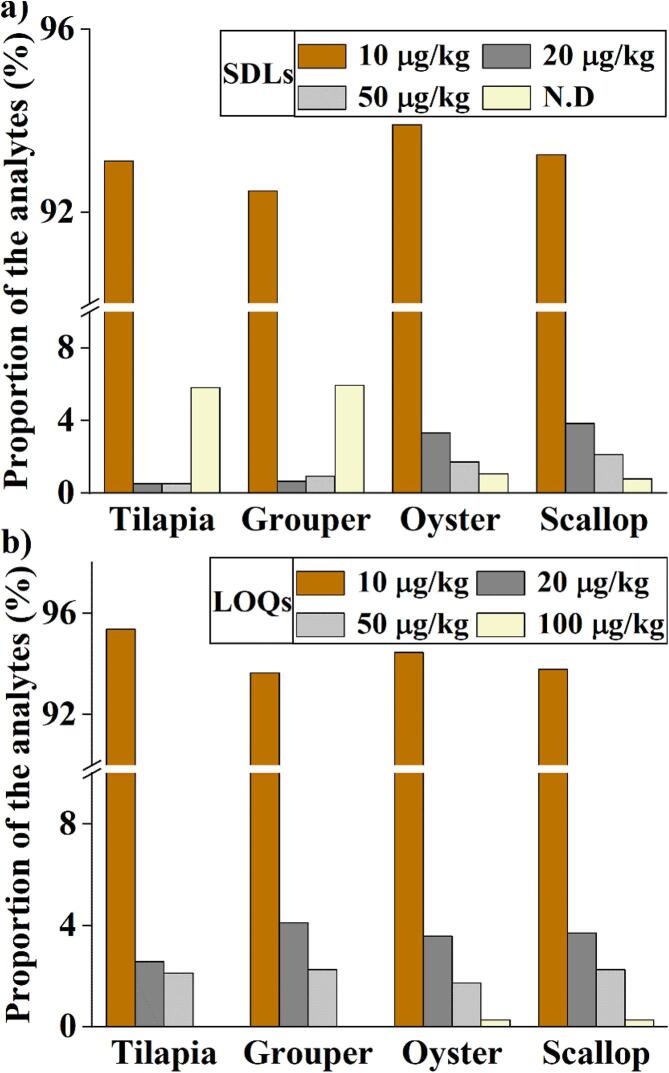
The distributions of (a) screening detection limits (SDLs) and (b) limits of quantification (LOQs) for the database compounds in four representative matrices.

According to the SANTE/12682/2019 guidelines of European Commission, SDL should be provided for qualitative multi-residue methods to establish the confidence of detection of an analyte at a certain concentration level (EU Commission, 2019). As shown in Table S3 and Fig. 2a, there were 93.12%, 92.46%, 93.91%, and 93.25% of the contaminants under study showing an SDL equal to or lower than the general default EU maximum residue level (MRL) of 0.01 mg/kg in the 4 matrices of tilapia, grouper, oyster and scallop, respectively, which demonstrates that the proposed screening method was adequately sensitive for the non-targeted screening purpose.

Table S4 shows a summary of the results of recovery and RSD. All the database compounds were found to have acceptable recoveries and RSDs in the 4 matrices as per the criteria set by the SANTE/2020/12830 guidelines (European Commission, 2021, European Commission, 2021), and over 93.5% of them had LOQs at the EU MRL threshold of 0.01 mg/kg in all matrices (Fig. 2b). These results suggest that the proposed screening method also had satisfactory trueness, repeatability, and sensitity for quantitative purposes.

In the case of matrix-matched calibration, a linear range from 10 to 250 ng/mL was observed for 94.31%, 92.73%, 92.99%, and 92.06% of the compounds in the solvents matched with the extracts from tilapia, grouper, oyster and scallop, respectively (Table 1 and S5). This linear range is sufficient to cover a contanminant concentration range of 0.005–0.125 mg/kg in the samples, which is of 2 orders of magnitude, indicating an acceptable quantitative capability of the LC/Q-TOF-HRMS instrument. As shown in Table 2, there were over 93.39% of the compounds showing r^2^ > 0.99 within their linear ranges in the matrix-matched calibration, which again implicates a good quantitative property of the proposed screening method in terms of linearity.

**Table 1:** The distributions of linear ranges for the database compounds in the four representative matrices.

Matrix	Linear range (ng/mL)	No. of analytes	% of analytes	Remarks
Tilapia	10–250	713	94.31	
	10–100	1	0.13	**Veterinary drugs** (glimepiride)
	20–250	4	0.53	**Pesticides** (tau-fluvalinate, 2,2′,4,4′,5,5′-hexachlorobiphenyl, methothrin, phorate sulfoxide)
	50–250	1	0.13	**Pesticides** (2,2′,3,4,4′,5′-hexachlorobiphenyl)
	non-linear	37	4.90	Pesticides (propham, ethalfluralin, aldrin, β-HCH, pendimethalin, chlorbenside, bromophos-ethyl, phenthoate, chlorflurenol-methyl, methidathion, oxadiazon, *cis*-permethrin, *trans*-permethrin, dichlobenil, nitrapyrin, tecnazene, hexachlorobenzene, chlorbufam, isofenphos, chlorfenson, bromopropylate, cyfluthrin, 3,5-dichloroaniline, *cis*-1,2,3,6-Tetrahydrophthalimide, heptachlor, prallethrin, chlorobenzilate, uniconazole, fluorodifen, fludioxonil, acrinathrin, λ-cyhalothrin, musk ketone, trifloxystrobin, demeton); **Veterinary drugs** (kanamycin sulfate, tobramycin)
	sum	756	100	
Grouper	10–250	701	92.73	
	10–100	2	0.26	**Pesticides** (propazine, ditalimfos)
	20–250	10	1.32	**Pesticides** (3,5-dichloroaniline, bioallethrin, sethoxydim, bromocylen, 2,2′,4,4′,5,5′-hexachlorobiphenyl, phorate sulfoxide, nadifloxacin, hygromycin B); **Veterinary drugs** (sparfloxacin, ketotifen)
	50–250	3	0.40	**Pesticides** (mepiquat chloride, 2,2′,3,4,4′,5′-hexachlorobiphenyl, dicloran)
	non-linear	40	5.29	**Pesticides** (propham, ethalfluralin, phorate, fonofos, aldrin, β-HCH, δ-HCH, chlorbenside, bromophos-ethyl, phenthoate, chlorflurenol-methyl, methidathion, oxadiazon, diclofop-methyl, *cis*-permethrin, *trans*-permethrin, dichlobenil, nitrapyrin, tecnazene, hexachlorobenzene, chlorbufam, isofenphos, chlorfenson, azinphos-ethyl, cyfluthrin, *cis*-1,2,3,6-tetrahydrophthalimide, heptachlor, prallethrin, chlorobenzilate, uniconazole, fluorodifen, fludioxonil, λ-cyhalothrin, musk ketone, trifloxystrobin, cafenstrole, demeton); **Veterinary drugs** (sulfacetamide, kanamycin sulfate, tobramycin)
	sum	756	100	
Oyster	10–150	10	1.32	**Pesticides** (β-HCH, flutolanil, terbufos, terbumeton, hexaconazole, fluazifop-butyl, pentachloroanisole, methabenzthiazuron); **Veterinary drugs** (repaglinide, pioglitazone hydrochloride)
	10–250	703	92.99	
	20–250	26	3.44	**Pesticides** (ethalfluralin, fonofos, chlorbenside, bromophos-ethyl, chlorflurenol-methyl, *cis*-permethrin, hexachlorobenzene, dicloran, benalaxyl, azinphos-ethyl, 3,5-dichloroaniline, plifenate, uniconazole, fluorodifen, λ-cyhalothrin, esfenvalerate, BDMC-1, octachlorostyrene, musk ketone, flurochloridone, 2,2′,3,4,4′,5,5′-heptachlorobiphenyl); **Veterinary drugs** (eprinomectin); **Persistent organic pollutants** (TDCPP, diethyl phthalate, diisobutyl phthalate, okadaic acid)
	50–250	11	1.46	**Pesticides** (α-endosulfan, tolylfluanid, 2,4′-DDD, cyfluthrin, tefluthrin, isocarbophos, 2,2′,4,4′,5,5′-hexachlorobiphenyl, acibenzolar-s-methyl, benfuresate); **Veterinary drugs** (1-aminohydantoin hydrochloride, kanamycin sulfate)
	100–250	2	0.26	**Pesticides** (2,2′,3,4,4′,5′-hexachlorobiphenyl, cafenstrole)
	non-linear	4	0.53	**Pesticides** (chlorbenside sulfone, bromopropylate, fludioxonil, demeton)
	sum	756	100	
Scallop	10–150	12	1.59	**Pesticides** (β-HCH, cypermethrin, terbufos, z-tetrachlorvinphos, disulfoton, propisochlor, fluazifop-butyl, pentachloroanisole, pyriminobac-methyl); **Veterinary drugs** (repaglinide, gliquidone, pioglitazone hydrochloride)
	10–250	696	92.06	
	100–250	2	0.26	**Pesticides** (2,2′,3,4,4′,5′-hexachlorobiphenyl, cafenstrole)
	20–250	29	3.84	**Pesticides** (fonofos, aldrin, chlorbenside, chlorflurenol-methyl, *cis*-permethrin, dichlobenil, hexachlorobenzene, dicloran, benalaxyl, 3,5-dichloroaniline, plifenate, uniconazole, flumetralin, fluorodifen, λ-cyhalothrin, esfenvalerate, BDMC-1, octachlorostyrene, musk ketone, flurochloridone, 2,2′,3,4,4′,5,5′-heptachlorobiphenyl, methomyl, 1-aminohydantoin hydrochloride); **Veterinary drugs** (sparfloxacin, eprinomectin, ivermectin); **Persistent organic pollutants** (diethyl phthalate, diisobutyl phthalate, okadaic acid)
	50–250	14	1.85	**Pesticides** (ethalfluralin, fenchlorphos, α-endosulfan, crotoxyphos, 2,4′-DDD, cyfluthrin, tefluthrin, isocarbophos, 2,2′,4,4′,5,5′-hexachlorobiphenyl, acibenzolar-s-methyl, benfuresate, methothrin, mepiquat chloride); **Veterinary drugs** (kanamycin sulfate)
	non-linear	3	0.40	**Pesticides** (chlorbenside sulfone, fludioxonil, demeton)
	sum	756	100

**Table 2:** The distributions of correlation coefficients (r^2^) for the database compounds in the four representative matrices.

Matrix	r^2^	No. of analytes	% of analytes	Remarks
Tilapia	≥0.990	706	93.39	
	0.980–0.990	7	0.93	**Pesticides** (chlorpyrifos, tetrasul, flurochloridone, bromuconazole, disulfoton, bupirimate, bifenthrin)
	0.900–0.980	6	0.79	**Pesticides** (cycloxydim, edifenphos, mexacarbate, fenthion sulfoxide, mirex, phthalimide)
	<0.900	37	4.89	**Pesticides** (propham, ethalfluralin, aldrin, β-HCH, pendimethalin, chlorbenside, bromophos-ethyl, phenthoate, chlorflurenol-methyl, methidathion, oxadiazon, *cis*-permethrin, *trans*-permethrin, dichlobenil, nitrapyrin, tecnazene, hexachlorobenzene, chlorbufam, isofenphos, chlorfenson, bromopropylate, cyfluthrin, 3,5-dichloroaniline, *cis*-1,2,3,6-tetrahydrophthalimide, heptachlor, prallethrin, chlorobenzilate, uniconazole, fluorodifen, fludioxonil, acrinathrin, λ-cyhalothrin, musk ketone, trifloxystrobin, demeton); **Veterinary drugs** (kanamycin sulfate, tobramycin)
	sum	756	100	
Grouper	≥0.990	710	93.92	
	0.980–0.990	4	0.53	**Pesticides** (tetramethrin, dipropetryn, fthalide, cyazofamid)
	0.900–0.980	1	0.13	**Pesticides** (bromuconazole)
	<0.900	41	5.42	**Pesticides** (propham, ethalfluralin, phorate, fonofos, aldrin, β-HCH, δ-HCH, chlorbenside, bromophos-ethyl, phenthoate, chlorflurenol-methyl, methidathion, oxadiazon, diclofop-methyl, *cis*-permethrin, *trans*-permethrin, dichlobenil, nitrapyrin, tecnazene, hexachlorobenzene, chlorbufam, isofenphos, chlorfenson, azinphos-ethyl, cyfluthrin, *cis*-1,2,3,6-Tetrahydrophthalimide, heptachlor, prallethrin, chlorobenzilate, uniconazole, fluorodifen, fludioxonil, λ-cyhalothrin, musk ketone, trifloxystrobin, cafenstrole, demeton); **Veterinary drugs** (sulfacetamide, kanamycin sulfate, tobramycin); **Persistent organic pollutants** (tributyl phosphate)
	sum	756	100	
Oyster	≥0.990	748	98.94	
	0.980–0.990	0	0	
	0.900–0.980	4	0.53	**Pesticides** (4,4′-DDE, pebulate, ditalimfos, dichlobenil)
	<0.900	4	0.53	**Pesticides** (chlorbenside sulfone, bromopropylate, fludioxonil, demeton)
	sum	756	100	
Scallop	≥0.990	753	99.60	
	0.980–0.990	0	0	
	0.900–0.980	0	0	
	<0.900	3	0.40	**Pesticides** (chlorbenside sulfone, fludioxonil, demeton)
	sum	756	100	

**Fig. 3:**
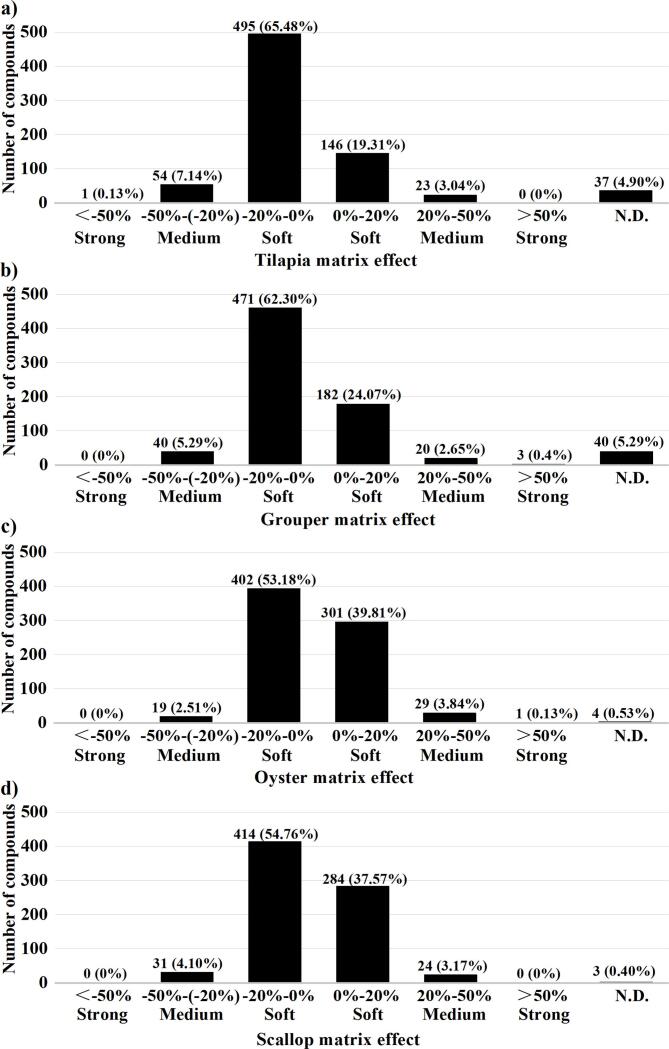
The distributions of matrix effects for the database compounds in the four representative matrices of (a) tilapia, (b) grouper, (c) oyster, and (d) scallop.

When matched with the extracts from tilapia and grouper, there were 37 and 40 compounds, respectively, without showing good linearity (i.e. r^2^ < 0.90) within the studied concentration range, which were much more than those, i.e., 4 and 3 compounds, when matched with the extracts from oyster and scallop, respectively (Table 1). It thus seems that fish muscle samples presented greater matrix effects than shellfish samples. In fact, matrix effects usually occur during ionization step in MS determinations, where matrix components may cause some quantitation problems by suppressing or enhancing the signals of coeluted target analytes. By comparing the ratio of the slope of the matrix-matched calibration curve to that of the solvent-only calibration curve (Table S5), the matrix effect of each compound can be expressed as soft signal suppression (−20% to 0%) or enhancement (0% to 20%), medium signal suppression (−50% to −20%) or enhancement (20% to 50%), and strong signal suppression (<−50%) or enhancement (>50%) according to Ferrer et al. (2011) and Kmellar et al. (2008) (Ferrer et al., 2011, Kmellár et al., 2008). There were 703 (92.99%) and 698 (92.33%) compounds showing soft matrix effects (between −20% and 20%, considered as no significant matrix effect (EU European Commission, 2021, European Commission, 2021)) when matched with the extracts from oyster and scallop, respectively, which were much more than those, i.e., 641 (84.79%) and 653 (86.37%) compounds, when matched with the extracts from tilapia and grouper, respectively (Fig. 3). This again implicated stronger matrix effects of fish muscle samples than shellfish samples.

### 3.4 Application to real-life samples

To further evaluate its practicability for real-world scenarios, the proposed method was applied to non-targeted screening analysis of 64 samples of fishery products from aquaculture farms and retail markets in China, and the positive analytes were simutaneously quantified by using the matrix-matched calibration curves established in the present study. Table S6 shows detailed information on the positive analytes in these real-life samples. Two positive tilapia samples were found containing the pesticide fenpropidin at the residue levels slightly higher than the established EU MRL of 0.02 mg/kg in meats of swine, bovine, sheep, goat and poultry animals (Authority, 2011). The pesticide metazachlor was found in one grouper sample at the concentration of 0.04 mg/kg, which was below the established EU MRL of 0.05 mg/kg in meats of swine, bovine, sheep, goat and poultry animals (Authority, 2014). The pesticide phorate sulfoxide was detected in another grouper sample at the concentration of 0.005 mg/kg, which was well below the established China MRL of 0.02 mg/kg in mammalian meats (China National Food Safety Standard, 2021). There was one oyster sample found containing the pesticide mefenpyr-diethyl at the residue level below the established Australia MRL of 0.01 mg/kg in mammalian meats (National Registration Authority for Agricultural Veterinary Chemicals, 2003). The pesticide methoprene was detected in one scallop sample at the concentration of 0.04 mg/kg, which was below the established United States MRL of 0.10 mg/kg in meats of swine, bovine, sheep, and goat animals (United States Environmental Protection Agency, 1991). Dihexyl phthalate, one of the POPs, was detected in 1 oyster sample at very low residue levels (<EU general MRL threshold of 0.01 mg/kg), and its MRL value is currently unavailable.

## 4 Conclusions

In summary, a new LC/Q-TOF-HRMS method has been developed and validated for wide-scope screening of multiclass chemical contaminants in fishery products. A mega-database containing information about retention times and MS and MS/MS accurate masses for 524 pesticides, 182 veterinary drugs, 32 POPs and 18 marine toxins has been established for high-throughput compound identification through retrospective library searching. Analytical performance evaluations revealed adequate sensitivity of the proposed method for non-targeted screening assessment purposes, as well as its satisfactory quantitative properties in terms of trueness, repeatability, sensitity, linearity and matrix effect. Practical application of this method to real-life samples validated its screening and quantification capabilities. These results indicated the desirable reliability, time efficiency and simplicity of the method to meet the requirements of high-throughput routine screening of a wide scope of suspicious chemical contaminants in aquaculture products.

## CRediT authorship contribution statement

**Mingkai Bai:** Data curation, Methodology, Formal analysis, Writing – original draft, Writing – review & editing. **Ruixue Tang:** Formal analysis, Writing – review & editing. **Guorong Li:** Investigation, Methodology. **Wenhai She:** Software, Investigation. **Gangjun Chen:** Validation, Software. **Hongmei Shen:** Funding acquisition, Supervision. **Suqin Zhu:** Writing – review & editing, Methodology. **Hongwei Zhang:** Project administration. **Haohao Wu:** Supervision.

## Declaration of Competing Interest

The authors declare that they have no known competing financial interests or personal relationships that could have appeared to influence the work reported in this paper.
