# Supplementary material for: High-throughput screening of 756 chemical contaminants in aquaculture products using liquid chromatography/quadrupole time-of-flight mass spectrometry
Source: Food Chem X. 2022 Jun 28;15:100380. doi: 10.1016/j.fochx.2022.100380 (PMC9532709; doi:10.1016/j.fochx.2022.100380)
Supplement: Supplementary data 1 [file mmc1.docx]

Supporting Information

**High-Throughput** **Screening of** **756 Chemical Contaminants in Aquaculture Products using** **Liquid Chromatography/Quadrupole Time-of-Flight Mass Spectrometry**

Mingkai Bai ^a^, Ruixue Tang ^b^, Guorong Li ^c^, Wenhai She ^d^, Gangjun Chen ^d^, Hongmei Shen ^b^, Suqin Zhu ^c^, Hongwei Zhang ^f^*, Haohao Wu ^a^*

*^a^* *College of Food Science and Engineering,* *Ocean University of China, 5 Yushan Road, Qingdao, 266003, China*

*^b^ Linxia Food Inspection and Testing Center,* *8 Renmin Road, Linxia,* *731100, China*

*^c^ Yin-chuan Administration for Market Regulation, 205 South Limin Street, Yinchuan,* *750001, China*

*^d^* *Guangdong Aquatic Resources Industrialization Engineering Technology Research Center,* *Guangzhou Luxe Seafood Enterprises Ltd., 1 Lushi Road, Guangzhou,* *510820, China*

*^e^* *Institute of Nutrition and Health, School of Public Health,* *Qingdao University,* *308 Ningxia Road, Qingdao, 266021, China*

*^f^* *Technology Center of Qingdao Customs, 83 Xinyue Road, Qingdao 266109, China*

*Authors to whom correspondence should be addressed; E-mails: light04@126.com (H. Zhang), [wuhaohao@ouc.edu.cn](mailto:wuhaohao@ouc.edu.cn) (H. Wu).

**Table S1. The** **substances excluded from the database.**

| **Persistent Organic Pollutants (50)** | | |
| --- | --- | --- |
| No. | Compound | Reason |
| 1 | Acenaphthylene | Low ionization efficiency |
| 2 | Acenaphthene |  |
| 3 | Anthracene |  |
| 4 | Benz(a)anthracene |  |
| 5 | Benzo(a)pyrene |  |
| 6 | benzo(B)fluoranthene |  |
| 7 | Benzo[ghi]perylene |  |
| 8 | benzo(K)fluoranthene |  |
| 9 | Chrysene |  |
| 10 | Benzo[k]tetraphene |  |
| 11 | Fluoranthene |  |
| 12 | 9H fluorene |  |
| 13 | Indeno(1,2,3-cd)pyrene |  |
| 14 | Pyrene |  |
| 15 | Naphthalene |  |
| 16 | Phenanthrene |  |
| 17 | 5-Methylchrysene |  |
| 18 | Benz[j]fluoranthene |  |
| 19 | Dibenzochrysene |  |
| 20 | Dibenzo[a,l]pyrene |  |
| 21 | dibenzo[a,e]pyrene |  |
| 22 | Dibenz[a,i]pyrene |  |
| 23 | 2,4-Dibromo-1-(4-bromophenoxy)benzene |  |
| 24 | bis(2,4-dibromophenyl) ether |  |
| 25 | 1,3,5-Tribromo-2-(2,4-dibromophenoxy)benzene |  |
| 26 | 2,2',4,4',5-pentabromodiphenyl ether |  |
| 27 | 1,3,5-Tribromo-2-(2,4,5-tribromophenoxy)benzene |  |
| 28 | 2,2',4,4',5,5'-hexabromodiphenyl ether |  |
| 29 | 1,2,3,5-Tetrabromo-4-(2,4,5-tribromophenoxy)benzene |  |
| 30 | Decabromodiphenyl Oxide |  |
| 31 | 4,4'-Dichlorobiphenyl |  |
| 32 | 2,6-Dichlorobiphenyl |  |
| 33 | 2,4,6-Trichlorobiphenyl |  |
| 34 | 3,4,4'-Trichlorobiphenyl |  |
| 35 | 2,2',6,6'-Tetrachlorobiphenyl |  |
| 36 | 3,3',4,4'-Tetrachlorobiphenyl |  |
| 37 | 3,3',4,4',5-Pentachlorobiphenyl |  |
| 38 | 2,2',4,6,6'-Pentachlorobiphenyl |  |
| 39 | 3,3',4,4',5,5'-Hexachlorobiphenyl |  |
| 40 | 2,2',4,4',6,6'-Hexachlorobiphenyl |  |
| 41 | 2,3,3',4,4',5,5'-Heptachlorobiphenyl |  |
| 42 | 2,2',3,4',5,6,6'-Heptachlorobiphenyl |  |
| 43 | 2,2',3,3',4,4',5,5'-Octachlorobiphenyl |  |
| 44 | 2,2',3,3',5,5',6,6'-Octachlorobiphenyl |  |
| 45 | 2,2',3,3',4,4',5,5',6-Nonachlorobiphenyl |  |
| 46 | 2,2',3,3',4,5,5',6,6'-Nonachlorobiphenyl |  |
| 47 | Biphenyl | Only detected in negative ion mode |
| 48 | 2-Chlorobiphenyl |  |
| 49 | 4-Chlorobiphenyl |  |
| 50 | Decachlorobiphenyl |  |
| **Veterinary drugs (8)** | | |
| **No.** | **Compound name** | **Reason** |
| 1 | 3-Amino-2-oxazolidinone | Low ionization efficiency |
| 2 | Semicarbazide hydrochloride |  |
| 3 | Dihydrostreptomycin sulfate |  |
| 4 | Neomycin B sulfate |  |
| 5 | Streptomycin sulfate |  |
| 6 | Gentamicin sulfate salt |  |
| 7 | Nigericin sodium |  |
| 8 | Narasin |  |

**Table S2. Accurate mass database of the studied pesticides, veterinary drugs, persistent organic pollutants, and marine toxins, including** **elemental composition, retention time (t_R_), monoisotopic mass, ionization type, experimental mass and characteristic fragment ions for each database compound.**

| **Category No.** | **Compound** | **Elemental composition** | **t_R_  (min)** | **Monoisotopic mass** | **Ionization type** | **Precursor ion** | **Fragment ions** | | | |
| --- | --- | --- | --- | --- | --- | --- | --- | --- | --- | --- |
|  |  |  |  |  |  |  | **1^st^ most abundant** | **2^ed^ most abundant** | | **(CE, V)** |
| **Pesticides** | | | | | | | | | | |
| 1 | Allidochlor | C_8_H_12_ClNO | 6.433 | 173.0607 | [M+H]^+^ | 174.0404 | 158.0574 | | 134.0324 | 15 |
| 2 | Dichlormid | C_8_H_11_Cl_2_NO | 7.780 | 207.0218 | [M+H]^+^ | 208.0275 | 175.0517 | | 141.9671 | 18 |
| 3 | Etridiazole | C_5_H_5_Cl_3_N_2_OS | 15.609 | 245.9188 | [M+H]^+^ | 246.9691 | 216.9961 | | 182.0263 | 20 |
| 4 | Chlormephos | C_5_H_12_ClO_2_PS_2_ | 10.466 | 233.9705 | [M+NH_4_]^+^ | 250.9842 | 223.9720 | | 191.9388 | 20 |
| 5 | Propham | C_10_H_13_NO_2_ | 8.146 | 179.0946 | [M+H]^+^ | 180.1080 | 161.1157 | | 141.9374 | 15 |
| 6 | Cycloate | C_11_H_21_NOS | 15.539 | 215.1344 | [M+H]^+^ | 216.1112 | 192.8777 | | 154.1247 | 18 |
| 7 | Diphenylamine | C_12_H_11_N | 13.194 | 169.0891 | [M+H]^+^ | 170.0691 | 154.9985 | | 139.0076 | 15 |
| 8 | Chlordimeform | C_10_H_13_ClN_2_ | 4.745 | 196.0767 | [M+H]^+^ | 197.0548 | 169.1312 | | 152.0271 | 18 |
| 9 | Ethalfluralin | C_13_H_14_F_3_N_3_O_4_ | 17.660 | 333.0936 | [M+H]^+^ | 334.2734 | 317.2888 | | 137.1382 | 25 |
| 10 | Phorate | C_7_H_17_O_2_PS_3_ | 13.661 | 260.0128 | [M+H]^+^ | 260.9864 | 243.0677 | | 217.9727 | 22 |
| 11 | Thiometon | C_6_H_15_O_2_PS_3_ | 16.038 | 245.9972 | [M+H]^+^ | 246.9691 | 202.9173 | | 182.8726 | 22 |
| 12 | Quintozene | C_6_Cl_5_NO_2_ | 14.899 | 292.8372 | [M+H]^+^ | 294.0400 | 233.0173 | | 164.0506 | 23 |
| 13 | Atrazine-desethyl | C_6_H_10_ClN_5_ | 5.136 | 187.0625 | [M+H]^+^ | 188.0413 | 170.0673 | | 142.9630 | 15 |
| 14 | Clomazone | C_12_H_14_ClNO_2_ | 8.953 | 239.0713 | [M+H]^+^ | 240.0471 | 200.0987 | | 158.0519 | 20 |
| 15 | Diazinon | C_12_H_21_N_2_O_3_PS | 14.915 | 304.1011 | [M+H]^+^ | 305.0736 | 231.0401 | | 181.1352 | 22 |
| 16 | Fonofos | C_10_H_15_OPS_2_ | 15.581 | 246.0302 | [M+H]^+^ | 247.0052 | 209.0358 | | 168.9906 | 20 |
| 17 | Etrimfos | C_10_H_17_N_2_O_4_PS | 14.899 | 292.0647 | [M+H]^+^ | 293.0379 | 265.0436 | | 233.0173 | 22 |
| 18 | Simazine | C_7_H_12_ClN_5_ | 6.425 | 201.0781 | [M+H]^+^ | 202.0562 | 174.0561 | | 166.1106 | 18 |
| 19 | Propetamphos | C_10_H_20_NO_4_PS | 17.660 | 281.0851 | [M+H]^+^ | 282.1112 | 250.9720 | | 239.1301 | 22 |
| 20 | Secbumeton | C_10_H_19_N_5_O | 6.026 | 225.1590 | [M+H]^+^ | 226.1357 | 170.1059 | | 142.0743 | 20 |
| 21 | Dichlofenthion | C_10_H_13_Cl_2_O_3_PS | 17.244 | 313.9700 | [M+H]^+^ | 314.9763 | 210.1619 | | 164.0737 | 22 |
| 22 | Propyzamide | C_12_H_11_Cl_2_NO | 18.907 | 255.0218 | [M+H]^+^ | 256.2307 | 174.8642 | | 156.8922 | 22 |
| 23 | Mexacarbate | C_12_H_18_N_2_O_2_ | 3.439 | 222.1368 | [M+H]^+^ | 223.1134 | 166.1247 | | 151.1013 | 20 |
| 24 | Aldrin | C_12_H_8_Cl_6_ | 17.458 | 361.8757 | [M+H]^+^ | 363.0189 | 250.0226 | | 202.0163 | 25 |
| 25 | Dinitramine | C_11_H_13_F_3_N_4_O_4_ | 15.381 | 322.0889 | [M+H]^+^ | 323.0604 | 279.8282 | | 262.0684 | 25 |
| 26 | Fenchlorphos | C_8_H_8_Cl_3_O_3_PS | 13.518 | 319.8997 | [M+H]^+^ | 320.9131 | 277.9367 | | 262.0709 | 25 |
| 27 | Prometryn | C_10_H_19_N_5_S | 7.448 | 241.1361 | [M+H]^+^ | 242.1121 | 214.0843 | | 200.0975 | 22 |
| 28 | Cyprazine | C_9_H_14_ClN_5_ | 7.439 | 227.0938 | [M+H]^+^ | 228.0704 | 186.0565 | | 144.0339 | 20 |
| 29 | Vinclozolin | C_12_H_9_Cl_2_NO_3_ | 17.244 | 284.9959 | [M+H]^+^ | 286.1464 | 262.9529 | | 246.0512 | 22 |
| 30 | β-HCH | C_6_H_6_Cl_6_ | 17.235 | 287.8601 | [M+H]^+^ | 288.1604 | 249.1214 | | 219.1019 | 22 |
| 31 | Metalaxyl | C_15_H_21_NO_4_ | 7.838 | 279.1471 | [M+H]^+^ | 280.1210 | 264.7977 | | 191.8217 | 22 |
| 32 | Chlorpyrifos | C_9_H_11_Cl_3_NO_3_PS | 17.660 | 348.9263 | [M+H]^+^ | 349.8967 | 279.1446 | | 229.0781 | 25 |
| 33 | Parathion-methyl | C_8_H_10_NO_5_PS | 12.138 | 263.0017 | [M+H]^+^ | 263.9756 | 217.8781 | | 206.1304 | 22 |
| 34 | Anthraquinone | C_14_H_8_O_2_ | 7.789 | 208.0524 | [M+H]^+^ | 209.9961 | 192.9260 | | 170.1061 | 18 |
| 35 | δ-HCH | C_6_H_6_Cl_6_ | 11.389 | 287.8601 | [M+NH_4_]^+^ | 305.0811 | 293.7745 | | 268.8135 | 20 |
| 36 | Fenthion | C_10_H_15_O_3_PS_2_ | 15.559 | 278.0200 | [M+H]^+^ | 279.0257 | 271.7274 | | 220.1332 | 22 |
| 37 | Malathion | C_10_H_19_O_6_PS_2_ | 13.069 | 330.0361 | [M+H]^+^ | 331.0077 | 210.9328 | | 146.9666 | 25 |
| 38 | Fenitrothion | C_9_H_12_NO_5_PS | 8.612 | 277.0174 | [M+H]^+^ | 278.0721 | 236.0640 | | 220.1294 | 22 |
| 39 | Paraoxon-ethyl | C_10_H_14_NO_6_P | 8.221 | 275.0559 | [M+H]^+^ | 276.0301 | 235.1396 | | 201.8908 | 22 |
| 40 | Triadimefon | C_14_H_16_ClN_3_O_2_ | 11.739 | 293.0931 | [M+H]^+^ | 294.0662 | 282.0400 | | 249.0025 | 25 |
| 41 | Parathion | C_10_H_14_NO_5_PS | 15.098 | 291.0330 | [M+H]^+^ | 292.0068 | 264.9673 | | 219.9050 | 25 |
| 42 | Pendimethalin | C_13_H_19_N_3_O_4_ | 19.082 | 281.1376 | [M+H]^+^ | 282.2452 | 242.2740 | | 215.8885 | 25 |
| 43 | Linuron | C_9_H_10_Cl_2_N_2_O_2_ | 11.186 | 248.0119 | [M+H]^+^ | 249.0175 | 223.9788 | | 204.0030 | 22 |
| 44 | Chlorbenside | C_13_H_10_Cl_2_S | 7.993 | 267.9880 | [M+H]^+^ | 268.9840 | 254.2471 | | 227.8827 | 25 |
| 45 | Bromophos-ethyl | C_10_H_12_BrCl_2_O_3_PS | 18.533 | 391.8805 | [M+H]^+^ | 392.8500 | 358.2254 | | 332.7934 | 28 |
| 46 | Quinalphos | C_12_H_15_N_2_O_3_PS | 14.283 | 298.0541 | [M+H]^+^ | 299.0268 | 284.2882 | | 230.2393 | 25 |
| 47 | trans-Chlordane (γ) | C_10_H_6_Cl_8_ | 17.826 | 405.7978 | [M+H]^+^ | 406.9376 | 352.8719 | | 285.8125 | 30 |
| 48 | Phenthoate | C_12_H_17_O_4_PS_2_ | 14.300 | 320.0306 | [M+H]^+^ | 321.0077 | 290.7435 | | 262.0666 | 28 |
| 49 | Metazachlor | C_14_H_16_ClN_3_O | 15.115 | 277.0982 | [M+H]^+^ | 277.9861 | 245.9922 | | 231.8601 | 22 |
| 50 | Fenothiocarb | C_13_H_19_NO_2_S | 13.302 | 253.1137 | [M+H]^+^ | 254.0885 | 239.9944 | | 229.0505 | 22 |
| 51 | Prothiophos | C_11_H_15_Cl_2_O_2_PS_2_ | 13.576 | 343.9628 | [M+H]^+^ | 344.8378 | 261.7742 | | 253.1801 | 25 |
| 52 | Chlorflurenol-methyl | C_15_H_11_ClO_3_ | 15.306 | 274.0397 | [M+H]^+^ | 274.9620 | 257.2608 | | 233.8603 | 22 |
| 53 | Dieldrin | C_12_H_8_Cl_6_O | 18.657 | 377.8706 | [M+NH_4_]^+^ | 395.3175 | 338.8214 | | 306.8301 | 30 |
| 54 | Procymidone | C_13_H_11_Cl_2_NO_2_ | 13.077 | 283.0167 | [M+H]^+^ | 283.9675 | 271.1197 | | 240.7931 | 22 |
| 55 | Methidathion | C_6_H_11_N_2_O_4_PS_3_ | 10.350 | 301.9619 | [M+H]^+^ | 302.9338 | 284.0468 | | 241.1309 | 25 |
| 56 | Cyanazine | C_9_H_13_ClN_6_ | 6.666 | 240.0890 | [M+H]^+^ | 241.0649 | 214.0841 | | 205.1163 | 20 |
| 57 | Napropamide | C_17_H_21_NO_2_ | 12.071 | 271.1572 | [M+H]^+^ | 272.1310 | 254.9497 | | 224.9837 | 22 |
| 58 | Oxadiazon | C_15_H_18_Cl_2_N_2_O_3_ | 13.069 | 344.0694 | [M+NH_4_]^+^ | 362.0476 | 347.6925 | | 319.1748 | 25 |
| 59 | Fenamiphos | C_13_H_22_NO_3_PS | 11.381 | 303.1058 | [M+H]^+^ | 304.0785 | 284.0412 | | 260.1960 | 22 |
| 60 | Tetrasul | C_12_H_6_Cl_4_S | 16.038 | 321.8944 | [M+H]^+^ | 322.9936 | 305.0506 | | 261.0433 | 25 |
| 61 | Aramite | C_15_H_23_ClO_4_S | 17.443 | 334.1006 | [M+NH_4_]^+^ | 352.0971 | 329.1226 | | 312.7936 | 28 |
| 62 | Bupirimate | C_13_H_24_N_4_O_3_S | 9.543 | 316.1569 | [M+H]^+^ | 317.1292 | 272.1830 | | 237.2843 | 22 |
| 63 | Carboxin | C_12_H_13_NO_2_S | 7.705 | 235.0667 | [M+H]^+^ | 236.0427 | 221.1586 | | 202.9486 | 20 |
| 64 | Flutolanil | C_17_H_16_F_3_NO_2_ | 13.327 | 323.1133 | [M+H]^+^ | 324.0848 | 262.1478 | | 256.3274 | 25 |
| 65 | 4,4'-DDD | C_14_H_10_Cl_4_ | 9.543 | 317.9537 | [M+H]^+^ | 318.1318 | 256.3366 | | 237.2796 | 22 |
| 66 | Ethion | C_9_H_22_O_4_P_2_S_4_ | 17.826 | 383.9876 | [M+H]^+^ | 384.9567 | 325.4484 | | 296.9296 | 28 |
| 67 | Sulprofos | C_12_H_19_O_2_PS_3_ | 17.859 | 322.0285 | [M+H]^+^ | 323.0001 | 305.1696 | | 289.2969 | 25 |
| 68 | Etaconazole | C_14_H_15_Cl_2_N_3_O_2_ | 11.606 | 327.0541 | [M+H]^+^ | 328.0259 | 257.0852 | | 205.0521 | 25 |
| 69 | Myclobutanil | C_15_H_17_ClN_4_ | 11.689 | 288.1142 | [M+H]^+^ | 289.0877 | 272.8931 | | 221.1681 | 25 |
| 70 | Diclofop-methyl | C_16_H_14_Cl_2_O_4_ | 17.718 | 340.0269 | [M+NH_4_]^+^ | 358.3299 | 298.3835 | | 255.3103 | 28 |
| 71 | Propiconazol | C_15_H_17_Cl_2_N_3_O_2_ | 13.402 | 341.0698 | [M+H]^+^ | 342.0404 | 273.2138 | | 189.1263 | 25 |
| 72 | Fensulfothion | C_11_H_17_O_4_PS_2_ | 8.387 | 308.0306 | [M+H]^+^ | 309.0031 | 235.0368 | | 219.0563 | 22 |
| 73 | Bifenthrin | C_23_H_22_ClF_3_O_2_ | 18.608 | 422.1260 | [M+NH_4_]^+^ | 439.3420 | 349.8836 | | 319.6638 | 28 |
| 74 | Mirex | C_10_Cl_12_ | 19.223 | 539.6262 | [M+H]^+^ | 540.4026 | 362.2002 | | 311.3772 | 32 |
| 75 | Benodanil | C_13_H_10_INO | 10.359 | 322.9807 | [M+H]^+^ | 323.9870 | 262.1478 | | 256.3274 | 25 |
| 76 | Nuarimol | C_17_H_12_ClFN_2_O | 8.845 | 314.0622 | [M+H]^+^ | 315.0347 | 252.1575 | | 243.1105 | 22 |
| 77 | 4,4'-Methoxychlor | C_16_H_15_Cl_3_O_2_ | 17.585 | 344.0138 | [M+H]^+^ | 345.0399 | 329.2549 | | 303.3853 | 25 |
| 78 | Oxadixyl | C_14_H_18_N_2_O_4_ | 6.649 | 278.1267 | [M+H]^+^ | 279.1005 | 220.2031 | | 192.2047 | 22 |
| 79 | Tetramethrin | C_19_H_25_NO_4_ | 17.069 | 331.1784 | [M+H]^+^ | 332.1493 | 303.2247 | | 259.1715 | 25 |
| 80 | Tebuconazol | C_16_H_22_ClN_3_O | 12.312 | 307.1451 | [M+H]^+^ | 308.1177 | 235.8967 | | 209.0965 | 25 |
| 81 | Norflurazon | C_12_H_9_ClF_3_N_3_O | 8.221 | 303.0386 | [M+H]^+^ | 304.0111 | 284.1202 | | 264.1107 | 25 |
| 82 | Pyridaphenthion | C_14_H_17_N_2_O_4_PS | 12.221 | 340.0647 | [M+H]^+^ | 341.0356 | 267.0789 | | 217.1642 | 25 |
| 83 | Phosmet | C_11_H_12_NO_4_PS_2_ | 11.248 | 316.9945 | [M+H]^+^ | 317.9666 | 273.1865 | | 194.1975 | 25 |
| 84 | Tetradifon | C_12_H_6_Cl_4_O_2_S | 17.244 | 353.8843 | [M+H]^+^ | 354.1305 | 282.0610 | | 242.9593 | 25 |
| 85 | Oxycarboxin | C_12_H_13_NO_4_S | 6.192 | 267.0565 | [M+H]^+^ | 268.0309 | 209.1100 | | 187.0173 | 22 |
| 86 | cis-Permethrin | C_21_H_20_Cl_2_O_3_ | 20.329 | 390.0790 | [M+H]^+^ | 391.2458 | 322.3420 | | 305.2035 | 28 |
| 87 | trans-Permethrin | C_21_H_20_Cl_2_O_3_ | 19.306 | 390.0790 | [M+H]^+^ | 391.3027 | 362.4428 | | 322.3339 | 28 |
| 88 | Pyrazophos | C_14_H_20_N_3_O_5_PS | 15.256 | 373.0861 | [M+H]^+^ | 374.0556 | 304.8522 | | 280.2588 | 28 |
| 89 | Cypermethrin | C_22_H_19_Cl_2_NO_3_ | 20.645 | 415.0742 | [M+NH_4_]^+^ | 433.3490 | 376.1004 | | 316.2510 | 30 |
| 90 | Fenvalerate | C_25_H_22_ClNO_3_ | 18.732 | 419.1288 | [M+NH_4_]^+^ | 438.3382 | 349.2489 | | 329.4001 | 30 |
| 91 | Deltamethrin | C_22_H_19_Br_2_NO_3_ | 18.682 | 502.9732 | [M+NH_4_]^+^ | 520.9643 | 421.1741 | | 377.1754 | 32 |
| 92 | EPTC | C_9_H_19_NOS | 13.369 | 189.1187 | [M+H]^+^ | 190.0959 | 162.8675 | | 149.9039 | 12 |
| 93 | Butylate | C_11_H_23_NOS | 9.369 | 217.1500 | [M+H]^+^ | 217.9818 | 200.1143 | | 188.1050 | 15 |
| 94 | Dichlobenil | C_7_H_3_Cl_2_N | 12.139 | 170.9643 | [M+H]^+^ | 171.9880 | 168.9468 | | 150.9521 | 12 |
| 95 | Pebulate | C_10_H_21_NOS | 15.598 | 203.1344 | [M+H]^+^ | 204.1100 | 187.1151 | | 169.9683 | 15 |
| 96 | Nitrapyrin | C_6_H_3_Cl_4_N | 5.694 | 228.9020 | [M+H]^+^ | 229.1071 | 204.8064 | | 188.0563 | 15 |
| 97 | Mevinphos | C_7_H_13_O_6_P | 5.710 | 224.0450 | [M+Na]^+^ | 246.9993 | 233.1056 | | 220.8018 | 18 |
| 98 | Chloroneb | C_8_H_8_Cl_2_O_2_ | 14.500 | 205.9901 | [M+NH_4_]^+^ | 223.9163 | 205.9219 | | 180.9187 | 15 |
| 99 | Tecnazene | C_6_HCl_4_NO_2_ | 15.498 | 258.8761 | [M+H]^+^ | 260.0336 | 243.9065 | | 216.8292 | 15 |
| 100 | Heptenophos | C_9_H_12_ClO_4_P | 8.346 | 250.0162 | [M+H]^+^ | 250.9885 | 223.9730 | | 191.8215 | 18 |
| 101 | Hexachlorobenzene | C_6_Cl_6_ | 19.066 | 281.8131 | [M+H]^+^ | 282.2436 | 252.1146 | | 240.8335 | 18 |
| 102 | Ethoprophos | C_8_H_19_O_2_PS_2_ | 11.548 | 242.0564 | [M+H]^+^ | 243.0307 | 228.2226 | | 212.2403 | 18 |
| 103 | Diallate | C_10_H_17_Cl_2_NOS | 12.987 | 269.0408 | [M+H]^+^ | 270.0890 | 254.2467 | | 240.1227 | 18 |
| 104 | Propachlor | C_11_H_14_ClNO | 8.521 | 211.0764 | [M+H]^+^ | 212.0525 | 183.0296 | | 172.0639 | 15 |
| 105 | Triﬂuralin | C_13_H_16_F_3_N_3_O_4_ | 12.754 | 335.1093 | [M+H]^+^ | 336.0399 | 278.7602 | | 259.8006 | 20 |
| 106 | Chlorpropham | C_10_H_12_ClNO_2_ | 5.652 | 213.0557 | [M+H]^+^ | 214.0800 | 196.9498 | | 156.9750 | 15 |
| 107 | Sulfotep | C_8_H_20_O_5_P_2_S_2_ | 16.030 | 322.0227 | [M+H]^+^ | 322.9923 | 306.9033 | | 242.0582 | 20 |
| 108 | Sulfallate | C_8_H_14_ClNS_2_ | 8.754 | 223.0256 | [M+H]^+^ | 224.0518 | 208.0339 | | 170.1017 | 15 |
| 109 | α-BHC | C_6_H_6_Cl_6_ | 13.610 | 287.8601 | [M+H]^+^ | 288.9750 | 271.1733 | | 256.1156 | 18 |
| 110 | Terbufos | C_9_H_21_O_2_PS_3_ | 10.425 | 288.0441 | [M+Na]^+^ | 311.9562 | 257.9531 | | 248.0074 | 18 |
| 111 | Terbumeton | C_10_H_19_N_5_O | 6.060 | 225.1590 | [M+H]^+^ | 226.1332 | 186.0507 | | 170.1022 | 15 |
| 112 | Profluralin | C_14_H_16_F_3_N_3_O_4_ | 14.777 | 347.1093 | [M+H]^+^ | 348.2323 | 305.2272 | | 268.8024 | 20 |
| 113 | Dioxathion | C_12_H_26_O_6_P_2_S_4_ | 19.265 | 456.0087 | [M+H]^+^ | 457.3042 | 359.7183 | | 278.8032 | 28 |
| 114 | Propazine | C_9_H_16_ClN_5_ | 9.694 | 229.1094 | [M+H]^+^ | 230.0838 | 188.0686 | | 146.0221 | 15 |
| 115 | Chlorbufam | C_11_H_10_ClNO_2_ | 15.671 | 223.0400 | [M+H]^+^ | 224.0307 | 205.9219 | | 180.9187 | 15 |
| 116 | Dicloran | C_6_H_4_Cl_2_N_2_O_2_ | 5.694 | 205.9650 | [M+H]^+^ | 207.1273 | 191.0725 | | 173.0925 | 15 |
| 117 | Terbuthylazine | C_9_H_16_ClN_5_ | 9.028 | 229.1094 | [M+H]^+^ | 230.0836 | 204.8344 | | 188.0507 | 15 |
| 118 | Monolinuron | C_9_H_11_ClN_2_O_2_ | 7.806 | 214.0509 | [M+H]^+^ | 215.0257 | 188.0075 | | 172.0648 | 15 |
| 119 | Flufenoxuron | C_21_H_11_ClF_6_N_2_O_3_ | 17.752 | 488.0362 | [M+H]^+^ | 488.9954 | 415.6890 | | 296.0519 | 30 |
| 120 | Cyanophos | C_9_H_10_NO_3_PS | 6.176 | 243.0119 | [M+H]^+^ | 244.0880 | 198.9839 | | 185.8976 | 15 |
| 121 | Chlorpyrifos-methyl | C_7_H_7_Cl_3_NO_3_PS | 16.105 | 320.8950 | [M+H]^+^ | 321.8621 | 290.0674 | | 216.8547 | 20 |
| 122 | Desmetryn | C_8_H_15_N_5_S | 5.594 | 213.1048 | [M+H]^+^ | 214.0803 | 198.1881 | | 185.0101 | 15 |
| 123 | Dimethachlor | C_13_H_18_ClNO_2_ | 8.746 | 255.1026 | [M+H]^+^ | 256.0757 | 231.0396 | | 215.0919 | 18 |
| 124 | Alachlor | C_14_H_20_ClNO_2_ | 16.787 | 269.1183 | [M+H]^+^ | 270.0125 | 254.2460 | | 240.1243 | 18 |
| 125 | Pirimiphos-methyl | C_11_H_20_N_3_O_3_PS | 14.878 | 305.0963 | [M+H]^+^ | 306.1011 | 263.0058 | | 189.3505 | 20 |
| 126 | Terbutryn | C_10_H_19_N_5_S | 7.315 | 241.1361 | [M+H]^+^ | 242.1100 | 214.1811 | | 186.1442 | 15 |
| 127 | Thiobencarb | C_12_H_16_ClNOS | 15.490 | 257.0641 | [M+H]^+^ | 258.0356 | 227.9826 | | 207.9357 | 18 |
| 128 | Aspon | C_12_H_28_O_5_P_2_S_2_ | 18.575 | 378.0853 | [M+H]^+^ | 379.0514 | 363.2813 | | 279.2982 | 25 |
| 129 | Dicofol | C_14_H_9_Cl_5_O | 16.272 | 367.9096 | [M+H]^+^ | 368.9533 | 323.3168 | | 281.1960 | 25 |
| 130 | Metolachlor | C_15_H_22_ClNO_2_ | 12.771 | 283.1339 | [M+H]^+^ | 284.1052 | 245.9590 | | 197.1533 | 20 |
| 131 | Oxychlordane | C_10_H_4_Cl_8_O | 18.633 | 419.7770 | [M+H]^+^ | 421.3066 | 266.9042 | | 237.0745 | 28 |
| 132 | Pirimiphos-ethyl | C_13_H_24_N_3_O_3_PS | 16.795 | 333.1276 | [M+H]^+^ | 334.1319 | 310.7950 | | 295.2772 | 22 |
| 133 | Methoprene | C_19_H_34_O_3_ | 13.793 | 310.2508 | [M+H]^+^ | 310.9936 | 178.1059 | | 162.1262 | 20 |
| 134 | Bromophos | C_8_H_8_BrCl_2_O_3_PS | 13.195 | 363.8492 | [M+H]^+^ | 364.8648 | 323.3183 | | 234.9627 | 25 |
| 135 | Dichlofluanid | C_9_H_11_Cl_2_FN_2_O_2_S_2_ | 14.492 | 331.9623 | [M+H]^+^ | 332.9277 | 295.2797 | | 269.1289 | 20 |
| 136 | Ethofumesate | C_13_H_18_O_5_S | 16.588 | 286.0875 | [M+H]^+^ | 287.0132 | 248.9267 | | 232.9387 | 20 |
| 137 | Isopropalin | C_15_H_23_N_3_O_4_ | 18.517 | 309.1689 | [M+H]^+^ | 310.1374 | 283.0802 | | 272.0903 | 20 |
| 138 | α-Endosulfan | C_9_H_6_Cl_6_O_3_S | 10.010 | 403.8169 | [M+H]^+^ | 404.1636 | 369.8779 | | 355.1439 | 28 |
| 139 | Propanil | C_9_H_9_Cl_2_NO | 16.696 | 217.0061 | [M+H]^+^ | 218.1245 | 196.9365 | | 162.0442 | 18 |
| 140 | Isofenphos | C_15_H_24_NO_4_PS | 13.602 | 345.1164 | [M+H]^+^ | 346.0045 | 300.1199 | | 216.2102 | 22 |
| 141 | Crufomate | C_12_H_19_ClNO_3_P | 11.698 | 291.0791 | [M+H]^+^ | 292.0489 | 205.9868 | | 182.0881 | 20 |
| 142 | Chlorfenvinphos | C_12_H_14_Cl_3_O_4_P | 14.126 | 357.9695 | [M+H]^+^ | 358.9359 | 321.0383 | | 292.0905 | 22 |
| 143 | cis-Chlordane (α) | C_10_H_6_Cl_8_ | 19.681 | 405.7978 | [M+H]^+^ | 406.3092 | 359.3001 | | 250.2464 | 28 |
| 144 | Tolylfluanid | C_10_H_13_Cl_2_FN_2_O_2_S_2_ | 15.872 | 345.9780 | [M+H]^+^ | 346.9444 | 287.8814 | | 214.9682 | 22 |
| 145 | 4,4'-DDE | C_14_H_8_Cl_4_ | 12.895 | 315.9380 | [M+H]^+^ | 317.0425 | 300.3694 | | 256.3388 | 20 |
| 146 | Butachlor | C_17_H_26_ClNO_2_ | 17.444 | 311.1652 | [M+H]^+^ | 312.1350 | 238.1723 | | 162.1876 | 20 |
| 147 | Chlozolinate | C_13_H_11_Cl_2_NO_5_ | 10.725 | 331.0014 | [M+H]^+^ | 332.0860 | 303.1151 | | 246.9010 | 20 |
| 148 | Crotoxyphos | C_14_H_19_O_6_P | 8.172 | 314.0919 | [M+H]^+^ | 315.2624 | 272.1915 | | 221.0481 | 20 |
| 149 | Iodofenphos | C_8_H_8_Cl_2_IO_3_PS | 16.255 | 411.8354 | [M+H]^+^ | 413.0074 | 363.2995 | | 324.9062 | 28 |
| 150 | Z-Tetrachlorvinphos | C_10_H_9_Cl_4_O_4_P | 15.664 | 363.8993 | [M+H]^+^ | 364.9775 | 301.3171 | | 244.8711 | 25 |
| 151 | Chlorbromuron | C_9_H_10_BrClN_2_O_2_ | 11.016 | 291.9614 | [M+H]^+^ | 292.9303 | 282.7844 | | 256.2172 | 22 |
| 152 | Profenofos | C_11_H_15_BrClO_3_PS | 16.405 | 371.9351 | [M+H]^+^ | 372.9009 | 304.8587 | | 287.9074 | 25 |
| 153 | Flurochloridone | C_12_H_10_Cl_2_F_3_NO | 13.494 | 311.0092 | [M+H]^+^ | 311.9783 | 178.0424 | | 162.0653 | 20 |
| 154 | Buprofezin | C_16_H_23_N_3_OS | 14.101 | 305.1562 | [M+H]^+^ | 306.0653 | 228.8908 | | 208.1243 | 20 |
| 155 | 2,4'-DDD | C_14_H_10_Cl_4_ | 10.708 | 317.9537 | [M+NH_4_]^+^ | 337.0404 | 310.0559 | | 296.4127 | 20 |
| 156 | Endrin | C_12_H_8_Cl_6_O | 16.396 | 377.8706 | [M+H]^+^ | 378.8919 | 337.2387 | | 295.2700 | 25 |
| 157 | Hexaconazole | C_14_H_17_Cl_2_N_3_O | 13.236 | 313.0749 | [M+H]^+^ | 314.0335 | 185.0559 | | 159.0354 | 20 |
| 158 | Chlorfenson | C_12_H_8_Cl_2_O_3_S | 18.201 | 301.9571 | [M+H]^+^ | 303.2117 | 286.7871 | | 164.1796 | 20 |
| 159 | 2,4'-DDT | C_14_H_9_Cl_5_ | 19.282 | 351.9147 | [M+H]^+^ | 353.2248 | 335.3856 | | 273.1916 | 24 |
| 160 | Paclobutrazol | C_15_H_20_ClN_3_O | 9.544 | 293.1295 | [M+H]^+^ | 294.0991 | 252.9544 | | 203.8705 | 18 |
| 161 | Methoprotryne | C_11_H_21_N_5_OS | 6.475 | 271.1467 | [M+H]^+^ | 272.1178 | 240.2010 | | 230.1789 | 18 |
| 162 | Erbon | C_11_H_9_Cl_5_O_3_ | 15.390 | 363.8994 | [M+H]^+^ | 365.0721 | 244.8711 | | 203.9973 | 25 |
| 163 | Chloropropylate | C_17_H_16_Cl_2_O_3_ | 20.754 | 338.0477 | [M+H]^+^ | 339.2398 | 296.4115 | | 97.1471 | 22 |
| 164 | Flamprop-methyl | C_17_H_15_ClFNO_3_ | 20.014 | 335.0724 | [M+H]^+^ | 336.2825 | 321.3948 | | 200.1696 | 22 |
| 165 | Nitrofen | C_12_H_7_Cl_2_NO_3_ | 19.914 | 282.9803 | [M+H]^+^ | 284.2574 | 252.1915 | | 176.2056 | 18 |
| 166 | Oxyﬂuorfen | C_15_H_11_ClF_3_NO_4_ | 17.444 | 361.0329 | [M+H]^+^ | 361.9988 | 319.0437 | | 275.0428 | 25 |
| 167 | Chlorthiophos | C_11_H_15_Cl_2_O_3_PS_2_ | 14.126 | 359.9577 | [M+H]^+^ | 360.9323 | 327.8405 | | 292.0904 | 25 |
| 168 | β-Endosulfan | C_9_H_6_Cl_6_O_3_S | 19.681 | 403.8169 | [M+H]^+^ | 405.3136 | 392.3205 | | 293.2153 | 28 |
| 169 | Flamprop-isopropyl | C_19_H_19_ClFNO_3_ | 15.390 | 363.1037 | [M+H]^+^ | 364.0688 | 321.3113 | | 205.9939 | 26 |
| 170 | 4,4'-DDT | C_14_H_9_Cl_5_ | 16.272 | 351.9147 | [M+NH_4_]^+^ | 369.9486 | 349.1212 | | 278.9159 | 27 |
| 171 | Carbophenothion | C_11_H_16_ClO_2_PS_3_ | 17.419 | 341.9739 | [M+H]^+^ | 343.1728 | 290.2010 | | 228.1382 | 25 |
| 172 | Benalaxyl | C_20_H_23_NO_3_ | 14.384 | 325.1678 | [M+H]^+^ | 326.1368 | 274.9741 | | 250.9192 | 22 |
| 173 | Edifenphos | C_14_H_15_O_2_PS_2_ | 19.922 | 310.0251 | [M]^+^ | 310.2712 | 283.0835 | | 256.3374 | 22 |
| 174 | Triazophos | C_12_H_16_N_3_O_3_PS | 12.887 | 313.0650 | [M+H]^+^ | 314.0437 | 288.1091 | | 256.3420 | 22 |
| 175 | Cyanofenphos | C_15_H_14_NO_2_PS | 12.480 | 303.0483 | [M+H]^+^ | 304.0840 | 220.8744 | | 201.1725 | 21 |
| 176 | Chlorbenside sulfone | C_13_H_10_Cl_2_O_2_S | 10.542 | 299.9779 | [M+NH_4_]^+^ | 317.9737 | 300.3779 | | 279.2886 | 22 |
| 177 | Endosulfan Sulfate | C_9_H_6_Cl_6_O_4_S | 20.147 | 419.8118 | [M+Na]^+^ | 443.2890 | 427.3012 | | 274.9013 | 28 |
| 178 | Bromopropylate | C_17_H_16_Br_2_O_3_ | 19.689 | 425.9466 | [M+H]^+^ | 427.2950 | 281.0461 | | 147.0636 | 28 |
| 179 | Benzoylprop-ethyl | C_18_H_17_Cl_2_NO_3_ | 15.540 | 365.0585 | [M+H]^+^ | 366.0240 | 302.8798 | | 262.0149 | 25 |
| 180 | Fenpropathrin | C_22_H_23_NO_3_ | 18.359 | 349.1678 | [M+H]^+^ | 350.1343 | 323.3046 | | 248.8543 | 25 |
| 181 | Leptophos | C_13_H_10_BrCl_2_O_2_PS | 17.627 | 409.8700 | [M+H]^+^ | 410.8964 | 365.8840 | | 286.9321 | 27 |
| 182 | EPN | C_14_H_14_NO_4_PS | 16.679 | 323.0381 | [M+H]^+^ | 324.0058 | 293.9568 | | 252.9820 | 22 |
| 183 | Hexazinone | C_12_H_20_N_4_O_2_ | 6.276 | 252.1586 | [M+H]^+^ | 253.1322 | 226.2374 | | 182.1682 | 18 |
| 184 | Phosalone | C_12_H_15_ClNO_4_PS_2_ | 16.596 | 366.9869 | [M+H]^+^ | 368.0638 | 352.8509 | | 306.9140 | 25 |
| 185 | Azinphos-methyl | C_10_H_12_N_3_O_3_PS_2_ | 10.550 | 317.0058 | [M+H]^+^ | 317.9740 | 272.1608 | | 239.3927 | 22 |
| 186 | Fenarimol | C_17_H_12_Cl_2_N_2_O | 10.924 | 330.0327 | [M+H]^+^ | 330.9998 | 311.3882 | | 248.3679 | 22 |
| 187 | Azinphos-ethyl | C_12_H_16_N_3_O_3_PS_2_ | 13.602 | 345.0371 | [M+H]^+^ | 346.0045 | 318.3010 | | 304.2941 | 22 |
| 188 | Prochloraz | C_15_H_16_Cl_3_N_3_O_2_ | 10.425 | 375.0308 | [M+H]^+^ | 375.9951 | 369.8046 | | 285.8411 | 25 |
| 189 | Coumaphos | C_14_H_16_ClO_5_PS | 16.147 | 362.0145 | [M+H]^+^ | 363.0184 | 320.2808 | | 232.9256 | 24 |
| 190 | Cyfluthrin | C_22_H_18_Cl_2_FNO_3_ | 18.597 | 433.0648 | [M+H]^+^ | 434.0673 | 274.8997 | | 208.0562 | 28 |
| 191 | tau-Fluvalinate | C_26_H_22_ClF_3_N_2_O_3_ | 18.949 | 502.1271 | [M+H]^+^ | 503.0855 | 427.4320 | | 364.8077 | 32 |
| 192 | Dichlorvos | C_4_H_7_Cl_2_O_4_P | 6.769 | 219.9459 | [M+H]^+^ | 220.9244 | 204.8894 | | 192.0708 | 18 |
| 193 | Biphenyl | C_6_H_5_C_6_H_5_ | 16.956 | 154.0783 | [M+Na]^+^ | 177.0647 | 149.1169 | | 119.1365 | 15 |
| 194 | Vernolate | C_10_H_21_NOS | 15.526 | 203.1344 | [M+H]^+^ | 204.1141 | 162.0954 | | 145.0639 | 16 |
| 195 | 3,5-Dichloroaniline | C_6_H_5_Cl_2_N | 9.779 | 160.9799 | [M+H]^+^ | 161.9613 | 144.9685 | | 132.1306 | 12 |
| 196 | Molinate | C_9_H_17_NOS | 10.736 | 187.1031 | [M+H]^+^ | 188.0832 | 173.0888 | | 159.0709 | 118 |
| 197 | E-Methacrifos | C_7_H_13_O_5_PS | 8.964 | 240.0221 | [M+H]^+^ | 241.1117 | 186.1439 | | 167.1458 | 18 |
| 198 | o-Phenylphenol | C_12_H_10_O | 5.023 | 170.0732 | [M+Na]^+^ | 193.0226 | 178.9685 | | 149.0796 | 121 |
| 199 | cis-1,2,3,6-Tetrahydrophthalimide | C_8_H_9_NO_2_ | 10.004 | 151.0633 | [M+H]^+^ | 152.0464 | 142.9889 | | 127.1076 | 12 |
| 200 | Fenobucarb | C_12_H_17_NO_2_ | 10.012 | 207.1259 | [M+H]^+^ | 208.1052 | 191.9645 | | 170.9084 | 16 |
| 201 | Benﬂuralin | C_13_H_16_F_3_N_3_O_4_ | 16.449 | 329.0521 | [M+H]^+^ | 330.3035 | 308.1583 | | 216.2090 | 25 |
| 202 | Hexaflumuron | C_16_H_8_Cl_2_F_6_N_2_O_3_ | 18.602 | 459.9816 | [M+Na]^+^ | 482.3678 | 463.2976 | | 311.3784 | 32 |
| 203 | Prometon | C_10_H_19_N_5_O | 6.020 | 225.1590 | [M+H]^+^ | 226.1377 | 184.1830 | | 110.1205 | 18 |
| 204 | Triallate | C_10_H_16_Cl_3_NOS | 17.945 | 303.0018 | [M+H]^+^ | 304.0083 | 263.9578 | | 221.0029 | 18 |
| 205 | Pyrimethanil | C_12_H_13_N_3_ | 6.944 | 199.1109 | [M+H]^+^ | 200.1178 | 183.0897 | | 107.0600 | 16 |
| 206 | γ-HCH | C_6_H_6_Cl_6_ | 19.933 | 288.0949 | [M+Na]^+^ | 310.2770 | 252.1900 | | 188.1714 | 20 |
| 207 | Disulfoton | C_8_H_19_O_2_PS_3_ | 10.445 | 274.0285 | [M]^+^ | 274.2430 | 256.3467 | | 228.1572 | 21 |
| 208 | Atrazine | C_8_H_14_ClN_5_ | 7.451 | 215.0938 | [M+H]^+^ | 216.0726 | 198.2528 | | 187.1662 | 18 |
| 209 | Heptachlor | C_10_H_5_Cl_7_ | 19.376 | 369.8211 | [M+H]^+^ | 371.0653 | 329.2893 | | 281.1279 | 26 |
| 210 | Iprobenfos | C_13_H_21_O_3_PS | 12.756 | 288.0949 | [M+H]^+^ | 289.0704 | 265.8999 | | 249.1544 | 21 |
| 211 | Isazofos | C_9_H_17_ClN_3_O_3_PS | 14.569 | 313.0417 | [M+H]^+^ | 314.1051 | 244.0449 | | 216.0085 | 22 |
| 212 | Plifenate | C_10_H_7_Cl_5_O_2_ | 14.711 | 333.8889 | [M+NH_4_]^+^ | 352.1164 | 338.4259 | | 320.3004 | 24 |
| 213 | Fenpropimorph | C_20_H_33_NO | 8.756 | 303.2562 | [M+H]^+^ | 304.2305 | 189.2283 | | 147.1733 | 20 |
| 214 | Transfluthrin | C_15_H_12_Cl_2_F_4_O_2_ | 17.006 | 370.0150 | [M+NH_4_]^+^ | 388.3085 | 345.2446 | | 330.4085 | 25 |
| 215 | Fluchloralin | C_12_H_13_ClF_3_N_3_O_4_ | 16.956 | 355.0547 | [M+H]^+^ | 356.2089 | 338.4332 | | 309.1450 | 25 |
| 216 | Tolclofos-methyl | C_9_H_11_Cl_2_O_3_PS | 16.008 | 299.9544 | [M+H]^+^ | 300.9293 | 282.3472 | | 212.3033 | 22 |
| 217 | Propisochlor | C_15_H_22_ClNO_2_ | 12.831 | 283.1339 | [M+H]^+^ | 284.0403 | 269.1941 | | 255.0599 | 20 |
| 218 | Ametryn | C_9_H_17_N_5_S | 6.594 | 227.1205 | [M+H]^+^ | 228.0990 | 186.1451 | | 158.1094 | 18 |
| 219 | Simetryn | C_8_H_15_N_5_S | 5.779 | 213.1048 | [M+H]^+^ | 214.0841 | 186.1441 | | 166.1688 | 18 |
| 220 | Metobromuron | C_9_H_11_BrN_2_O_2_ | 8.291 | 258.0004 | [M+H]^+^ | 258.9770 | 192.9683 | | 173.9315 | 20 |
| 221 | Metribuzin | C_8_H_14_N_4_OS | 6.736 | 214.0888 | [M+H]^+^ | 215.0680 | 198.2459 | | 166.1654 | 18 |
| 222 | Dimethipin | C_6_H_10_O_4_S_2_ | 14.104 | 210.0021 | [M+NH_4_]^+^ | 227.9693 | 221.1869 | | 198.1596 | 18 |
| 223 | ε-HCH | C_6_H_6_Cl_6_ | 7.594 | 287.8601 | [M+H]^+^ | 288.7483 | 275.8023 | | 189.0448 | 20 |
| 224 | Dipropetryn | C_11_H_21_N_5_S | 8.673 | 255.1518 | [M+H]^+^ | 256.1291 | 228.1988 | | 214.1809 | 20 |
| 225 | Formothion | C_6_H_12_NO_4_PS_2_ | 12.248 | 256.9945 | [M+H]^+^ | 258.2435 | 251.9917 | | 235.0964 | 20 |
| 226 | Diethofencarb | C_14_H_21_NO_4_ | 10.270 | 267.1471 | [M+H]^+^ | 268.1230 | 226.1761 | | 197.1357 | 20 |
| 227 | Dimepiperate | C_15_H_21_NOS | 12.831 | 263.1344 | [M+Na]^+^ | 286.0371 | 264.8843 | | 246.1324 | 21 |
| 228 | Bioallethrin | C_19_H_26_O_3_ | 17.405 | 302.1882 | [M+H]^+^ | 303.1632 | 272.0212 | | 261.0723 | 22 |
| 229 | 2,4'-DDE | C_14_H_8_Cl_4_ | 16.349 | 315.9380 | [M+NH_4_]^+^ | 334.1208 | 295.2802 | | 289.2332 | 22 |
| 230 | Fenson | C_12_H_9_ClO_3_S | 14.187 | 267.9961 | [M+H]^+^ | 268.9724 | 248.9025 | | 228.1722 | 20 |
| 231 | Diphenamid | C_16_H_17_NO | 8.981 | 239.1310 | [M+H]^+^ | 240.1081 | 231.8838 | | 205.9873 | 18 |
| 232 | Chlorthion | C_8_H_9_ClNO_5_PS | 9.739 | 296.9628 | [M+H]^+^ | 298.0813 | 282.1865 | | 264.1051 | 22 |
| 233 | Prallethrin | C_19_H_24_O_3_ | 16.523 | 300.1725 | [M+H]^+^ | 301.1474 | 269.0131 | | 175.0329 | 22 |
| 234 | Penconazole | C_13_H_15_Cl_2_N_3_ | 19.925 | 283.0643 | [M+H]^+^ | 284.2624 | 251.1152 | | 215.9583 | 21 |
| 235 | Mecarbam | C_10_H_20_NO_5_PS_2_ | 14.104 | 329.0521 | [M+H]^+^ | 330.0254 | 304.0700 | | 284.0643 | 20 |
| 236 | Tetraconazole | C_13_H_11_Cl_2_F_4_N_3_O | 12.507 | 371.0215 | [M+H]^+^ | 371.9936 | 359.8096 | | 313.4175 | 25 |
| 237 | Propaphos | C_13_H_21_O_4_PS | 13.530 | 304.0898 | [M+H]^+^ | 305.0646 | 280.8944 | | 262.2952 | 20 |
| 238 | Flumetralin | C_16_H_12_ClF_4_N_3_O_4_ | 18.602 | 421.0452 | [M+NH_4_]^+^ | 438.3424 | 412.2501 | | 376.3821 | 32 |
| 239 | Triadimenol | C_14_H_18_ClN_3_O_2_ | 9.663 | 295.1088 | [M+H]^+^ | 296.0844 | 255.9542 | | 218.9134 | 20 |
| 240 | Pretilachlor | C_17_H_26_ClNO_2_ | 16.340 | 311.1652 | [M+H]^+^ | 312.1398 | 287.2517 | | 162.1089 | 20 |
| 241 | Kresoxim-methyl | C_18_H_19_NO_4_ | 13.921 | 313.1314 | [M+H]^+^ | 314.0161 | 295.0871 | | 272.1495 | 20 |
| 242 | Fluazifop-butyl | C_19_H_20_F_3_NO_4_ | 17.505 | 383.1344 | [M+H]^+^ | 384.1070 | 325.4670 | | 282.1576 | 25 |
| 243 | Chlorfluazuron | C_20_H_9_Cl_3_F_5_N_3_O_3_ | 18.054 | 538.9630 | [M+H]^+^ | 539.9304 | 509.7481 | | 378.0613 | 30 |
| 244 | Chlorobenzilate | C_16_H_14_Cl_2_O_3_ | 17.413 | 324.0320 | [M+H]^+^ | 325.1443 | 256.3373 | | 239.2084 | 22 |
| 245 | Uniconazole | C_15_H_18_ClN_3_O | 11.434 | 291.1138 | [M+H]^+^ | 292.0894 | 218.1174 | | 170.1695 | 20 |
| 246 | Flusilazole | C_16_H_15_F_2_N_3_Si | 12.781 | 315.1003 | [M+H]^+^ | 316.0745 | 279.9424 | | 272.3329 | 20 |
| 247 | Fluorodifen | C_13_H_7_F_3_N_2_O_5_ | 14.004 | 328.0307 | [M+NH_4_]^+^ | 346.2959 | 328.4150 | | 304.8463 | 25 |
| 248 | Diniconazole | C_15_H_17_Cl_2_N_3_O | 16.232 | 325.0749 | [M+H]^+^ | 326.1454 | 308.0933 | | 233.5621 | 20 |
| 249 | Piperonyl butoxide | C_19_H_30_O_5_ | 13.189 | 337.1790 | [M+H]^+^ | 338.1528 | 296.2727 | | 270.0424 | 22 |
| 250 | Propargite | C_19_H_26_O_4_S | 18.095 | 350.1552 | [M+NH_4_]^+^ | 368.1547 | 163.0757 | | 253.1381 | 25 |
| 251 | Mepronil | C_17_H_19_NO_2_ | 12.698 | 269.1416 | [M+H]^+^ | 270.1181 | 254.3290 | | 198.1481 | 18 |
| 252 | Dimefuron | C_15_H_19_ClN_4_O_3_ | 9.097 | 338.1146 | [M+H]^+^ | 339.0879 | 297.2801 | | 265.0954 | 25 |
| 253 | Diﬂufenican | C_19_H_11_F_5_N_2_O_2_ | 16.432 | 394.0741 | [M+H]^+^ | 395.0459 | 372.8028 | | 340.8447 | 28 |
| 254 | Fenazaquin | C_20_H_22_N_2_O | 17.363 | 306.1732 | [M+H]^+^ | 307.1486 | 161.1921 | | 147.1122 | 22 |
| 255 | Phenothrin | C_23_H_26_O_3_ | 18.960 | 350.1882 | [M+H]^+^ | 351.1611 | 308.1292 | | 252.1354 | 25 |
| 256 | Fludioxonil | C_12_H_6_F_2_N_2_O_2_ | 11.102 | 248.0397 | [M+NH_4_]^+^ | 266.0424 | 225.1697 | | 180.1635 | 18 |
| 257 | Fenoxycarb | C_17_H_19_NO_4_ | 13.447 | 301.1314 | [M+H]^+^ | 302.1064 | 228.8932 | | 172.9230 | 20 |
| 258 | Sethoxydim | C_17_H_29_NO_3_S | 17.064 | 327.1868 | [M+H]^+^ | 328.1612 | 310.3985 | | 252.2820 | 22 |
| 259 | Anilofos | C_13_H_19_ClNO_3_PS_2_ | 15.160 | 367.0233 | [M+H]^+^ | 367.9958 | 358.7484 | | 323.3232 | 25 |
| 260 | Acrinathrin | C_26_H_21_F_6_NO_5_ | 18.735 | 541.1324 | [M+Na]^+^ | 564.0797 | 181.1282 | | 370.1670 | 35 |
| 261 | λ-Cyhalothrin | C_23_H_19_ClF_3_NO_3_ | 17.139 | 449.1006 | [M+NH_4_]^+^ | 467.0268 | 406.0524 | | 339.2205 | 30 |
| 262 | Mefenacet | C_16_H_14_N_2_O_2_S | 11.567 | 298.0776 | [M+H]^+^ | 299.0527 | 262.9895 | | 215.9807 | 20 |
| 263 | Permethrin | C_21_H_20_Cl_2_O_3_ | 20.315 | 390.0790 | [M+H]^+^ | 391.2476 | 375.9186 | | 280.8066 | 25 |
| 264 | Pyridaben | C_19_H_25_ClN_2_OS | 18.403 | 364.1376 | [M+H]^+^ | 365.1107 | 294.0866 | | 343.2858 | 25 |
| 265 | Fluoroglycofen-ethyl | C_18_H_13_ClF_3_NO_7_ | 17.139 | 447.0333 | [M+NH_4_]^+^ | 465.0288 | 378.2861 | | 342.7921 | 32 |
| 266 | Bitertanol | C_20_H_23_N_3_O_2_ | 20.773 | 337.1790 | [M+H]^+^ | 338.3073 | 296.4147 | | 261.3289 | 25 |
| 267 | Etofenprox | C_25_H_28_O_3_ | 19.035 | 376.2038 | [M+NH_4_]^+^ | 394.2009 | 321.2398 | | 303.2238 | 28 |
| 268 | Cycloxydim | C_17_H_27_NO_3_S | 13.488 | 325.1712 | [M+H]^+^ | 326.0488 | 298.8362 | | 266.1155 | 20 |
| 269 | α-Cypermethrin | C_22_H_19_Cl_2_NO_3_ | 18.611 | 415.0742 | [M+Na]^+^ | 438.3425 | 374.3613 | | 351.1811 | 30 |
| 270 | Flucythrinate | C_26_H_23_F_2_NO_4_ | 18.203 | 451.1595 | [M+NH_4_]^+^ | 469.1553 | 414.8474 | | 317.9856 | 32 |
| 271 | Esfenvalerate | C_25_H_22_ClNO_3_ | 18.727 | 419.1288 | [M+NH_4_]^+^ | 437.1242 | 411.2815 | | 391.3531 | 32 |
| 272 | Difenoconazole | C_19_H_17_Cl_2_N_3_O_3_ | 14.744 | 405.0647 | [M+H]^+^ | 406.0358 | 390.7809 | | 377.7888 | 30 |
| 273 | Flumioxazin | C_19_H_15_FN_2_O_4_ | 10.852 | 354.1016 | [M+H]^+^ | 355.0738 | 299.1651 | | 327.1973 | 25 |
| 274 | Flumiclorac-pentyl | C_21_H_23_ClFNO_5_ | 17.355 | 423.1249 | [M+NH_4_]^+^ | 441.1216 | 418.8171 | | 388.7836 | 30 |
| 275 | Dimefox | C_4_H_12_FN_2_OP | 4.057 | 154.0671 | [M+H]^+^ | 155.0493 | 138.9458 | | 127.9545 | 12 |
| 276 | Disulfoton-sulfoxide | C_8_H_19_O_3_PS_3_ | 7.516 | 290.0234 | [M+H]^+^ | 290.9978 | 267.7140 | | 257.0441 | 25 |
| 277 | Pentachlorobenzene | C_6_HCl_5_ | 15.209 | 247.8521 | [M+H]^+^ | 248.9925 | 226.8295 | | 176.0107 | 20 |
| 278 | Triisobutyl phosphate | C_12_H_27_O_4_P | 14.768 | 266.1647 | [M+H]^+^ | 267.1403 | 223.8727 | | 200.0834 | 22 |
| 279 | Crimidine | C_7_H_10_ClN_3_ | 4.489 | 171.0563 | [M+H]^+^ | 172.0371 | 136.0857 | | 119.0592 | 15 |
| 280 | BDMC-1 | C_10_H_12_BrNO_2_ | 18.194 | 257.0051 | [M+Na]^+^ | 280.2303 | 253.1226 | | 221.8536 | 22 |
| 281 | Chlorfenprop-methyl | C_10_H_10_Cl_2_O_2_ | 18.111 | 232.0058 | [M+Na]^+^ | 254.9413 | 229.8528 | | 195.8976 | 22 |
| 282 | Thionazin | C_8_H_13_N_2_O_3_PS | 9.138 | 248.0385 | [M+H]^+^ | 249.0149 | 223.9716 | | 188.6545 | 22 |
| 283 | 2,3,5,6-tetrachloroaniline | C_6_H_3_Cl_4_N | 10.543 | 228.9020 | [M+H]^+^ | 229.9993 | 174.0545 | | 188.0524 | 22 |
| 284 | Tributyl phosphate | C_12_H_27_O_4_P | 15.075 | 266.1647 | [M+H]^+^ | 267.1398 | 225.8311 | | 200.0843 | 22 |
| 285 | 2,3,4,5-Tetrachloroanisole | C_7_H_4_Cl_4_O | 19.562 | 243.9016 | [M+H]^+^ | 245.1963 | 226.2199 | | 191.1402 | 22 |
| 286 | Pentachloroanisole | C_7_H_3_Cl_5_O | 7.734 | 277.8627 | [M+NH_4_]^+^ | 295.0177 | 272.8164 | | 230.8457 | 25 |
| 287 | Tebutam | C_15_H_23_NO | 12.631 | 233.1780 | [M+H]^+^ | 234.1552 | 206.0826 | | 193.1046 | 22 |
| 288 | Dioxabenzofos | C_8_H_9_O_3_PS | 10.626 | 216.0010 | [M+H]^+^ | 216.9791 | 203.0854 | | 172.8557 | 22 |
| 289 | Methabenzthiazuron | C_10_H_11_N_3_OS | 7.184 | 221.0623 | [M+H]^+^ | 222.0403 | 166.1246 | | 200.6534 | 22 |
| 290 | Simeton | C_8_H_15_N_5_O | 4.506 | 197.1277 | [M+H]^+^ | 198.1069 | 128.0797 | | 185.9887 | 20 |
| 291 | Atratone | C_9_H_17_N_5_O | 5.313 | 211.1433 | [M+H]^+^ | 212.1220 | 170.1023 | | 194.9006 | 20 |
| 292 | Atrazine-desisopropyl | C_5_H_8_ClN_5_ | 3.857 | 173.0468 | [M+H]^+^ | 174.0273 | 146.9491 | | 133.9269 | 18 |
| 293 | Terbufos sulfone | C_9_H_21_O_4_PS_3_ | 12.240 | 320.0340 | [M+H]^+^ | 321.0069 | 279.8604 | | 230.9350 | 28 |
| 294 | Tefluthrin | C_17_H_14_ClF_7_O_2_ | 18.867 | 418.0571 | [M+H]^+^ | 419.0599 | 346.0102 | | 364.7619 | 32 |
| 295 | Bromocylen | C_8_H_5_BrCl_6_ | 18.197 | 389.7706 | [M+H]^+^ | 391.0833 | 381.5744 | | 351.8558 | 30 |
| 296 | Trietazine | C_9_H_16_ClN_5_ | 9.911 | 229.1094 | [M+NH_4_]^+^ | 246.9911 | 192.9820 | | 202.0372 | 22 |
| 297 | Cycluron | C_11_H_22_N_2_O | 7.333 | 198.1732 | [M+H]^+^ | 199.1529 | 157.8786 | | 171.1004 | 18 |
| 298 | 2,6-Dichlorobenzamide | C_7_H_5_Cl_2_NO | 4.032 | 188.9748 | [M+H]^+^ | 189.9545 | 149.9032 | | 127.0106 | 18 |
| 299 | 2,4,4'-Trichlorobiphenyl | C_12_H_7_Cl_3_ | 10.518 | 257.9770 | [M+NH_4_]^+^ | 276.0500 | 268.8156 | | 214.1190 | 22 |
| 300 | 2,4,5-Trichlorobiphenyl | C_12_H_7_Cl_3_ | 18.901 | 255.9613 | [M+H]^+^ | 256.2314 | 216.8893 | | 202.0855 | 22 |
| 301 | Sebuthylazine-desethyl | C_7_H_12_ClN_5_ | 6.078 | 201.0781 | [M+H]^+^ | 202.0572 | 195.0948 | | 174.0828 | 18 |
| 302 | 2,3,4,5-Tetrachloroaniline | C_6_H_3_Cl_4_N | 8.822 | 228.9020 | [M+H]^+^ | 230.0868 | 204.8083 | | 188.0471 | 20 |
| 303 | musk ambrette | C_12_H_16_N_2_O_5_ | 13.770 | 268.1059 | [M+H]^+^ | 269.1108 | 200.0834 | | 228.7856 | 22 |
| 304 | Musk xylene | C_12_H_15_N_3_O_6_ | 14.102 | 297.0961 | [M+H]^+^ | 297.9371 | 148.0378 | | 276.8140 | 22 |
| 305 | Pentachloroaniline | C_6_H_2_Cl_5_N | 18.194 | 262.8630 | [M+NH_4_]^+^ | 280.2303 | 221.8536 | | 175.8331 | 25 |
| 306 | Aziprotryne | C_7_H_11_N_7_S | 10.527 | 225.0797 | [M+H]^+^ | 226.0574 | 170.1020 | | 208.0969 | 20 |
| 307 | Sebuthylazine | C_9_H_16_ClN_5_ | 11.807 | 229.1094 | [M+H]^+^ | 230.0866 | 218.8129 | | 174.0523 | 20 |
| 308 | Isocarbamid | C_8_H_15_N_3_O_2_ | 5.506 | 185.1164 | [M+H]^+^ | 186.1206 | 145.9275 | | 156.8847 | 15 |
| 309 | 2,2',5,5'-Tetrachlorobiphenyl | C_12_H_6_Cl_4_ | 9.911 | 291.9380 | [M+H]^+^ | 292.9774 | 259.9592 | | 233.8264 | 25 |
| 310 | Musk moskene | C_14_H_18_N_2_O_4_ | 14.461 | 278.1267 | [M+Na]^+^ | 301.1671 | 256.2581 | | 240.2574 | 25 |
| 311 | Prosulfocarb | C_14_H_21_NOS | 16.655 | 251.1344 | [M+H]^+^ | 252.1102 | 223.9737 | | 195.9094 | 22 |
| 312 | Dimethenamid | C_12_H_18_ClNO_2_S | 10.527 | 275.0747 | [M+H]^+^ | 276.0499 | 206.8033 | | 156.8855 | 22 |
| 313 | Fenchlorphos-oxon | C_8_H_8_Cl_3_O_4_P | 11.333 | 303.9226 | [M+H]^+^ | 304.8956 | 264.8336 | | 236.8980 | 25 |
| 314 | Paraoxon-methyl | C_8_H_10_NO_6_P | 6.635 | 247.0246 | [M+H]^+^ | 248.0008 | 231.0263 | | 202.0371 | 22 |
| 315 | Monalide | C_13_H_18_ClNO | 14.036 | 239.1077 | [M+H]^+^ | 240.0844 | 223.1793 | | 200.0961 | 22 |
| 316 | Tibetene musk | C_13_H_18_N_2_O_4_ | 19.924 | 266.1267 | [M+NH_4_]^+^ | 284.2612 | 266.0413 | | 242.2398 | 24 |
| 317 | Isobenzan | C_9_H_4_Cl_8_O | 19.391 | 407.7770 | [M+H]^+^ | 408.2716 | 391.1845 | | 363.1661 | 28 |
| 318 | Octachlorostyrene | C_8_Cl_8_ | 18.263 | 375.7508 | [M+H]^+^ | 376.3244 | 260.7860 | | 336.1099 | 25 |
| 319 | Pyrimitate | C_11_H_20_N_3_O_3_PS | 13.204 | 305.0963 | [M+H]^+^ | 306.0661 | 262.8076 | | 247.8175 | 22 |
| 320 | Isodrin | C_12_H_8_Cl_6_ | 11.876 | 361.8757 | [M+H]^+^ | 363.3249 | 361.2276 | | 303.7794 | 25 |
| 321 | Isomethiozin | C_12_H_20_N_4_OS | 13.495 | 268.1358 | [M+H]^+^ | 269.1111 | 254.2433 | | 240.1255 | 20 |
| 322 | Trichloronate | C_10_H_12_Cl_3_O_2_PS | 9.232 | 331.9361 | [M+H]^+^ | 333.0044 | 296.7577 | | 265.8514 | 25 |
| 323 | Chlorthal-dimethyl | C_10_H_6_Cl_4_O_4_ | 17.898 | 329.9020 | [M+Na]^+^ | 353.2302 | 276.7876 | | 211.9183 | 25 |
| 324 | 4,4'-Dichlorobenzophenone | C_13_H_8_Cl_2_O | 16.414 | 249.9952 | [M+H]^+^ | 250.9712 | 221.0762 | | 196.9581 | 20 |
| 325 | Nitrothal-isopropyl | C_14_H_17_NO_6_ | 10.718 | 295.1056 | [M+Na]^+^ | 318.2656 | 287.9657 | | 261.7700 | 22 |
| 326 | Musk ketone | C_14_H_18_N_2_O_5_ | 7.192 | 294.1216 | [M+H]^+^ | 294.9892 | 280.4569 | | 250.8050 | 25 |
| 327 | Rabenzazol | C_12_H_12_N_4_ | 7.458 | 212.1062 | [M+H]^+^ | 213.0848 | 170.1000 | | 199.0433 | 20 |
| 328 | Cyprodinil | C_14_H_15_N_3_ | 8.855 | 225.1266 | [M+H]^+^ | 226.1048 | 210.1017 | | 185.1059 | 20 |
| 329 | Fuberidazole | C_11_H_8_N_2_O | 4.326 | 184.0637 | [M+H]^+^ | 185.0435 | 145.9283 | | 166.9430 | 18 |
| 330 | Isofenphos-oxon | C_15_H_24_NO_5_P | 10.552 | 329.1392 | [M+H]^+^ | 330.1120 | 200.9931 | | 303.0200 | 30 |
| 331 | Dicapthon | C_8_H_9_ClNO_5_PS | 18.138 | 296.9628 | [M]^+^ | 297.2089 | 280.1971 | | 258.8089 | 28 |
| 332 | 2,2',4,5,5'-Pentachlorobiphenyl | C_12_H_5_Cl_5_ | 15.038 | 325.8990 | [M+H]^+^ | 326.8774 | 284.2840 | | 258.7883 | 30 |
| 333 | MCPA-butoxyethyl ester | C_15_H_21_ClO_4_ | 13.878 | 300.1128 | [M+H]^+^ | 301.1359 | 172.8579 | | 271.7404 | 30 |
| 334 | Isocarbophos | C_11_H_16_NO_4_PS | 14.820 | 289.0538 | [M]^+^ | 289.1583 | 273.7907 | | 254.8681 | 28 |
| 335 | Phorate sulfone | C_7_H_17_O_4_PS_3_ | 16.697 | 292.0027 | [M+H]^+^ | 293.1384 | 252.8285 | | 277.2210 | 25 |
| 336 | Chlorfenethol | C_14_H_12_Cl_2_O | 20.359 | 266.0265 | [M+H]^+^ | 267.2720 | 242.2416 | | 200.0838 | 22 |
| 337 | trans-Nonachlor | C_10_H_5_Cl_9_ | 14.571 | 439.7588 | [M+H]^+^ | 440.8888 | 398.1698 | | 320.1856 | 30 |
| 338 | Dinobuton | C_14_H_18_N_2_O_7_ | 16.592 | 326.1114 | [M+H]^+^ | 327.0794 | 257.8025 | | 276.8294 | 25 |
| 339 | Tribufos | C_12_H_27_OPS_3_ | 18.560 | 314.0962 | [M+H]^+^ | 315.0690 | 300.4586 | | 256.2578 | 25 |
| 340 | Flurochloridone | C_12_H_10_Cl_2_F_3_NO | 16.431 | 311.0092 | [M+NH_4_]^+^ | 330.3018 | 318.7626 | | 268.0467 | 25 |
| 341 | Bromfenvinphos | C_12_H_14_BrCl_2_O_4_P | 14.460 | 401.9190 | [M+H]^+^ | 402.8884 | 350.7378 | | 321.1243 | 30 |
| 342 | Perthan | C_18_H_20_Cl_2_ | 9.845 | 306.0942 | [M+H]^+^ | 306.9923 | 278.0683 | | 232.8232 | 25 |
| 343 | Ditalimfos | C_12_H_14_NO_4_PS | 10.637 | 299.0381 | [M+H]^+^ | 300.0657 | 263.8075 | | 180.8655 | 25 |
| 344 | 2,3,4,4',5-Pentachlorobiphenyl | C_12_H_5_Cl_5_ | 12.098 | 325.8990 | [M+H]^+^ | 326.9077 | 310.2163 | | 270.1828 | 25 |
| 345 | 4,4'-Dibromobenzophenone | C_13_H_8_Br_2_O | 20.764 | 337.8942 | [M+H]^+^ | 338.3063 | 256.2558 | | 285.0968 | 25 |
| 346 | Flutriafol | C_16_H_13_F_2_N_3_O | 7.624 | 301.1027 | [M+H]^+^ | 302.0771 | 272.9486 | | 245.8133 | 25 |
| 347 | Mephosfolan | C_8_H_16_NO_3_PS_2_ | 6.485 | 269.0309 | [M+H]^+^ | 270.0066 | 254.2506 | | 240.1233 | 22 |
| 348 | Athidathion | C_8_H_15_N_2_O_4_PS_3_ | 13.712 | 329.9932 | [M+H]^+^ | 330.9660 | 315.2164 | | 280.8947 | 25 |
| 349 | 2,2',4,4',5,5'-Hexachlorobiphenyl | C_12_H_4_Cl_6_ | 17.263 | 361.8757 | [M+H]^+^ | 363.1065 | 362.2338 | | 323.2195 | 27 |
| 350 | Diclobutrazol | C_15_H_19_Cl_2_N_3_O | 12.157 | 327.0905 | [M+H]^+^ | 328.0629 | 267.1559 | | 251.8768 | 25 |
| 351 | Disulfoton-sulfone | C_8_H_19_O_4_PS_3_ | 10.804 | 306.0183 | [M+H]^+^ | 307.0209 | 284.7597 | | 124.9807 | 25 |
| 352 | Hexythiazox | C_17_H_21_ClN_2_O_2_S | 17.637 | 352.1012 | [M+H]^+^ | 353.0724 | 338.3290 | | 323.1793 | 25 |
| 353 | 2,2',3,4,4',5'-Hexachlorobiphenyl | C_12_H_4_Cl_6_ | 18.760 | 361.8757 | [M+H]^+^ | 363.1729 | 323.2266 | | 259.0126 | 28 |
| 354 | Triamiphos | C_12_H_19_N_6_OP | 7.234 | 294.1358 | [M+H]^+^ | 294.9894 | 240.2265 | | 216.9230 | 25 |
| 355 | Resmethrin | C_22_H_26_O_3_ | 18.693 | 338.1882 | [M+H]^+^ | 339.1599 | 325.1366 | | 247.3770 | 25 |
| 356 | Cyproconazole | C_15_H_18_ClN_3_O | 10.211 | 291.1138 | [M+H]^+^ | 292.0880 | 282.7631 | | 237.0290 | 22 |
| 357 | Benzyl butyl phthalate | C_19_H_20_O_4_ | 17.065 | 312.1362 | [M+H]^+^ | 313.1402 | 296.2892 | | 239.7960 | 25 |
| 358 | Clodinafop-propargyl | C_17_H_13_ClFNO_4_ | 15.367 | 349.0517 | [M+H]^+^ | 350.0233 | 318.7197 | | 282.1371 | 25 |
| 359 | Fenthion sulfoxide | C_10_H_15_O_4_PS_2_ | 6.818 | 294.0149 | [M+H]^+^ | 295.1102 | 273.8317 | | 193.8707 | 25 |
| 360 | Fluotrimazole | C_22_H_16_F_3_N_3_ | 12.292 | 379.1296 | [M+H]^+^ | 379.9625 | 360.7103 | | 320.7422 | 25 |
| 361 | Fluroxypyr-1-methylheptyl ester | C_15_H_21_Cl_2_FN_2_O_3_ | 18.111 | 366.0913 | [M+H]^+^ | 367.0616 | 349.1952 | | 225.9814 | 27 |
| 362 | Fenthion-sulfone | C_10_H_15_O_5_PS_2_ | 15.824 | 310.0099 | [M+H]^+^ | 311.0700 | 294.7426 | | 224.8363 | 25 |
| 363 | Triphenyl phosphate | C_18_H_15_O_4_P | 15.217 | 326.0708 | [M+H]^+^ | 327.0437 | 312.3223 | | 269.7637 | 25 |
| 364 | Metamitron | C_10_H_10_N_4_O | 4.872 | 202.0855 | [M+H]^+^ | 203.0644 | 185.8883 | | 160.9906 | 18 |
| 365 | 2,2',3,4,4',5,5'-Heptachlorobiphenyl | C_12_H_3_Cl_7_ | 7.786 | 395.8367 | [M+H]^+^ | 397.2228 | 353.7915 | | 287.9672 | 28 |
| 366 | Tebufenpyrad | C_18_H_24_ClN_3_O | 16.581 | 333.1608 | [M+H]^+^ | 334.1332 | 279.8016 | | 251.0719 | 25 |
| 367 | Cloquintocet-mexyl | C_18_H_22_ClNO_3_ | 7.059 | 335.1288 | [M+H]^+^ | 336.0682 | 318.9963 | | 295.1888 | 25 |
| 368 | Lenacil | C_13_H_18_N_2_O_2_ | 6.859 | 234.1368 | [M+H]^+^ | 235.1139 | 216.8355 | | 180.8708 | 20 |
| 369 | Bromuconazole | C_13_H_12_BrCl_2_N_3_O | 12.040 | 374.9541 | [M+H]^+^ | 375.9798 | 308.8062 | | 295.1909 | 25 |
| 370 | Nitralin | C_13_H_19_N_3_O_6_S | 14.843 | 345.0995 | [M+H]^+^ | 346.0708 | 323.2378 | | 279.7291 | 25 |
| 371 | Fenamiphos sulfoxide | C_13_H_22_NO_4_PS | 6.294 | 319.1007 | [M+H]^+^ | 320.0742 | 305.2706 | | 250.8742 | 25 |
| 372 | Fenamiphos sulfone | C_13_H_22_NO_5_PS | 16.473 | 335.0956 | [M+H]^+^ | 336.1010 | 320.7192 | | 241.7632 | 25 |
| 373 | Fenpiclonil | C_11_H_6_Cl_2_N_2_ | 10.444 | 235.9908 | [M+H]^+^ | 236.9669 | 229.3533 | | 185.9166 | 20 |
| 374 | Fluquinconazole | C_16_H_8_Cl_2_FN_5_O | 10.926 | 375.0090 | [M+H]^+^ | 375.9251 | 285.8073 | | 212.7901 | 28 |
| 375 | Fenbuconazole | C_19_H_17_ClN_4_ | 13.188 | 336.1142 | [M+H]^+^ | 337.0863 | 313.0267 | | 234.8041 | 25 |
| 376 | Ethylene brassylate | C_15_H_26_O_4_ | 16.697 | 270.1831 | [M+H]^+^ | 271.1579 | 254.2513 | | 231.1225 | 25 |
| 377 | Propoxur | C_11_H_15_NO_3_ | 17.609 | 209.1052 | [M+NH_4_]^+^ | 227.1064 | 186.1433 | | 172.1512 | 20 |
| 378 | Isoprocarb | C_11_H_15_NO_2_ | 8.245 | 193.1103 | [M+H]^+^ | 194.0887 | 176.9745 | | 161.1727 | 15 |
| 379 | Methamidophos | C_2_H_8_NO_2_PS | 0.728 | 141.0013 | [M+H]^+^ | 142.0045 | 125.0333 | | 110.0301 | 12 |
| 380 | Acenaphthene | C_12_H_10_ | 10.141 | 154.0783 | [M+NH_4_]^+^ | 172.1417 | 144.9502 | | 129.0383 | 15 |
| 381 | Dibutyl succinate | C_12_H_22_O_4_ | 14.540 | 230.1518 | [M+Na]^+^ | 253.1086 | 242.0611 | | 226.2119 | 22 |
| 382 | Phthalimide | C_8_H_5_NO_2_ | 5.129 | 147.0320 | [M+H]^+^ | 148.0351 | 134.6275 | | 147.1491 | 12 |
| 383 | Chlorethoxyfos | C_6_H_11_Cl_4_O_3_PS | 17.285 | 333.8921 | [M+NH_4_]^+^ | 351.1772 | 300.0594 | | 208.0425 | 28 |
| 384 | Pencycuron | C_19_H_21_ClN_2_O | 16.355 | 328.1342 | [M+H]^+^ | 329.1384 | 309.1779 | | 290.1092 | 28 |
| 385 | Tebuthiuron | C_9_H_16_N_4_OS | 6.158 | 228.1045 | [M+H]^+^ | 229.0810 | 188.1685 | | 172.1530 | 20 |
| 386 | Demeton-S-methyl | C_6_H_15_O_3_PS_2_ | 13.473 | 230.0200 | [M+H]^+^ | 231.0119 | 185.0238 | | 172.1520 | 20 |
| 387 | Cadusafos | C_10_H_23_O_2_PS_2_ | 6.607 | 270.0877 | [M+H]^+^ | 270.9818 | 216.0783 | | 202.0499 | 22 |
| 388 | Phenanthrene | C_14_H_10_ | 8.170 | 178.0783 | [M+NH_4_]^+^ | 196.0467 | 182.1631 | | 149.9621 | 18 |
| 389 | Spiroxamine | C_18_H_35_NO_2_ | 8.803 | 297.2668 | [M+H]^+^ | 298.2400 | 254.1644 | | 144.1928 | 25 |
| 390 | Fenpyroximate | C_24_H_27_N_3_O_4_ | 17.792 | 421.2002 | [M+H]^+^ | 422.1682 | 366.2364 | | 231.1717 | 32 |
| 391 | Tebupirimfos | C_13_H_23_N_2_O_3_PS | 17.484 | 318.1167 | [M+H]^+^ | 319.0886 | 303.0647 | | 273.2194 | 28 |
| 392 | Prohydrojasmon | C_15_H_26_O_3_ | 15.788 | 254.1882 | [M+H]^+^ | 255.1631 | 249.0235 | | 186.1441 | 22 |
| 393 | Fenpropidin | C_19_H_31_N | 8.628 | 273.2457 | [M+H]^+^ | 274.2196 | 265.2067 | | 228.2006 | 22 |
| 394 | Dicloran | C_6_H_4_Cl_2_N_2_O_2_ | 6.441 | 205.9650 | [M+H]^+^ | 206.9287 | 189.9968 | | 181.0641 | 18 |
| 395 | Pyroquilon | C_11_H_11_NO | 6.216 | 173.0841 | [M+H]^+^ | 174.0642 | 162.9946 | | 144.0991 | 15 |
| 396 | Propyzamide | C_12_H_11_Cl_2_NO | 11.813 | 255.0218 | [M+H]^+^ | 255.9963 | 186.1446 | | 163.1353 | 22 |
| 397 | Pirimicarb | C_11_H_18_N_4_O_2_ | 4.661 | 238.1430 | [M+H]^+^ | 239.1180 | 195.2250 | | 182.1923 | 20 |
| 398 | Phosphamidon | C_10_H_19_ClNO_5_P | 6.333 | 299.0689 | [M+H]^+^ | 300.0418 | 238.1636 | | 225.1535 | 25 |
| 399 | Benoxacor | C_11_H_11_Cl_2_NO_2_ | 11.677 | 259.0167 | [M+H]^+^ | 260.0215 | 256.0183 | | 219.9029 | 22 |
| 400 | Bromobutide | C_15_H_22_BrNO | 14.394 | 311.0885 | [M+H]^+^ | 312.0931 | 283.0171 | | 236.1943 | 25 |
| 401 | Acetochlor | C_14_H_20_ClNO_2_ | 13.044 | 269.1183 | [M+H]^+^ | 270.0924 | 263.8578 | | 254.3276 | 22 |
| 402 | Tridiphane | C_10_H_7_Cl_5_O | 15.314 | 317.8940 | [M+NH_4_]^+^ | 335.9960 | 299.8333 | | 295.2749 | 25 |
| 403 | Terbucarb | C_17_H_27_NO_2_ | 7.347 | 277.2042 | [M+NH_4_]^+^ | 295.0054 | 255.0841 | | 201.0536 | 23 |
| 404 | Esprocarb | C_15_H_23_NOS | 17.168 | 265.1500 | [M+H]^+^ | 266.1245 | 235.0701 | | 180.9806 | 22 |
| 405 | Fenfuram | C_12_H_11_NO_2_ | 7.879 | 201.0790 | [M+H]^+^ | 202.0573 | 201.1906 | | 187.9835 | 18 |
| 406 | Acibenzolar-S-methyl | C_8_H_6_N_2_OS_2_ | 7.256 | 209.9922 | [M+NH_4_]^+^ | 227.0944 | 186.1433 | | 172.1512 | 20 |
| 407 | Benfuresate | C_12_H_16_O_4_S | 11.753 | 256.0769 | [M+Na]^+^ | 279.0626 | 263.3133 | | 163.2066 | 22 |
| 408 | Dithiopyr | C_15_H_16_F_5_NO_2_S_2_ | 17.135 | 401.0543 | [M+H]^+^ | 402.0225 | 365.8828 | | 331.5202 | 28 |
| 409 | Metalaxyl-m | C_15_H_21_NO_4_ | 7.846 | 279.1471 | [M+H]^+^ | 280.1205 | 279.3783 | | 220.2007 | 22 |
| 410 | Malaoxon | C_10_H_19_O_7_PS | 7.081 | 314.0589 | [M+H]^+^ | 315.0314 | 254.9239 | | 195.0517 | 22 |
| 411 | Simeconazole | C_14_H_20_FN_3_OSi | 11.222 | 293.1360 | [M+H]^+^ | 294.1099 | 268.1078 | | 246.9019 | 22 |
| 412 | Chlorthal-dimethyl | C_10_H_6_Cl_4_O_4_ | 12.168 | 329.9020 | [M+H]^+^ | 330.9099 | 310.1849 | | 275.1457 | 22 |
| 413 | Thiazopyr | C_16_H_17_F_5_N_2_O_2_S | 15.547 | 396.0931 | [M+H]^+^ | 397.0625 | 377.1870 | | 335.1345 | 28 |
| 414 | Dimethylvinphos | C_10_H_10_Cl_3_O_4_P | 14.790 | 329.9382 | [M+H]^+^ | 330.3005 | 312.4081 | | 292.0872 | 22 |
| 415 | Butralin | C_14_H_21_N_3_O_4_ | 17.967 | 295.1532 | [M+H]^+^ | 296.1262 | 271.2527 | | 223.1036 | 22 |
| 416 | Zoxamide | C_14_H_16_Cl_3_NO_2_ | 15.330 | 335.0247 | [M+H]^+^ | 335.9958 | 293.2645 | | 245.8681 | 22 |
| 417 | Pyrifenox | C_14_H_12_Cl_2_N_2_O | 7.173 | 294.0327 | [M+H]^+^ | 295.0060 | 288.8205 | | 237.0627 | 22 |
| 418 | Allethrin | C_19_H_26_O_3_ | 17.512 | 302.1882 | [M+H]^+^ | 303.1072 | 302.2054 | | 264.1087 | 22 |
| 419 | Dimethametryn | C_11_H_21_N_5_S | 8.545 | 255.1518 | [M+H]^+^ | 256.1270 | 186.1448 | | 158.1086 | 20 |
| 420 | Quinoclamine | C_10_H_6_ClNO_2_ | 6.674 | 207.0087 | [M+H]^+^ | 207.9858 | 189.9964 | | 164.0129 | 18 |
| 421 | Methothrin | C_19_H_26_O_3_ | 19.680 | 302.1882 | [M+H]^+^ | 303.1610 | 291.1512 | | 249.1142 | 22 |
| 422 | Flufenacet | C_14_H_13_F_4_N_3_O_2_S | 13.385 | 363.0665 | [M+H]^+^ | 364.0365 | 331.1965 | | 323.3165 | 25 |
| 423 | Fenoxanil | C_15_H_18_Cl_2_N_2_O_2_ | 14.945 | 328.0745 | [M+H]^+^ | 329.0779 | 298.9646 | | 270.9661 | 22 |
| 424 | Fthalide | C_8_H_2_Cl_4_O_2_ | 14.856 | 269.8809 | [M+H]^+^ | 271.0621 | 254.3240 | | 225.1903 | 22 |
| 425 | Furalaxyl | C_17_H_19_NO_4_ | 10.424 | 301.1314 | [M+H]^+^ | 302.1044 | 287.8296 | | 221.0729 | 22 |
| 426 | Thiamethoxam | C_8_H_10_ClN_5_O_3_S | 4.503 | 291.0193 | [M+H]^+^ | 291.9927 | 248.1255 | | 236.0952 | 22 |
| 427 | Mepanipyrim | C_14_H_13_N_3_ | 11.946 | 223.1109 | [M+H]^+^ | 224.0879 | 188.1344 | | 160.1344 | 18 |
| 428 | Captan | C_9_H_8_Cl_3_NO_2_S | 16.237 | 298.9341 | [M+H]^+^ | 299.1047 | 272.9660 | | 257.9378 | 22 |
| 429 | Bromacil | C_9_H_13_BrN_2_O_2_ | 6.441 | 260.0160 | [M+H]^+^ | 261.0187 | 246.2674 | | 229.1696 | 20 |
| 430 | Picoxystrobin | C_18_H_16_F_3_NO_4_ | 14.931 | 367.1031 | [M+H]^+^ | 368.0730 | 355.2579 | | 274.3400 | 32 |
| 431 | Butamifos | C_13_H_21_N_2_O_4_PS | 16.636 | 332.0960 | [M+H]^+^ | 333.0671 | 295.2748 | | 247.1485 | 28 |
| 432 | Imazamethabenz-methyl | C_16_H_20_N_2_O_3_ | 5.950 | 288.1474 | [M+H]^+^ | 289.1211 | 257.2040 | | 229.2045 | 25 |
| 433 | (E)-Metominostrobin | C_16_H_16_N_2_O_3_ | 8.985 | 284.1161 | [M+H]^+^ | 285.0900 | 210.1544 | | 196.1414 | 25 |
| 434 | TCMTB | C_9_H_6_N_2_S_3_ | 4.753 | 237.9693 | [M+H]^+^ | 239.1189 | 195.2261 | | 182.1922 | 22 |
| 435 | Methiocarb sulfone | C_11_H_15_NO_4_S | 12.415 | 257.0722 | [M+H]^+^ | 258.0261 | 201.0565 | | 137.0598 | 25 |
| 436 | Imazalil | C_14_H_14_Cl_2_N_2_O | 7.148 | 296.0483 | [M+H]^+^ | 297.0185 | 255.0838 | | 159.0346 | 25 |
| 437 | Isoprothiolane | C_12_H_18_O_4_S_2_ | 12.827 | 290.0647 | [M+H]^+^ | 291.0380 | 272.8525 | | 221.1382 | 25 |
| 438 | Cyflufenamid | C_20_H_17_F_5_N_2_O_2_ | 16.861 | 412.1210 | [M+H]^+^ | 413.1244 | 368.7632 | | 289.0761 | 35 |
| 439 | Pyriminobac-methyl | C_17_H_19_N_3_O_6_ | 11.954 | 361.1274 | [M+H]^+^ | 362.0976 | 347.2596 | | 324.3115 | 30 |
| 440 | Isoxathion | C_13_H_16_NO_4_PS | 16.420 | 313.0538 | [M+H]^+^ | 314.0263 | 276.1162 | | 227.1528 | 30 |
| 441 | (Z)-Metominostrobin | C_16_H_16_N_2_O_3_ | 8.187 | 284.1161 | [M+H]^+^ | 285.0895 | 263.2228 | | 246.1264 | 25 |
| 442 | Diofenolan | C_18_H_20_O_4_ | 16.977 | 300.1362 | [M+H]^+^ | 301.1062 | 282.3643 | | 242.1935 | 28 |
| 443 | Thifluzamide | C_13_H_6_Br_2_F_6_N_2_O_2_S | 14.424 | 525.8421 | [M+H]^+^ | 526.8052 | 463.9932 | | 344.0866 | 38 |
| 444 | Quinoxyfen | C_15_H_8_Cl_2_FNO | 16.312 | 306.9967 | [M+H]^+^ | 307.9694 | 272.1056 | | 214.0509 | 25 |
| 445 | Chlorfenapyr | C_15_H_11_BrClF_3_N_2_O | 14.507 | 405.9695 | [M+Na]^+^ | 429.0305 | 385.1319 | | 345.0612 | 30 |
| 446 | Trifloxystrobin | C_20_H_19_F_3_N_2_O_4_ | 14.491 | 408.1297 | [M+Na]^+^ | 431.0278 | 416.1373 | | 296.8506 | 32 |
| 447 | Imibenconazole-oxon-desbenzyl | C_10_H_8_Cl_2_N_4_O | 17.659 | 270.0075 | [M+H]^+^ | 271.1362 | 254.3228 | | 197.1566 | 22 |
| 448 | Isoxadifen-ethyl | C_18_H_17_NO_3_ | 15.306 | 295.1208 | [M+H]^+^ | 296.1251 | 263.1804 | | 232.1466 | 25 |
| 449 | Fipronil | C_12_H_4_Cl_2_F_6_N_4_OS | 14.865 | 435.9387 | [M+H]^+^ | 436.9062 | 390.8292 | | 361.1287 | 32 |
| 450 | Imiprothrin | C_17_H_22_N_2_O_4_ | 17.592 | 318.1580 | [M+H]^+^ | 319.0884 | 303.2413 | | 217.1217 | 25 |
| 451 | Carfentrazone-ethyl | C_15_H_14_Cl_2_F_3_N_3_O_3_ | 14.491 | 411.0364 | [M+H]^+^ | 412.0040 | 400.7926 | | 376.3759 | 28 |
| 452 | Epoxiconazole | C_17_H_13_ClFN_3_O | 12.457 | 329.0731 | [M+H]^+^ | 330.0782 | 310.1850 | | 290.1787 | 22 |
| 453 | Pyraflufen-ethyl | C_15_H_13_Cl_2_F_3_N_2_O_4_ | 16.603 | 412.0204 | [M+H]^+^ | 413.0897 | 380.0619 | | 295.1628 | 30 |
| 454 | Pyributicarb | C_18_H_22_N_2_O_2_S | 17.701 | 330.1402 | [M+H]^+^ | 331.1116 | 310.1836 | | 284.1466 | 25 |
| 455 | Thenylchlor | C_16_H_18_ClNO_2_S | 13.401 | 323.0747 | [M+NH_4_]^+^ | 341.0700 | 326.8616 | | 270.9467 | 25 |
| 456 | Clethodim | C_17_H_26_ClNO_3_S | 7.446 | 359.1322 | [M+H]^+^ | 360.0989 | 343.8122 | | 300.2921 | 25 |
| 457 | Mefenpyr-diethyl | C_16_H_18_Cl_2_N_2_O_4_ | 15.846 | 372.0644 | [M+H]^+^ | 373.0342 | 342.8186 | | 300.9644 | 28 |
| 458 | Famphur | C_10_H_16_NO_5_PS_2_ | 11.482 | 325.0208 | [M+H]^+^ | 326.0250 | 289.9168 | | 269.1085 | 22 |
| 459 | Etoxazole | C_21_H_23_F_2_NO_2_ | 18.405 | 359.1697 | [M+H]^+^ | 360.1628 | 321.3038 | | 304.1952 | 25 |
| 460 | Pyriproxyfen | C_20_H_19_NO_3_ | 17.385 | 321.1365 | [M+H]^+^ | 322.1084 | 286.1084 | | 234.1276 | 22 |
| 461 | Picolinafen | C_19_H_12_F_4_N_2_O_2_ | 17.069 | 376.0835 | [M+H]^+^ | 377.0530 | 332.8931 | | 308.1292 | 25 |
| 462 | Iprodione | C_13_H_13_Cl_2_N_3_O_3_ | 4.952 | 329.0334 | [M+H]^+^ | 330.0744 | 285.1361 | | 249.9411 | 22 |
| 463 | Piperophos | C_14_H_28_NO_3_PS_2_ | 16.312 | 353.1248 | [M+H]^+^ | 354.0957 | 252.1023 | | 213.0488 | 25 |
| 464 | Ofurace | C_14_H_16_ClNO_3_ | 7.929 | 281.0819 | [M+H]^+^ | 282.0555 | 252.1911 | | 235.9564 | 22 |
| 465 | Bifenazate | C_17_H_20_N_2_O_3_ | 12.811 | 300.1474 | [M+H]^+^ | 301.1203 | 242.1877 | | 172.9248 | 25 |
| 466 | Endrin ketone | C_12_H_8_Cl_6_O | 15.846 | 377.8706 | [M+NH_4_]^+^ | 395.0151 | 378.1720 | | 352.1030 | 28 |
| 467 | Clomeprop | C_16_H_15_Cl_2_NO_2_ | 16.777 | 323.0480 | [M+H]^+^ | 324.0201 | 311.2089 | | 261.1094 | 22 |
| 468 | Fenamidone | C_17_H_17_N_3_OS | 11.572 | 311.1092 | [M+H]^+^ | 312.0820 | 283.8536 | | 273.0113 | 22 |
| 469 | Naproanilide | C_19_H_17_NO_2_ | 13.875 | 291.1259 | [M+H]^+^ | 292.0990 | 271.8417 | | 231.0839 | 22 |
| 470 | Pyraclostrobin | C_19_H_18_ClN_3_O_4_ | 15.663 | 387.0986 | [M+H]^+^ | 388.0682 | 177.1522 | | 145.1208 | 28 |
| 471 | Lactofen | C_19_H_15_ClF_3_NO_7_ | 17.617 | 461.0489 | [M+Na]^+^ | 483.9963 | 451.1327 | | 437.0961 | 35 |
| 472 | Tralkoxydim | C_20_H_27_NO_3_ | 10.420 | 329.1991 | [M+H]^+^ | 330.1673 | 284.1635 | | 267.1603 | 22 |
| 473 | Pyraclofos | C_14_H_18_ClN_2_O_3_PS | 14.973 | 360.0464 | [M+H]^+^ | 361.0501 | 335.8514 | | 323.3159 | 22 |
| 474 | Dialifos | C_14_H_17_ClNO_4_PS_2_ | 16.642 | 393.0025 | [M+H]^+^ | 394.0054 | 377.0924 | | 357.0859 | 25 |
| 475 | Spirodiclofen | C_21_H_24_Cl_2_O_4_ | 18.590 | 410.1052 | [M+H]^+^ | 411.0731 | 363.8753 | | 295.2627 | 28 |
| 476 | Halfenprox | C_24_H_23_BrF_2_O_3_ | 14.291 | 476.0799 | [M+NH_4_]^+^ | 494.0700 | 465.0966 | | 352.0914 | 35 |
| 477 | Flurtamone | C_18_H_14_F_3_NO_2_ | 10.657 | 333.0977 | [M+H]^+^ | 334.0692 | 313.1947 | | 295.2717 | 22 |
| 478 | Pyriftalid | C_15_H_14_N_2_O_4_S | 11.297 | 318.0674 | [M+H]^+^ | 319.0394 | 301.3651 | | 268.0263 | 22 |
| 479 | Silafluofen | C_25_H_29_FO_2_Si | 16.736 | 408.1921 | [M+H]^+^ | 409.0983 | 375.1767 | | 253.0671 | 32 |
| 480 | Pyrimidifen | C_20_H_28_ClN_3_O_2_ | 11.630 | 377.1870 | [M+H]^+^ | 378.1570 | 184.1263 | | 157.0997 | 30 |
| 481 | Acetamiprid | C_10_H_11_ClN_4_ | 5.566 | 222.0672 | [M+H]^+^ | 223.0442 | 210.1721 | | 187.1650 | 18 |
| 482 | Butafenacil | C_20_H_18_ClF_3_N_2_O_6_ | 14.299 | 474.0805 | [M+NH_4_]^+^ | 492.0720 | 435.9549 | | 311.3904 | 35 |
| 483 | Cafenstrole | C_16_H_22_N_4_O_3_S | 13.326 | 350.1413 | [M+H]^+^ | 351.1119 | 344.8047 | | 287.1517 | 25 |
| 484 | Fluridone | C_19_H_14_F_3_NO | 13.143 | 329.1027 | [M+H]^+^ | 330.0050 | 310.1833 | | 284.1527 | 22 |
| 485 | Heptachlor-2,3-exo-epoxide | C_10_H_5_Cl_7_O | 10.014 | 385.8160 | [M+H]^+^ | 387.1384 | 354.0385 | | 342.1160 | 25 |
| 486 | Methamidophos | C_2_H_8_NO_2_PS | 1.487 | 141.0013 | [M+H]^+^ | 142.0006 | 130.9027 | | 117.9331 | 12 |
| 487 | Carbofuran | C_12_H_15_NO_3_ | 7.387 | 221.1052 | [M+H]^+^ | 222.1193 | 165.0927 | | 137.0621 | 18 |
| 488 | Acetamiprid | C_10_H_11_ClN_4_ | 5.749 | 222.0672 | [M+H]^+^ | 223.0817 | 210.0853 | | 178.8964 | 18 |
| 489 | Trichlorfon | C_4_H_8_Cl_3_O_4_P | 4.926 | 255.9226 | [M+H]^+^ | 256.9379 | 230.1338 | | 204.8143 | 20 |
| 490 | Demeton | C_16_H_38_O_6_P_2_S_4_ | 6.325 | 516.1026 | [M+H]^+^ | 517.3898 | 461.2178 | | 328.7314 | 38 |
| 491 | Phorate sulfoxide | C_7_H_17_O_3_PS_3_ | 7.928 | 276.0077 | [M+Na]^+^ | 299.0067 | 176.0057 | | 146.9709 | 25 |
| 492 | Oxycarboxin | C_12_H_13_NO_4_S | 6.389 | 267.0565 | [M+H]^+^ | 268.0719 | 242.2418 | | 185.0082 | 22 |
| 493 | Phoxim | C_12_H_15_N_2_O_3_PS | 16.229 | 298.0541 | [M+H]^+^ | 299.1327 | 245.0981 | | 176.8868 | 25 |
| 494 | Methoxyfenozide | C_22_H_28_N_2_O_3_ | 5.281 | 368.2100 | [M+NH_4_]^+^ | 387.1935 | 307.2495 | | 259.8860 | 35 |
| 495 | Diafenthiuron | C_23_H_32_N_2_OS | 18.539 | 384.2235 | [M+H]^+^ | 385.2445 | 329.1739 | | 287.1256 | 35 |
| 496 | Thifensulfuron-methyl | C_12_H_13_N_5_O_6_S_2_ | 7.113 | 387.0307 | [M+H]^+^ | 388.0510 | 311.8448 | | 204.9662 | 35 |
| 497 | Ethoxysulfuron | C_15_H_18_N_4_O_7_S | 12.535 | 398.0896 | [M+H]^+^ | 399.1101 | 335.7260 | | 295.1923 | 35 |
| 498 | Spinosad | C_41_H_65_NO_10_ | 13.815 | 731.9680 | [M+H]^+^ | 732.4944 | 189.1144 | | 157.0869 | 38 |
| 499 | Mepiquat chloride | C_7_H_16_ClN | 6.925 | 149.0971 | [M+NH_4_]^+^ | 167.1136 | 135.0167 | | 106.0425 | 15 |
| 500 | Tricyclazole | C_9_H_7_N_3_S | 5.874 | 189.0361 | [M+H]^+^ | 190.0490 | 173.0135 | | 163.0333 | 15 |
| 501 | Isoproturon | C_12_H_18_N_2_O | 7.862 | 206.1419 | [M+H]^+^ | 207.1556 | 190.9358 | | 174.9102 | 18 |
| 502 | Pymetrozine | C_10_H_11_N_5_O | 10.982 | 217.0964 | [M+H]^+^ | 218.1103 | 176.8857 | | 128.9883 | 18 |
| 503 | Flumetsulam | C_12_H_9_F_2_N_5_O_2_S | 5.998 | 325.0445 | [M+H]^+^ | 326.0630 | 262.0883 | | 242.0819 | 22 |
| 504 | Thiodicarb | C_10_H_18_N_4_O_4_S_3_ | 7.379 | 354.0490 | [M+H]^+^ | 355.0679 | 295.2023 | | 264.8060 | 25 |
| 505 | Cinosulfuron | C_15_H_19_N_5_O_7_S | 7.321 | 413.1005 | [M+H]^+^ | 414.1218 | 183.0530 | | 157.0739 | 28 |
| 506 | Pyrazosulfuron-ethyl | C_14_H_18_N_6_O_7_S | 11.936 | 414.0958 | [M+H]^+^ | 415.1176 | 213.9936 | | 188.0147 | 28 |
| 507 | Methomyl | C_5_H_10_N_2_O_2_S | 5.541 | 162.0463 | [M+H]^+^ | 163.0874 | 173.0211 | | 145.9263 | 12 |
| 508 | Cymoxanil | C_7_H_10_N_4_O_3_ | 16.975 | 198.0753 | [M+H]^+^ | 199.0220 | 183.9941 | | 170.0000 | 15 |
| 509 | Omethoate | C_5_H_12_NO_4_PS | 2.549 | 213.0225 | [M+H]^+^ | 214.0929 | 201.9369 | | 157.8882 | 18 |
| 510 | Ethoxyquin | C_14_H_19_NO | 7.287 | 217.1467 | [M+H]^+^ | 218.1610 | 202.1249 | | 190.1238 | 18 |
| 511 | Aldoxycarb | C_7_H_14_N_2_O_4_S | 3.562 | 222.0674 | [M+H]^+^ | 223.1399 | 207.1207 | | 166.0735 | 18 |
| 512 | Imazapic | C_14_H_17_N_3_O_3_ | 5.341 | 275.1270 | [M+H]^+^ | 276.1432 | 231.1163 | | 216.0795 | 22 |
| 513 | Uniconazole | C_15_H_18_ClN_3_O | 12.443 | 291.1138 | [M+H]^+^ | 292.1307 | 218.0508 | | 170.1108 | 25 |
| 514 | Clofentezine | C_14_H_8_Cl_2_N_4_ | 16.377 | 302.0126 | [M+H]^+^ | 303.0305 | 172.8671 | | 138.0125 | 25 |
| 515 | Vamidothion sulfone | C_8_H_18_NO_6_PS_2_ | 4.260 | 319.3300 | [M+Na]^+^ | 342.0319 | 200.0380 | | 169.0115 | 22 |
| 516 | Terbufos sulfone | C_9_H_21_O_4_PS_3_ | 12.942 | 320.0340 | [M+Na]^+^ | 343.0352 | 240.9765 | | 193.0067 | 22 |
| 517 | Cyazofamid | C_13_H_13_ClN_4_O_2_S | 15.420 | 324.0448 | [M+H]^+^ | 325.0626 | 246.0600 | | 217.0391 | 22 |
| 518 | Florasulam | C_12_H_8_F_3_N_5_O_3_S | 7.437 | 359.0300 | [M+H]^+^ | 360.0495 | 321.2054 | | 279.2197 | 25 |
| 519 | Benzoximate | C_18_H_18_ClNO_5_ | 16.975 | 363.0874 | [M+Na]^+^ | 386.0896 | 329.1734 | | 287.1259 | 25 |
| 520 | Chlormequat chloride | C_5_H_13_Cl_2_N | 6.578 | 157.0425 | [M+NH_4_]^+^ | 175.0044 | 140.9491 | | 125.0226 | 15 |
| 521 | Sethoxydim | C_17_H_29_NO_3_S | 17.364 | 327.1868 | [M+H]^+^ | 328.1987 | 295.1986 | | 220.1327 | 25 |
| 522 | Folpet | C_9_H_4_Cl_3_NO_2_S | 13.116 | 294.9028 | [M+NH_4_]^+^ | 313.2304 | 437.2006 | | 296.2257 | 25 |
| 523 | Methiocarb | C_11_H_15_NO_2_S | 11.115 | 225.0824 | [M+H]^+^ | 226.0846 | 167.0175 | | 134.8941 | 18 |
| 524 | Cartap hydrochloride | C_7_H_16_ClN_3_O_2_S_2_ | 6.588 | 273.0372 | [M+NH_4_]^+^ | 290.0391 | 267.8004 | | 256.2705 | 25 |
| **Persistent organic pollutants** | | | | | | | | | | |
| 1 | Propyl phosphate | C_9_H_21_O_4_P | 9.025 | 224.1177 | [M+H]^+^ | 225.0926 | 200.0739 | | 182.0634 | 20 |
| 2 | Triisobutyl phosphate | C_12_H_27_O_4_P | 14.788 | 266.1647 | [M+H]^+^ | 267.1364 | 249.1776 | | 232.8010 | 22 |
| 3 | Tris(2-butoxyethyl) phosphate | C_18_H_39_O_7_P | 16.351 | 398.2433 | [M+H]^+^ | 399.2095 | 281.0498 | | 247.9503 | 32 |
| 4 | 2-Ethylhexyldiphenyl phosphate | C_20_H_27_O_4_P | 18.430 | 362.1647 | [M+H]^+^ | 363.1314 | 324.2264 | | 298.7713 | 32 |
| 5 | Tris(2-ethylhexyl) Phosphate | C_24_H_51_O_4_P | 21.840 | 434.3525 | [M+H]^+^ | 435.3163 | 332.0789 | | 267.0481 | 35 |
| 6 | Tris(3-methylphenyl) phosphate | C_21_H_21_O_4_P | 17.840 | 368.1177 | [M+H]^+^ | 369.0844 | 279.0772 | | 243.0561 | 32 |
| 7 | Tri-p-tolyl phosphate | C_21_H_21_O_4_P | 19.262 | 368.1177 | [M+H]^+^ | 369.2573 | 354.0974 | | 287.8950 | 32 |
| 8 | Tris(2-methylphenyl) phosphate | C_21_H_21_O_4_P | 17.870 | 368.1177 | [M+Na]^+^ | 391.0977 | 354.9801 | | 317.2930 | 35 |
| 9 | Tris(1,3-dichloroisopropyl)phosphate | C_9_H_15_Cl_6_O_4_P | 14.763 | 427.8839 | [M+H]^+^ | 428.8471 | 371.6677 | | 293.1843 | 35 |
| 10 | Cresyl diphenyl phosphate | C_19_H_17_O_4_P | 16.476 | 340.0864 | [M+H]^+^ | 341.0546 | 265.0597 | | 229.0408 | 30 |
| 11 | Phenyl phosphate | C_18_H_15_O_4_P | 15.688 | 326.0708 | [M+H]^+^ | 326.9700 | 268.0314 | | 197.8663 | 28 |
| 12 | Tris(1-Chloro-2-Propyl) Phosphate | C_9_H_18_Cl_3_O_4_P | 11.170 | 326.0008 | [M+H]^+^ | 326.9697 | 264.7961 | | 209.8759 | 28 |
| 13 | Tributyl phosphate | C_12_H_27_O_4_P | 15.528 | 266.1647 | [M+H]^+^ | 267.1368 | 208.8300 | | 180.8168 | 22 |
| 14 | tris(2-chloroethyl) phosphate | C_6_H_12_Cl_3_O_4_P | 7.403 | 283.9539 | [M+H]^+^ | 284.9245 | 270.1344 | | 251.8163 | 25 |
| 15 | Triethyl phosphate | C_6_H_15_O_4_P | 9.066 | 182.0708 | [M+H]^+^ | 183.0481 | 155.0464 | | 137.9617 | 15 |
| 16 | Trimethyl phosphate | C_3_H_9_O_4_P | 2.389 | 140.0238 | [M+H]^+^ | 141.0046 | 109.0046 | | 101.9496 | 10 |
| 17 | Benzyl butyl phthalate | C_19_H_20_O_4_ | 16.754 | 312.1362 | [M+H]^+^ | 313.1051 | 254.8627 | | 219.7692 | 28 |
| 18 | [Diphenyl phthalate](https://www.chemsrc.com/en/cas/84-62-8_510543.html) | C_20_H_14_O_4_ | 15.956 | 318.0892 | [M+H]^+^ | 319.0517 | 225.0561 | | 197.0624 | 28 |
| 19 | [β-Butoxyethyl phthalate](https://www.chemsrc.com/en/cas/117-83-9_750429.html) | C_20_H_30_O_6_ | 17.012 | 366.2042 | [M+H]^+^ | 367.2898 | 340.9045 | | 238.9201 | 32 |
| 20 | [Dibutyl phthalate](https://www.chemsrc.com/en/cas/84-74-2_336203.html) | C_16_H_22_O_4_ | 16.845 | 278.1518 | [M+H]^+^ | 279.1231 | 245.2277 | | 216.8593 | 25 |
| 21 | [Dicyclohexyl phthalate](https://www.chemsrc.com/en/cas/84-61-7_670264.html) | C_20_H_26_O_4_ | 18.442 | 330.1831 | [M+H]^+^ | 331.1516 | 240.2734 | | 227.8229 | 28 |
| 22 | [Dimethyl phthalate](https://www.chemsrc.com/en/cas/131-11-3_28935.html) | C_10_H_10_O_4_ | 15.113 | 194.0579 | [M+NH_4_]^+^ | 211.1701 | 187.8724 | | 172.9185 | 18 |
| 23 | dipentyl phthalate | C_18_H_26_O_4_ | 18.351 | 306.1831 | [M+H]^+^ | 307.1528 | 292.0466 | | 265.0726 | 25 |
| 24 | [Diethyl phthalate](https://www.chemsrc.com/en/cas/84-66-2_401979.html) | C_12_H_14_O_4_ | 5.703 | 222.0892 | [M+H]^+^ | 223.0645 | 207.0339 | | 192.0060 | 18 |
| 25 | Diisobutyl phthalate | C_16_H_22_O_4_ | 20.493 | 278.1518 | [M+H]^+^ | 279.1229 | 245.2260 | | 219.2068 | 25 |
| 26 | Dihexyl phthalate | C_20_H_30_O_4_ | 19.265 | 334.2144 | [M+H]^+^ | 335.1823 | 251.2376 | | 209.1849 | 25 |
| 27 | Dioctyl phthalate | C_24_H_38_O_4_ | 20.305 | 390.2770 | [M+H]^+^ | 391.3647 | 359.0457 | | 311.1675 | 35 |
| 28 | Bis(2-methoxyethyl) phthalate | C_14_H_18_O_6_ | 7.401 | 282.1103 | [M+H]^+^ | 283.2457 | 265.2532 | | 163.1518 | 25 |
| 29 | bis(2-ethoxyethyl) benzene-1,2-dicarboxylate | C_16_H_22_O_6_ | 10.076 | 310.1416 | [M+H]^+^ | 311.2755 | 268.3027 | | 226.2530 | 28 |
| 30 | 1,2-Benzenedicarboxylic acid, 1,2-bis(1,3-dimethylbutyl) ester | C_20_H_30_O_4_ | 19.041 | 334.2144 | [M+H]^+^ | 335.1827 | 321.3232 | | 303.3040 | 28 |
| 31 | bis(2-ethylhexyl) phthalate | C_24_H_38_O_4_ | 20.504 | 390.2770 | [M+H]^+^ | 391.2419 | 376.2719 | | 306.1448 | 35 |
| 32 | Dinonyl phthalate | C_26_H_42_O_4_ | 21.303 | 418.3083 | [M+H]^+^ | 419.2716 | 293.1459 | | 265.1397 | 35 |
| **Marine toxins** | | | | | | | | | | |
| 1 | N-Sulfocarbamoylgonyautoxin-2 | C_10_H_17_N_7_O_11_S_2_ | 0.727 | 475.0427 | [M+H]^+^ | 476.2539 | 339.9188 | | 323.2334 | 30 |
| 2 | N-Sulfocarbamoylgonyautoxin-3 | C_10_H_17_N_7_O_11_S_2_ | 4.488 | 475.0427 | [M+H]^+^ | 476.2539 | 343.3340 | | 324.2784 | 35 |
| 3 | Decarbamovlgonyautoxin-2 | C_9_H_16_N_6_O_7_S | 0.605 | 352.0801 | [M+H]^+^ | 353.0431 | 255.0682 | | 206.8596 | 28 |
| 4 | Decarbamovlgonyautoxin-3 | C_9_H_16_N_6_O_7_S | 0.740 | 352.0801 | [M+H]^+^ | 353.0435 | 273.0925 | | 213.0631 | 27 |
| 5 | Decarbamoylneosaxitoxin dihydrochloride | C_9_H_18_N_6_O_4_Cl_2_ | 0.558 | 272.1233 | [M+H]^+^ | 273.0913 | 207.1668 | | 225.1789 | 24 |
| 6 | Decarbamoylsaxitoxin | C_9_H_16_N_6_O_3_ | 0.559 | 256.1284 | [M+H]^+^ | 257.0965 | 239.0864 | | 222.0623 | 22 |
| 7 | Gonyautoxin-1 | C_10_H_17_N_7_O_9_S | 0.615 | 411.0808 | [M+H]^+^ | 412.1022 | 316.1434 | | 298.1339 | 36 |
| 8 | Gonyautoxin-4 | C_10_H_17_N_7_O_9_S | 0.707 | 411.0808 | [M+Na]^+^ | 434.7926 | 323.2337 | | 363.8264 | 34 |
| 9 | Gonyautoxin-2 | C_10_H_17_N_7_O_8_S | 0.608 | 395.0859 | [M+H]^+^ | 396.1066 | 298.0854 | | 196.0496 | 36 |
| 10 | Gonyautoxin-3 | C_10_H_17_N_7_O_8_S | 0.667 | 395.0859 | [M+H]^+^ | 396.1038 | 220.0743 | | 250.8965 | 35 |
| 11 | Gonyautoxin-6 | C_10_H_17_N_7_O_8_S | 0.569 | 395.0859 | [M+H]^+^ | 396.0443 | 238.0788 | | 177.0475 | 36 |
| 12 | Neosaxitoxin | C_10_H_17_N_7_O_5_ | 0.566 | 315.1291 | [M+H]^+^ | 316.0937 | 195.0533 | | 225.0719 | 28 |
| 13 | Saxitoxin dihydrochloride | C_10_H_19_N_7_O_4_Cl_2_ | 0.556 | 299.0876 | [M+H]^+^ | 300.0997 | 204.0878 | | 282.1320 | 26 |
| 14 | Tetrodotoxin | C_11_H_17_N_3_O_8_ | 0.603 | 319.1016 | [M+H]^+^ | 320.1088 | 302.1001 | | 284.0895 | 28 |
| 15 | Microcystin RR | C_49_H_75_N_13_O_12_ | 6.778 | 1037.5658 | [M+2H]^2+^ | 519.8095 | 477.2450 | | 455.2669 | 35 |
| 16 | Microcystin LR | C_49_H_74_N_10_O_12_ | 7.651 | 994.5488 | [M+H]^+^ | 995.5560 | 849.6284 | | 823.1368 | 45 |
| 17 | Okadaic Acid | C_44_H_68_O_13_ | 13.584 | 805.0000 | [M+H]^+^ | 806.0073 | 636.7943 | | 517.8241 | 35 |
| 18 | Nodularin | C_41_H_60_N_8_O_10_ | 6.822 | 824.4432 | [M+H]^+^ | 825.4505 | 797.4559 | | 781.4507 | 42 |
| **Veterinary drugs** | | | | | | | | | | |
| 1 | Sulfabenzamide | C_13_H_12_N_2_O_3_S | 6.222 | 276.0569 | [M+H]^+^ | 277.0297 | 186.0099 | | 165.8959 | 22 |
| 2 | Sulfadiazine | C_10_H_10_N_4_O_2_S | 3.503 | 250.0524 | [M+H]^+^ | 251.0338 | 184.0565 | | 169.0414 | 20 |
| 3 | Sulfadimethoxine | C_12_H_14_N_4_O_4_S | 5.482 | 310.0736 | [M+H]^+^ | 311.0442 | 245.0682 | | 217.9878 | 25 |
| 4 | Sulfamethazine | C_12_H_14_N_4_O_2_S | 4.301 | 278.0837 | [M+H]^+^ | 279.0557 | 213.0799 | | 186.0021 | 25 |
| 5 | Sulfadoxine | C_12_H_14_N_4_O_4_S | 6.363 | 310.0736 | [M+H]^+^ | 311.0443 | 245.0681 | | 217.9893 | 25 |
| 6 | Sulfamerazine | C_11_H_12_N_4_O_2_S | 3.511 | 264.0681 | [M+H]^+^ | 265.0412 | 199.0665 | | 171.9878 | 22 |
| 7 | Sulfamethizole | C_9_H_10_N_4_O_2_S_2_ | 4.675 | 270.0245 | [M+H]^+^ | 270.9973 | 198.0440 | | 177.9512 | 22 |
| 8 | Sulfamethoxypyridazine | C_11_H_12_N_4_O_3_S | 4.700 | 280.0630 | [M+H]^+^ | 281.0347 | 215.0592 | | 187.9813 | 25 |
| 9 | Sulfaphenazole | C_15_H_14_N_4_O_2_S | 6.496 | 314.0837 | [M+H]^+^ | 315.0547 | 221.9998 | | 158.0428 | 25 |
| 10 | Sulfapyrazole | C_16_H_16_N_4_O_2_S | 6.721 | 328.0994 | [M+H]^+^ | 329.0695 | 236.0138 | | 175.0747 | 25 |
| 11 | Sulfapyridine | C_11_H_11_N_3_O_2_S | 3.503 | 249.0572 | [M+H]^+^ | 250.0311 | 232.0585 | | 184.0868 | 22 |
| 12 | Sulfaquinoxaline | C_14_H_12_N_4_O_2_S | 6.396 | 300.0681 | [M+H]^+^ | 301.0398 | 285.1427 | | 208.0160 | 25 |
| 13 | Sulfathiazole | C_9_H_9_N_3_O_2_S_2_ | 3.498 | 255.0136 | [M+H]^+^ | 256.0211 | 237.2164 | | 209.1852 | 22 |
| 14 | Sulfisomidine | C_12_H_14_N_4_O_2_S | 2.804 | 278.0837 | [M+H]^+^ | 279.0559 | 186.0027 | | 155.9843 | 25 |
| 15 | Trimethoprim | C_14_H_18_N_4_O_3_ | 4.160 | 290.1379 | [M+H]^+^ | 291.1096 | 275.1132 | | 261.0975 | 25 |
| 16 | Cinoxacin | C_12_H_10_N_2_O_5_ | 5.548 | 262.0590 | [M+H]^+^ | 263.0319 | 245.0209 | | 217.0277 | 25 |
| 17 | Danofloxacin | C_19_H_20_FN_3_O_3_ | 4.825 | 357.1489 | [M+H]^+^ | 358.1560 | 340.1453 | | 316.1816 | 32 |
| 18 | Difloxacin | C_21_H_19_F_2_N_3_O_3_ | 5.376 | 399.1394 | [M+H]^+^ | 400.1494 | 382.1352 | | 356.1559 | 35 |
| 19 | Enrofloxacin | C_19_H_22_FN_3_O_3_ | 4.858 | 359.1645 | [M+H]^+^ | 360.1717 | 342.1594 | | 316.1818 | 32 |
| 20 | Flumequine | C_14_H_12_FNO_3_ | 5.972 | 261.0801 | [M+H]^+^ | 262.0370 | 245.0266 | | 244.0256 | 22 |
| 21 | Gatifloxacin | C_19_H_22_FN_3_O_4_ | 5.007 | 375.1594 | [M+H]^+^ | 376.1273 | 358.1161 | | 332.1366 | 32 |
| 22 | Lomefloxacin | C_17_H_19_F_2_N_3_O_3_ | 4.633 | 351.1394 | [M+H]^+^ | 352.1085 | 334.0959 | | 308.1171 | 32 |
| 23 | Marbofloxacin | C_17_H_19_FN_4_O_4_ | 4.192 | 362.1390 | [M+H]^+^ | 363.1069 | 345.0936 | | 320.0636 | 32 |
| 24 | Moxifloxacin | C_21_H_24_FN_3_O_4_ | 5.415 | 401.1751 | [M+H]^+^ | 402.1415 | 384.1282 | | 358.1505 | 35 |
| 25 | Nalidixic acid | C_12_H_12_N_2_O_3_ | 6.853 | 232.0848 | [M+H]^+^ | 233.0601 | 215.0490 | | 187.0195 | 18 |
| 26 | Ofloxacin | C_18_H_20_FN_3_O_4_ | 4.409 | 361.1438 | [M+H]^+^ | 362.1119 | 344.1092 | | 319.1249 | 32 |
| 27 | Orbifloxacin | C_19_H_20_F_3_N_3_O_3_ | 4.858 | 395.1457 | [M+H]^+^ | 396.1124 | 378.0991 | | 352.1224 | 35 |
| 28 | Oxolinic acid | C_13_H_11_NO_5_ | 7.095 | 261.0637 | [M+H]^+^ | 262.0534 | 246.0473 | | 245.0452 | 22 |
| 29 | Sarafloxacin | C_20_H_17_F_2_N_3_O_3_ | 5.276 | 385.1238 | [M+H]^+^ | 386.1290 | 368.1195 | | 342.1406 | 35 |
| 30 | Sparfloxacin | C_19_H_22_F_2_N_4_O_3_ | 5.292 | 392.1660 | [M+H]^+^ | 393.1735 | 375.1609 | | 349.1827 | 35 |
| 31 | Tosufloxacin | C_19_H_15_F_3_N_4_O_3_ | 5.710 | 404.1096 | [M+H]^+^ | 405.1217 | 387.1053 | | 367.0979 | 35 |
| 32 | Albendazole | C_12_H_15_N_3_O_2_S | 6.734 | 265.0885 | [M+H]^+^ | 266.0613 | 235.0374 | | 234.0351 | 22 |
| 33 | Albendazole-2-aminosulfone | C_10_H_13_N_3_O_2_S | 3.633 | 239.0728 | [M+H]^+^ | 240.0470 | 207.9842 | | 198.0010 | 22 |
| 34 | Albendazole sulfoxide | C_12_H_15_N_3_O_3_S | 4.672 | 281.0834 | [M+H]^+^ | 282.0551 | 265.0516 | | 240.0086 | 22 |
| 35 | Mebendazole-amine | C_14_H_11_N_3_O | 5.038 | 237.0902 | [M+H]^+^ | 238.0644 | 206.0062 | | 105.0103 | 22 |
| 36 | Thiabendazole | C_10_H_7_N_3_S | 3.466 | 201.0361 | [M+H]^+^ | 202.0124 | 175.0021 | | 131.0338 | 18 |
| 37 | Dimetridazole | C_5_H_7_N_3_O_2_ | 2.577 | 141.0538 | [M+H]^+^ | 142.0353 | 112.0384 | | 101.9277 | 12 |
| 38 | Fenbendazole | C_15_H_13_N_3_O_2_S | 7.915 | 299.0728 | [M+H]^+^ | 300.0438 | 268.0173 | | 189.9762 | 25 |
| 39 | Flubendazole | C_16_H_12_FN_3_O_3_ | 6.951 | 313.0863 | [M+H]^+^ | 314.0565 | 283.0337 | | 282.0288 | 25 |
| 40 | Hydroxy ipronidazole | C_7_H_11_N_3_O_3_ | 4.473 | 185.0800 | [M+H]^+^ | 186.0575 | 168.0472 | | 138.0499 | 15 |
| 41 | Ipronidazole | C_7_H_11_N_3_O_2_ | 5.446 | 169.0851 | [M+H]^+^ | 170.0639 | 140.0664 | | 124.0742 | 15 |
| 42 | Mebendazole | C_16_H_13_N_3_O_3_ | 6.660 | 295.0957 | [M+H]^+^ | 296.0671 | 264.0391 | | 209.8582 | 22 |
| 43 | Cambendazole | C_14_H_14_N_4_O_2_S | 5.221 | 302.0837 | [M+H]^+^ | 303.0537 | 261.0076 | | 242.9986 | 25 |
| 44 | 5-Hydroxythiabendazole | C_10_H_7_N_3_OS | 3.044 | 217.0310 | [M+H]^+^ | 218.0065 | 199.9353 | | 191.0001 | 18 |
| 45 | Levamisole | C_11_H_12_N_2_S | 3.498 | 204.0721 | [M+H]^+^ | 205.0793 | 188.0533 | | 178.0690 | 18 |
| 46 | Metronidazole | C_6_H_9_N_3_O_3_ | 2.373 | 171.0644 | [M+H]^+^ | 172.0724 | 153.8891 | | 128.0462 | 15 |
| 47 | Oxfendazole | C_15_H_13_N_3_O_3_S | 5.647 | 315.0678 | [M+H]^+^ | 316.0381 | 284.0106 | | 267.0085 | 25 |
| 48 | Oxibendazole | C_12_H_15_N_3_O_3_ | 5.789 | 249.1113 | [M+H]^+^ | 250.0849 | 218.0586 | | 176.0154 | 22 |
| 49 | Ronidazole | C_6_H_8_N_4_O_4_ | 7.245 | 200.0546 | [M+H]^+^ | 201.0311 | 186.8919 | | 173.0380 | 18 |
| 50 | Secnidazole | C_7_H_11_N_3_O_3_ | 3.427 | 185.0800 | [M+H]^+^ | 186.0576 | 168.0465 | | 128.0201 | 15 |
| 51 | Tinidazole | C_8_H_13_N_3_O_4_S | 3.984 | 247.0627 | [M+H]^+^ | 248.0361 | 202.0446 | | 154.0344 | 22 |
| 52 | Triclabendazole | C_14_H_9_Cl_3_N_2_OS | 14.266 | 357.9501 | [M+H]^+^ | 358.9576 | 345.9310 | | 308.9649 | 28 |
| 53 | Clindamycin | C_18_H_33_ClN_2_O_5_S | 5.567 | 424.1799 | [M+H]^+^ | 425.1937 | 389.2060 | | 174.1153 | 32 |
| 54 | Doramectin | C_50_H_74_O_14_ | 6.699 | 898.5079 | [M+NH_4_]^+^ | 916.4982 | 859.4921 | | 777.4137 | 42 |
| 55 | Eprinomectin | C_50_H_75_NO_14_ | 17.550 | 913.5187 | [M+Na]^+^ | 936.5223 | 832.8622 | | 754.5963 | 42 |
| 56 | Ivermectin | C_48_H_74_O_14_ | 17.731 | 874.5079 | [M+H]^+^ | 875.4950 | 844.5102 | | 700.0560 | 38 |
| 57 | Leucomycin A1 | C_40_H_67_NO_14_ | 7.554 | 785.4562 | [M+H]^+^ | 786.4743 | 558.3246 | | 540.3119 | 38 |
| 58 | Spiramycin | C_43_H_74_N_2_O_14_ | 5.159 | 842.5140 | [M+H]^+^ | 843.5337 | 700.4338 | | 642.5857 | 38 |
| 59 | Tilmicosin | C_46_H_80_N_2_O_13_ | 5.858 | 868.5660 | [M+H]^+^ | 869.5855 | 803.5459 | | 696.4679 | 38 |
| 60 | Tylosin | C_46_H_77_NO_17_ | 6.548 | 915.5192 | [M+H]^+^ | 916.5403 | 772.4440 | | 598.3570 | 42 |
| 61 | Virginiamycin M1 | C_28_H_35_N_3_O_7_ | 7.779 | 525.2475 | [M+H]^+^ | 526.2523 | 530.2232 | | 506.2189 | 35 |
| 62 | Beclomethasone | C_22_H_29_ClO_5_ | 7.201 | 408.1704 | [M+H]^+^ | 409.1361 | 373.1350 | | 277.1190 | 32 |
| 63 | Beclomethasone dipropionate | C_28_H_37_ClO_7_ | 15.259 | 520.2228 | [M+H]^+^ | 521.1844 | 463.5698 | | 441.7501 | 35 |
| 64 | Betamethasone dipropionate | C_28_H_37_FO_7_ | 14.510 | 504.2523 | [M+H]^+^ | 505.2147 | 393.1525 | | 337.1380 | 35 |
| 65 | Betamethasone valerate | C_27_H_37_FO_6_ | 12.598 | 476.2574 | [M+H]^+^ | 477.2205 | 440.8481 | | 401.6354 | 32 |
| 66 | Chlormadinone acetate | C_23_H_29_ClO_4_ | 14.161 | 404.1754 | [M+H]^+^ | 405.1412 | 301.1016 | | 269.0934 | 32 |
| 67 | Clobetasol 17- propionate | C_25_H_32_ClFO_5_ | 13.903 | 466.1922 | [M+H]^+^ | 467.1559 | 459.0493 | | 427.1132 | 35 |
| 68 | Clobetasone butyrate | C_26_H_32_ClFO_5_ | 15.758 | 478.1922 | [M+H]^+^ | 479.1555 | 371.0966 | | 343.1039 | 35 |
| 69 | Cortisone | C_21_H_28_O_5_ | 6.485 | 360.1937 | [M+H]^+^ | 361.1610 | 343.1483 | | 267.1371 | 30 |
| 70 | Deflazacort | C_25_H_31_NO_6_ | 7.874 | 441.2151 | [M+H]^+^ | 442.1796 | 424.1650 | | 400.1665 | 35 |
| 71 | Dexamethasone | C_22_H_29_FO_5_ | 7.034 | 392.1999 | [M+H]^+^ | 393.1662 | 355.1491 | | 279.1350 | 30 |
| 72 | Diflorasone Diacetate | C_26_H_32_F_2_O_7_ | 11.816 | 494.2116 | [M+H]^+^ | 495.1742 | 477.9998 | | 438.5668 | 35 |
| 73 | Epitestosterone | C_19_H_28_O_2_ | 9.862 | 288.2089 | [M+H]^+^ | 289.1802 | 271.1686 | | 253.1588 | 25 |
| 74 | Fludrocortisone | C_21_H_29_FO_5_ | 6.460 | 380.1999 | [M+H]^+^ | 381.1666 | 363.1542 | | 325.1385 | 30 |
| 75 | Flumethasone | C_22_H_28_F_2_O_5_ | 7.092 | 410.1905 | [M+H]^+^ | 411.1558 | 240.9994 | | 209.0651 | 30 |
| 76 | Flumethasone pivalate | C_27_H_36_F_2_O_6_ | 14.302 | 494.2480 | [M+H]^+^ | 495.2098 | 297.0837 | | 253.0855 | 32 |
| 77 | Fluocinolone acetonide | C_24_H_30_F_2_O_6_ | 7.583 | 452.2010 | [M+H]^+^ | 453.1656 | 428.9102 | | 373.1331 | 32 |
| 78 | Flurandrenolide | C_24_H_33_FO_6_ | 7.550 | 436.2261 | [M+H]^+^ | 437.1910 | 343.1262 | | 323.1246 | 30 |
| 79 | Fluoromethalone | C_22_H_29_FO_4_ | 7.858 | 376.2050 | [M+H]^+^ | 377.1718 | 339.1531 | | 263.1052 | 28 |
| 80 | Fluticasone propionate | C_25_H_31_F_3_O_5_S | 14.327 | 500.1844 | [M+H]^+^ | 501.1465 | 429.5860 | | 420.9976 | 38 |
| 81 | Halcinonide | C_24_H_32_ClFO_5_ | 13.155 | 454.1922 | [M+H]^+^ | 455.1565 | 435.1474 | | 417.1375 | 30 |
| 82 | Hydrocortisone | C_21_H_30_O_5_ | 6.402 | 362.2093 | [M+H]^+^ | 363.1771 | 345.1616 | | 327.1539 | 32 |
| 83 | Megestrol | C_22_H_30_O_3_ | 11.026 | 342.2195 | [M+H]^+^ | 343.1881 | 325.1764 | | 282.1595 | 30 |
| 84 | Melengestrol acetate | C_25_H_32_O_4_ | 14.103 | 396.2301 | [M+H]^+^ | 397.1968 | 337.1745 | | 294.1584 | 32 |
| 85 | Methylprednisolone | C_22_H_30_O_5_ | 6.893 | 374.2093 | [M+H]^+^ | 375.1762 | 357.1384 | | 321.1405 | 32 |
| 86 | Mometasone Furoate | C_27_H_30_Cl_2_O_6_ | 14.377 | 520.1419 | [M+H]^+^ | 521.1035 | 439.7704 | | 355.1043 | 38 |
| 87 | Prednicarbate | C_27_H_36_O_8_ | 13.288 | 488.2410 | [M+H]^+^ | 489.2042 | 448.1936 | | 325.1383 | 35 |
| 88 | Testosterone | C_19_H_28_O_2_ | 8.423 | 288.2089 | [M+H]^+^ | 289.1800 | 253.1584 | | 213.1297 | 25 |
| 89 | Triamcinolone acetonide | C_24_H_31_FO_6_ | 7.309 | 434.2105 | [M+H]^+^ | 435.1750 | 397.1570 | | 339.1180 | 32 |
| 90 | Bambuterol | C_18_H_29_N_3_O_5_ | 5.507 | 367.2107 | [M+H]^+^ | 368.1784 | 313.1185 | | 295.1072 | 32 |
| 91 | Hydroxymethyl clenbuterol | C_12_H_18_Cl_2_N_2_O_2_ | 4.326 | 292.0745 | [M+H]^+^ | 293.0455 | 277.0295 | | 202.9818 | 25 |
| 92 | Clencyclohexerol | C_14_H_20_Cl_2_N_2_O_2_ | 3.927 | 318.0902 | [M+H]^+^ | 319.0597 | 301.0502 | | 243.0065 | 25 |
| 93 | Clenhexerol | C_14_H_22_Cl_2_N_2_O | 6.272 | 304.1109 | [M+H]^+^ | 305.0816 | 220.9908 | | 204.9874 | 25 |
| 94 | Clenisopenterol | C_13_H_20_Cl_2_N_2_O | 6.047 | 290.0953 | [M+H]^+^ | 291.0668 | 275.0513 | | 216.9946 | 25 |
| 95 | Clenproperol | C_11_H_16_Cl_2_N_2_O | 4.509 | 262.0640 | [M+H]^+^ | 263.0370 | 247.0209 | | 245.0247 | 22 |
| 96 | Fenoterol | C_17_H_21_NO_4_ | 3.386 | 303.1471 | [M+H]^+^ | 304.1186 | 286.1055 | | 152.0415 | 25 |
| 97 | Formoterol | C_19_H_24_N_2_O_4_ | 5.182 | 344.1736 | [M+H]^+^ | 345.1424 | 327.1296 | | 162.0230 | 28 |
| 98 | Pirbuterol | C_12_H_20_N_2_O_3_ | 0.725 | 240.1474 | [M+H]^+^ | 241.1214 | 185.0606 | | 167.0519 | 20 |
| 99 | Ractopamine | C_18_H_23_NO_3_ | 4.683 | 301.1678 | [M+H]^+^ | 302.1385 | 284.1259 | | 164.0779 | 25 |
| 100 | Ritodrine | C_17_H_21_NO_3_ | 3.652 | 287.1521 | [M+H]^+^ | 288.1235 | 270.1144 | | 232.9113 | 25 |
| 101 | Salmeterol | C_25_H_37_NO_4_ | 7.802 | 415.2723 | [M+H]^+^ | 416.2380 | 380.2173 | | 324.7764 | 32 |
| 102 | Sotalol | C_12_H_20_N_2_O_3_S | 6.047 | 272.1195 | [M+H]^+^ | 273.0919 | 255.0801 | | 213.0356 | 25 |
| 103 | Terbutaline | C_12_H_19_NO_3_ | 0.777 | 225.1365 | [M+H]^+^ | 226.1480 | 191.1117 | | 173.1027 | 20 |
| 104 | Tulobuterol | C_12_H_18_ClNO | 5.033 | 227.1077 | [M+H]^+^ | 228.0824 | 194.8658 | | 154.0145 | 20 |
| 105 | Clenpenterol | C_13_H_20_Cl_2_N_2_O | 5.340 | 290.0953 | [M+H]^+^ | 291.0665 | 273.0536 | | 204.9783 | 25 |
| 106 | 4-Acetamidophenol | C_8_H_9_NO_2_ | 2.280 | 151.0633 | [M+H]^+^ | 152.0430 | 135.0327 | | 124.9284 | 12 |
| 107 | Chlorpromazine | C_17_H_19_ClN_2_S | 7.910 | 318.0957 | [M+H]^+^ | 319.0651 | 274.0079 | | 245.9782 | 25 |
| 108 | Clopidol | C_7_H_7_Cl_2_NO | 2.854 | 190.9905 | [M+H]^+^ | 191.9670 | 155.9898 | | 116.9652 | 15 |
| 109 | Dapsone | C_12_H_12_N_2_O_2_S | 5.208 | 248.0619 | [M+H]^+^ | 249.0351 | 218.0520 | | 155.9833 | 20 |
| 110 | Carbadox | C_11_H_10_N_4_O_4_ | 4.185 | 262.0702 | [M+H]^+^ | 263.0419 | 246.7632 | | 229.0368 | 22 |
| 111 | Haloperidol | C_21_H_23_ClFNO_2_ | 6.821 | 375.1401 | [M+H]^+^ | 376.1073 | 358.0945 | | 206.0648 | 28 |
| 112 | Azaperol | C_19_H_24_FN_3_O | 4.210 | 329.1903 | [M+H]^+^ | 330.1592 | 312.1465 | | 279.8373 | 25 |
| 113 | Azaperone | C_19_H_22_FN_3_O | 4.767 | 327.1747 | [M+H]^+^ | 328.1435 | 310.1161 | | 165.0413 | 25 |
| 114 | Propionylpromazine | C_20_H_24_N_2_OS | 7.461 | 340.1609 | [M+H]^+^ | 341.1295 | 296.0718 | | 268.0419 | 25 |
| 115 | Xylazine | C_12_H_16_N_2_S | 4.983 | 220.1034 | [M+H]^+^ | 221.0781 | 164.0234 | | 147.0642 | 20 |
| 116 | Carbamazepine | C_15_H_12_N_2_O | 6.910 | 236.0950 | [M+H]^+^ | 237.0696 | 194.0649 | | 179.0416 | 20 |
| 117 | Diphenhydramine | C_17_H_21_NO | 6.569 | 255.1623 | [M+H]^+^ | 256.1355 | 168.0593 | | 167.0561 | 22 |
| 118 | Imipramine | C_19_H_24_N_2_ | 7.243 | 280.1939 | [M+H]^+^ | 281.1657 | 236.1084 | | 220.0798 | 22 |
| 119 | Sulpiride | C_15_H_23_N_3_O_4_S | 2.744 | 341.1409 | [M+H]^+^ | 342.1098 | 213.9826 | | 148.8514 | 28 |
| 120 | Zolpidem | C_19_H_21_N_3_O | 5.555 | 307.1685 | [M+H]^+^ | 308.1392 | 263.0797 | | 249.7979 | 25 |
| 121 | Fluoxetine | C_17_H_18_F_3_NO | 7.742 | 309.1340 | [M+H]^+^ | 310.1049 | 280.6974 | | 266.1055 | 25 |
| 122 | Coffeine | C_8_H_10_N_4_O_2_ | 3.412 | 194.0804 | [M+H]^+^ | 195.0574 | 180.0392 | | 150.8866 | 15 |
| 123 | Codeine | C_18_H_21_NO_3_ | 3.096 | 299.1521 | [M+H]^+^ | 300.1219 | 282.1094 | | 243.0660 | 25 |
| 124 | 1,7-Dimethylxanthine | C_7_H_8_N_4_O_2_ | 2.538 | 180.0647 | [M+H]^+^ | 181.0424 | 165.8253 | | 140.8684 | 15 |
| 125 | Chloroprocaine | C_13_H_19_ClN_2_O_2_ | 4.121 | 270.1135 | [M+H]^+^ | 271.0854 | 197.9999 | | 153.9767 | 22 |
| 126 | Cinchocaine | C_20_H_29_N_3_O_2_ | 7.339 | 343.2260 | [M+H]^+^ | 344.1948 | 271.1071 | | 228.0689 | 28 |
| 127 | Lidocaine | C_14_H_22_N_2_O | 4.445 | 234.1732 | [M+H]^+^ | 235.1467 | 218.8884 | | 195.8810 | 20 |
| 128 | Procainamide | C_13_H_21_N_3_O | 1.535 | 235.1685 | [M+H]^+^ | 236.1428 | 195.8858 | | 178.8754 | 20 |
| 129 | Procaine | C_13_H_20_N_2_O_2_ | 2.790 | 236.1525 | [M+H]^+^ | 237.1261 | 164.0418 | | 120.0194 | 20 |
| 130 | Tetracaine | C_15_H_24_N_2_O_2_ | 6.516 | 264.1838 | [M+H]^+^ | 265.1561 | 243.8050 | | 220.1008 | 22 |
| 131 | Brompheniramine | C_16_H_19_BrN_2_ | 5.761 | 318.0732 | [M+H]^+^ | 319.0426 | 244.7850 | | 227.8218 | 25 |
| 132 | Cetirizine | C_21_H_25_ClN_2_O_3_ | 7.357 | 388.1554 | [M+H]^+^ | 389.1216 | 373.9685 | | 268.7586 | 28 |
| 133 | Chlorpheniramine | C_16_H_19_ClN_2_ | 5.528 | 274.1237 | [M+H]^+^ | 275.0953 | 230.0395 | | 216.0146 | 22 |
| 134 | Fluphenazine | C_22_H_26_F_3_N_3_OS | 7.889 | 437.1749 | [M+H]^+^ | 438.1389 | 398.1245 | | 308.0344 | 32 |
| 135 | Hydroxyzine | C_21_H_27_ClN_2_O_2_ | 7.216 | 374.1761 | [M+H]^+^ | 375.1429 | 329.1936 | | 315.7050 | 28 |
| 136 | Promethazine | C_17_H_20_N_2_S | 6.917 | 284.1347 | [M+H]^+^ | 285.1060 | 240.0508 | | 225.0263 | 25 |
| 137 | Terfenadine | C_32_H_41_NO_2_ | 11.590 | 471.3137 | [M+H]^+^ | 472.2769 | 454.2623 | | 436.2532 | 35 |
| 138 | Bifonazole | C_22_H_18_N_2_ | 8.485 | 310.1470 | [M+H]^+^ | 311.1163 | 243.0816 | | 233.0753 | 25 |
| 139 | Econazole | C_18_H_15_Cl_3_N_2_O | 10.706 | 380.0250 | [M+H]^+^ | 380.9914 | 281.4564 | | 193.0209 | 28 |
| 140 | Griseofulvin | C_17_H_17_ClO_6_ | 8.211 | 352.0714 | [M+H]^+^ | 353.0390 | 321.0117 | | 285.0140 | 25 |
| 141 | Ketoconazole | C_26_H_28_Cl_2_N_4_O_4_ | 7.063 | 530.1488 | [M+H]^+^ | 531.1092 | 489.0961 | | 421.0591 | 35 |
| 142 | Naftifine | C_21_H_21_N | 7.496 | 287.1674 | [M+H]^+^ | 288.1380 | 170.0665 | | 141.0429 | 22 |
| 143 | Flunixin | C_14_H_11_F_3_N_2_O_2_ | 8.581 | 296.0773 | [M+H]^+^ | 297.0479 | 279.0356 | | 264.0149 | 25 |
| 144 | Ketotifen | C_19_H_19_NOS | 5.804 | 309.1187 | [M+H]^+^ | 310.0880 | 292.0737 | | 249.0381 | 25 |
| 145 | Lornoxicam | C_13_H_10_ClN_3_O_4_S_2_ | 7.101 | 370.9801 | [M+H]^+^ | 371.9471 | 310.7168 | | 249.9073 | 28 |
| 146 | Melitracen | C_21_H_25_N | 8.157 | 291.1987 | [M+H]^+^ | 292.1695 | 247.1122 | | 232.0899 | 25 |
| 147 | Oxaprozin | C_18_H_15_NO_3_ | 11.242 | 293.1052 | [M+H]^+^ | 294.0758 | 276.0643 | | 234.0563 | 25 |
| 148 | Antipyrine | C_11_H_12_N_2_O | 4.623 | 188.0950 | [M+H]^+^ | 189.0721 | 161.0778 | | 147.0640 | 15 |
| 149 | Sulfaguanidine | C_7_H_10_N_4_O_2_S | 1.102 | 214.0524 | [M+H]^+^ | 215.0586 | 174.0513 | | 156.0092 | 16 |
| 150 | Difloxacin hydrochloride | C_21_H_20_ClF_2_N_3_O_3_ | 5.495 | 399.3910 | [M+H]^+^ | 400.1506 | 386.1829 | | 335.7522 | 28 |
| 151 | Fleroxacin | C_17_H_18_F_3_N_3_O_3_ | 4.603 | 369.1300 | [M+H]^+^ | 370.1384 | 352.1262 | | 326.1463 | 25 |
| 152 | Enoxacin | C_15_H_17_FN_4_O_3_ | 5.819 | 320.1285 | [M+H]^+^ | 321.1380 | 256.2610 | | 233.0697 | 25 |
| 153 | Sarafloxacin hydrochloride | C_20_H_18_ClF_2_N_3_O_3_ | 5.438 | 385.3600 | [M+H]^+^ | 386.1257 | 330.1010 | | 279.2178 | 28 |
| 154 | Nadifloxacin | C_19_H_21_FN_2_O_4_ | 6.861 | 360.1485 | [M+H]^+^ | 361.1580 | 343.1440 | | 301.0962 | 25 |
| 155 | Norfloxacin | C_16_H_18_FN_3_O_3_ | 4.746 | 319.1332 | [M+H]^+^ | 320.1440 | 303.1350 | | 275.1552 | 22 |
| 156 | Gemifloxacin mesylate | C_19_H_24_FN_5_O_7_S | 5.663 | 389.1380 | [M+H]^+^ | 390.1508 | 352.1389 | | 313.1246 | 28 |
| 157 | Glipizide | C_21_H_27_N_5_O_4_S | 8.270 | 445.1784 | [M+H]^+^ | 446.1858 | 417.0209 | | 295.1891 | 28 |
| 158 | Repaglinide | C_27_H_36_N_2_O_4_ | 8.737 | 452.2675 | [M+H]^+^ | 453.2774 | 254.0788 | | 230.1887 | 28 |
| 159 | Hygromycin B | C_20_H_37_N_3_O_13_ | 15.542 | 527.2326 | [M+H]^+^ | 528.2177 | 402.2891 | | 386.1043 | 28 |
| 160 | Sulfisoxazole | C_11_H_13_N_3_O_3_S | 6.236 | 267.0678 | [M+H]^+^ | 268.0753 | 262.8363 | | 201.0602 | 20 |
| 161 | Amikacin | C_22_H_43_N_5_O_13_ | 8.556 | 585.2857 | [M+H]^+^ | 586.3882 | 277.0614 | | 196.1290 | 28 |
| 162 | Tolbutamide | C_12_H_18_N_2_O_3_S | 8.558 | 270.1038 | [M+H]^+^ | 271.1134 | 248.8089 | | 229.1017 | 22 |
| 163 | Gliclazide | C_15_H_21_N_3_O_3_S | 10.363 | 323.1304 | [M+H]^+^ | 324.1399 | 295.1872 | | 250.9905 | 25 |
| 164 | Gliquidone | C_27_H_33_N_3_O_6_S | 17.171 | 527.2090 | [M+H]^+^ | 528.2195 | 460.8050 | | 350.0309 | 28 |
| 165 | Glyburide | C_23_H_28_ClN_3_O_5_S | 13.280 | 493.1438 | [M+H]^+^ | 494.1529 | 351.1554 | | 306.0679 | 28 |
| 166 | 1-Aminohydantoin hydrochloride | C_3_H_6_ClN_3_O_2_ | 0.731 | 115.0149 | [M+H]^+^ | 116.0441 | 113.0350 | | 110.0594 | 10 |
| 167 | Pioglitazone hydrochloride | C_19_H_21_ClN_2_O_3_S | 6.134 | 356.0961 | [M+H]^+^ | 357.2609 | 309.1844 | | 266.0333 | 28 |
| 168 | Metformin hydrochloride | C_4_H_12_ClN_5_ | 0.661 | 129.0781 | [M+H]^+^ | 130.1110 | 123.1121 | | 120.0822 | 12 |
| 169 | Glimepiride | C_24_H_34_N_4_O_5_S | 13.658 | 490.2250 | [M+H]^+^ | 491.2318 | 335.1041 | | 181.0962 | 28 |
| 170 | Sulfamonomethoxine | C_11_H_12_N_4_O_3_S | 5.502 | 280.0630 | [M+H]^+^ | 281.0653 | 263.2393 | | 237.8811 | 18 |
| 171 | Sulfacetamide | C_8_H_10_N_2_O_3_S | 2.902 | 214.0412 | [M+H]^+^ | 215.0624 | 200.1235 | | 187.1020 | 16 |
| 172 | Sulfachlorpyridazine | C_10_H_9_ClN_4_O_2_S | 5.653 | 284.0135 | [M+H]^+^ | 285.2039 | 254.0531 | | 225.9036 | 20 |
| 173 | Sulfamoxole | C_11_H_13_N_3_O_3_S | 4.461 | 267.0678 | [M+H]^+^ | 268.0753 | 229.8500 | | 140.0138 | 20 |
| 174 | Sulfanitran | C_14_H_13_N_3_O_5_S | 7.363 | 335.0576 | [M+H]^+^ | 336.0639 | 320.0169 | | 230.2354 | 25 |
| 175 | Sulfameter | C_11_H_12_N_4_O_3_S | 4.699 | 280.0630 | [M+H]^+^ | 281.0699 | 215.0937 | | 188.0133 | 20 |
| 176 | Sulfamethoxazole | C_10_H_11_N_3_O_3_S | 5.667 | 253.0521 | [M+H]^+^ | 254.0602 | 226.1303 | | 186.0803 | 20 |
| 177 | Buformin hydrochloride | C_6_H_16_ClN_5_ | 1.193 | 157.1094 | [M+H]^+^ | 158.1545 | 140.1108 | | 120.0816 | 15 |
| 178 | Phenformin hydrochloride | C_10_H_16_ClN_5_ | 3.764 | 205.1094 | [M+H]^+^ | 206.1400 | 198.1021 | | 172.8601 | 20 |
| 179 | Glibornuride | C_18_H_26_N_2_O_4_S | 11.663 | 366.1613 | [M+H]^+^ | 367.1684 | 323.2234 | | 296.3280 | 25 |
| 180 | Ciprofloxacin | C_17_H_18_FN_3_O_3_ | 4.594 | 331.1332 | [M+H]^+^ | 332.1406 | 314.1292 | | 288.1525 | 25 |
| 181 | Kanamycin sulfate | C_18_H_38_N_4_O_15_S | 4.621 | 582.2054 | [M+H]^+^ | 583.1022 | 515.1002 | | 303.0494 | 35 |
| 182 | Tobramycin | C_18_H_37_N_5_O_9_ | 7.937 | 467.2591 | [M+H]^+^ | 468.1573 | 283.2609 | | 230.2435 | 28 |

**Table S3.** The screening detection limits (SDL) of the 756 database compounds in the four representative matrices.

| **No.** | **Compound** | **Tilapia** | **Grouper** | **Oyster** | **Scallop** |
| --- | --- | --- | --- | --- | --- |
|  |  | **SDL mg/kg** | **SDL mg/kg** | **SDL mg/kg** | **SDL mg/kg** |
| 1 | Allidochlor | 0.01 | 0.01 | 0.01 | 0.01 |
| 2 | Dichlormid | 0.01 | 0.01 | 0.01 | 0.01 |
| 3 | Etridiazole | 0.01 | 0.01 | 0.01 | 0.01 |
| 4 | Chlormephos | 0.01 | 0.01 | 0.01 | 0.01 |
| 5 | Propham | N.D | N.D | 0.01 | 0.01 |
| 6 | Cycloate | 0.01 | 0.01 | 0.01 | 0.01 |
| 7 | Diphenylamine | 0.01 | 0.01 | 0.01 | 0.01 |
| 8 | Chlordimeform | 0.01 | 0.01 | 0.01 | 0.01 |
| 9 | Ethalfluralin | N.D | N.D | 0.02 | 0.05 |
| 10 | Phorate | 0.01 | N.D | 0.01 | 0.01 |
| 11 | Thiometon | 0.01 | 0.01 | 0.01 | 0.01 |
| 12 | Quintozene | 0.01 | 0.01 | 0.01 | 0.01 |
| 13 | Atrazine-desethyl | 0.01 | 0.01 | 0.01 | 0.01 |
| 14 | Clomazone | 0.01 | 0.01 | 0.01 | 0.01 |
| 15 | Diazinon | 0.01 | 0.01 | 0.01 | 0.01 |
| 16 | Fonofos | 0.01 | N.D | 0.02 | 0.02 |
| 17 | Etrimfos | 0.01 | 0.01 | 0.01 | 0.01 |
| 18 | Simazine | 0.01 | 0.01 | 0.01 | 0.01 |
| 19 | Propetamphos | 0.01 | 0.01 | 0.01 | 0.01 |
| 20 | Secbumeton | 0.01 | 0.01 | 0.01 | 0.01 |
| 21 | Dichlofenthion | 0.01 | 0.01 | 0.01 | 0.01 |
| 22 | Propyzamide | 0.01 | 0.01 | 0.01 | 0.01 |
| 23 | Mexacarbate | 0.01 | 0.01 | 0.01 | 0.01 |
| 24 | Aldrin | N.D | N.D | 0.01 | 0.02 |
| 25 | Dinitramine | 0.01 | 0.01 | 0.01 | 0.01 |
| 26 | Fenchlorphos | 0.01 | 0.01 | 0.01 | 0.01 |
| 27 | Prometryn | 0.01 | 0.01 | 0.01 | 0.01 |
| 28 | Cyprazine | 0.01 | 0.01 | 0.01 | 0.01 |
| 29 | Vinclozolin | 0.01 | 0.01 | 0.01 | 0.01 |
| 30 | β-HCH | N.D | N.D | 0.01 | 0.01 |
| 31 | Metalaxyl | 0.01 | 0.01 | 0.01 | 0.01 |
| 32 | Chlorpyrifos | 0.01 | 0.01 | 0.01 | 0.01 |
| 33 | Parathion-methyl | 0.01 | 0.01 | 0.01 | 0.01 |
| 34 | Anthraquinone | 0.01 | 0.01 | 0.01 | 0.01 |
| 35 | δ-HCH | 0.01 | N.D | 0.01 | 0.01 |
| 36 | Fenthion | 0.01 | 0.01 | 0.01 | 0.01 |
| 37 | Malathion | 0.01 | 0.01 | 0.01 | 0.01 |
| 38 | Fenitrothion | 0.01 | 0.01 | 0.01 | 0.01 |
| 39 | Paraoxon-ethyl | 0.01 | 0.01 | 0.01 | 0.01 |
| 40 | Triadimefon | 0.01 | 0.01 | 0.01 | 0.01 |
| 41 | Parathion | 0.01 | 0.01 | 0.01 | 0.01 |
| 42 | Pendimethalin | N.D | 0.01 | 0.01 | 0.01 |
| 43 | Linuron | 0.01 | 0.01 | 0.01 | 0.01 |
| 44 | Chlorbenside | N.D | N.D | 0.02 | 0.02 |
| 45 | Bromophos-ethyl | N.D | N.D | 0.02 | 0.01 |
| 46 | Quinalphos | 0.01 | 0.01 | 0.01 | 0.01 |
| 47 | trans-Chlordane (γ) | 0.01 | 0.01 | 0.01 | 0.01 |
| 48 | Phenthoate | N.D | N.D | 0.01 | 0.01 |
| 49 | Metazachlor | 0.01 | 0.01 | 0.01 | 0.01 |
| 50 | Fenothiocarb | 0.01 | 0.01 | 0.01 | 0.01 |
| 51 | Prothiophos | 0.01 | 0.01 | 0.01 | 0.01 |
| 52 | Chlorflurenol-methyl | N.D | N.D | 0.02 | 0.02 |
| 53 | Dieldrin | 0.01 | 0.01 | 0.01 | 0.01 |
| 54 | Procymidone | 0.01 | 0.01 | 0.01 | 0.01 |
| 55 | Methidathion | N.D | N.D | 0.01 | 0.01 |
| 56 | Cyanazine | 0.01 | 0.01 | 0.01 | 0.01 |
| 57 | Napropamide | 0.01 | 0.01 | 0.01 | 0.01 |
| 58 | Oxadiazon | N.D | N.D | 0.01 | 0.01 |
| 59 | Fenamiphos | 0.01 | 0.01 | 0.01 | 0.01 |
| 60 | Tetrasul | 0.01 | 0.01 | 0.01 | 0.01 |
| 61 | Aramite | 0.01 | 0.01 | 0.01 | 0.01 |
| 62 | Bupirimate | 0.01 | 0.01 | 0.01 | 0.01 |
| 63 | Carboxin | 0.01 | 0.01 | 0.01 | 0.01 |
| 64 | Flutolanil | 0.01 | 0.01 | 0.01 | 0.01 |
| 65 | 4,4'-DDD | 0.01 | 0.01 | 0.01 | 0.01 |
| 66 | Ethion | 0.01 | 0.01 | 0.01 | 0.01 |
| 67 | Sulprofos | 0.01 | 0.01 | 0.01 | 0.01 |
| 68 | Etaconazole | 0.01 | 0.01 | 0.01 | 0.01 |
| 69 | Myclobutanil | 0.01 | 0.01 | 0.01 | 0.01 |
| 70 | Diclofop-methyl | 0.01 | N.D | 0.01 | 0.01 |
| 71 | Propiconazol | 0.01 | 0.01 | 0.01 | 0.01 |
| 72 | Fensulfothion | 0.01 | 0.01 | 0.01 | 0.01 |
| 73 | Bifenthrin | 0.01 | 0.01 | 0.01 | 0.01 |
| 74 | Mirex | 0.01 | 0.01 | 0.01 | 0.01 |
| 75 | Benodanil | 0.01 | 0.01 | 0.01 | 0.01 |
| 76 | Nuarimol | 0.01 | 0.01 | 0.01 | 0.01 |
| 77 | 4,4'-Methoxychlor | 0.01 | 0.01 | 0.01 | 0.01 |
| 78 | Oxadixyl | 0.01 | 0.01 | 0.01 | 0.01 |
| 79 | Tetramethrin | 0.01 | 0.01 | 0.01 | 0.01 |
| 80 | Tebuconazol | 0.01 | 0.01 | 0.01 | 0.01 |
| 81 | Norflurazon | 0.01 | 0.01 | 0.01 | 0.01 |
| 82 | Pyridaphenthion | 0.01 | 0.01 | 0.01 | 0.01 |
| 83 | Phosmet | 0.01 | 0.01 | 0.01 | 0.01 |
| 84 | Tetradifon | 0.01 | 0.01 | 0.01 | 0.01 |
| 85 | Oxycarboxin | 0.01 | 0.01 | 0.01 | 0.01 |
| 86 | cis-Permethrin | N.D | N.D | 0.02 | 0.02 |
| 87 | trans-Permethrin | N.D | N.D | 0.01 | 0.01 |
| 88 | Pyrazophos | 0.01 | 0.01 | 0.01 | 0.01 |
| 89 | Cypermethrin | 0.01 | 0.01 | 0.01 | 0.01 |
| 90 | Fenvalerate | 0.01 | 0.01 | 0.01 | 0.01 |
| 91 | Deltamethrin | 0.01 | 0.01 | 0.01 | 0.01 |
| 92 | EPTC | 0.01 | 0.01 | 0.01 | 0.01 |
| 93 | Butylate | 0.01 | 0.01 | 0.01 | 0.01 |
| 94 | Dichlobenil | N.D | N.D | 0.01 | 0.02 |
| 95 | Pebulate | 0.01 | 0.01 | 0.01 | 0.01 |
| 96 | Nitrapyrin | N.D | N.D | 0.01 | 0.01 |
| 97 | Mevinphos | 0.01 | 0.01 | 0.01 | 0.01 |
| 98 | Chloroneb | 0.01 | 0.01 | 0.01 | 0.01 |
| 99 | Tecnazene | N.D | N.D | 0.01 | 0.01 |
| 100 | Heptenophos | 0.01 | 0.01 | 0.01 | 0.01 |
| 101 | Hexachlorobenzene | N.D | N.D | 0.02 | 0.02 |
| 102 | Ethoprophos | 0.01 | 0.01 | 0.01 | 0.01 |
| 103 | Diallate | 0.01 | 0.01 | 0.01 | 0.01 |
| 104 | Propachlor | 0.01 | 0.01 | 0.01 | 0.01 |
| 105 | Triﬂuralin | 0.01 | 0.01 | 0.01 | 0.01 |
| 106 | Chlorpropham | 0.01 | 0.01 | 0.01 | 0.01 |
| 107 | Sulfotep | 0.01 | 0.01 | 0.01 | 0.01 |
| 108 | Sulfallate | 0.01 | 0.01 | 0.01 | 0.01 |
| 109 | α-BHC | 0.01 | 0.01 | 0.01 | 0.01 |
| 110 | Terbufos | 0.01 | 0.01 | 0.01 | 0.01 |
| 111 | Terbumeton | 0.01 | 0.01 | 0.01 | 0.01 |
| 112 | Profluralin | 0.01 | 0.01 | 0.01 | 0.01 |
| 113 | Dioxathion | 0.01 | 0.01 | 0.01 | 0.01 |
| 114 | Propazine | 0.01 | 0.01 | 0.01 | 0.01 |
| 115 | Chlorbufam | N.D | N.D | 0.01 | 0.01 |
| 116 | Dicloran | 0.05 | 0.05 | 0.02 | 0.02 |
| 117 | Terbuthylazine | 0.01 | 0.01 | 0.01 | 0.01 |
| 118 | Monolinuron | 0.01 | 0.01 | 0.01 | 0.01 |
| 119 | Flufenoxuron | 0.01 | 0.01 | 0.01 | 0.01 |
| 120 | Cyanophos | 0.01 | 0.01 | 0.01 | 0.01 |
| 121 | Chlorpyrifos-methyl | 0.01 | 0.01 | 0.01 | 0.01 |
| 122 | Desmetryn | 0.01 | 0.01 | 0.01 | 0.01 |
| 123 | Dimethachlor | 0.01 | 0.01 | 0.01 | 0.01 |
| 124 | Alachlor | 0.01 | 0.01 | 0.01 | 0.01 |
| 125 | Pirimiphos-methyl | 0.01 | 0.01 | 0.01 | 0.01 |
| 126 | Terbutryn | 0.01 | 0.01 | 0.01 | 0.01 |
| 127 | Thiobencarb | 0.01 | 0.01 | 0.01 | 0.01 |
| 128 | Aspon | 0.01 | 0.01 | 0.01 | 0.01 |
| 129 | Dicofol | 0.01 | 0.01 | 0.01 | 0.01 |
| 130 | Metolachlor | 0.01 | 0.01 | 0.01 | 0.01 |
| 131 | Oxychlordane | 0.01 | 0.01 | 0.01 | 0.01 |
| 132 | Pirimiphos-ethyl | 0.01 | 0.01 | 0.01 | 0.01 |
| 133 | Methoprene | 0.01 | 0.01 | 0.01 | 0.01 |
| 134 | Bromophos | 0.01 | 0.01 | 0.01 | 0.01 |
| 135 | Dichlofluanid | 0.01 | 0.01 | 0.01 | 0.01 |
| 136 | Ethofumesate | 0.01 | 0.01 | 0.01 | 0.01 |
| 137 | Isopropalin | 0.01 | 0.01 | 0.01 | 0.01 |
| 138 | α-Endosulfan | 0.01 | 0.01 | 0.05 | 0.05 |
| 139 | Propanil | 0.01 | 0.01 | 0.01 | 0.01 |
| 140 | Isofenphos | N.D | N.D | 0.01 | 0.01 |
| 141 | Crufomate | 0.01 | 0.01 | 0.01 | 0.01 |
| 142 | Chlorfenvinphos | 0.01 | 0.01 | 0.01 | 0.01 |
| 143 | cis-Chlordane (α) | 0.01 | 0.01 | 0.01 | 0.01 |
| 144 | Tolylfluanid | 0.01 | 0.01 | 0.05 | 0.01 |
| 145 | 4,4'-DDE | 0.01 | 0.01 | 0.01 | 0.01 |
| 146 | Butachlor | 0.01 | 0.01 | 0.01 | 0.01 |
| 147 | Chlozolinate | 0.01 | 0.01 | 0.01 | 0.01 |
| 148 | Crotoxyphos | 0.01 | 0.01 | 0.01 | 0.05 |
| 149 | Iodofenphos | 0.01 | 0.01 | 0.01 | 0.01 |
| 150 | Z-Tetrachlorvinphos | 0.01 | 0.01 | 0.01 | 0.01 |
| 151 | Chlorbromuron | 0.01 | 0.01 | 0.01 | 0.01 |
| 152 | Profenofos | 0.01 | 0.01 | 0.01 | 0.01 |
| 153 | Flurochloridone | 0.01 | 0.01 | 0.01 | 0.01 |
| 154 | Buprofezin | 0.01 | 0.01 | 0.01 | 0.01 |
| 155 | 2,4'-DDD | 0.01 | 0.01 | N.D | 0.05 |
| 156 | Endrin | 0.01 | 0.01 | 0.01 | 0.01 |
| 157 | Hexaconazole | 0.01 | 0.01 | 0.01 | 0.01 |
| 158 | Chlorfenson | N.D | N.D | 0.01 | 0.01 |
| 159 | 2,4'-DDT | 0.01 | 0.01 | 0.01 | 0.01 |
| 160 | Paclobutrazol | N.D | 0.01 | 0.01 | 0.01 |
| 161 | Methoprotryne | 0.01 | 0.01 | 0.01 | 0.01 |
| 162 | Erbon | 0.01 | 0.01 | 0.01 | 0.01 |
| 163 | Chloropropylate | 0.01 | 0.01 | 0.01 | 0.01 |
| 164 | Flamprop-methyl | 0.01 | 0.01 | 0.01 | 0.01 |
| 165 | Nitrofen | 0.01 | 0.01 | 0.01 | 0.01 |
| 166 | Oxyﬂuorfen | 0.01 | 0.01 | 0.01 | 0.01 |
| 167 | Chlorthiophos | 0.01 | 0.01 | 0.01 | 0.01 |
| 168 | β-Endosulfan | 0.01 | 0.01 | 0.01 | 0.01 |
| 169 | Flamprop-isopropyl | 0.01 | 0.01 | 0.01 | 0.01 |
| 170 | 4,4'-DDT | 0.01 | 0.01 | 0.01 | 0.01 |
| 171 | Carbophenothion | 0.01 | 0.01 | 0.01 | 0.01 |
| 172 | Benalaxyl | 0.01 | 0.01 | 0.02 | 0.02 |
| 173 | Edifenphos | 0.01 | 0.01 | 0.01 | 0.01 |
| 174 | Triazophos | 0.01 | 0.01 | 0.01 | 0.01 |
| 175 | Cyanofenphos | 0.01 | 0.01 | 0.01 | 0.01 |
| 176 | Chlorbenside sulfone | 0.01 | 0.01 | N.D | N.D |
| 177 | Endosulfan Sulfate | 0.01 | 0.01 | 0.01 | 0.01 |
| 178 | Bromopropylate | N.D | 0.01 | N.D | 0.01 |
| 179 | Benzoylprop-ethyl | 0.01 | 0.01 | 0.01 | 0.01 |
| 180 | Fenpropathrin | 0.01 | 0.01 | 0.01 | 0.01 |
| 181 | Leptophos | 0.01 | 0.01 | 0.01 | 0.01 |
| 182 | EPN | 0.01 | 0.01 | 0.01 | 0.01 |
| 183 | Hexazinone | 0.01 | 0.01 | 0.01 | 0.01 |
| 184 | Phosalone | 0.01 | 0.01 | 0.01 | 0.01 |
| 185 | Azinphos-methyl | 0.01 | 0.01 | 0.01 | 0.01 |
| 186 | Fenarimol | 0.01 | 0.01 | 0.01 | 0.01 |
| 187 | Azinphos-ethyl | 0.01 | N.D | 0.02 | 0.01 |
| 188 | Prochloraz | 0.01 | 0.01 | 0.01 | 0.01 |
| 189 | Coumaphos | 0.01 | 0.01 | 0.01 | 0.01 |
| 190 | Cyfluthrin | N.D | N.D | 0.05 | 0.05 |
| 191 | tau-Fluvalinate | 0.05 | 0.01 | 0.01 | 0.01 |
| 192 | Dichlorvos | 0.01 | 0.01 | 0.01 | 0.01 |
| 193 | Biphenyl | 0.01 | 0.01 | 0.01 | 0.01 |
| 194 | Vernolate | 0.01 | 0.01 | 0.01 | 0.01 |
| 195 | 3,5-Dichloroaniline | N.D | 0.05 | 0.02 | 0.02 |
| 196 | Molinate | 0.01 | 0.01 | 0.01 | 0.01 |
| 197 | E-Methacrifos | 0.01 | 0.01 | 0.01 | 0.01 |
| 198 | o-Phenylphenol | 0.01 | 0.01 | 0.01 | 0.01 |
| 199 | cis-1,2,3,6-Tetrahydrophthalimide | N.D | N.D | 0.01 | 0.01 |
| 200 | Fenobucarb | 0.01 | 0.01 | 0.01 | 0.01 |
| 201 | Benﬂuralin | 0.01 | 0.01 | 0.01 | 0.01 |
| 202 | Hexaflumuron | 0.01 | 0.01 | 0.01 | 0.01 |
| 203 | Prometon | 0.01 | 0.01 | 0.01 | 0.01 |
| 204 | Triallate | 0.01 | 0.01 | 0.01 | 0.01 |
| 205 | Pyrimethanil | 0.01 | 0.01 | 0.01 | 0.01 |
| 206 | γ-HCH | 0.01 | 0.01 | 0.01 | 0.01 |
| 207 | Disulfoton | 0.01 | 0.01 | 0.01 | 0.01 |
| 208 | Atrazine | 0.01 | 0.01 | 0.01 | 0.01 |
| 209 | Heptachlor | N.D | N.D | 0.01 | 0.01 |
| 210 | Iprobenfos | 0.01 | 0.01 | 0.01 | 0.01 |
| 211 | Isazofos | 0.01 | 0.01 | 0.01 | 0.01 |
| 212 | Plifenate | 0.01 | 0.01 | N.D | 0.02 |
| 213 | Fenpropimorph | 0.01 | 0.01 | 0.01 | 0.01 |
| 214 | Transfluthrin | 0.01 | 0.01 | 0.01 | 0.01 |
| 215 | Fluchloralin | 0.01 | 0.01 | 0.01 | 0.01 |
| 216 | Tolclofos-methyl | 0.01 | 0.01 | 0.01 | 0.01 |
| 217 | Propisochlor | 0.01 | 0.01 | 0.01 | 0.05 |
| 218 | Ametryn | 0.01 | 0.01 | 0.01 | 0.01 |
| 219 | Simetryn | 0.01 | 0.01 | 0.01 | 0.01 |
| 220 | Metobromuron | 0.01 | 0.01 | 0.01 | 0.01 |
| 221 | Metribuzin | 0.01 | 0.01 | 0.01 | 0.01 |
| 222 | Dimethipin | 0.01 | 0.01 | 0.01 | 0.01 |
| 223 | ε-HCH | 0.01 | 0.01 | 0.05 | 0.02 |
| 224 | Dipropetryn | 0.01 | 0.01 | 0.01 | 0.01 |
| 225 | Formothion | 0.01 | 0.01 | 0.01 | 0.01 |
| 226 | Diethofencarb | 0.01 | 0.01 | 0.01 | 0.01 |
| 227 | Dimepiperate | 0.01 | 0.01 | 0.01 | 0.01 |
| 228 | Bioallethrin | 0.01 | 0.05 | 0.01 | 0.01 |
| 229 | 2,4'-DDE | 0.01 | 0.01 | 0.01 | 0.01 |
| 230 | Fenson | 0.01 | 0.01 | 0.01 | 0.01 |
| 231 | Diphenamid | N.D | 0.01 | 0.01 | 0.01 |
| 232 | Chlorthion | 0.01 | 0.01 | 0.01 | 0.01 |
| 233 | Prallethrin | N.D | N.D | 0.01 | 0.01 |
| 234 | Penconazole | 0.01 | 0.01 | 0.01 | 0.01 |
| 235 | Mecarbam | 0.01 | 0.01 | 0.01 | 0.01 |
| 236 | Tetraconazole | 0.01 | 0.01 | 0.01 | 0.01 |
| 237 | Propaphos | 0.01 | 0.01 | 0.01 | 0.01 |
| 238 | Flumetralin | 0.01 | 0.01 | 0.01 | 0.02 |
| 239 | Triadimenol | 0.01 | 0.01 | 0.01 | 0.01 |
| 240 | Pretilachlor | 0.01 | 0.01 | 0.01 | 0.01 |
| 241 | Kresoxim-methyl | 0.01 | 0.01 | 0.01 | 0.01 |
| 242 | Fluazifop-butyl | 0.01 | 0.01 | 0.01 | 0.01 |
| 243 | Chlorfluazuron | 0.01 | 0.01 | 0.01 | 0.01 |
| 244 | Chlorobenzilate | N.D | N.D | 0.01 | 0.01 |
| 245 | Uniconazole | N.D | N.D | 0.01 | 0.01 |
| 246 | Flusilazole | 0.01 | 0.01 | 0.01 | 0.01 |
| 247 | Fluorodifen | N.D | N.D | 0.02 | 0.02 |
| 248 | Diniconazole | 0.01 | 0.01 | 0.01 | 0.01 |
| 249 | Piperonyl butoxide | 0.01 | 0.01 | 0.01 | 0.01 |
| 250 | Propargite | 0.01 | 0.01 | 0.01 | 0.01 |
| 251 | Mepronil | 0.01 | 0.01 | 0.01 | 0.01 |
| 252 | Dimefuron | 0.01 | 0.01 | 0.01 | 0.01 |
| 253 | Diﬂufenican | 0.01 | 0.01 | 0.01 | 0.01 |
| 254 | Fenazaquin | 0.01 | 0.01 | 0.01 | 0.01 |
| 255 | Phenothrin | 0.01 | 0.01 | 0.01 | 0.01 |
| 256 | Fludioxonil | N.D | N.D | N.D | N.D |
| 257 | Fenoxycarb | 0.01 | 0.01 | 0.01 | 0.01 |
| 258 | Sethoxydim | 0.01 | 0.02 | 0.01 | 0.01 |
| 259 | Anilofos | 0.01 | 0.01 | 0.01 | 0.01 |
| 260 | Acrinathrin | N.D | 0.01 | 0.01 | 0.01 |
| 261 | λ-Cyhalothrin | N.D | N.D | 0.02 | 0.02 |
| 262 | Mefenacet | 0.01 | 0.01 | 0.01 | 0.01 |
| 263 | Permethrin | 0.01 | 0.01 | 0.01 | 0.01 |
| 264 | Pyridaben | 0.01 | 0.01 | 0.01 | 0.01 |
| 265 | Fluoroglycofen-ethyl | 0.01 | 0.01 | 0.01 | 0.01 |
| 266 | Bitertanol | 0.01 | 0.01 | 0.01 | 0.01 |
| 267 | Etofenprox | 0.01 | 0.01 | 0.01 | 0.01 |
| 268 | Cycloxydim | 0.01 | 0.01 | 0.01 | 0.01 |
| 269 | α-Cypermethrin | 0.01 | 0.01 | 0.01 | 0.01 |
| 270 | Flucythrinate | 0.01 | 0.01 | 0.01 | 0.01 |
| 271 | Esfenvalerate | 0.01 | 0.01 | 0.02 | 0.02 |
| 272 | Difenoconazole | 0.01 | 0.01 | 0.01 | 0.01 |
| 273 | Flumioxazin | 0.01 | 0.01 | 0.01 | 0.01 |
| 274 | Flumiclorac-pentyl | 0.01 | 0.01 | 0.01 | 0.01 |
| 275 | Dimefox | 0.01 | 0.01 | 0.01 | 0.01 |
| 276 | Disulfoton-sulfoxide | 0.01 | 0.01 | 0.01 | 0.01 |
| 277 | Pentachlorobenzene | 0.01 | 0.01 | 0.01 | 0.01 |
| 278 | Triisobutyl phosphate | 0.01 | 0.01 | 0.01 | 0.01 |
| 279 | Crimidine | 0.01 | 0.01 | 0.01 | 0.01 |
| 280 | BDMC-1 | 0.01 | 0.01 | 0.02 | 0.02 |
| 281 | Chlorfenprop-methyl | 0.01 | 0.01 | 0.01 | 0.01 |
| 282 | Thionazin | 0.01 | 0.01 | 0.01 | 0.01 |
| 283 | 2,3,5,6-tetrachloroaniline | 0.01 | 0.01 | 0.01 | 0.01 |
| 284 | Tributyl phosphate | 0.01 | 0.01 | 0.01 | 0.01 |
| 285 | 2,3,4,5-Tetrachloroanisole | 0.01 | 0.01 | 0.01 | 0.01 |
| 286 | Pentachloroanisole | 0.01 | 0.01 | 0.01 | 0.01 |
| 287 | Tebutam | 0.01 | 0.01 | 0.01 | 0.01 |
| 288 | Dioxabenzofos | 0.01 | 0.01 | 0.01 | 0.01 |
| 289 | Methabenzthiazuron | 0.01 | 0.01 | 0.05 | 0.01 |
| 290 | Simeton | 0.01 | 0.01 | 0.01 | 0.01 |
| 291 | Atratone | 0.01 | 0.01 | 0.01 | 0.01 |
| 292 | Atrazine-desisopropyl | 0.01 | 0.01 | 0.01 | 0.01 |
| 293 | Terbufos sulfone | 0.01 | 0.01 | 0.01 | 0.01 |
| 294 | Tefluthrin | 0.01 | 0.01 | 0.05 | 0.05 |
| 295 | Bromocylen | 0.01 | N.D | 0.01 | 0.01 |
| 296 | Trietazine | 0.01 | 0.01 | 0.01 | 0.01 |
| 297 | Cycluron | 0.01 | 0.01 | 0.01 | 0.01 |
| 298 | 2,6-Dichlorobenzamide | 0.01 | 0.01 | 0.01 | 0.01 |
| 299 | 2,4,4'-Trichlorobiphenyl | 0.01 | 0.01 | 0.01 | 0.01 |
| 300 | 2,4,5-Trichlorobiphenyl | 0.01 | 0.01 | 0.01 | 0.01 |
| 301 | Sebuthylazine-desethyl | 0.01 | 0.01 | 0.01 | 0.01 |
| 302 | 2,3,4,5-Tetrachloroaniline | 0.01 | 0.01 | 0.01 | 0.01 |
| 303 | musk ambrette | 0.01 | 0.01 | 0.01 | 0.01 |
| 304 | Musk xylene | 0.01 | 0.01 | 0.01 | 0.01 |
| 305 | Pentachloroaniline | 0.01 | 0.01 | 0.01 | 0.01 |
| 306 | Aziprotryne | 0.01 | 0.01 | 0.01 | 0.01 |
| 307 | Sebuthylazine | 0.01 | 0.01 | 0.01 | 0.01 |
| 308 | Isocarbamid | 0.01 | 0.01 | 0.01 | 0.01 |
| 309 | 2,2',5,5'-Tetrachlorobiphenyl | 0.01 | 0.01 | 0.01 | 0.01 |
| 310 | Musk moskene | 0.01 | 0.01 | 0.01 | 0.01 |
| 311 | Prosulfocarb | 0.01 | 0.01 | 0.01 | 0.01 |
| 312 | Dimethenamid | 0.01 | 0.01 | 0.01 | 0.01 |
| 313 | Fenchlorphos-oxon | 0.01 | 0.01 | 0.01 | 0.01 |
| 314 | Paraoxon-methyl | 0.01 | 0.01 | 0.01 | 0.01 |
| 315 | Monalide | 0.01 | 0.01 | 0.01 | 0.01 |
| 316 | Tibetene musk | 0.01 | 0.01 | 0.01 | 0.01 |
| 317 | Isobenzan | 0.01 | 0.01 | 0.01 | 0.01 |
| 318 | Octachlorostyrene | 0.01 | 0.01 | 0.02 | 0.02 |
| 319 | Pyrimitate | 0.01 | 0.01 | 0.01 | 0.01 |
| 320 | Isodrin | 0.01 | 0.01 | 0.01 | 0.01 |
| 321 | Isomethiozin | 0.01 | 0.01 | 0.01 | 0.01 |
| 322 | Trichloronate | 0.01 | 0.01 | 0.01 | 0.01 |
| 323 | Chlorthal-dimethyl | 0.01 | 0.01 | 0.01 | 0.01 |
| 324 | 4,4'-Dichlorobenzophenone | 0.01 | 0.01 | 0.01 | 0.01 |
| 325 | Nitrothal-isopropyl | 0.01 | 0.01 | 0.01 | 0.01 |
| 326 | Musk ketone | N.D | N.D | 0.02 | 0.02 |
| 327 | Rabenzazol | N.D | 0.01 | 0.01 | 0.01 |
| 328 | Cyprodinil | 0.01 | 0.01 | 0.01 | 0.01 |
| 329 | Fuberidazole | 0.01 | 0.01 | 0.01 | 0.01 |
| 330 | Isofenphos-oxon | 0.01 | 0.01 | 0.01 | 0.01 |
| 331 | Dicapthon | 0.01 | 0.01 | 0.01 | 0.01 |
| 332 | 2,2',4,5,5'-Pentachlorobiphenyl | 0.01 | 0.01 | 0.01 | 0.01 |
| 333 | MCPA-butoxyethyl ester | 0.01 | 0.01 | 0.01 | 0.01 |
| 334 | Isocarbophos | 0.01 | 0.01 | 0.05 | 0.05 |
| 335 | Phorate sulfone | 0.01 | 0.01 | 0.01 | 0.01 |
| 336 | Chlorfenethol | 0.01 | 0.01 | 0.01 | 0.01 |
| 337 | trans-Nonachlor | 0.01 | 0.01 | 0.01 | 0.01 |
| 338 | Dinobuton | 0.01 | 0.01 | 0.01 | 0.01 |
| 339 | Tribufos | 0.01 | 0.01 | 0.01 | 0.01 |
| 340 | Flurochloridone | 0.01 | 0.01 | 0.02 | 0.02 |
| 341 | Bromfenvinphos | 0.01 | 0.01 | 0.01 | 0.01 |
| 342 | Perthan | 0.01 | 0.01 | 0.01 | 0.01 |
| 343 | Ditalimfos | 0.01 | 0.01 | 0.01 | 0.01 |
| 344 | 2,3,4,4',5-Pentachlorobiphenyl | 0.01 | 0.01 | 0.01 | 0.01 |
| 345 | 4,4'-Dibromobenzophenone | 0.01 | 0.01 | 0.01 | 0.01 |
| 346 | Flutriafol | 0.01 | 0.01 | 0.01 | 0.01 |
| 347 | Mephosfolan | 0.01 | 0.01 | 0.01 | 0.01 |
| 348 | Athidathion | 0.01 | 0.01 | 0.01 | 0.01 |
| 349 | 2,2',4,4',5,5'-Hexachlorobiphenyl | N.D | N.D | 0.05 | 0.05 |
| 350 | Diclobutrazol | 0.01 | 0.01 | 0.01 | 0.01 |
| 351 | Disulfoton-sulfone | 0.01 | 0.01 | 0.01 | 0.01 |
| 352 | Hexythiazox | 0.01 | 0.01 | 0.01 | 0.01 |
| 353 | 2,2',3,4,4',5'-Hexachlorobiphenyl | 0.05 | N.D | N.D | N.D |
| 354 | Triamiphos | 0.01 | 0.01 | 0.01 | 0.01 |
| 355 | Resmethrin | 0.01 | 0.01 | 0.01 | 0.01 |
| 356 | Cyproconazole | 0.01 | 0.01 | 0.01 | 0.01 |
| 357 | Benzyl butyl phthalate | 0.01 | 0.01 | 0.01 | 0.01 |
| 358 | Clodinafop-propargyl | 0.01 | 0.01 | 0.01 | 0.01 |
| 359 | Fenthion sulfoxide | 0.01 | 0.01 | 0.01 | 0.01 |
| 360 | Fluotrimazole | 0.05 | 0.01 | 0.01 | 0.01 |
| 361 | Fluroxypyr-1-methylheptyl ester | 0.01 | 0.01 | 0.01 | 0.01 |
| 362 | Fenthion-sulfone | 0.01 | 0.01 | 0.01 | 0.01 |
| 363 | Triphenyl phosphate | 0.01 | 0.01 | 0.01 | 0.01 |
| 364 | Metamitron | 0.01 | 0.01 | 0.01 | 0.01 |
| 365 | 2,2',3,4,4',5,5'-Heptachlorobiphenyl | 0.01 | 0.01 | 0.02 | 0.02 |
| 366 | Tebufenpyrad | 0.01 | 0.01 | 0.01 | 0.01 |
| 367 | Cloquintocet-mexyl | 0.01 | 0.01 | 0.01 | 0.01 |
| 368 | Lenacil | 0.01 | 0.01 | 0.01 | 0.01 |
| 369 | Bromuconazole | 0.01 | 0.01 | 0.01 | 0.01 |
| 370 | Nitralin | 0.01 | 0.01 | 0.01 | 0.01 |
| 371 | Fenamiphos sulfoxide | 0.01 | 0.01 | 0.01 | 0.01 |
| 372 | Fenamiphos sulfone | 0.01 | 0.01 | 0.01 | 0.01 |
| 373 | Fenpiclonil | 0.01 | 0.01 | 0.01 | 0.01 |
| 374 | Fluquinconazole | 0.01 | 0.01 | 0.01 | 0.01 |
| 375 | Fenbuconazole | 0.01 | 0.01 | 0.01 | 0.01 |
| 376 | Ethylene brassylate | 0.01 | 0.01 | 0.01 | 0.01 |
| 377 | Propoxur | 0.01 | 0.01 | 0.01 | 0.01 |
| 378 | Isoprocarb | 0.01 | 0.01 | 0.01 | 0.01 |
| 379 | Methamidophos | 0.01 | 0.01 | 0.01 | 0.01 |
| 380 | Acenaphthene | 0.01 | 0.01 | 0.01 | 0.01 |
| 381 | Dibutyl succinate | 0.01 | 0.01 | 0.01 | 0.01 |
| 382 | Phthalimide | 0.01 | 0.01 | 0.01 | 0.01 |
| 383 | Chlorethoxyfos | 0.01 | 0.01 | 0.01 | 0.01 |
| 384 | Pencycuron | 0.01 | 0.01 | 0.01 | 0.01 |
| 385 | Tebuthiuron | 0.01 | 0.01 | 0.01 | 0.01 |
| 386 | Demeton-S-methyl | 0.01 | 0.01 | 0.01 | 0.01 |
| 387 | Cadusafos | 0.01 | 0.01 | 0.01 | 0.01 |
| 388 | Phenanthrene | 0.01 | 0.01 | 0.01 | 0.01 |
| 389 | Spiroxamine | 0.01 | 0.01 | 0.01 | 0.01 |
| 390 | Fenpyroximate | 0.01 | 0.01 | 0.01 | 0.01 |
| 391 | Tebupirimfos | 0.01 | 0.01 | 0.01 | 0.01 |
| 392 | Prohydrojasmon | 0.01 | 0.01 | 0.01 | 0.01 |
| 393 | Fenpropidin | 0.01 | 0.01 | 0.01 | 0.01 |
| 394 | Dicloran | 0.01 | 0.01 | 0.01 | 0.01 |
| 395 | Pyroquilon | 0.01 | 0.01 | 0.01 | 0.01 |
| 396 | Propyzamide | 0.01 | 0.01 | 0.01 | 0.01 |
| 397 | Pirimicarb | 0.01 | 0.01 | 0.01 | 0.01 |
| 398 | Phosphamidon | 0.01 | 0.01 | 0.01 | 0.01 |
| 399 | Benoxacor | 0.01 | 0.01 | 0.01 | 0.01 |
| 400 | Bromobutide | 0.01 | 0.01 | 0.01 | 0.01 |
| 401 | Acetochlor | 0.01 | 0.01 | 0.01 | 0.01 |
| 402 | Tridiphane | 0.01 | 0.01 | 0.01 | 0.01 |
| 403 | Terbucarb | 0.01 | 0.01 | 0.01 | 0.01 |
| 404 | Esprocarb | 0.01 | 0.01 | 0.01 | 0.01 |
| 405 | Fenfuram | 0.01 | 0.01 | 0.01 | 0.01 |
| 406 | Acibenzolar-S-methyl | 0.01 | 0.01 | 0.05 | 0.05 |
| 407 | Benfuresate | 0.01 | 0.01 | 0.05 | 0.05 |
| 408 | Dithiopyr | 0.01 | 0.01 | 0.01 | 0.01 |
| 409 | Metalaxyl-m | 0.01 | 0.01 | 0.01 | 0.01 |
| 410 | Malaoxon | 0.01 | 0.01 | 0.01 | 0.01 |
| 411 | Simeconazole | 0.01 | 0.01 | 0.01 | 0.01 |
| 412 | Chlorthal-dimethyl | 0.01 | 0.01 | 0.01 | 0.01 |
| 413 | Thiazopyr | 0.01 | 0.01 | 0.01 | 0.01 |
| 414 | Dimethylvinphos | 0.01 | 0.01 | 0.01 | 0.01 |
| 415 | Butralin | 0.01 | 0.01 | 0.01 | 0.01 |
| 416 | Zoxamide | 0.01 | 0.01 | 0.01 | 0.01 |
| 417 | Pyrifenox | 0.01 | 0.01 | 0.01 | 0.01 |
| 418 | Allethrin | 0.01 | 0.01 | 0.01 | 0.01 |
| 419 | Dimethametryn | 0.01 | 0.01 | 0.01 | 0.01 |
| 420 | Quinoclamine | 0.01 | 0.01 | 0.01 | 0.01 |
| 421 | Methothrin | 0.02 | 0.01 | 0.01 | 0.05 |
| 422 | Flufenacet | 0.01 | 0.01 | 0.01 | 0.01 |
| 423 | Fenoxanil | 0.01 | 0.01 | 0.01 | 0.01 |
| 424 | Fthalide | 0.01 | 0.01 | 0.01 | 0.01 |
| 425 | Furalaxyl | 0.01 | 0.01 | 0.01 | 0.01 |
| 426 | Thiamethoxam | 0.01 | 0.01 | 0.01 | 0.01 |
| 427 | Mepanipyrim | 0.01 | 0.01 | 0.01 | 0.01 |
| 428 | Captan | 0.01 | 0.01 | 0.01 | 0.01 |
| 429 | Bromacil | 0.01 | 0.01 | 0.01 | 0.01 |
| 430 | Picoxystrobin | 0.01 | 0.01 | 0.01 | 0.01 |
| 431 | Butamifos | 0.01 | 0.01 | 0.01 | 0.01 |
| 432 | Imazamethabenz-methyl | 0.01 | 0.01 | 0.01 | 0.01 |
| 433 | (E)-Metominostrobin | 0.01 | 0.01 | 0.01 | 0.01 |
| 434 | TCMTB | 0.01 | 0.01 | 0.01 | 0.01 |
| 435 | Methiocarb sulfone | 0.01 | 0.01 | 0.01 | 0.01 |
| 436 | Imazalil | 0.01 | 0.01 | 0.01 | 0.01 |
| 437 | Isoprothiolane | 0.01 | 0.01 | 0.01 | 0.01 |
| 438 | Cyflufenamid | 0.01 | 0.01 | 0.01 | 0.01 |
| 439 | Pyriminobac-methyl | 0.01 | 0.01 | 0.01 | 0.05 |
| 440 | Isoxathion | 0.01 | 0.01 | 0.01 | 0.01 |
| 441 | (Z)-Metominostrobin | 0.01 | 0.01 | 0.01 | 0.01 |
| 442 | Diofenolan | 0.01 | 0.01 | 0.01 | 0.01 |
| 443 | Thifluzamide | 0.01 | 0.01 | 0.01 | 0.01 |
| 444 | Quinoxyfen | 0.01 | 0.01 | 0.01 | 0.01 |
| 445 | Chlorfenapyr | 0.01 | 0.01 | 0.01 | 0.01 |
| 446 | Trifloxystrobin | N.D | N.D | 0.01 | 0.01 |
| 447 | Imibenconazole-oxon-desbenzyl | 0.01 | 0.01 | 0.01 | 0.01 |
| 448 | Isoxadifen-ethyl | 0.01 | 0.01 | 0.01 | 0.01 |
| 449 | Fipronil | 0.01 | 0.01 | 0.01 | 0.01 |
| 450 | Imiprothrin | 0.01 | 0.01 | 0.01 | 0.01 |
| 451 | Carfentrazone-ethyl | 0.01 | 0.01 | 0.01 | 0.01 |
| 452 | Epoxiconazole | 0.01 | 0.01 | 0.01 | 0.01 |
| 453 | Pyraflufen-ethyl | 0.01 | 0.01 | 0.01 | 0.01 |
| 454 | Pyributicarb | 0.01 | 0.01 | 0.01 | 0.01 |
| 455 | Thenylchlor | 0.01 | 0.01 | 0.01 | 0.01 |
| 456 | Clethodim | 0.01 | 0.01 | 0.01 | 0.01 |
| 457 | Mefenpyr-diethyl | 0.01 | 0.01 | 0.01 | 0.01 |
| 458 | Famphur | 0.01 | 0.01 | 0.01 | 0.01 |
| 459 | Etoxazole | 0.01 | 0.01 | 0.01 | 0.01 |
| 460 | Pyriproxyfen | 0.01 | 0.01 | 0.01 | 0.01 |
| 461 | Picolinafen | 0.01 | 0.01 | 0.01 | 0.01 |
| 462 | Iprodione | 0.01 | 0.01 | 0.01 | 0.01 |
| 463 | Piperophos | 0.01 | 0.01 | 0.01 | 0.01 |
| 464 | Ofurace | 0.01 | 0.01 | 0.01 | 0.01 |
| 465 | Bifenazate | 0.01 | 0.01 | 0.01 | 0.01 |
| 466 | Endrin ketone | 0.01 | 0.01 | 0.01 | 0.01 |
| 467 | Clomeprop | 0.01 | 0.01 | 0.01 | 0.01 |
| 468 | Fenamidone | 0.01 | 0.01 | 0.01 | 0.01 |
| 469 | Naproanilide | 0.01 | 0.01 | 0.01 | 0.01 |
| 470 | Pyraclostrobin | 0.01 | 0.01 | 0.01 | 0.01 |
| 471 | Lactofen | 0.01 | 0.01 | 0.01 | 0.01 |
| 472 | Tralkoxydim | 0.01 | 0.01 | 0.01 | 0.01 |
| 473 | Pyraclofos | 0.01 | 0.01 | 0.01 | 0.01 |
| 474 | Dialifos | 0.01 | 0.01 | 0.01 | 0.01 |
| 475 | Spirodiclofen | 0.01 | 0.05 | 0.01 | 0.01 |
| 476 | Halfenprox | 0.01 | 0.01 | 0.01 | 0.01 |
| 477 | Flurtamone | 0.01 | 0.01 | 0.01 | 0.01 |
| 478 | Pyriftalid | 0.01 | 0.01 | 0.01 | 0.01 |
| 479 | Silafluofen | 0.01 | 0.01 | 0.01 | 0.01 |
| 480 | Pyrimidifen | 0.01 | 0.01 | 0.01 | 0.01 |
| 481 | Acetamiprid | 0.01 | 0.01 | 0.01 | 0.01 |
| 482 | Butafenacil | 0.01 | 0.01 | 0.01 | 0.01 |
| 483 | Cafenstrole | 0.01 | N.D | N.D | N.D |
| 484 | Fluridone | 0.01 | 0.01 | 0.01 | 0.01 |
| 485 | Heptachlor-2,3-exo-epoxide | 0.01 | 0.01 | 0.01 | 0.01 |
| 486 | Methamidophos | 0.01 | 0.01 | 0.01 | 0.01 |
| 487 | Carbofuran | 0.01 | 0.01 | 0.01 | 0.01 |
| 488 | Acetamiprid | 0.01 | 0.01 | 0.01 | 0.01 |
| 489 | Trichlorfon | 0.01 | 0.01 | 0.01 | 0.01 |
| 490 | Demeton | N.D | N.D | N.D | N.D |
| 491 | Phorate sulfoxide | 0.02 | 0.02 | 0.02 | 0.02 |
| 492 | Oxycarboxin | 0.01 | 0.01 | 0.01 | 0.01 |
| 493 | Phoxim | 0.01 | 0.01 | 0.01 | 0.01 |
| 494 | Methoxyfenozide | 0.01 | 0.01 | 0.01 | 0.01 |
| 495 | Diafenthiuron | 0.01 | 0.01 | 0.01 | 0.01 |
| 496 | Thifensulfuron-methyl | 0.01 | 0.01 | 0.01 | 0.01 |
| 497 | Ethoxysulfuron | 0.01 | 0.01 | 0.01 | 0.01 |
| 498 | Spinosad | 0.01 | 0.01 | 0.01 | 0.01 |
| 499 | Mepiquat chloride | 0.01 | 0.05 | 0.01 | 0.05 |
| 500 | Tricyclazole | 0.01 | 0.01 | 0.01 | 0.01 |
| 501 | Isoproturon | 0.01 | 0.01 | 0.01 | 0.01 |
| 502 | Pymetrozine | 0.01 | 0.01 | 0.01 | 0.01 |
| 503 | Flumetsulam | 0.01 | 0.01 | 0.01 | 0.01 |
| 504 | Thiodicarb | 0.01 | 0.01 | 0.01 | 0.01 |
| 505 | Cinosulfuron | 0.01 | 0.01 | 0.01 | 0.01 |
| 506 | Pyrazosulfuron-ethyl | 0.01 | 0.01 | 0.01 | 0.01 |
| 507 | Methomyl | 0.01 | 0.01 | 0.01 | 0.02 |
| 508 | Cymoxanil | 0.01 | 0.01 | 0.01 | 0.01 |
| 509 | Omethoate | 0.02 | 0.01 | 0.05 | 0.01 |
| 510 | Ethoxyquin | 0.01 | 0.01 | 0.01 | 0.01 |
| 511 | Aldoxycarb | 0.01 | N.D | 0.01 | 0.01 |
| 512 | Imazapic | 0.01 | 0.01 | 0.01 | 0.01 |
| 513 | Uniconazole | 0.01 | 0.01 | 0.01 | 0.01 |
| 514 | Clofentezine | 0.01 | 0.01 | 0.01 | 0.01 |
| 515 | Vamidothion sulfone | 0.01 | 0.01 | 0.01 | 0.05 |
| 516 | Terbufos sulfone | 0.01 | 0.01 | 0.01 | 0.01 |
| 517 | Cyazofamid | 0.02 | 0.01 | 0.01 | 0.01 |
| 518 | Florasulam | 0.01 | 0.01 | 0.01 | 0.01 |
| 519 | Benzoximate | 0.01 | 0.01 | 0.01 | 0.01 |
| 520 | Chlormequat chloride | 0.01 | 0.01 | 0.01 | 0.01 |
| 521 | Sethoxydim | 0.01 | 0.01 | 0.01 | 0.01 |
| 522 | Folpet | 0.01 | 0.01 | 0.01 | 0.01 |
| 523 | Methiocarb | 0.01 | 0.05 | 0.01 | 0.01 |
| 524 | Cartap hydrochloride | 0.01 | 0.01 | 0.01 | 0.01 |
| 525 | Propyl phosphate | 0.01 | 0.01 | 0.01 | 0.01 |
| 526 | Triisobutyl phosphate | 0.01 | 0.01 | 0.01 | 0.01 |
| 527 | Tris(2-butoxyethyl) phosphate | 0.01 | 0.01 | 0.01 | 0.01 |
| 528 | 2-Ethylhexyldiphenyl phosphate | 0.01 | 0.01 | 0.01 | 0.01 |
| 529 | Tris(2-ethylhexyl) Phosphate | 0.01 | 0.01 | 0.01 | 0.01 |
| 530 | Tris(3-methylphenyl) phosphate | 0.01 | 0.01 | 0.01 | 0.01 |
| 531 | Tri-p-tolyl phosphate | 0.01 | 0.01 | 0.01 | 0.01 |
| 532 | Tris(2-methylphenyl) phosphate | 0.01 | 0.01 | 0.01 | 0.01 |
| 533 | Tris(1,3-dichloroisopropyl) phosphate | 0.01 | 0.01 | 0.02 | 0.01 |
| 534 | Cresyl diphenyl phosphate | 0.01 | 0.01 | 0.01 | 0.01 |
| 535 | Phenyl phosphate | 0.01 | 0.01 | 0.01 | 0.01 |
| 536 | Tris(1-Chloro-2-Propyl) Phosphate | 0.01 | 0.01 | 0.01 | 0.01 |
| 537 | Tributyl phosphate | 0.01 | 0.01 | 0.01 | 0.01 |
| 538 | tris(2-chloroethyl) phosphate | 0.01 | 0.01 | 0.01 | 0.01 |
| 539 | Triethyl phosphate | 0.01 | 0.01 | 0.01 | 0.01 |
| 540 | Trimethyl phosphate | 0.01 | 0.01 | 0.01 | 0.01 |
| 541 | Benzyl butyl phthalate | 0.01 | 0.01 | 0.01 | 0.01 |
| 542 | [Diphenyl phthalate](https://www.chemsrc.com/en/cas/84-62-8_510543.html) | 0.01 | 0.01 | 0.01 | 0.01 |
| 543 | [β-Butoxyethyl phthalate](https://www.chemsrc.com/en/cas/117-83-9_750429.html) | 0.01 | 0.01 | 0.01 | 0.01 |
| 544 | [Dibutyl phthalate](https://www.chemsrc.com/en/cas/84-74-2_336203.html) | 0.01 | 0.01 | 0.01 | 0.01 |
| 545 | [Dicyclohexyl phthalate](https://www.chemsrc.com/en/cas/84-61-7_670264.html) | 0.01 | 0.01 | 0.01 | 0.01 |
| 546 | [Dimethyl phthalate](https://www.chemsrc.com/en/cas/131-11-3_28935.html) | 0.01 | 0.01 | 0.01 | 0.01 |
| 547 | dipentyl phthalate | 0.01 | 0.01 | 0.01 | 0.01 |
| 548 | [Diethyl phthalate](https://www.chemsrc.com/en/cas/84-66-2_401979.html) | 0.01 | 0.01 | 0.02 | 0.02 |
| 549 | Diisobutyl phthalate | 0.01 | 0.01 | 0.02 | 0.02 |
| 550 | Dihexyl phthalate | 0.01 | 0.01 | 0.01 | 0.01 |
| 551 | Dioctyl phthalate | 0.01 | 0.01 | 0.01 | 0.01 |
| 552 | Bis(2-methoxyethyl) phthalate | 0.01 | 0.01 | 0.01 | 0.01 |
| 553 | bis(2-ethoxyethyl) benzene-1,2-dicarboxylate | 0.01 | 0.01 | 0.01 | 0.01 |
| 554 | 1,2-Benzenedicarboxylic acid, 1,2-bis(1,3-dimethylbutyl) ester | 0.01 | 0.01 | 0.01 | 0.01 |
| 555 | bis(2-ethylhexyl) phthalate | 0.01 | 0.01 | 0.01 | 0.01 |
| 556 | Dinonyl phthalate | 0.01 | 0.01 | 0.01 | 0.01 |
| 557 | N-Sulfocarbamoylgonyautoxin-2 | 0.01 | 0.01 | 0.01 | 0.01 |
| 558 | N-Sulfocarbamoylgonyautoxin-3 | 0.01 | 0.01 | 0.01 | 0.01 |
| 559 | Decarbamovlgonyautoxin-2 | 0.01 | 0.01 | 0.01 | 0.01 |
| 560 | Decarbamovlgonyautoxin-3 | 0.01 | 0.01 | 0.01 | 0.01 |
| 561 | Decarbamoylneosaxitoxin dihydrochloride | 0.01 | 0.01 | 0.01 | 0.01 |
| 562 | Decarbamoylsaxitoxin | 0.01 | 0.01 | 0.01 | 0.01 |
| 563 | Gonyautoxin-1 | 0.01 | 0.01 | 0.01 | 0.01 |
| 564 | Gonyautoxin-4 | 0.01 | 0.01 | 0.01 | 0.01 |
| 565 | Gonyautoxin-2 | 0.01 | 0.01 | 0.01 | 0.01 |
| 566 | Gonyautoxin-3 | 0.01 | 0.01 | 0.01 | 0.01 |
| 567 | Gonyautoxin-6 | 0.01 | 0.01 | 0.01 | 0.01 |
| 568 | Neosaxitoxin | 0.01 | 0.01 | 0.01 | 0.01 |
| 569 | Saxitoxin dihydrochloride | 0.01 | 0.01 | 0.01 | 0.01 |
| 570 | Tetrodotoxin | 0.01 | 0.01 | 0.01 | 0.01 |
| 571 | Microcystin RR | 0.01 | 0.01 | 0.01 | 0.01 |
| 572 | Microcystin LR | 0.01 | 0.01 | 0.01 | 0.01 |
| 573 | Okadaic Acid | 0.01 | 0.01 | 0.02 | 0.02 |
| 574 | Nodularin | 0.01 | 0.01 | 0.01 | 0.01 |
| 575 | Sulfabenzamide | 0.01 | 0.01 | 0.01 | 0.01 |
| 576 | Sulfadiazine | 0.01 | 0.01 | 0.01 | 0.01 |
| 577 | Sulfadimethoxine | 0.01 | 0.01 | 0.01 | 0.01 |
| 578 | Sulfamethazine | 0.01 | 0.01 | 0.01 | 0.01 |
| 579 | Sulfadoxine | 0.01 | 0.01 | 0.01 | 0.01 |
| 580 | Sulfamerazine | 0.01 | 0.01 | 0.01 | 0.01 |
| 581 | Sulfamethizole | 0.01 | 0.01 | 0.01 | 0.01 |
| 582 | Sulfamethoxypyridazine | 0.01 | 0.01 | 0.01 | 0.01 |
| 583 | Sulfaphenazole | 0.01 | 0.01 | 0.01 | 0.01 |
| 584 | Sulfapyrazole | 0.01 | 0.01 | 0.01 | 0.01 |
| 585 | Sulfapyridine | 0.01 | 0.01 | 0.01 | 0.01 |
| 586 | Sulfaquinoxaline | 0.01 | 0.01 | 0.01 | 0.01 |
| 587 | Sulfathiazole | 0.01 | 0.01 | 0.01 | 0.01 |
| 588 | Sulfisomidine | 0.01 | 0.01 | 0.01 | 0.01 |
| 589 | Trimethoprim | 0.01 | 0.01 | 0.01 | 0.01 |
| 590 | Cinoxacin | 0.01 | 0.01 | 0.01 | 0.01 |
| 591 | Danofloxacin | 0.01 | 0.01 | 0.01 | 0.01 |
| 592 | Difloxacin | 0.01 | 0.01 | 0.01 | 0.01 |
| 593 | Enrofloxacin | 0.01 | 0.01 | 0.01 | 0.01 |
| 594 | Flumequine | 0.01 | 0.01 | 0.01 | 0.01 |
| 595 | Gatifloxacin | 0.01 | 0.01 | 0.01 | 0.01 |
| 596 | Lomefloxacin | 0.01 | 0.05 | 0.01 | 0.01 |
| 597 | Marbofloxacin | 0.01 | 0.01 | 0.01 | 0.01 |
| 598 | Moxifloxacin | 0.01 | 0.01 | 0.01 | 0.01 |
| 599 | Nalidixic acid | 0.01 | 0.01 | 0.01 | 0.01 |
| 600 | Ofloxacin | 0.01 | 0.01 | 0.01 | 0.01 |
| 601 | Orbifloxacin | 0.01 | 0.01 | 0.01 | 0.01 |
| 602 | Oxolinic acid | 0.01 | 0.01 | 0.01 | 0.01 |
| 603 | Sarafloxacin | 0.01 | 0.01 | 0.01 | 0.01 |
| 604 | Sparfloxacin | 0.01 | N.D | 0.01 | N.D |
| 605 | Tosufloxacin | 0.01 | 0.01 | 0.01 | 0.01 |
| 606 | Albendazole | 0.01 | 0.01 | 0.01 | 0.01 |
| 607 | Albendazole-2-aminosulfone | 0.01 | 0.01 | 0.01 | 0.01 |
| 608 | Albendazole sulfoxide | N.D | 0.01 | 0.01 | 0.01 |
| 609 | Mebendazole-amine | 0.01 | 0.01 | 0.01 | 0.01 |
| 610 | Thiabendazole | 0.01 | 0.01 | 0.01 | 0.01 |
| 611 | Dimetridazole | 0.01 | 0.01 | 0.01 | 0.01 |
| 612 | Fenbendazole | 0.01 | 0.01 | 0.01 | 0.01 |
| 613 | Flubendazole | 0.01 | 0.01 | 0.01 | 0.01 |
| 614 | Hydroxy ipronidazole | 0.01 | 0.01 | 0.01 | 0.01 |
| 615 | Ipronidazole | 0.01 | 0.01 | 0.01 | 0.01 |
| 616 | Mebendazole | 0.01 | 0.01 | 0.01 | 0.01 |
| 617 | Cambendazole | 0.01 | 0.01 | 0.01 | 0.01 |
| 618 | 5-Hydroxythiabendazole | 0.01 | 0.01 | 0.01 | 0.01 |
| 619 | Levamisole | 0.01 | 0.01 | 0.01 | 0.01 |
| 620 | Metronidazole | 0.01 | 0.01 | 0.01 | 0.01 |
| 621 | Oxfendazole | N.D | 0.01 | 0.01 | 0.01 |
| 622 | Oxibendazole | 0.01 | 0.01 | 0.01 | 0.01 |
| 623 | Ronidazole | 0.01 | 0.01 | 0.01 | 0.01 |
| 624 | Secnidazole | 0.01 | 0.01 | 0.01 | 0.01 |
| 625 | Tinidazole | 0.01 | 0.01 | 0.01 | 0.01 |
| 626 | Triclabendazole | 0.01 | 0.01 | 0.01 | 0.01 |
| 627 | Clindamycin | 0.01 | 0.01 | 0.01 | 0.01 |
| 628 | Doramectin | 0.01 | 0.01 | 0.01 | 0.01 |
| 629 | Eprinomectin | 0.01 | 0.01 | 0.02 | 0.02 |
| 630 | Ivermectin | 0.01 | 0.01 | 0.01 | 0.02 |
| 631 | Leucomycin A1 | 0.01 | 0.01 | 0.01 | 0.01 |
| 632 | Spiramycin | 0.01 | 0.01 | 0.01 | 0.01 |
| 633 | Tilmicosin | 0.01 | 0.01 | 0.01 | 0.01 |
| 634 | Tylosin | 0.01 | 0.01 | 0.01 | 0.01 |
| 635 | Virginiamycin M1 | 0.01 | 0.01 | 0.01 | 0.01 |
| 636 | Beclomethasone | 0.01 | 0.01 | 0.01 | 0.01 |
| 637 | Beclomethasone dipropionate | 0.01 | 0.01 | 0.01 | 0.01 |
| 638 | Betamethasone dipropionate | 0.01 | 0.01 | 0.01 | 0.01 |
| 639 | Betamethasone valerate | 0.01 | 0.01 | 0.01 | 0.01 |
| 640 | Chlormadinone acetate | 0.01 | 0.01 | 0.01 | 0.01 |
| 641 | Clobetasol 17- propionate | 0.01 | 0.01 | 0.01 | 0.01 |
| 642 | Clobetasone butyrate | 0.01 | 0.01 | 0.01 | 0.01 |
| 643 | Cortisone | 0.01 | 0.01 | 0.01 | 0.01 |
| 644 | Deflazacort | 0.01 | 0.01 | 0.01 | 0.01 |
| 645 | Dexamethasone | 0.01 | 0.01 | 0.01 | 0.01 |
| 646 | Diflorasone Diacetate | 0.01 | 0.01 | 0.01 | 0.01 |
| 647 | Epitestosterone | 0.01 | 0.01 | 0.01 | 0.01 |
| 648 | Fludrocortisone | 0.01 | 0.01 | 0.01 | 0.01 |
| 649 | Flumethasone | 0.01 | 0.01 | 0.01 | 0.01 |
| 650 | Flumethasone pivalate | 0.01 | 0.01 | 0.01 | 0.01 |
| 651 | Fluocinolone acetonide | 0.01 | 0.01 | 0.01 | 0.01 |
| 652 | Flurandrenolide | 0.01 | 0.01 | 0.01 | 0.01 |
| 653 | Fluoromethalone | 0.01 | 0.01 | 0.01 | 0.01 |
| 654 | Fluticasone propionate | 0.01 | 0.01 | 0.01 | 0.01 |
| 655 | Halcinonide | 0.01 | 0.01 | 0.01 | 0.01 |
| 656 | Hydrocortisone | 0.01 | 0.01 | 0.01 | 0.01 |
| 657 | Megestrol | 0.01 | 0.01 | 0.01 | 0.01 |
| 658 | Melengestrol acetate | 0.01 | 0.01 | 0.01 | 0.01 |
| 659 | Methylprednisolone | 0.01 | 0.01 | 0.01 | 0.01 |
| 660 | Mometasone Furoate | 0.01 | 0.01 | 0.01 | 0.01 |
| 661 | Prednicarbate | 0.01 | 0.01 | 0.01 | 0.01 |
| 662 | Testosterone | 0.01 | 0.01 | 0.01 | 0.01 |
| 663 | Triamcinolone acetonide | 0.01 | 0.01 | 0.01 | 0.01 |
| 664 | Bambuterol | 0.01 | 0.01 | 0.01 | 0.01 |
| 665 | Hydroxymethyl clenbuterol | 0.01 | 0.01 | 0.01 | 0.01 |
| 666 | Clencyclohexerol | 0.01 | 0.01 | 0.01 | 0.01 |
| 667 | Clenhexerol | 0.01 | 0.01 | 0.01 | 0.01 |
| 668 | Clenisopenterol | 0.01 | 0.01 | 0.01 | 0.01 |
| 669 | Clenproperol | 0.01 | 0.01 | 0.01 | 0.01 |
| 670 | Fenoterol | 0.01 | 0.01 | 0.01 | 0.01 |
| 671 | Formoterol | 0.01 | 0.01 | 0.01 | 0.01 |
| 672 | Pirbuterol | 0.01 | 0.01 | 0.01 | 0.01 |
| 673 | Ractopamine | 0.01 | 0.01 | 0.01 | 0.01 |
| 674 | Ritodrine | 0.01 | 0.01 | 0.01 | 0.01 |
| 675 | Salmeterol | 0.01 | 0.01 | 0.01 | 0.01 |
| 676 | Sotalol | 0.01 | 0.01 | 0.01 | 0.01 |
| 677 | Terbutaline | 0.01 | 0.01 | 0.01 | 0.01 |
| 678 | Tulobuterol | 0.01 | 0.01 | 0.01 | 0.01 |
| 679 | Clenpenterol | 0.01 | 0.01 | 0.01 | 0.01 |
| 680 | 4-Acetamidophenol | 0.01 | 0.01 | 0.01 | 0.01 |
| 681 | Chlorpromazine | 0.01 | 0.01 | 0.01 | 0.01 |
| 682 | Clopidol | 0.01 | 0.01 | 0.01 | 0.01 |
| 683 | Dapsone | 0.01 | 0.01 | 0.01 | 0.01 |
| 684 | Carbadox | 0.01 | 0.01 | 0.01 | 0.01 |
| 685 | Haloperidol | 0.01 | 0.01 | 0.01 | 0.01 |
| 686 | Azaperol | 0.01 | 0.01 | 0.01 | 0.01 |
| 687 | Azaperone | 0.01 | 0.01 | 0.01 | 0.01 |
| 688 | Propionylpromazine | 0.01 | 0.01 | 0.01 | 0.01 |
| 689 | Xylazine | 0.01 | 0.01 | 0.01 | 0.01 |
| 690 | Carbamazepine | 0.01 | 0.01 | 0.01 | 0.01 |
| 691 | Diphenhydramine | 0.01 | 0.01 | 0.01 | 0.01 |
| 692 | Imipramine | 0.01 | 0.01 | 0.01 | 0.01 |
| 693 | Sulpiride | 0.01 | 0.01 | 0.01 | 0.01 |
| 694 | Zolpidem | 0.01 | 0.01 | 0.01 | 0.01 |
| 695 | Fluoxetine | 0.01 | 0.01 | 0.01 | 0.01 |
| 696 | Coffeine | 0.01 | 0.01 | 0.01 | 0.01 |
| 697 | Codeine | 0.01 | 0.01 | 0.01 | 0.01 |
| 698 | 1,7-Dimethylxanthine | 0.01 | 0.01 | 0.01 | 0.01 |
| 699 | Chloroprocaine | N.D | 0.01 | 0.01 | 0.01 |
| 700 | Cinchocaine | 0.01 | 0.01 | 0.01 | 0.01 |
| 701 | Lidocaine | 0.01 | 0.01 | 0.01 | 0.01 |
| 702 | Procainamide | 0.01 | 0.01 | 0.01 | 0.01 |
| 703 | Procaine | 0.01 | 0.01 | 0.01 | 0.01 |
| 704 | Tetracaine | 0.01 | 0.01 | 0.01 | 0.01 |
| 705 | Brompheniramine | 0.01 | 0.01 | 0.01 | 0.01 |
| 706 | Cetirizine | 0.01 | 0.01 | 0.01 | 0.01 |
| 707 | Chlorpheniramine | 0.01 | 0.01 | 0.01 | 0.01 |
| 708 | Fluphenazine | 0.01 | 0.01 | 0.01 | 0.01 |
| 709 | Hydroxyzine | 0.01 | 0.01 | 0.01 | 0.01 |
| 710 | Promethazine | 0.01 | 0.01 | 0.01 | 0.01 |
| 711 | Terfenadine | 0.01 | 0.01 | 0.01 | 0.01 |
| 712 | Bifonazole | 0.01 | 0.01 | 0.01 | 0.01 |
| 713 | Econazole | 0.01 | 0.01 | 0.01 | 0.01 |
| 714 | Griseofulvin | 0.01 | 0.01 | 0.01 | 0.01 |
| 715 | Ketoconazole | 0.01 | 0.01 | 0.01 | 0.01 |
| 716 | Naftifine | 0.01 | 0.01 | 0.01 | 0.01 |
| 717 | Flunixin | 0.01 | 0.01 | 0.01 | 0.01 |
| 718 | Ketotifen | 0.01 | 0.02 | 0.01 | 0.01 |
| 719 | Lornoxicam | 0.01 | 0.01 | 0.01 | 0.01 |
| 720 | Melitracen | 0.01 | 0.01 | 0.01 | 0.01 |
| 721 | Oxaprozin | 0.01 | 0.01 | 0.01 | 0.01 |
| 722 | Antipyrine | 0.01 | 0.01 | 0.01 | 0.01 |
| 723 | Sulfaguanidine | 0.01 | 0.01 | 0.01 | 0.01 |
| 724 | Difloxacin hydrochloride | 0.01 | 0.01 | 0.01 | 0.01 |
| 725 | Fleroxacin | 0.01 | 0.01 | 0.01 | 0.01 |
| 726 | Enoxacin | 0.01 | 0.01 | 0.01 | 0.01 |
| 727 | Sarafloxacin hydrochloride | 0.01 | 0.01 | 0.01 | 0.01 |
| 728 | Nadifloxacin | 0.01 | 0.02 | 0.01 | 0.01 |
| 729 | Norfloxacin | 0.01 | 0.01 | 0.01 | 0.01 |
| 730 | Gemifloxacin mesylate | 0.01 | 0.01 | 0.01 | 0.01 |
| 731 | Glipizide | 0.01 | 0.01 | 0.01 | 0.01 |
| 732 | Repaglinide | 0.01 | 0.01 | 0.01 | 0.01 |
| 733 | Hygromycin B | 0.01 | 0.02 | 0.01 | 0.01 |
| 734 | Sulfisoxazole | 0.01 | 0.01 | 0.01 | 0.01 |
| 735 | Amikacin | 0.01 | 0.01 | 0.01 | 0.01 |
| 736 | Tolbutamide | 0.01 | 0.01 | 0.01 | 0.01 |
| 737 | Gliclazide | 0.01 | 0.01 | 0.01 | 0.01 |
| 738 | Gliquidone | 0.01 | 0.01 | 0.01 | 0.01 |
| 739 | Glyburide | 0.01 | 0.01 | 0.01 | 0.01 |
| 740 | 1-Aminohydantoin hydrochloride | 0.01 | 0.01 | 0.05 | 0.02 |
| 741 | Pioglitazone hydrochloride | 0.01 | 0.01 | 0.01 | 0.01 |
| 742 | Metformin hydrochloride | 0.01 | 0.01 | 0.01 | 0.01 |
| 743 | Glimepiride | 0.01 | 0.01 | 0.01 | 0.01 |
| 744 | Sulfamonomethoxine | 0.01 | 0.01 | 0.01 | 0.01 |
| 745 | Sulfacetamide | 0.01 | N.D | 0.01 | 0.01 |
| 746 | Sulfachlorpyridazine | 0.01 | 0.01 | 0.01 | 0.01 |
| 747 | Sulfamoxole | 0.01 | 0.01 | 0.01 | 0.01 |
| 748 | Sulfanitran | 0.01 | 0.01 | 0.01 | 0.01 |
| 749 | Sulfameter | 0.01 | 0.01 | 0.01 | 0.01 |
| 750 | Sulfamethoxazole | 0.01 | 0.01 | 0.01 | 0.01 |
| 751 | Buformin hydrochloride | 0.01 | 0.01 | 0.01 | 0.01 |
| 752 | Phenformin hydrochloride | 0.01 | 0.01 | 0.01 | 0.01 |
| 753 | Glibornuride | 0.01 | 0.01 | 0.01 | 0.01 |
| 754 | Ciprofloxacin | 0.01 | 0.01 | 0.01 | 0.01 |
| 755 | Kanamycin sulfate | N.D | N.D | 0.05 | 0.05 |
| 756 | Tobramycin | N.D | N.D | 0.01 | 0.01 |

**Table S4.** The recoveries and relative standard deviations (RSD) for the 756 database compounds in the four representative matrices at different spiking levels.

| **No.** | **Compound name** | **Tilapia** | | | **Grouper** | | | **Oyster** | | | **Scallop** | | |
| --- | --- | --- | --- | --- | --- | --- | --- | --- | --- | --- | --- | --- | --- |
|  |  | **Recovery, % (RSD, %)** | | | **Recovery, % (RSD, %)** | | | **Recovery, % (RSD, %)** | | | **Recovery, % (RSD, %)** | | |
|  |  | **10 μg/kg** | **20 μg/kg** | **50 μg/kg** | **10 μg/kg** | **20 μg/kg** | **50 μg/kg** | **10 μg/kg** | **20 μg/kg** | **50 μg/kg** | **10 μg/kg** | **20 μg/kg** | **50 μg/kg** |
| 1 | Allidochlor | 86.25(2.8) | 90.25(6.1) | 92.58(3.5) | 91.25(3.8) | 92.58(2.9) | 95.28(5.4) | 85.35(11.3) | 82.49(7.4) | 79.33(4.6) | 89.25(4.3) | 87.34(6.4) | 85.49(4.7) |
| 2 | Dichlormid | 70.25(5.9) | 78.35(5.2) | 80.23(6.2) | 80.14(2.6) | 81.2(5.1) | 85.31(2.9) | 95.35(8.7) | 93.64(4.3) | 91.22(7.2) | 98.34(7.2) | 95.38(5.9) | 91.25(7.3) |
| 3 | Etridiazole | 81.24(5.4) | 82.45(7.0) | 85.76(6.1) | 94.25(5.4) | 90.21(5.4) | 88.21(4.8) | 85.35(7.7) | 83.24(5.2) | 80.79(3.7) | 94.25(8.6) | 91.46(6.4) | 88.21(4.2) |
| 4 | Chlormephos | 98.25(3.2) | 100.25(3.4) | 102.24(5.2) | 80.21(2.8) | 82.54(5.1) | 85.24(7.5) | 94.25(7.7) | 91.47(5.4) | 89.37(4.3) | 91.55(7.1) | 88.34(6.3) | 86.47(5.4) |
| 5 | Propham | N.D | N.D | N.D | N.D | N.D | N.D | 91.55(7.4) | 95.47(5.3) | 88.34(3.0) | 90.28(7.2) | 88.25(8.5) | 86.39(4.4) |
| 6 | Cycloate | 91.25(5.1) | 95.24(5.1) | 97.52(6.4) | 91.25(3.5) | 85.24(4.8) | 78.25(6.7) | 89.35(7.5) | 86.37(4.3) | 84.26(3.5) | 93.55(10.3) | 93.64(8.7) | 90.21(5.4) |
| 7 | Diphenylamine | 76.28(3.8) | 79.38(6.5) | 82.21(6.8) | 90.24(3.4) | 95.28(6.8) | 97.36(7.1) | 93.65(8.6) | 91.25(7.4) | 88.44(3.3) | 99.78(6.5) | 95.46(7.3) | 93.67(1.6) |
| 8 | Chlordimeform | 92.58(4.5) | 93.25(5.4) | 94.58(6.1) | 77.63(4.2) | 80.25(6.7) | 86.74(9.5) | 94.55(9.6) | 91.47(4.3) | 88.43(3.3) | 104.37(6.4) | 100.25(4.7) | 98.37(5.5) |
| 9 | Ethalfluralin | N.D | N.D | N.D | N.D | N.D | N.D | N.D | 74.26(17.5) | 75.38(17.0) | N.D | N.D | 70.21(19.5) |
| 10 | Phorate | 74.21(2.7) | 76.24(6.2) | 79.21(8.7) | N.D | N.D | N.D | 91.45(8.1) | 88.45(5.4) | 86.34(4.3) | 87.19(8.3) | 86.24(6.6) | 84.37(4.2) |
| 11 | Thiometon | 90.54(6.4) | 88.16(7.5) | 86.74(8.1) | 97.28(6.1) | 95.87(5.5) | 90.24(8.1) | 68.55(16.3) | 72.45(13.2) | 75.64(13.0) | 73.55(16.4) | 75.34(14.7) | 77.54(10.4) |
| 12 | Quintozene | 75.84(6.8) | 77.28(6.7) | 80.98(5.7) | 99.57(6.1) | 102.67(4.8) | 90.84(9.7) | 82.35(8.5) | 80.29(4.6) | 78.35(2.6) | 97.68(10.4) | 96.54(8.6) | 94.21(5.2) |
| 13 | Atrazine-desethyl | 70.28(3.5) | 75.84(6.2) | 77.89(6.7) | 72.89(5.6) | 76.24(6.1) | 82.45(7.1) | 88.35(6.3) | 86.59(4.4) | 84.57(2.6) | 106.57(5.5) | 102.45(6.4) | 99.54(6.4) |
| 14 | Clomazone | 90.87(3.4) | 92.54(3.6) | 98.63(2.5) | 85.63(7.8) | 87.54(4.8) | 89.75(6.7) | 96.55(8.8) | 95.48(9.5) | 93.15(4.6) | 91.48(8.5) | 89.38(5.7) | 87.55(3.7) |
| 15 | Diazinon | 97.55(3.4) | 98.75(6.1) | 100.2(5.4) | 72.54(2.5) | 80.36(4.4) | 82.56(4.5) | 94.55(7.3) | 91.85(5.4) | 89.66(7.2) | 97.54(6.4) | 95.34(4.3) | 92.87(3.7) |
| 16 | Fonofos | 90.54(3.5) | 92.44(4.8) | 96.87(8.7) | N.D | N.D | N.D | N.D | 80.74(8.7) | 82.64(5.4) | N.D | 110.48(13.6) | 113.67(10.5) |
| 17 | Etrimfos | 92.45(3.9) | 95.48(5.4) | 96.87(6.5) | 88.76(5.1) | 85.47(3.4) | 82.19(6.4) | 91.45(7.0) | 89.67(5.7) | 87.46(6.4) | 96.54(6.4) | 94.28(5.4) | 91.57(3.3) |
| 18 | Simazine | 85.64(4.8) | 88.54(4.5) | 90.54(5.4) | 103.21(4.1) | 100.52(5.4) | 95.5(6.5) | 94.25(8.3) | 93.46(6.2) | 91.28(4.0) | 98.25(8.0) | 96.38(6.5) | 94.25(3.2) |
| 19 | Propetamphos | 66.87(6.5) | 72.51(6.3) | 85.24(3.5) | 93.54(4.1) | 95.68(3.5) | 99.87(6.4) | 88.25(8.7) | 86.39(7.5) | 84.25(4.1) | 91.58(8.6) | 89.67(7.1) | 84.25(3.5) |
| 20 | Secbumeton | 71.25(13.8) | 78.65(3.9) | 85.61(5.8) | 73.25(4.1) | 76.34(5.5) | 80.46(6.5) | 107.5(6.4) | 104.38(8.0) | 101.34(6.4) | 78.96(6.3) | 75.28(5.5) | 74.84(4.9) |
| 21 | Dichlofenthion | 80.67(4.8) | 82.56(6.5) | 88.65(6.4) | 82.67(6.5) | 80.68(6.4) | 77.68(5.4) | 96.55(5.9) | 94.72(3.7) | 91.47(4.4) | 97.84(6.4) | 95.41(5.2) | 93.47(4.0) |
| 22 | Propyzamide | 90.25(4.2) | 91.65(6.5) | 84.36(2.8) | 92.87(6.4) | 95.87(4.8) | 99.87(9.8) | 91.25(7.6) | 89.65(5.3) | 86.59(6.4) | 97.48(7.5) | 96.31(6.0) | 94.28(3.7) |
| 23 | Mexacarbate | 102.56(3.5) | 95.87(5.4) | 80.62(7.4) | 97.55(4.6) | 94.68(6.4) | 90.54(8.7) | 99.35(5.2) | 98.67(3.5) | 96.28(6.3) | 88.59(7.5) | 86.52(4.4) | 84.15(2.6) |
| 24 | Aldrin | N.D | N.D | N.D | N.D | N.D | N.D | 89.65(7.7) | 87.41(6.4) | 85.27(3.2) | N.D | 72.48(15.3) | 73.49(11.3) |
| 25 | Dinitramine | 92.54(4.5) | 95.47(6.4) | 99.87(9.7) | 73.84(6.4) | 79.84(6.4) | 85.74(6.4) | 97.45(8.3) | 95.24(7.7) | 93.72(4.3) | 107.96(4.3) | 105.24(5.4) | 102.25(5.5) |
| 26 | Fenchlorphos | 91.58(5.1) | 95.64(7.1) | 90.87(6.4) | 81.04(3.8) | 85.97(4.1) | 88.54(3.8) | 89.55(6.3) | 87.59(6.3) | 85.24(3.2) | 74.58(16.4) | 73.59(15.2) | 75.89(13.9) |
| 27 | Prometryn | 85.14(3.2) | 80.24(3.8) | 89.67(4.8) | 94.28(2.9) | 95.47(6.4) | 98.75(5.4) | 95.65(4.6) | 93.87(4.0) | 91.47(6.4) | 90.25(8.1) | 87.26(6.3) | 85.94(5.5) |
| 28 | Cyprazine | 83.41(4.2) | 84.27(7.4) | 80.54(6.5) | 92.54(5.4) | 88.64(3.5) | 90.54(6.7) | 86.15(8.0) | 86.39(4.3) | 83.47(4.1) | 108.95(6.4) | 105.38(4.4) | 103.24(6.6) |
| 29 | Vinclozolin | 86.47(6.2) | 88.74(6.1) | 84.25(6.7) | 94.84(6.1) | 92.14(6.9) | 90.13(9.7) | 96.35(7.4) | 95.88(5.4) | 91.25(4.3) | 98.63(4.6) | 95.38(3.1) | 92.78(6.5) |
| 30 | β-HCH | N.D | N.D | N.D | N.D | N.D | N.D | 98.65(7.1) | 96.45(5.9) | 94.27(3.7) | 98.64(5.2) | 96.38(7.6) | 93.54(2.5) |
| 31 | Metalaxyl | 96.87(4.1) | 95.04(5.9) | 92.15(5.8) | 103.54(2.6) | 98.57(7.4) | 95.12(6.7) | 91.55(8.5) | 93.67(9.6) | 88.27(4.5) | 89.36(5.3) | 87.54(7.3) | 85.29(5.2) |
| 32 | Chlorpyrifos | 92.87(6.5) | 90.51(4.8) | 94.87(7.4) | 82.44(3.8) | 85.15(7.8) | 80.59(3.9) | 105.5(8.3) | 102.34(7.2) | 99.64(6.2) | 96.38(8.5) | 95.27(4.3) | 93.47(3.6) |
| 33 | Parathion-methyl | 101.54(2.9) | 98.74(4.9) | 95.28(9.6) | 79.84(2.8) | 85.74(6.5) | 88.63(6.7) | 97.55(3.7) | 98.74(6.3) | 93.48(5.2) | 87.64(5.3) | 85.69(7.2) | 88.63(5.7) |
| 34 | Anthraquinone | 90.85(2.6) | 85.74(6.8) | 83.12(5.9) | 89.14(3.5) | 85.41(6.4) | 84.12(6.5) | 93.55(8.0) | 90.24(8.3) | 88.24(7.5) | 94.28(7.6) | 91.29(6.5) | 89.67(5.3) |
| 35 | δ-HCH | 103.84(3.8) | 98.14(5.1) | 90.56(6.2) | N.D | N.D | N.D | 89.65(7.3) | 86.34(6.4) | 84.57(3.6) | 88.54(6.4) | 86.27(5.2) | 85.34(4.3) |
| 36 | Fenthion | 71.68(2.9) | 80.48(6.2) | 85.25(9.1) | 95.48(2.5) | 88.41(3.8) | 80.14(6.1) | 95.65(6.6) | 93.74(8.5) | 90.27(7.0) | 92.35(4.6) | 90.28(5.7) | 88.23(3.2) |
| 37 | Malathion | 82.14(6.0) | 85.14(3.9) | 85.04(6.2) | 85.14(2.1) | 88.14(6.5) | 80.15(4.9) | 92.15(7.3) | 90.24(8.2) | 88.37(6.5) | 94.28(5.4) | 91.27(7.3) | 88.54(4.7) |
| 38 | Fenitrothion | 74.15(4.2) | 77.09(6.2) | 83.65(6.8) | 99.54(3.1) | 103.25(5.1) | 95.21(6.1) | 105.5(6.4) | 102.69(7.2) | 99.68(5.3) | 93.48(6.3) | 90.25(5.5) | 87.32(4.9) |
| 39 | Paraoxon-ethyl | 82.15(6.1) | 85.64(7.8) | 89.45(5.4) | 70.54(3.5) | 76.25(4.5) | 80.12(6.8) | 88.75(11.6) | 86.52(9.3) | 85.26(4.3) | 85.26(8.3) | 82.67(6.5) | 80.24(9.4) |
| 40 | Triadimefon | 102.67(2.2) | 100.24(3.1) | 93.25(5.8) | 64.15(23.6) | 70.54(12.8) | 76.19(3.7) | 88.25(15.6) | 86.95(17.2) | 83.29(13.0) | 96.58(8.3) | 94.28(7.4) | 91.24(4.3) |
| 41 | Parathion | 94.87(3.8) | 91.25(5.4) | 95.25(6.6) | 75.36(3.8) | 77.65(6.4) | 80.12(10.7) | 95.25(7.2) | 93.41(6.6) | 91.25(3.3) | 90.28(7.5) | 88.26(5.5) | 86.37(3.9) |
| 42 | Pendimethalin | N.D | N.D | N.D | 102.54(3.6) | 98.65(6.5) | 94.13(6.5) | 96.35(7.5) | 94.25(5.4) | 91.68(2.8) | 96.35(6.3) | 95.47(5.7) | 93.82(3.6) |
| 43 | Linuron | 79.68(3.9) | 85.64(6.2) | 84.26(7.2) | 75.81(16.2) | 81.54(3.6) | 84.63(6.9) | 85.25(6.4) | 86.59(7.5) | 82.31(3.7) | 84.57(14.0) | 85.96(11.5) | 80.47(8.8) |
| 44 | Chlorbenside | N.D | N.D | N.D | N.D | N.D | N.D | N.D | 85.64(11.7) | 86.26(8.4) | N.D | 74.52(6.3) | 76.58(11.6) |
| 45 | Bromophos-ethyl | N.D | N.D | N.D | N.D | N.D | N.D | N.D | 117.85(10.4) | 116.52(13.0) | 94.75(5.4) | 92.18(6.3) | 90.19(8.3) |
| 46 | Quinalphos | 91.58(6.8) | 88.15(6.1) | 84.97(9.1) | 100.25(4.8) | 95.12(5.2) | 90.25(6.5) | 95.65(4.3) | 92.87(7.6) | 89.97(5.3) | 91.28(3.7) | 89.74(6.3) | 87.14(5.0) |
| 47 | trans-Chlordane (γ) | 95.68(6.5) | 90.87(3.9) | 85.41(6.8) | 86.24(3.5) | 90.21(4.5) | 100.23(5.8) | 93.55(9.1) | 90.25(7.4) | 87.49(6.3) | 88.76(8.5) | 86.25(6.4) | 85.77(4.4) |
| 48 | Phenthoate | N.D | N.D | N.D | N.D | N.D | N.D | 105.5(6.4) | 102.48(5.3) | 98.74(6.6) | 100.23(5.6) | 97.48(6.4) | 95.41(5.3) |
| 49 | Metazachlor | 87.15(2.9) | 90.65(6.8) | 92.15(6.8) | 94.85(3.8) | 90.15(8.5) | 87.15(6.9) | 88.75(8.2) | 86.35(7.4) | 84.19(6.5) | 92.86(7.4) | 90.28(6.3) | 87.15(5.0) |
| 50 | Fenothiocarb | 83.65(2.9) | 84.23(7.3) | 87.62(6.89) | 85.21(2.6) | 88.32(6.01) | 93.58(2.63) | 86.35(9.6) | 84.12(8.4) | 81.49(5.8) | 79.84(9.7) | 77.52(8.0) | 76.29(6.4) |
| 51 | Prothiophos | 98.2(6.9) | 96.52(3.1) | 95.78(6.9) | 100.63(2.9) | 103.85(6.8) | 106.87(4.5) | 83.75(8.7) | 86.57(7.2) | 82.67(6.3) | 96.54(5.2) | 93.74(4.2) | 92.08(7.0) |
| 52 | Chlorflurenol-methyl | N.D | N.D | N.D | N.D | N.D | N.D | N.D | 72.54(16.6) | 73.24(14.2) | N.D | 73.96(15.2) | 74.29(12.9) |
| 53 | Dieldrin | 83.65(2.9) | 84.23(7.3) | 87.62(6.89) | 85.21(3.2) | 88.32(6.01) | 93.58(8.9) | 78.25(6.5) | 76.21(4.6) | 74.98(3.3) | 96.57(8.5) | 95.31(4.6) | 93.28(2.5) |
| 54 | Procymidone | 103.56(3.5) | 98.86(4.5) | 90.58(8.3) | 75.46(2.8) | 77.85(4.8) | 82.69(5.9) | 106.5(7.5) | 104.26(5.0) | 103.47(5.2) | 94.63(5.2) | 92.68(3.6) | 90.22(7.2) |
| 55 | Methidathion | N.D | N.D | N.D | N.D | N.D | N.D | 75.35(4.7) | 77.35(6.2) | 74.38(5.2) | 77.56(9.3) | 76.29(8.4) | 74.12(3.5) |
| 56 | Cyanazine | 105.87(3.8) | 98.78(6.9) | 92.58(5.98) | 85.67(4.32) | 88.78(4.98) | 90.85(3.7) | 86.55(7.2) | 85.69(7.0) | 82.31(4.5) | 97.26(4.2) | 95.38(6.3) | 93.26(7.0) |
| 57 | Napropamide | 91.84(4.7) | 89.34(8.2) | 95.7(9.7) | 78.12(7.4) | 82.98(6.89) | 85.62(12.9) | 95.25(7.7) | 93.64(8.5) | 91.24(5.1) | 97.48(5.1) | 94.77(8.1) | 91.06(4.3) |
| 58 | Oxadiazon | N.D | N.D | N.D | N.D | N.D | N.D | 88.45(7.6) | 85.37(5.1) | 83.06(4.1) | 95.87(7.3) | 92.85(5.5) | 89.63(4.1) |
| 59 | Fenamiphos | 79.68(3.8) | 82.57(6.8) | 85.94(6.7) | 74.81(2.1) | 76.87(3.8) | 80.59(5.9) | 88.55(11.4) | 86.34(8.8) | 85.94(5.3) | 89.67(7.4) | 88.45(5.2) | 85.39(4.3) |
| 60 | Tetrasul | 105.64(3.8) | 98.41(6.8) | 90.24(4.5) | 91.58(2.8) | 88.64(3.69) | 86.19(3.8) | 72.25(14.7) | 75.63(11.0) | 78.64(8.3) | 78.59(8.2) | 80.24(6.4) | 82.64(5.2) |
| 61 | Aramite | 94.87(2.9) | 90.28(3.9) | 91.28(4.2) | 70.28(4.6) | 73.85(6.4) | 80.21(12.6) | 106.5(6.3) | 104.27(6.4) | 101.54(3.8) | 92.57(8.2) | 90.47(5.4) | 88.16(3.8) |
| 62 | Bupirimate | 93.84(7.5) | 90.85(6.2) | 88.79(4.9) | 89.51(2.9) | 92.84(3.8) | 100.85(6.8) | 113.5(7.0) | 110.28(5.4) | 107.69(4.2) | 88.69(4.7) | 85.34(6.2) | 82.57(5.4) |
| 63 | Carboxin | 97.54(3.8) | 95.28(4.8) | 90.58(6.8) | 103.85(6.5) | 100.85(7.9) | 94.86(10.8) | 92.65(6.4) | 95.28(8.0) | 89.37(4.8) | 110.52(6.3) | 106.37(4.7) | 102.47(3.6) |
| 64 | Flutolanil | 87.15(3.9) | 90.58(5.2) | 93.58(8.9) | 95.48(5.8) | 97.28(8.5) | 87.28(11.8) | 115.5(8.5) | 112.34(10.5) | 108.75(6.4) | 67.94(6.4) | 70.25(7.4) | 73.69(11.8) |
| 65 | 4,4'-DDD | 89.67(5.3) | 86.15(6.7) | 83.19(3.8) | 79.54(4.1) | 83.18(5.6) | 86.94(6.8) | 89.65(8.8) | 86.15(6.7) | 83.19(3.8) | 93.64(8.1) | 91.24(5.2) | 88.64(7.3) |
| 66 | Ethion | 79.54(5.6) | 82.15(3.9) | 84.59(6.9) | 110.58(3.6) | 100.58(6.9) | 90.48(7.9) | 79.55(15.7) | 82.15(13.9) | 80.57(9.8) | 108.76(6.4) | 105.46(5.4) | 102.39(4.9) |
| 67 | Sulprofos | 74.52(12.9) | 73.48(16.5) | 77.54(7.9) | 83.15(2.9) | 85.75(3.9) | 88.47(14.6) | 85.65(9.0) | 83.48(11.6) | 80.56(9.4) | 98.25(8.3) | 95.64(7.3) | 93.46(6.4) |
| 68 | Etaconazole | 96.87(3.8) | 94.86(4.9) | 90.28(10.2) | 108.45(3.6) | 102.57(5.8) | 94.87(11.2) | 76.35(15.6) | 78.25(13.2) | 80.54(10.5) | 95.28(9.4) | 92.35(4.7) | 90.28(6.4) |
| 69 | Myclobutanil | 82.78(6.5) | 84.75(6.7) | 78.75(18.4) | 98.41(6.3) | 94.87(6.5) | 90.16(9.4) | 88.25(9.3) | 90.58(7.6) | 84.26(6.3) | 88.69(7.3) | 86.59(9.0) | 85.21(7.4) |
| 70 | Diclofop-methyl | 97.85(3.9) | 95.25(5.6) | 90.24(6.8) | N.D | N.D | N.D | 96.55(7.5) | 95.25(4.3) | 93.68(6.4) | 105.92(6.3) | 102.54(5.3) | 98.56(6.0) |
| 71 | Propiconazol | 100.2(4.2) | 97.58(3.7) | 93.87(5.7) | 91.42(5.1) | 89.54(6.7) | 86.45(8.8) | 89.65(7.6) | 92.46(5.4) | 88.26(4.1) | 90.54(6.3) | 89.54(7.0) | 86.45(8.3) |
| 72 | Fensulfothion | 79.68(5.4) | 82.57(6.8) | 95.48(8.4) | 98.45(4.1) | 103.5(5.9) | 105.9(8.4) | 86.55(7.3) | 86.34(4.6) | 84.16(3.9) | 80.21(14.7) | 79.38(11.5) | 77.23(8.3) |
| 73 | Bifenthrin | 94.87(5.1) | 90.24(6.5) | 88.75(10.2) | 74.18(6.7) | 80.18(7.4) | 82.14(9.8) | 94.65(6.4) | 92.45(7.5) | 89.67(6.0) | 84.26(13.5) | 82.79(11.0) | 79.62(13.5) |
| 74 | Mirex | 90.54(6.4) | 92.54(6.8) | 95.48(8.7) | 97.58(3.8) | 94.88(5.87) | 87.57(8.7) | 96.25(5.5) | 94.21(4.4) | 92.76(5.2) | 96.37(6.6) | 93.48(5.9) | 90.27(4.2) |
| 75 | Benodanil | 82.54(6.5) | 84.67(7.9) | 89.54(6.2) | 108.36(4.8) | 102.8(6.8) | 94.47(8.4) | 87.65(7.4) | 85.29(5.7) | 83.25(3.0) | 85.69(8.0) | 83.46(4.3) | 81.49(5.4) |
| 76 | Nuarimol | 92.58(3.5) | 88.14(2.8) | 86.14(7.6) | 98.45(5.8) | 95.48(6.9) | 93.42(4.8) | 102.5(6.5) | 99.63(7.5) | 97.52(4.4) | 98.45(7.0) | 95.48(5.3) | 93.42(2.6) |
| 77 | 4,4'-Methoxychlor | 76.48(6.5) | 80.15(7.5) | 82.75(10.5) | 79.52(3.8) | 83.47(8.56) | 85.47(9.5) | 86.35(11.3) | 84.76(8.6) | 82.75(5.4) | 78.54(6.3) | 76.25(5.5) | 77.49(8.7) |
| 78 | Oxadixyl | 110.8(3.8) | 105.87(4.9) | 99.87(8.6) | 95.87(6.8) | 93.47(7.1) | 86.86(8.4) | 93.55(6.4) | 90.56(7.3) | 88.23(5.5) | 88.19(5.2) | 86.37(6.5) | 84.29(7.2) |
| 79 | Tetramethrin | 92.85(4.8) | 90.15(5.3) | 88.14(6.9) | 89.52(6.5) | 92.58(3.9) | 85.23(10.9) | 94.25(6.5) | 91.57(5.6) | 89.37(7.5) | 96.38(7.5) | 94.26(5.2) | 91.37(6.3) |
| 80 | Tebuconazol | 77.56(6.1) | 80.52(3.9) | 83.15(10.2) | 82.54(6.9) | 85.47(9.6) | 78.54(6.8) | 89.65(7.5) | 87.26(5.4) | 84.29(6.4) | 82.54(7.6) | 80.29(6.3) | 78.54(4.2) |
| 81 | Norflurazon | 88.48(3.2) | 85.17(6.9) | 89.25(4.6) | 94.28(2.1) | 95.87(6.9) | 90.18(10.2) | 93.45(7.2) | 95.28(7.4) | 90.28(4.2) | 91.68(9.5) | 88.37(6.5) | 85.32(4.1) |
| 82 | Pyridaphenthion | 87.19(3.2) | 90.18(6.8) | 94.7(12.1) | 95.18(4.5) | 90.18(8.9) | 88.17(6.9) | 87.15(9.4) | 90.18(6.3) | 85.34(8.8) | 89.36(6.4) | 92.58(7.4) | 95.68(8.4) |
| 83 | Phosmet | 82.49(5.1) | 77.98(6.8) | 90.18(6.9) | 76.58(3.9) | 80.76(5.4) | 83.49(5.4) | 82.55(8.4) | 80.64(7.4) | 78.69(5.3) | 99.38(6.4) | 97.46(9.0) | 94.26(10.7) |
| 84 | Tetradifon | 74.89(5.4) | 79.15(6.8) | 83.15(6.9) | 84.75(6.4) | 86.18(3.9) | 90.25(9.4) | 93.65(6.6) | 91.25(7.0) | 88.76(4.1) | 85.93(14.8) | 83.26(11.3) | 81.94(8.7) |
| 85 | Oxycarboxin | 77.89(4.5) | 79.58(6.3) | 82.67(8.6) | 106.5(3.9) | 103.48(5.6) | 95.22(9.4) | 88.95(7.3) | 85.27(5.4) | 82.67(4.2) | 88.97(8.5) | 86.59(6.3) | 84.39(7.6) |
| 86 | cis-Permethrin | N.D | N.D | N.D | N.D | N.D | N.D | N.D | 83.54(8.0) | 80.26(5.4) | N.D | 102.59(6.4) | 103.49(8.3) |
| 87 | trans-Permethrin | N.D | N.D | N.D | N.D | N.D | N.D | 98.65(5.4) | 96.34(4.3) | 92.65(4.3) | 88.95(7.5) | 85.37(11.3) | 88.39(8.1) |
| 88 | Pyrazophos | 104.8(4.3) | 100.8(8.7) | 97.18(6.9) | 91.85(4.2) | 88.76(6.9) | 94.85(2.9) | 91.25(4.3) | 95.67(7.3) | 87.46(5.3) | 75.26(8.5) | 74.12(6.3) | 79.68(8.9) |
| 89 | Cypermethrin | 72.19(6.9) | 75.48(7.2) | 81.76(5.9) | 83.54(5.4) | 85.21(6.9) | 88.45(6.9) | 91.35(7.6) | 88.68(4.6) | 85.73(3.2) | 60.28(19.7) | 68.76(16.4) | 70.49(14.3) |
| 90 | Fenvalerate | 85.47(4.9) | 86.17(6.4) | 91.25(7.2) | 72.58(4.2) | 75.25(6.5) | 77.49(3.9) | 93.25(7.6) | 91.22(4.0) | 88.34(5.6) | 81.27(12.6) | 79.46(8.2) | 85.14(6.4) |
| 91 | Deltamethrin | 80.49(5.4) | 77.98(6.1) | 90.18(6.5) | 86.58(3.2) | 80.76(6.8) | 90.25(6.9) | 107.5(6.3) | 104.66(5.4) | 99.63(2.7) | 92.45(7.1) | 89.67(5.3) | 95.48(10.7) |
| 92 | EPTC | 70.28(3.9) | 73.48(5.1) | 76.95(6.5) | 87.94(2.6) | 89.18(4.3) | 91.48(6.1) | 88.65(9.4) | 86.21(7.3) | 85.21(5.0) | 79.51(8.7) | 76.58(7.0) | 73.18(4.6) |
| 93 | Butylate | 69.58(16.4) | 75.63(16.9) | 77.96(7.9) | 70.49(12.3) | 75.48(6.1) | 79.64(7.2) | 95.85(11.6) | 92.18(8.4) | 89.37(6.0) | 82.49(8.6) | 78.45(7.3) | 76.94(5.7) |
| 94 | Dichlobenil | N.D | N.D | N.D | N.D | N.D | N.D | 96.35(4.3) | 93.68(7.8) | 90.87(5.1) | N.D | 77.49(11.8) | 78.25(8.4) |
| 95 | Pebulate | 81.65(6.1) | 83.94(7.3) | 88.49(6.5) | 75.48(2.9) | 77.93(5.6) | 80.16(3.9) | 94.85(9.0) | 91.25(7.7) | 88.49(3.6) | 101.58(6.4) | 97.45(3.6) | 95.84(5.3) |
| 96 | Nitrapyrin | N.D | N.D | N.D | N.D | N.D | N.D | 96.55(5.9) | 94.27(6.4) | 90.24(8.3) | 104.68(7.3) | 99.38(4.3) | 96.28(5.2) |
| 97 | Mevinphos | 90.84(5.6) | 92.87(6.5) | 96.84(3.8) | 105.64(3.2) | 107.68(4.8) | 101.74(3.6) | 99.65(10.4) | 95.28(8.0) | 93.68(4.3) | 95.28(7.5) | 91.28(6.5) | 88.78(5.3) |
| 98 | Chloroneb | 80.65(3.6) | 83.85(7.4) | 87.96(7.9) | 74.85(3.3) | 75.48(6.5) | 79.56(7.2) | 92.15(11.5) | 90.28(8.3) | 88.49(7.0) | 82.49(8.2) | 81.26(6.4) | 80.16(4.2) |
| 99 | Tecnazene | N.D | N.D | N.D | N.D | N.D | N.D | 88.95(8.7) | 85.74(6.2) | 83.67(4.8) | 93.64(9.2) | 91.24(7.2) | 88.74(5.2) |
| 100 | Heptenophos | 90.68(3.9) | 95.87(6.9) | 98.17(3.9) | 84.65(3.2) | 87.49(6.5) | 92.54(4.5) | 98.15(9.6) | 96.28(6.5) | 92.64(5.0) | 93.64(7.5) | 91.05(8.7) | 87.49(5.2) |
| 101 | Hexachlorobenzene | N.D | N.D | N.D | N.D | N.D | N.D | N.D | 83.94(8.5) | 85.67(4.3) | N.D | 77.93(5.1) | 78.16(3.2) |
| 102 | Ethoprophos | 80.52(4.8) | 83.69(7.6) | 87.65(8.6) | 91.85(3.2) | 96.54(6.7) | 98.74(8.2) | 91.55(7.3) | 88.24(9.3) | 87.65(5.2) | 83.64(4.5) | 80.24(5.2) | 77.15(3.6) |
| 103 | Diallate | 70.15(13.2) | 73.85(14.8) | 77.68(9.6) | 90.65(3.6) | 92.85(5.8) | 88.75(4.6) | 86.25(8.5) | 84.76(6.4) | 82.75(3.2) | 105.28(7.2) | 102.48(4.3) | 95.24(3.9) |
| 104 | Propachlor | 83.41(6.3) | 85.67(6.8) | 88.62(6.5) | 90.21(4.1) | 88.25(6.9) | 93.48(6.1) | 96.35(7.2) | 94.28(4.8) | 91.02(3.7) | 94.86(4.3) | 92.38(7.3) | 89.63(5.4) |
| 105 | Triﬂuralin | 90.25(3.5) | 92.85(4.6) | 93.87(3.9) | 84.12(3.6) | 88.64(5.4) | 90.58(6.2) | 98.55(9.4) | 96.47(7.3) | 92.64(4.2) | 89.35(7.6) | 88.64(4.2) | 84.27(6.3) |
| 106 | Chlorpropham | 70.35(4.5) | 73.26(6.4) | 75.96(4.8) | 80.23(6.3) | 83.65(6.5) | 85.76(6.3) | 94.25(7.7) | 91.22(6.4) | 88.49(3.2) | 78.98(4.3) | 75.23(9.3) | 72.64(2.5) |
| 107 | Sulfotep | 81.59(6.2) | 84.69(6.1) | 88.74(7.2) | 84.38(2.6) | 87.65(2.9) | 89.31(11.3) | 89.65(8.5) | 87.49(5.5) | 85.21(2.6) | 109.36(5.9) | 106.37(6.4) | 103.26(7.7) |
| 108 | Sulfallate | 108.52(6.3) | 93.65(4.8) | 92.51(2.6) | 99.85(3.6) | 94.25(3.6) | 88.84(2.3) | 108.5(7.5) | 105.24(6.6) | 102.87(5.5) | 93.45(5.2) | 94.25(6.5) | 89.67(4.4) |
| 109 | α-BHC | 72.48(3.6) | 75.62(5.6) | 78.69(3.4) | 80.25(4.5) | 83.45(6.2) | 85.64(3.9) | 95.45(7.3) | 93.46(5.8) | 91.28(4.2) | 98.57(6.4) | 97.42(8.7) | 95.28(7.4) |
| 110 | Terbufos | 90.25(5.1) | 92.54(4.2) | 91.54(6.3) | 66.25(13.8) | 70.15(14.5) | 73.58(6.9) | 78.55(6.7) | 76.43(4.6) | 75.82(1.7) | 65.28(14.6) | 69.38(11.6) | 73.58(8.5) |
| 111 | Terbumeton | 73.56(5.2) | 75.86(4.9) | 75.19(3.8) | 88.45(2.6) | 90.15(4.9) | 92.85(6.6) | 87.55(7.2) | 89.63(8.7) | 85.62(3.8) | 105.26(7.5) | 102.74(4.3) | 99.78(6.4) |
| 112 | Profluralin | 81.45(6.7) | 84.59(8.6) | 87.15(4.9) | 74.56(6.5) | 77.48(7.4) | 81.49(8.2) | 95.65(7.5) | 96.38(5.5) | 91.58(7.3) | 94.28(6.4) | 91.47(5.3) | 88.57(7.1) |
| 113 | Dioxathion | 83.45(2.6) | 84.18(5.3) | 86.49(2.8) | 79.58(6.5) | 82.45(7.6) | 88.49(6.5) | 85.25(8.0) | 84.18(4.3) | 81.67(6.5) | 88.25(9.7) | 86.37(4.6) | 82.67(5.2) |
| 114 | Propazine | 66.75(6.5) | 70.15(6.5) | 72.46(5.9) | 108.25(5.8) | 110.25(9.8) | 113.87(7.9) | 92.55(4.2) | 90.15(8.2) | 95.28(5.6) | 95.86(7.5) | 97.48(8.3) | 92.68(5.2) |
| 115 | Chlorbufam | N.D | N.D | N.D | N.D | N.D | N.D | 97.45(8.3) | 95.28(4.2) | 91.32(2.9) | 87.59(6.4) | 84.59(8.7) | 81.49(2.1) |
| 116 | Dicloran | 50.12(22.4) | 59.48(25.4) | 70.85(13.9) | N.D | N.D | 73.49(6.9) | N.D | 105.28(15.5) | 104.28(9.0) | N.D | 74.85(11.5) | 73.49(12.6) |
| 117 | Terbuthylazine | 73.48(16.5) | 78.16(6.9) | 83.15(8.1) | 79.48(5.5) | 83.46(7.6) | 86.49(6.4) | 89.65(13.6) | 86.35(9.5) | 85.72(4.7) | 89.76(7.9) | 87.46(5.3) | 86.49(4.7) |
| 118 | Monolinuron | 87.14(3.6) | 85.16(7.8) | 71.29(9.4) | 87.02(6.8) | 89.24(6.6) | 92.16(6.5) | 98.65(6.4) | 96.34(5.3) | 94.78(6.8) | 97.02(6.8) | 95.28(4.7) | 91.28(3.7) |
| 119 | Flufenoxuron | 86.15(4.2) | 88.19(5.8) | 91.78(6.5) | 74.19(3.2) | 77.49(8.1) | 79.62(8.6) | 91.25(9.4) | 88.19(4.9) | 86.99(3.9) | 101.54(6.3) | 98.77(4.4) | 96.38(6.5) |
| 120 | Cyanophos | 77.49(6.2) | 79.15(10.6) | 85.49(6.3) | 95.84(2.6) | 90.48(7.6) | 91.76(6.8) | 104.5(8.3) | 101.47(4.3) | 98.63(7.5) | 104.27(8.3) | 101.26(5.7) | 97.65(7.4) |
| 121 | Chlorpyrifos-methyl | 94.51(5.1) | 90.65(11.6) | 87.14(6.5) | 90.76(3.2) | 92.54(3.9) | 95.84(4.2) | 96.35(9.2) | 94.28(5.7) | 91.62(4.6) | 94.58(11.3) | 91.62(7.4) | 88.36(6.5) |
| 122 | Desmetryn | 64.18(15.5) | 68.18(16.4) | 75.29(3.8) | 83.12(6.9) | 85.16(3.9) | 89.60(5.9) | 86.35(8.2) | 88.36(10.9) | 84.26(6.3) | 105.26(7.1) | 102.49(4.9) | 98.68(9.3) |
| 123 | Dimethachlor | 104.25(4.1) | 98.14(6.5) | 99.18(10.6) | 82.19(1.6) | 85.64(3.9) | 92.48(5.6) | 103.5(11.3) | 101.68(8.8) | 98.63(5.9) | 88.39(5.7) | 85.64(8.3) | 83.59(7.3) |
| 124 | Alachlor | 80.26(3.9) | 85.14(11.6) | 88.95(6.9) | 71.58(8.1) | 75.16(3.9) | 79.84(5.2) | 75.35(8.3) | 72.46(4.3) | 73.62(8.2) | 83.59(16.3) | 80.57(14.9) | 79.84(14.3) |
| 125 | Pirimiphos-methyl | 83.19(8.6) | 85.14(7.9) | 87.09(6.3) | 92.68(4.9) | 95.46(8.3) | 99.10(7.6) | 87.35(12.7) | 85.14(9.7) | 87.09(5.4) | 98.77(8.2) | 95.46(9.4) | 96.87(6.4) |
| 126 | Terbutryn | 105.26(6.3) | 96.25(7.2) | 93.48(5.2) | 85.19(3.6) | 87.16(6.8) | 90.28(5.6) | 88.95(7.3) | 86.53(5.5) | 84.75(2.9) | 78.52(6.4) | 75.49(8.0) | 80.54(9.3) |
| 127 | Thiobencarb | 84.96(6.2) | 88.75(3.6) | 87.16(3.9) | 75.18(5.9) | 77.19(3.9) | 79.19(5.9) | 91.55(7.6) | 88.75(6.5) | 87.16(5.9) | 89.65(7.1) | 91.46(6.4) | 88.26(2.7) |
| 128 | Aspon | 92.65(5.4) | 90.78(3.9) | 88.16(6.4) | 86.52(11.3) | 88.49(7.1) | 89.17(6.6) | 88.65(7.5) | 86.25(6.3) | 85.29(4.9) | 99.67(8.3) | 97.46(6.1) | 95.62(8.7) |
| 129 | Dicofol | 87.14(4.6) | 89.52(7.6) | 92.48(6.1) | 82.09(3.3) | 88.51(5.6) | 92.78(6.5) | 87.15(8.7) | 89.52(10.3) | 92.48(7.5) | 94.52(13.7) | 91.48(11.6) | 89.37(7.1) |
| 130 | Metolachlor | 65.28(10.6) | 69.99(14.9) | 73.49(8.9) | 86.15(6.8) | 88.96(6.7) | 89.49(10.6) | 99.45(10.7) | 98.62(13.5) | 95.26(4.6) | 95.26(6.4) | 98.96(7.4) | 94.58(10.3) |
| 131 | Oxychlordane | 84.75(5.3) | 86.54(9.8) | 90.25(6.8) | 72.15(7.9) | 76.84(14.6) | 80.25(4.6) | 89.25(13.7) | 86.54(11.5) | 84.75(7.3) | 86.53(4.3) | 84.26(1.7) | 82.25(4.6) |
| 132 | Pirimiphos-ethyl | 74.26(5.8) | 79.18(7.1) | 82.16(6.8) | 90.56(5.5) | 88.15(6.7) | 91.86(6.9) | 107.5(6.4) | 104.59(8.6) | 106.39(6.5) | 99.25(5.5) | 97.48(7.4) | 94.86(6.9) |
| 133 | Methoprene | 71.49(6.6) | 78.16(6.9) | 82.96(9.5) | 83.95(7.1) | 88.69(7.8) | 90.15(6.9) | 97.65(7.6) | 99.64(10.2) | 95.23(4.7) | 94.87(8.3) | 93.25(6.4) | 90.15(7.3) |
| 134 | Bromophos | 97.16(6.2) | 99.85(8.6) | 102.94(9.7) | 93.85(2.7) | 95.84(6.9) | 88.19(6.4) | 75.25(14.3) | 78.65(16.4) | 74.25(11.3) | 89.65(7.3) | 91.26(5.7) | 85.32(3.5) |
| 135 | Dichlofluanid | 85.49(6.9) | 88.95(6.9) | 90.25(7.5) | 79.25(2.3) | 82.59(8.1) | 86.29(5.2) | 107.5(9.6) | 103.26(8.2) | 99.36(5.4) | 95.26(6.3) | 92.67(8.5) | 90.36(2.1) |
| 136 | Ethofumesate | 94.58(5.6) | 91.28(7.2) | 88.15(11.2) | 74.16(6.3) | 77.18(3.6) | 76.95(6.5) | 98.55(9.4) | 95.24(8.2) | 88.15(12.4) | 89.67(6.4) | 87.18(3.7) | 86.95(2.6) |
| 137 | Isopropalin | 70.29(5.1) | 72.89(6.4) | 76.59(3.6) | 90.15(6.2) | 88.16(4.8) | 84.65(5.6) | 97.65(8.4) | 96.38(7.2) | 94.58(5.3) | 88.69(8.9) | 83.16(7.5) | 84.65(9.6) |
| 138 | α-Endosulfan | 83.16(7.1) | 88.49(6.1) | 94.32(5.9) | 83.19(3.5) | 85.16(11.6) | 87.49(5.8) | N.D | N.D | 72.15(16.4) | N.D | N.D | 97.48(15.6) |
| 139 | Propanil | 83.54(2.6) | 85.64(6.4) | 88.15(6.3) | 101.15(5.1) | 97.18(4.6) | 99.63(5.1) | 96.35(6.4) | 95.64(4.3) | 91.58(7.6) | 97.58(8.3) | 95.26(6.4) | 93.47(2.7) |
| 140 | Isofenphos | N.D | N.D | N.D | N.D | N.D | N.D | 86.35(7.3) | 85.64(8.6) | 82.74(5.2) | 101.15(9.0) | 97.18(7.3) | 99.68(4.4) |
| 141 | Crufomate | 90.85(5.4) | 92.54(3.7) | 96.54(6.3) | 84.15(3.2) | 88.14(3.9) | 92.35(4.5) | 89.65(5.3) | 86.59(6.5) | 84.75(3.6) | 96.38(8.2) | 94.28(7.2) | 92.35(2.5) |
| 142 | Chlorfenvinphos | 74.57(8.9) | 78.59(6.8) | 83.19(6.4) | 76.58(4.8) | 80.57(3.6) | 84.24(5.9) | 74.55(8.6) | 78.59(6.9) | 73.25(3.2) | 92.76(6.5) | 90.58(10.3) | 89.36(5.9) |
| 143 | cis-Chlordane (α) | 76.54(5.9) | 78.69(8.9) | 82.64(5.6) | 83.54(6.1) | 86.49(8.2) | 90.56(6.9) | 82.45(5.5) | 80.59(7.4) | 85.73(8.6) | 79.63(5.5) | 82.67(8.3) | 76.25(9.3) |
| 144 | Tolylfluanid | 76.49(5.1) | 82.16(6.3) | 85.46(8.6) | 82.65(6.4) | 85.64(7.8) | 88.64(5.4) | N.D | N.D | 85.46(14.7) | 94.22(8.2) | 91.26(6.5) | 88.64(2.5) |
| 145 | 4,4'-DDE | 82.64(5.6) | 86.79(7.4) | 92.45(6.5) | 83.54(6.2) | 85.67(6.8) | 88.96(8.7) | 74.35(15.4) | 76.79(12.5) | 78.36(8.6) | 79.35(6.5) | 80.67(4.1) | 76.55(6.4) |
| 146 | Butachlor | 85.47(4.7) | 87.64(2.9) | 83.69(9.5) | 93.25(4.8) | 96.58(8.4) | 94.57(6.1) | 92.75(6.4) | 90.47(5.4) | 88.46(7.2) | 93.25(6.4) | 96.58(8.7) | 90.47(5.1) |
| 147 | Chlozolinate | 72.58(3.4) | 77.68(7.2) | 81.49(5.5) | 84.15(6.2) | 85.78(4.2) | 88.91(6.4) | 87.65(9.3) | 85.46(6.2) | 84.26(3.3) | 91.58(7.5) | 88.44(5.3) | 85.56(4.0) |
| 148 | Crotoxyphos | 81.47(5.2) | 85.47(3.6) | 88.61(5.8) | 74.85(2.7) | 80.16(6.8) | 86.14(8.5) | 94.15(8.7) | 91.64(6.6) | 88.61(2.5) | N.D | N.D | 71.46(15.5) |
| 149 | Iodofenphos | 93.65(6.4) | 96.47(8.5) | 97.15(8.6) | 72.15(6.2) | 75.68(5.8) | 78.68(8.2) | 85.35(4.3) | 83.56(6.5) | 81.47(9.5) | 88.47(8.0) | 90.28(6.3) | 85.47(4.6) |
| 150 | Z-Tetrachlorvinphos | 91.58(4.8) | 93.58(6.7) | 95.49(6.7) | 81.64(6.4) | 85.75(6.8) | 90.58(11.2) | 96.45(7.4) | 95.31(5.5) | 91.46(4.4) | 75.46(23.6) | 77.46(18.6) | 74.25(15.4) |
| 151 | Chlorbromuron | 82.91(3.6) | 85.74(6.2) | 89.64(6.9) | 74.58(5.1) | 78.69(6.7) | 81.59(6.3) | 88.65(10.3) | 85.74(9.2) | 83.26(7.3) | 97.48(6.4) | 95.16(6.3) | 92.14(8.2) |
| 152 | Profenofos | 64.58(6.3) | 72.58(7.3) | 75.68(4.9) | 77.65(2.6) | 82.65(5.6) | 88.65(5.3) | 90.25(8.0) | 88.79(5.4) | 86.59(3.2) | 88.26(9.7) | 86.49(4.6) | 85.35(5.2) |
| 153 | Flurochloridone | 91.58(4.2) | 95.84(6.2) | 96.54(3.5) | 76.85(8.5) | 79.68(9.3) | 82.94(6.3) | 103.5(8.3) | 101.44(6.2) | 98.25(4.2) | 98.37(7.4) | 95.38(6.5) | 93.67(7.4) |
| 154 | Buprofezin | 92.58(3.6) | 90.58(6.6) | 86.59(6.3) | 91.28(7.7) | 95.68(8.2) | 96.48(5.3) | 96.35(5.5) | 98.14(7.3) | 94.21(8.4) | 99.48(7.8) | 97.68(8.2) | 96.48(5.0) |
| 155 | 2,4'-DDD | 88.54(2.6) | 92.51(4.8) | 94.78(6.3) | 82.59(6.3) | 85.69(7.6) | 87.59(8.8) | N.D | N.D | 54.26(25.4) | N.D | N.D | 74.59(18.3) |
| 156 | Endrin | 87.25(2.6) | 88.96(3.8) | 85.47(6.9) | 93.58(2.2) | 91.64(6.3) | 88.69(4.6) | 85.45(9.2) | 88.96(7.0) | 85.47(4.3) | 93.58(5.2) | 91.64(6.8) | 88.69(4.6) |
| 157 | Hexaconazole | 85.64(5.4) | 88.96(6.6) | 86.55(7.8) | 77.85(6.2) | 80.65(7.2) | 84.96(4.4) | 60.15(15.6) | 68.78(16.5) | 72.32(7.9) | 89.37(9.1) | 87.46(5.3) | 84.96(6.4) |
| 158 | Chlorfenson | N.D | N.D | N.D | N.D | N.D | N.D | 93.65(8.1) | 95.48(7.1) | 89.37(4.3) | 82.59(6.3) | 80.65(7.5) | 78.26(4.7) |
| 159 | 2,4'-DDT | 91.58(5.2) | 88.64(3.3) | 85.41(5.6) | 90.25(5.4) | 92.65(4.8) | 95.78(6.3) | 96.55(6.4) | 98.64(3.4) | 92.38(7.3) | 94.55(7.3) | 92.65(4.9) | 95.78(8.4) |
| 160 | Paclobutrazol | 53.69(15.4) | 58.49(10.2) | 63.68(6.8) | 84.15(3.6) | 86.94(5.8) | 88.64(7.2) | 98.25(8.0) | 96.34(10.5) | 94.26(6.8) | 106.37(13.5) | 104.23(11.3) | 99.67(8.5) |
| 161 | Methoprotryne | 102.26(5.3) | 105.62(6.7) | 104.96(9.8) | 94.52(3.6) | 92.48(4.4) | 88.49(6.5) | 101.5(7.4) | 105.62(8.4) | 97.64(6.3) | 98.25(9.0) | 96.45(6.3) | 94.75(5.4) |
| 162 | Erbon | 76.85(8.5) | 79.65(6.2) | 81.54(5.8) | 94.58(6.5) | 92.16(4.4) | 88.49(2.6) | 105.5(8.0) | 101.89(10.3) | 97.26(5.9) | 94.58(6.6) | 92.16(4.2) | 88.49(2.7) |
| 163 | Chloropropylate | 91.58(3.8) | 90.48(6.2) | 88.54(6.2) | 74.25(3.5) | 80.54(6.9) | 82.35(6.9) | 98.65(6.3) | 96.25(5.2) | 94.23(7.2) | 88.26(8.4) | 85.26(7.2) | 82.35(6.9) |
| 164 | Flamprop-methyl | 92.45(3.9) | 90.48(6.2) | 87.45(5.8) | 85.14(3.8) | 88.49(6.2) | 90.85(6.4) | 88.25(7.3) | 90.48(6.9) | 85.26(5.8) | 85.14(3.9) | 88.49(6.3) | 80.23(4.2) |
| 165 | Nitrofen | 105.26(3.9) | 103.59(4.8) | 95.48(3.6) | 96.48(2.2) | 95.18(3.6) | 91.08(6.3) | 83.65(7.7) | 80.26(5.5) | 81.22(3.3) | 78.36(13.7) | 80.26(12.0) | 75.64(6.8) |
| 166 | Oxyﬂuorfen | 90.58(2.6) | 88.49(3.8) | 85.14(6.7) | 85.15(6.4) | 78.49(10.2) | 88.49(8.1) | 90.55(7.3) | 88.49(3.9) | 85.14(6.2) | 97.64(9.2) | 95.28(7.5) | 92.38(6.1) |
| 167 | Chlorthiophos | 108.54(6.8) | 103.65(7.1) | 98.18(6.5) | 72.58(4.5) | 75.98(4.4) | 82.54(6.3) | 96.35(5.2) | 95.48(6.4) | 90.28(6.5) | 91.58(8.6) | 90.28(7.2) | 88.24(6.3) |
| 168 | β-Endosulfan | 74.18(2.6) | 78.49(6.8) | 85.25(6.5) | 83.25(1.4) | 85.49(6.9) | 88.76(6.1) | 71.45(9.3) | 75.26(8.5) | 72.39(6.3) | 77.59(11.4) | 75.46(8.3) | 74.15(5.0) |
| 169 | Flamprop-isopropyl | 82.51(6.5) | 85.49(3.1) | 88.49(6.1) | 74.26(2.9) | 78.65(3.1) | 85.63(8.2) | 88.95(10.3) | 86.49(8.2) | 88.49(6.2) | 88.29(9.3) | 86.39(7.2) | 85.63(8.2) |
| 170 | 4,4'-DDT | 94.15(6.4) | 86.49(7.2) | 90.85(4.6) | 83.49(4.1) | 85.49(6.5) | 88.46(7.2) | 88.35(15.7) | 86.49(12.4) | 84.25(7.1) | 96.38(4.3) | 94.23(6.2) | 91.27(8.1) |
| 171 | Carbophenothion | 74.58(24.8) | 79.54(11.5) | 81.59(6.8) | 91.48(3.6) | 93.65(5.9) | 95.48(6.2) | 77.35(8.3) | 79.54(11.5) | 74.16(4.6) | 91.48(6.4) | 93.65(4.2) | 87.16(6.4) |
| 172 | Benalaxyl | 84.06(6.5) | 88.41(7.2) | 90.48(6.9) | 73.25(2.5) | 75.69(4.8) | 78.59(6.3) | N.D | 74.26(13.6) | 76.39(11.5) | N.D | 108.62(12.4) | 107.49(11.5) |
| 173 | Edifenphos | 89.51(5.2) | 92.58(6.8) | 94.18(3.9) | 95.21(3.9) | 92.58(4.6) | 88.49(2.9) | 95.25(6.4) | 92.58(6.9) | 89.37(3.1) | 95.21(4.0) | 92.58(4.5) | 88.49(2.2) |
| 174 | Triazophos | 91.58(4.4) | 93.75(6.6) | 95.84(8.2) | 72.49(3.6) | 75.48(6.5) | 82.49(3.8) | 85.65(7.5) | 84.26(8.2) | 81.14(5.0) | 89.69(6.4) | 86.34(5.3) | 83.69(4.6) |
| 175 | Cyanofenphos | 84.15(3.8) | 88.49(3.4) | 90.58(5.8) | 79.41(2.3) | 83.54(6.2) | 86.35(6.5) | 94.25(5.4) | 92.16(4.3) | 89.67(6.4) | 95.66(7.2) | 92.46(7.1) | 90.15(4.1) |
| 176 | Chlorbenside sulfone | 73.49(2.6) | 76.48(5.5) | 79.48(7.9) | 70.48(2.6) | 73.89(3.4) | 78.59(6.4) | N.D | N.D | N.D | N.D | N.D | N.D |
| 177 | Endosulfan Sulfate | 88.49(2.9) | 85.49(6.9) | 81.08(6.5) | 84.51(3.2) | 88.49(6.5) | 91.58(7.2) | 89.35(6.3) | 87.49(5.5) | 84.26(8.0) | 91.46(7.4) | 88.49(6.1) | 86.59(8.2) |
| 178 | Bromopropylate | N.D | N.D | N.D | 89.25(3.4) | 92.58(6.4) | 95.12(3.9) | N.D | N.D | N.D | 99.87(7.6) | 97.16(6.5) | 95.12(3.6) |
| 179 | Benzoylprop-ethyl | 71.54(3.3) | 75.89(4.6) | 78.93(7.4) | 81.52(4.5) | 83.59(6.4) | 88.75(7.7) | 97.45(6.4) | 95.89(4.7) | 93.68(6.4) | 82.59(7.0) | 83.59(6.4) | 79.38(4.1) |
| 180 | Fenpropathrin | 90.52(5.2) | 92.84(6.2) | 95.18(7.5) | 77.59(4.2) | 79.38(6.4) | 82.59(6.8) | 90.55(8.3) | 88.49(6.3) | 85.26(4.2) | 99.48(5.5) | 97.16(5.5) | 95.63(6.5) |
| 181 | Leptophos | 84.18(3.2) | 86.15(6.5) | 88.19(3.8) | 89.25(5.2) | 91.57(6.6) | 93.84(7.5) | 99.35(6.5) | 97.16(4.3) | 95.33(3.8) | 98.26(6.4) | 95.57(5.4) | 93.84(7.5) |
| 182 | EPN | 82.54(3.6) | 84.98(5.5) | 87.49(3.8) | 73.58(6.2) | 75.89(7.9) | 77.49(9.5) | 78.25(6.4) | 76.39(4.2) | 74.33(6.4) | 83.49(4.7) | 81.73(5.3) | 77.49(9.9) |
| 183 | Hexazinone | 74.65(3.1) | 78.95(6.2) | 80.65(6.4) | 85.41(2.8) | 87.95(3.9) | 91.58(6.5) | 92.55(7.4) | 88.95(6.4) | 85.39(6.9) | 90.14(7.2) | 87.95(4.0) | 85.34(5.2) |
| 184 | Phosalone | 82.54(3.2) | 85.19(5.1) | 88.59(7.1) | 90.52(3.5) | 93.84(4.8) | 99.84(6.5) | 89.65(6.4) | 85.19(4.2) | 86.34(5.5) | 82.49(12.5) | 85.19(5.5) | 79.36(8.6) |
| 185 | Azinphos-methyl | 86.21(1.2) | 83.17(9.5) | 79.61(15.3) | 69.54(5.5) | 73.59(7.2) | 75.49(5.9) | 98.35(11.4) | 95.37(8.3) | 91.48(7.3) | 96.34(7.2) | 93.59(5.4) | 90.25(6.1) |
| 186 | Fenarimol | 90.25(5.3) | 92.58(8.4) | 88.52(3.9) | 86.54(6.5) | 89.65(8.2) | 93.64(10.2) | 96.35(8.2) | 94.12(4.6) | 91.46(6.4) | 95.48(7.2) | 96.34(6.4) | 93.64(10.4) |
| 187 | Azinphos-ethyl | 84.59(3.9) | 88.59(6.5) | 89.19(6.5) | N.D | N.D | N.D | N.D | 78.36(13.7) | 79.64(11.1) | 97.64(5.4) | 99.65(6.3) | 94.28(10.1) |
| 188 | Prochloraz | 80.65(3.3) | 85.49(6.6) | 88.49(7.8) | 90.58(3.6) | 92.87(5.5) | 94.87(6.2) | 100.5(6.3) | 97.46(5.4) | 94.16(4.1) | 96.38(5.5) | 94.28(8.3) | 91.87(6.1) |
| 189 | Coumaphos | 76.59(6.9) | 80.29(7.5) | 83.59(11.9) | 105.69(9.8) | 98.47(8.2) | 94.87(6.5) | 105.5(6.9) | 102.38(4.2) | 99.15(5.2) | 86.94(8.2) | 88.19(7.2) | 82.56(4.1) |
| 190 | Cyfluthrin | N.D | N.D | N.D | N.D | N.D | N.D | N.D | N.D | 72.39(13.7) | N.D | N.D | 79.36(16.0) |
| 191 | tau-Fluvalinate | N.D | 68.92(3.5) | 70.28(10.8) | 94.58(8.4) | 98.48(9.6) | 97.18(8.9) | 88.25(7.2) | 85.39(8.3) | 81.49(4.3) | 83.64(8.9) | 81.87(4.7) | 78.36(2.5) |
| 192 | Dichlorvos | 86.35(4.6) | 88.96(7.8) | 91.48(7.5) | 87.25(4.6) | 90.15(6.3) | 91.48(8.6) | 93.55(6.4) | 91.28(5.2) | 87.34(2.4) | 72.46(13.7) | 75.62(8.6) | 77.22(4.4) |
| 193 | Biphenyl | 84.16(3.2) | 88.76(5.7) | 90.36(8.3) | 92.64(5.3) | 94.86(6.8) | 96.35(8.4) | 92.25(5.8) | 90.58(6.3) | 87.16(4.2) | 97.26(5.4) | 94.26(4.1) | 91.28(6.4) |
| 194 | Vernolate | 82.95(6.3) | 85.49(7.5) | 88.96(8.7) | 77.19(5.2) | 82.49(6.5) | 85.39(7.8) | 82.95(8.3) | 80.47(6.4) | 78.96(5.2) | 97.32(6.4) | 94.28(4.8) | 91.27(6.5) |
| 195 | 3,5-Dichloroaniline | N.D | N.D | N.D | N.D | 68.25(7.2) | 70.18(6.3) | N.D | 72.64(16.4) | 73.58(14.6) | N.D | 80.25(13.7) | 82.67(15.5) |
| 196 | Molinate | 71.36(10.3) | 75.18(8.2) | 77.91(6.3) | 87.25(4.6) | 90.15(6.3) | 91.48(8.6) | 96.35(6.4) | 94.27(5.7) | 91.38(3.3) | 96.25(8.3) | 94.18(6.4) | 91.48(5.5) |
| 197 | E-Methacrifos | 62.18(5.4) | 65.48(10.6) | 70.28(8.2) | 82.49(5.5) | 85.94(6.8) | 88.19(12.8) | 85.45(4.1) | 83.69(3.3) | 79.64(2.2) | 89.57(8.7) | 87.94(6.4) | 85.17(4.6) |
| 198 | o-Phenylphenol | 87.49(6.2) | 88.96(7.8) | 91.48(7.5) | 74.18(5.2) | 75.69(6.8) | 78.49(10.5) | 88.65(10.6) | 85.69(7.9) | 81.48(5.3) | 96.58(7.2) | 94.23(5.2) | 91.62(4.3) |
| 199 | cis-1,2,3,6-Tetrahydrophthalimide | N.D | N.D | N.D | N.D | N.D | N.D | 83.65(7.3) | 85.99(5.3) | 79.62(2.4) | 86.37(7.7) | 84.19(4.3) | 81.96(3.5) |
| 200 | Fenobucarb | 71.54(3.5) | 73.59(6.4) | 76.54(8.3) | 81.94(6.5) | 85.32(4.8) | 84.19(11.3) | 102.5(8.3) | 99.64(6.5) | 96.54(8.9) | 95.68(6.4) | 92.68(4.2) | 89.67(2.2) |
| 201 | Benﬂuralin | 91.48(3.6) | 95.47(7.2) | 96.34(1.5) | 93.52(7.5) | 95.21(8.4) | 98.63(4.7) | 97.25(6.4) | 95.47(5.3) | 96.34(2.2) | 104.87(8.5) | 101.59(7.2) | 98.63(4.7) |
| 202 | Hexaflumuron | 73.54(8.6) | 75.64(9.2) | 78.65(6.7) | 67.25(4.3) | 69.32(10.5) | 71.25(8.9) | 98.25(7.7) | 96.54(3.3) | 94.12(3.0) | 97.62(8.7) | 94.27(6.3) | 90.18(5.2) |
| 203 | Prometon | 74.12(3.6) | 76.48(6.) | 78.54(6.4) | 90.25(6.4) | 91.85(7.2) | 93.58(9.8) | 95.15(6.4) | 93.15(8.5) | 89.18(6.5) | 90.25(6.8) | 91.85(7.3) | 86.32(4.3) |
| 204 | Triallate | 71.25(15.6) | 75.69(8.4) | 79.65(6.8) | 81.35(6.2) | 84.67(8.2) | 88.36(9.7) | 89.65(8.6) | 87.54(6.4) | 84.59(6.9) | 93.68(10.3) | 94.67(8.7) | 90.21(6.4) |
| 205 | Pyrimethanil | 91.58(5.4) | 95.24(7.6) | 99.68(9.4) | 97.25(3.4) | 99.54(6.3) | 103.5(5.8) | 96.35(7.3) | 95.24(6.3) | 92.68(5.2) | 97.25(9.3) | 99.54(8.5) | 94.18(6.4) |
| 206 | γ-HCH | 102.5(4.6) | 98.54(5.8) | 95.42(6.3) | 84.25(3.1) | 88.34(6.7) | 86.24(7.2) | 97.45(8.2) | 98.54(5.2) | 95.42(7.0) | 88.92(7.3) | 85.46(7.0) | 83.24(4.2) |
| 207 | Disulfoton | 95.24(3.6) | 98.64(5.6) | 100.26(6.3) | 83.25(1.6) | 85.69(7.4) | 86.97(8.3) | 86.95(7.2) | 88.63(5.3) | 82.57(3.1) | 74.62(23.6) | 72.69(22.2) | 77.63(17.3) |
| 208 | Atrazine | 76.35(4.5) | 78.65(6.8) | 80.25(6.9) | 72.59(3.8) | 75.69(4.6) | 78.49(11.8) | 78.35(5.3) | 80.23(6.4) | 76.39(4.6) | 96.38(11.3) | 94.25(9.3) | 91.25(7.3) |
| 209 | Heptachlor | N.D | N.D | N.D | N.D | N.D | N.D | 97.65(8.3) | 95.24(6.3) | 91.46(2.6) | 100.32(7.3) | 97.26(6.3) | 95.22(4.1) |
| 210 | Iprobenfos | 80.25(3.6) | 85.12(5.2) | 88.19(7.2) | 91.54(4.2) | 93.54(6.5) | 96.48(7.6) | 94.25(7.3) | 91.38(6.3) | 88.19(4.2) | 98.26(6.4) | 96.47(5.6) | 94.28(3.8) |
| 211 | Isazofos | 90.62(4.3) | 92.51(5.2) | 96.25(7.8) | 86.25(5.2) | 88.25(7.4) | 91.62(14.2) | 104.5(8.6) | 101.96(7.3) | 98.25(5.3) | 96.38(6.5) | 93.28(4.2) | 91.62(2.2) |
| 212 | Plifenate | 72.56(5.2) | 75.24(8.2) | 77.16(8.2) | 73.25(4.2) | 76.59(5.8) | 80.59(12.8) | N.D | 62.35(22.5) | 67.49(18.6) | N.D | 70.25(15.3) | 73.68(13.0) |
| 213 | Fenpropimorph | 69.25(15.2) | 71.58(4.9) | 75.28(6.5) | 87.54(6.5) | 89.65(7.2) | 90.21(8.6) | 93.65(9.4) | 91.24(7.5) | 88.37(6.5) | 87.54(8.3) | 89.65(7.2) | 85.69(5.1) |
| 214 | Transfluthrin | 75.21(6.3) | 78.65(6.5) | 80.25(8.5) | 77.85(5.2) | 79.63(5.2) | 82.54(6.9) | 78.65(6.4) | 76.35(4.2) | 77.63(6.3) | 95.28(8.3) | 94.25(6.2) | 90.28(3.5) |
| 215 | Fluchloralin | 91.54(6.6) | 94.87(7.6) | 95.23(4.8) | 74.36(4.2) | 76.38(5.3) | 79.25(6.8) | 95.65(8.1) | 94.87(9.4) | 90.58(5.6) | 88.39(5.6) | 89.67(7.7) | 85.34(4.1) |
| 216 | Tolclofos-methyl | 91.54(5.5) | 95.34(6.4) | 97.48(6.8) | 92.58(4.8) | 93.57(6.8) | 98.57(8.6) | 93.65(8.5) | 90.78(5.3) | 88.25(3.6) | 97.58(5.6) | 96.37(6.0) | 93.67(4.3) |
| 217 | Propisochlor | 86.35(4.5) | 88.69(5.9) | 90.25(7.8) | 90.54(4.5) | 93.64(5.2) | 95.84(9.7) | 95.35(9.4) | 92.46(7.3) | 88.35(5.5) | 59.38(25.4) | 65.79(21.5) | 72.36(19.3) |
| 218 | Ametryn | 91.84(6.3) | 95.48(5.2) | 94.12(6.3) | 88.25(5.5) | 91.84(6.8) | 93.48(7.6) | 86.35(8.3) | 84.57(6.4) | 81.25(4.2) | 88.25(9.6) | 91.84(4.7) | 85.63(2.6) |
| 219 | Simetryn | 81.48(1.9) | 85.64(7.2) | 88.94(8.3) | 81.95(7.6) | 85.41(8.2) | 88.49(9.5) | 89.65(8.3) | 87.36(6.5) | 85.32(6.2) | 83.52(8.7) | 85.41(8.4) | 80.25(4.4) |
| 220 | Metobromuron | 82.15(6.3) | 83.84(5.2) | 84.95(11.6) | 67.52(15.3) | 70.25(5.4) | 72.35(6.4) | 98.55(10.4) | 95.38(7.2) | 94.38(6.4) | 96.57(6.5) | 94.38(5.4) | 90.28(3.2) |
| 221 | Metribuzin | 80.35(6.4) | 83.49(7.6) | 85.96(7.9) | 66.59(9.8) | 71.58(8.4) | 73.59(6.8) | 83.65(7.3) | 85.64(8.4) | 80.29(4.8) | 88.69(6.4) | 86.25(4.4) | 85.49(1.4) |
| 222 | Dimethipin | 84.58(6.3) | 85.96(7.2) | 87.49(10.6) | 91.48(5.2) | 93.58(6.7) | 96.87(8.3) | 97.85(8.0) | 95.96(5.7) | 91.46(4.2) | 96.47(3.5) | 93.58(6.0) | 90.35(5.4) |
| 223 | ε-HCH | 81.75(8.5) | 83.74(6.8) | 85.97(9.4) | 97.56(3.9) | 98.89(6.5) | 101.28(3.9) | N.D | 68.49(22.2) | 71.24(19.0) | N.D | 73.12(8.8) | 72.19(6.0) |
| 224 | Dipropetryn | 91.58(6.7) | 95.86(7.8) | 97.46(5.2) | 76.35(4.5) | 78.96(6.4) | 80.25(4.1) | 96.25(8.3) | 95.86(4.6) | 93.47(2.4) | 95.68(6.5) | 92.37(4.1) | 88.97(2.5) |
| 225 | Formothion | 94.85(4.6) | 92.76(2.6) | 88.49(9.4) | 73.49(5.2) | 76.59(8.3) | 78.52(8.9) | 80.55(6.4) | 78.69(9.0) | 75.35(5.5) | 74.69(13.7) | 76.98(14.6) | 75.36(8.9) |
| 226 | Diethofencarb | 64.86(15.6) | 68.74(9.5) | 73.25(6.4) | 74.12(3.2) | 76.59(8.2) | 80.15(4.5) | 85.35(15.7) | 83.57(11.5) | 78.26(8.3) | 80.98(9.2) | 78.36(4.6) | 76.39(3.2) |
| 227 | Dimepiperate | 88.35(4.3) | 89.64(7.2) | 92.46(11.6) | 85.23(4.6) | 88.64(8.3) | 90.63(9.1) | 91.25(6.4) | 88.36(6.5) | 86.39(5.5) | 98.65(6.4) | 96.58(4.8) | 92.38(8.4) |
| 228 | Bioallethrin | 86.32(4.6) | 89.76(8.4) | 92.48(7.6) | N.D | 69.34(10.3) | 71.59(11.6) | 93.55(5.6) | 91.78(6.9) | 88.39(8.3) | 96.38(9.5) | 94.12(7.4) | 91.23(5.6) |
| 229 | 2,4'-DDE | 71.54(5.2) | 73.58(6.9) | 78.65(5.4) | 86.54(3.2) | 88.97(5.4) | 91.87(7.2) | 77.55(6.4) | 78.69(8.4) | 75.46(3.5) | 79.68(8.5) | 81.23(6.5) | 77.25(5.5) |
| 230 | Fenson | 91.25(3.8) | 92.48(3.6) | 95.78(6.9) | 93.48(5.8) | 96.79(4.8) | 95.38(6.4) | 94.65(7.4) | 92.48(6.3) | 88.49(5.4) | 90.28(7.7) | 88.25(7.2) | 85.32(4.7) |
| 231 | Diphenamid | 55.36(15.7) | 64.59(10.6) | 65.23(8.9) | 87.54(3.4) | 89.36(4.5) | 92.54(6.5) | 93.65(8.3) | 96.37(7.2) | 90.21(8.4) | 79.46(8.0) | 77.59(6.5) | 75.23(4.2) |
| 232 | Chlorthion | 77.52(6.5) | 79.65(8.5) | 81.59(11.9) | 72.59(4.8) | 75.96(5.8) | 77.69(6.4) | 75.65(6.4) | 74.28(4.2) | 78.63(6.5) | 83.56(9.3) | 80.41(7.2) | 77.69(6.3) |
| 233 | Prallethrin | N.D | N.D | N.D | N.D | N.D | N.D | 87.95(8.5) | 86.23(5.5) | 84.51(4.0) | 94.85(7.4) | 91.46(6.4) | 88.37(6.4) |
| 234 | Penconazole | 75.31(4.7) | 77.34(6.9) | 80.18(7.4) | 92.34(4.8) | 95.48(6.7) | 94.67(8.1) | 85.75(7.5) | 87.34(6.5) | 82.49(5.7) | 99.15(4.3) | 97.48(5.4) | 94.67(4.6) |
| 235 | Mecarbam | 65.19(20.6) | 68.15(4.9) | 72.36(13.4) | 73.25(5.5) | 76.31(6.8) | 75.28(4.9) | 77.65(13.3) | 79.63(11.9) | 75.42(8.1) | 87.54(13.3) | 85.74(8.7) | 83.65(6.0) |
| 236 | Tetraconazole | 76.25(5.1) | 78.69(6.4) | 80.31(6.9) | 87.64(3.4) | 88.19(5.7) | 90.74(10.5) | 83.55(7.0) | 80.14(5.4) | 78.96(3.5) | 89.64(8.6) | 88.19(8.0) | 85.41(6.3) |
| 237 | Propaphos | 84.75(11.3) | 86.96(7.4) | 88.39(10.5) | 91.78(5.4) | 93.61(6.4) | 95.61(8.4) | 95.25(6.6) | 91.22(5.4) | 88.39(2.5) | 103.54(8.6) | 99.61(6.5) | 97.25(4.9) |
| 238 | Flumetralin | 72.58(4.8) | 75.63(4.9) | 78.63(4.9) | 84.63(7.2) | 88.61(8.3) | 90.19(7.3) | 98.55(6.4) | 96.58(5.4) | 95.18(5.0) | N.D | 75.69(12.6) | 73.64(8.2) |
| 239 | Triadimenol | 85.15(7.1) | 87.16(8.7) | 90.14(6.4) | 93.49(11.2) | 95.36(4.8) | 98.31(6.8) | 89.65(7.4) | 87.16(8.1) | 85.19(3.3) | 85.67(11.3) | 82.25(8.2) | 80.47(6.3) |
| 240 | Pretilachlor | 88.26(4.8) | 91.67(6.4) | 95.46(8.7) | 91.46(8.4) | 93.47(6.4) | 94.19(7.2) | 96.35(7.1) | 94.58(6.3) | 88.67(4.3) | 99.68(6.4) | 95.82(5.2) | 94.19(4.2) |
| 241 | Kresoxim-methyl | 95.48(7.2) | 94.16(5.5) | 98.49(6.7) | 83.16(6.4) | 87.49(6.7) | 88.94(8.2) | 98.35(6.6) | 97.16(6.0) | 94.58(4.6) | 96.87(7.9) | 94.26(6.3) | 95.68(4.4) |
| 242 | Fluazifop-butyl | 81.79(6.1) | 85.47(3.6) | 88.19(6.4) | 93.87(7.2) | 90.78(4.4) | 84.14(10.6) | 89.65(10.4) | 87.46(8.8) | 85.47(4.4) | 98.65(6.5) | 96.57(5.6) | 94.38(3.2) |
| 243 | Chlorfluazuron | 80.9(7.4) | 82.49(8.4) | 85.49(6.2) | 92.16(4.9) | 94.85(6.7) | 96.43(7.6) | 87.35(9.3) | 85.46(6.3) | 82.45(3.5) | 92.16(6.5) | 90.25(4.3) | 88.45(3.3) |
| 244 | Chlorobenzilate | N.D | N.D | N.D | N.D | N.D | N.D | 95.65(7.3) | 91.47(5.4) | 88.39(4.3) | 98.67(8.3) | 96.35(7.2) | 95.23(5.7) |
| 245 | Uniconazole | N.D | N.D | N.D | N.D | N.D | N.D | 94.25(6.9) | 92.49(8.5) | 89.47(4.8) | 96.35(8.7) | 94.85(6.6) | 90.25(3.3) |
| 246 | Flusilazole | 81.49(3.7) | 85.49(6.7) | 87.15(8.7) | 93.48(6.9) | 90.87(8.6) | 98.48(10.3) | 88.35(10.3) | 87.49(7.7) | 85.15(6.5) | 85.23(5.1) | 83.69(4.3) | 80.35(2.5) |
| 247 | Fluorodifen | N.D | N.D | N.D | N.D | N.D | N.D | N.D | 98.36(11.2) | 99.25(10.4) | N.D | 82.57(8.7) | 84.55(9.4) |
| 248 | Diniconazole | 87.25(4.9) | 89.62(5.5) | 92.78(6.4) | 87.14(5.1) | 90.87(4.6) | 92.17(8.7) | 95.35(8.2) | 93.68(7.2) | 89.32(4.3) | 95.38(8.1) | 92.46(5.2) | 89.67(8.9) |
| 249 | Piperonyl butoxide | 82.49(6.4) | 85.49(5.4) | 87.15(6.7) | 87.64(5.1) | 89.56(11.4) | 93.47(8.9) | 89.65(5.3) | 87.62(4.2) | 85.67(6.4) | 96.57(6.5) | 94.16(5.3) | 91.47(3.5) |
| 250 | Propargite | 92.84(4.7) | 94.86(8.3) | 97.18(6.5) | 93.48(7.2) | 95.86(8.3) | 90.37(9.9) | 88.65(9.8) | 86.34(7.5) | 85.23(5.1) | 98.56(8.3) | 96.37(5.1) | 94.25(3.3) |
| 251 | Mepronil | 86.4(7.2) | 88.96(6.7) | 92.49(6.1) | 94.25(1.8) | 97.65(4.8) | 100.3(7.3) | 97.55(8.5) | 95.36(4.6) | 92.49(6.3) | 98.25(8.7) | 97.65(5.7) | 95.68(3.5) |
| 252 | Dimefuron | 84.52(5.6) | 88.76(7.4) | 90.35(7.6) | 82.49(7.2) | 85.21(6.4) | 88.63(7.6) | 103.5(8.6) | 101.24(6.3) | 98.25(4.6) | 95.63(12.4) | 93.47(9.5) | 91.28(8.4) |
| 253 | Diﬂufenican | 90.37(5.8) | 93.48(6.4) | 95.78(7.6) | 86.59(5.4) | 88.49(6.7) | 90.28(3.7) | 98.25(6.5) | 96.34(5.9) | 94.15(4.3) | 96.35(9.2) | 95.45(7.1) | 92.38(5.3) |
| 254 | Fenazaquin | 82.54(6.5) | 86.24(4.8) | 84.19(8.5) | 87.59(4.6) | 90.25(5.7) | 92.48(6.8) | 105.5(6.5) | 101.47(4.3) | 98.63(2.5) | 96.35(7.3) | 93.68(4.7) | 92.48(2.9) |
| 255 | Phenothrin | 76.25(5.4) | 78.64(8.5) | 80.49(9.4) | 90.48(6.4) | 92.84(7.6) | 95.48(8.7) | 77.65(8.8) | 78.64(6.3) | 75.96(4.2) | 94.63(7.6) | 92.84(6.9) | 89.25(3.1) |
| 256 | Fludioxonil | N.D | N.D | N.D | N.D | N.D | N.D | N.D | N.D | N.D | N.D | N.D | N.D |
| 257 | Fenoxycarb | 84.35(9.5) | 86.25(6.2) | 90.86(7.6) | 91.68(4.6) | 93.18(5.7) | 95.38(7.8) | 88.55(6.4) | 86.25(5.5) | 85.26(3.1) | 99.68(4.6) | 97.18(5.7) | 95.38(4.2) |
| 258 | Sethoxydim | 68.14(4.2) | 70.25(5.6) | 73.58(9.3) | N.D | 75.26(6.4) | 80.25(15.6) | 88.55(3.6) | 86.25(5.5) | 84.12(6.4) | 85.47(6.3) | 83.57(5.5) | 82.41(7.4) |
| 259 | Anilofos | 71.85(6.4) | 73.49(6.8) | 76.49(7.5) | 85.24(6.3) | 88.47(7.8) | 91.58(8.7) | 95.65(6.3) | 93.49(5.1) | 89.25(6.2) | 78.95(12.7) | 76.45(9.4) | 85.64(3.3) |
| 260 | Acrinathrin | N.D | N.D | N.D | 91.87(8.4) | 93.48(9.5) | 95.87(6.5) | 75.85(7.2) | 73.49(6.3) | 76.49(2.2) | 93.25(7.5) | 91.28(6.5) | 87.49(5.4) |
| 261 | λ-Cyhalothrin | N.D | N.D | N.D | N.D | N.D | N.D | N.D | 80.26(12.4) | 78.35(15.6) | N.D | 75.42(9.7) | 74.15(8.6) |
| 262 | Mefenacet | 87.56(10.3) | 89.25(7.4) | 91.54(6.3) | 92.48(7.1) | 95.48(6.2) | 98.17(6.3) | 97.55(7.0) | 95.63(4.6) | 91.54(6.4) | 90.48(7.6) | 88.32(6.8) | 85.34(4.4) |
| 263 | Permethrin | 91.25(7.2) | 93.48(8.3) | 95.19(9.3) | 104.2(5.2) | 106.3(5.6) | 108.24(7.9) | 97.55(7.3) | 95.23(6.3) | 90.19(3.6) | 99.35(6.4) | 96.78(5.7) | 93.25(4.6) |
| 264 | Pyridaben | 75.36(4.2) | 77.68(6.4) | 79.48(7.4) | 73.26(6.9) | 77.49(8.2) | 74.12(11.3) | 88.35(7.0) | 86.33(5.5) | 82.57(6.4) | 98.25(7.3) | 96.38(5.4) | 94.26(3.1) |
| 265 | Fluoroglycofen-ethyl | 71.85(8.2) | 73.49(7.9) | 80.23(6.9) | 75.24(3.5) | 78.47(5.4) | 81.58(6.4) | 85.65(6.6) | 81.35(8.6) | 78.65(3.5) | 79.68(11.5) | 78.47(9.4) | 75.32(7.5) |
| 266 | Bitertanol | 79.35(4.1) | 83.49(7.1) | 86.49(10.2) | 84.26(5.1) | 86.39(8.2) | 91.58(7.5) | 95.25(8.1) | 93.49(6.3) | 90.21(2.1) | 97.45(7.5) | 95.28(6.6) | 93.48(2.6) |
| 267 | Etofenprox | 84.35(5.2) | 86.34(7.1) | 90.25(8.3) | 93.48(5.4) | 95.24(10.8) | 96.24(7.6) | 89.35(9.4) | 86.34(7.5) | 85.47(6.4) | 96.25(8.0) | 94.21(4.9) | 91.24(3.7) |
| 268 | Cycloxydim | 76.53(5.4) | 77.38(4.9) | 80.12(5.9) | 73.24(4.6) | 75.68(6.9) | 77.19(5.8) | 75.95(8.1) | 77.38(6.4) | 80.12(3.3) | 93.24(7.7) | 91.48(6.6) | 88.35(4.4) |
| 269 | α-Cypermethrin | 80.25(10.3) | 82.49(6.9) | 85.37(4.9) | 86.37(4.2) | 88.47(8.4) | 92.58(5.9) | 75.25(16.4) | 72.12(13.7) | 78.63(9.6) | 93.58(5.7) | 95.24(4.4) | 88.79(3.3) |
| 270 | Flucythrinate | 91.35(4.8) | 93.87(4.6) | 96.25(8.3) | 95.21(4.6) | 96.87(6.8) | 99.37(5.1) | 97.25(6.3) | 95.82(5.5) | 91.38(7.4) | 97.58(8.3) | 96.87(6.9) | 92.58(2.7) |
| 271 | Esfenvalerate | 85.32(4.9) | 87.19(6.5) | 90.36(7.4) | 82.54(6.7) | 85.69(7.3) | 87.93(4.9) | N.D | 90.25(9.0) | 92.36(6.4) | N.D | 81.69(13.7) | 82.93(11.4) |
| 272 | Difenoconazole | 93.65(8.2) | 95.49(7.6) | 98.16(4.3) | 86.31(4.9) | 88.37(5.8) | 93.64(4.7) | 96.35(7.3) | 95.49(6.2) | 91.28(5.4) | 92.58(8.3) | 89.27(6.7) | 87.15(4.8) |
| 273 | Flumioxazin | 74.21(6.4) | 76.93(8.5) | 79.24(10.6) | 89.65(4.9) | 87.24(5.8) | 85.49(6.9) | 88.55(10.2) | 86.93(8.6) | 82.57(5.4) | 89.65(12.3) | 87.24(8.7) | 85.49(5.2) |
| 274 | Flumiclorac-pentyl | 71.85(5.9) | 73.49(8.4) | 76.49(13.6) | 89.63(7.4) | 94.38(6.4) | 92.18(14.6) | 97.55(9.4) | 95.24(7.3) | 93.15(4.2) | 96.42(8.3) | 94.38(4.2) | 95.28(3.7) |
| 275 | Dimefox | 81.64(6.5) | 83.76(4.4) | 86.92(7.2) | 88.59(6.3) | 89.73(7.4) | 92.46(8.1) | 94.65(9.4) | 92.48(4.7) | 90.25(4.1) | 95.68(8.3) | 91.48(6.3) | 88.94(7.3) |
| 276 | Disulfoton-sulfoxide | 74.51(12.4) | 76.94(7.4) | 78.53(6.4) | 86.15(5.2) | 88.34(10.3) | 89.76(8.9) | 89.35(9.3) | 88.34(7.2) | 86.31(4.7) | 95.48(8.5) | 92.37(7.2) | 89.76(8.9) |
| 277 | Pentachlorobenzene | 83.15(4.1) | 85.61(5.2) | 88.47(6.4) | 90.16(3.4) | 93.49(7.2) | 88.37(7.4) | 96.35(7.2) | 94.28(5.3) | 91.47(6.5) | 98.16(5.5) | 95.34(6.2) | 92.63(2.7) |
| 278 | Triisobutyl phosphate | 72.54(3.4) | 75.26(5.4) | 77.68(7.8) | 76.54(4.6) | 78.64(6.7) | 80.16(7.8) | 86.95(6.5) | 88.48(7.9) | 84.26(4.3) | 97.46(8.1) | 95.28(7.2) | 93.47(4.2) |
| 279 | Crimidine | 83.49(5.4) | 85.62(6.7) | 88.24(7.4) | 81.85(3.5) | 85.48(6.4) | 88.34(6.8) | 90.45(8.2) | 87.46(6.4) | 85.46(4.4) | 87.49(8.2) | 85.14(6.7) | 83.47(3.0) |
| 280 | BDMC-1 | 91.58(6.4) | 93.48(7.4) | 95.12(3.5) | 93.64(5.1) | 95.61(7.3) | 98.16(8.2) | N.D | 72.46(15.7) | 73.46(13.5) | N.D | 78.26(11.5) | 80.57(9.4) |
| 281 | Chlorfenprop-methyl | 115.24(8.4) | 110.35(7.2) | 106.31(5.6) | 83.16(5.1) | 85.64(7.6) | 88.19(6.4) | 97.45(10.4) | 95.46(8.1) | 93.47(6.5) | 92.38(7.7) | 90.28(8.7) | 88.15(5.4) |
| 282 | Thionazin | 75.46(3.6) | 77.19(6.1) | 75.18(8.2) | 81.49(8.4) | 84.59(6.2) | 86.14(7.4) | 89.35(6.4) | 86.37(7.6) | 85.18(3.0) | 95.38(7.2) | 92.68(6.5) | 89.37(4.1) |
| 283 | 2,3,5,6-tetrachloroaniline | 82.49(6.7) | 83.64(5.4) | 85.45(8.3) | 75.21(6.3) | 77.69(8.4) | 79.84(12.4) | 85.35(7.2) | 83.64(9.7) | 80.39(6.3) | 90.38(7.7) | 88.16(8.5) | 85.29(4.4) |
| 284 | Tributyl phosphate | 82.49(14.5) | 84.59(6.4) | 87.24(7.9) | 75.64(8.6) | 78.54(8.7) | 80.26(9.7) | 96.35(7.5) | 93.47(5.2) | 94.36(6.1) | 85.67(8.4) | 83.49(7.4) | 80.26(4.2) |
| 285 | 2,3,4,5-Tetrachloroanisole | 96.85(3.9) | 100.2(4.5) | 98.74(3.9) | 97.24(5.6) | 95.48(7.2) | 90.18(6.9) | 96.85(8.5) | 94.22(7.2) | 91.48(3.7) | 92.37(6.5) | 90.34(5.2) | 88.14(3.0) |
| 286 | Pentachloroanisole | 80.45(5.6) | 83.64(6.5) | 87.49(5.8) | 73.84(6.6) | 75.49(7.2) | 77.68(6.8) | 97.35(8.2) | 95.46(6.6) | 93.42(5.9) | 90.48(8.3) | 88.37(6.5) | 85.35(4.3) |
| 287 | Tebutam | 90.58(6.5) | 92.87(7.6) | 94.58(8.4) | 90.58(3.5) | 93.48(6.5) | 95.48(8.4) | 99.35(10.3) | 97.46(8.3) | 94.58(4.2) | 90.58(3.9) | 93.48(6.6) | 86.25(2.2) |
| 288 | Dioxabenzofos | 92.47(6.2) | 94.18(5.9) | 94.17(8.6) | 87.15(13.4) | 89.47(5.9) | 91.84(7.2) | 105.5(8.3) | 102.46(7.3) | 99.38(5.2) | 98.37(6.5) | 96.38(7.5) | 95.48(6.3) |
| 289 | Methabenzthiazuron | 73.48(4.5) | 76.49(6.5) | 80.15(6.3) | 81.48(10.2) | 83.49(11.7) | 86.14(6.7) | 55.65(23.6) | 65.38(19.0) | 72.59(15.6) | 86.39(9.3) | 85.49(7.4) | 82.16(5.6) |
| 290 | Simeton | 85.21(6.2) | 88.19(7.5) | 91.84(6.5) | 88.94(5.4) | 92.84(7.9) | 94.87(8.6) | 98.35(7.5) | 96.35(5.7) | 94.27(8.7) | 98.37(10.4) | 97.28(8.4) | 94.87(6.5) |
| 291 | Atratone | 81.75(9.1) | 83.59(6.8) | 88.49(10.3) | 84.76(7.2) | 88.95(6.2) | 91.54(3.8) | 83.65(15.0) | 81.47(11.6) | 78.25(8.4) | 88.96(9.3) | 86.35(7.4) | 84.52(6.4) |
| 292 | Atrazine-desisopropyl | 91.85(7.2) | 93.84(7.9) | 96.87(4.2) | 81.54(2.1) | 84.75(6.5) | 87.63(9.7) | 106.5(9.2) | 102.85(7.5) | 98.87(4.8) | 95.68(8.4) | 94.75(6.6) | 91.47(4.1) |
| 293 | Terbufos sulfone | 65.21(18.6) | 68.39(5.9) | 71.84(8.4) | 86.54(6.2) | 88.94(8.5) | 91.87(10.5) | 90.45(8.3) | 88.49(6.4) | 85.49(2.9) | 82.98(8.6) | 79.36(6.2) | 77.34(5.5) |
| 294 | Tefluthrin | 83.54(7.6) | 88.59(8.6) | 91.84(8.5) | 94.21(3.6) | 98.41(6.9) | 103.54(7.2) | N.D | N.D | 77.46(13.3) | N.D | N.D | 80.47(13.7) |
| 295 | Bromocylen | 72.84(5.6) | 76.84(8.6) | 80.17(5.9) | N.D | 64.14(6.9) | 67.59(8.3) | 94.65(8.4) | 91.67(6.3) | 88.25(4.3) | 75.69(8.4) | 78.39(7.4) | 77.48(6.4) |
| 296 | Trietazine | 81.26(4.4) | 85.63(4.9) | 88.37(5.4) | 90.16(3.4) | 94.87(8.8) | 97.65(4.8) | 83.65(11.4) | 85.63(8.3) | 79.38(4.7) | 100.25(10.3) | 98.25(8.4) | 95.65(4.6) |
| 297 | Cycluron | 89.63(4.1) | 92.45(5.4) | 93.21(7.9) | 79.63(10.2) | 83.49(6.4) | 85.49(7.4) | 93.65(8.4) | 92.45(5.8) | 89.35(8.5) | 97.28(8.2) | 95.48(6.2) | 92.48(8.0) |
| 298 | 2,6-Dichlorobenzamide | 71.49(5.2) | 75.48(5.2) | 77.49(6.9) | 88.19(4.4) | 91.48(5.8) | 93.49(11.6) | 89.35(8.2) | 87.26(6.3) | 85.98(4.3) | 107.35(9.4) | 104.25(6.5) | 99.35(4.0) |
| 299 | 2,4,4'-Trichlorobiphenyl | 87.51(6.4) | 89.34(3.4) | 93.48(7.8) | 91.48(3.4) | 93.49(5.8) | 95.12(11.6) | 90.55(8.4) | 88.72(4.3) | 86.39(6.5) | 96.38(5.5) | 93.49(4.3) | 91.28(2.5) |
| 300 | 2,4,5-Trichlorobiphenyl | 85.21(6.4) | 88.79(8.2) | 90.15(7.4) | 86.34(3.3) | 89.76(5.7) | 92.46(8.4) | 95.35(7.3) | 93.25(6.5) | 90.15(7.5) | 99.48(10.3) | 95.38(7.2) | 92.46(8.1) |
| 301 | Sebuthylazine-desethyl | 78.62(4.6) | 79.62(5.4) | 81.49(6.7) | 87.49(3.3) | 89.63(4.8) | 90.31(8.2) | 89.35(6.5) | 87.35(4.2) | 85.39(5.4) | 84.26(11.3) | 81.37(6.4) | 78.39(5.4) |
| 302 | 2,3,4,5-Tetrachloroaniline | 70.25(6.4) | 73.84(5.8) | 76.31(14.6) | 86.32(4.8) | 88.69(7.9) | 93.49(6.2) | 89.35(9.0) | 87.25(4.7) | 85.39(6.4) | 94.28(6.4) | 91.47(5.4) | 88.39(6.5) |
| 303 | musk ambrette | 83.15(6.1) | 86.54(6.4) | 88.16(5.6) | 96.12(4.7) | 97.58(3.8) | 100.21(4.9) | 98.35(7.2) | 96.34(5.2) | 92.48(3.5) | 85.46(7.3) | 82.34(8.3) | 80.23(3.6) |
| 304 | Musk xylene | 77.25(13.5) | 79.75(8.4) | 80.15(9.7) | 93.48(5.6) | 95.78(7.5) | 99.48(11.6) | 89.35(13.5) | 86.37(9.8) | 85.35(6.5) | 88.36(17.5) | 85.39(14.3) | 82.57(11.7) |
| 305 | Pentachloroaniline | 75.21(4.3) | 77.96(4.9) | 80.45(6.3) | 99.63(4.5) | 97.25(4.9) | 94.68(8.5) | 92.55(6.3) | 90.78(4.3) | 87.45(6.5) | 99.63(8.4) | 97.25(6.5) | 94.68(8.6) |
| 306 | Aziprotryne | 93.18(6.3) | 96.15(5.2) | 99.58(8.4) | 87.15(3.6) | 88.29(4.3) | 93.48(8.5) | 99.25(9.3) | 96.15(5.3) | 95.68(8.7) | 89.15(3.9) | 88.29(4.4) | 85.43(6.4) |
| 307 | Sebuthylazine | 79.18(4.2) | 83.65(5.9) | 85.49(7.7) | 88.25(4.6) | 91.25(8.5) | 94.18(11.6) | 96.35(8.3) | 95.16(4.7) | 92.58(5.9) | 93.68(11.9) | 91.25(8.4) | 89.35(4.3) |
| 308 | Isocarbamid | 75.48(4.1) | 78.49(6.7) | 81.49(8.7) | 77.19(4.8) | 79.48(6.9) | 82.49(13.5) | 102.5(7.0) | 99.65(6.8) | 97.25(8.6) | 97.58(8.3) | 95.23(6.5) | 92.38(7.1) |
| 309 | 2,2',5,5'-Tetrachlorobiphenyl | 85.21(6.7) | 88.94(8.5) | 90.65(9.5) | 90.85(4.3) | 94.87(7.5) | 96.48(6.9) | 90.25(6.4) | 88.94(7.3) | 85.35(4.3) | 95.28(6.4) | 94.87(7.6) | 90.85(3.2) |
| 310 | Musk moskene | 72.18(5.9) | 75.18(9.4) | 77.49(6.8) | 78.59(5.8) | 82.49(6.8) | 85.14(6.9) | 88.25(8.4) | 85.94(7.2) | 86.95(4.3) | 80.36(11.5) | 82.49(6.9) | 77.23(6.5) |
| 311 | Prosulfocarb | 86.23(4.2) | 88.61(6.4) | 90.48(9.2) | 90.25(4.4) | 93.48(7.1) | 95.48(6.4) | 98.25(9.4) | 95.48(7.2) | 93.48(6.4) | 97.26(5.9) | 95.48(6.4) | 91.47(4.3) |
| 312 | Dimethenamid | 77.16(3.6) | 78.14(6.4) | 81.46(7.5) | 73.14(6.4) | 75.12(8.4) | 77.64(9.4) | 90.25(7.3) | 86.39(5.5) | 84.46(5.2) | 89.36(7.1) | 86.52(8.5) | 84.52(9.8) |
| 313 | Fenchlorphos-oxon | 74.21(5.4) | 77.49(8.4) | 76.31(8.4) | 81.49(10.2) | 86.32(4.9) | 88.49(7.3) | 87.55(8.3) | 85.36(6.5) | 83.16(5.4) | 91.47(6.4) | 93.46(5.8) | 88.49(5.3) |
| 314 | Paraoxon-methyl | 80.13(4.2) | 83.49(6.4) | 88.76(4.6) | 93.18(4.1) | 95.17(7.9) | 99.14(10.8) | 94.25(6.4) | 90.58(4.2) | 88.76(6.5) | 99.38(5.4) | 97.26(4.2) | 95.38(6.5) |
| 315 | Monalide | 69.18(10.5) | 73.49(3.6) | 74.19(5.4) | 80.19(5.1) | 83.34(5.4) | 88.64(5.9) | 90.35(10.6) | 87.16(8.3) | 85.35(6.5) | 88.25(12.4) | 87.46(9.4) | 85.34(5.6) |
| 316 | Tibetene musk | 91.25(4.7) | 93.58(6.5) | 96.18(7.2) | 81.75(4.6) | 83.49(7.9) | 86.44(8.2) | 98.75(8.2) | 96.48(5.3) | 94.18(6.4) | 85.96(7.3) | 83.49(7.8) | 81.47(5.3) |
| 317 | Isobenzan | 67.15(6.3) | 70.18(10.6) | 73.14(6.5) | 78.49(3.6) | 81.49(6.5) | 84.71(7.6) | 92.55(8.4) | 89.39(6.5) | 86.39(7.2) | 93.58(6.4) | 91.46(5.3) | 88.35(8.3) |
| 318 | Octachlorostyrene | 71.54(6.9) | 75.18(4.9) | 76.19(5.9) | 94.18(2.2) | 90.58(3.6) | 88.19(6.9) | N.D | 73.59(14.3) | 74.26(11.9) | N.D | 78.63(13.6) | 76.59(12.8) |
| 319 | Pyrimitate | 94.28(5.1) | 91.58(6.4) | 88.17(10.2) | 105.28(4.1) | 101.89(6.5) | 98.18(9.4) | 74.25(10.4) | 75.66(8.3) | 77.63(5.1) | 97.15(8.2) | 95.64(7.2) | 93.76(6.5) |
| 320 | Isodrin | 70.18(4.1) | 71.49(5.9) | 74.18(4.9) | 81.24(5.4) | 83.49(6.4) | 85.49(7.8) | 89.35(6.4) | 87.34(6.0) | 84.57(2.2) | 84.59(3.7) | 83.49(6.5) | 81.22(1.8) |
| 321 | Isomethiozin | 84.16(7.2) | 86.21(8.1) | 88.18(6.9) | 90.28(4.5) | 93.48(4.3) | 95.47(3.2) | 98.25(8.3) | 96.48(5.3) | 92.48(7.3) | 98.45(9.3) | 97.15(6.4) | 95.47(4.1) |
| 322 | Trichloronate | 71.49(4.4) | 73.19(5.8) | 76.49(6.8) | 91.48(5.1) | 93.47(6.4) | 95.18(6.2) | 96.25(8.3) | 94.27(6.4) | 91.05(5.1) | 95.68(7.3) | 93.47(8.5) | 90.28(2.4) |
| 323 | Chlorthal-dimethyl | 68.15(6.7) | 70.19(8.4) | 73.14(6.1) | 84.71(4.4) | 81.49(6.5) | 87.64(5.4) | 74.15(6.9) | 73.69(8.5) | 76.98(2.5) | 84.71(4.8) | 81.49(6.9) | 79.36(2.2) |
| 324 | 4,4'-Dichlorobenzophenone | 89.63(11.4) | 92.48(7.4) | 95.48(7.1) | 77.36(4.8) | 79.64(6.6) | 82.49(8.4) | 89.65(11.9) | 87.45(7.9) | 84.35(3.9) | 92.35(8.7) | 89.76(5.5) | 87.49(4.1) |
| 325 | Nitrothal-isopropyl | 68.52(17.3) | 72.19(5.6) | 70.25(4.8) | 80.23(4.3) | 82.49(5.4) | 86.49(8.1) | 74.55(22.4) | 72.19(13.7) | 70.25(18.3) | 91.46(7.3) | 89.36(5.7) | 86.49(3.2) |
| 326 | Musk ketone | N.D | N.D | N.D | N.D | N.D | N.D | N.D | 70.34(7.0) | 73.58(4.2) | N.D | 85.69(10.5) | 82.45(7.6) |
| 327 | Rabenzazol | 55.46(18.6) | 62.49(16.4) | 65.14(20.1) | 86.21(5.3) | 88.49(3.6) | 91.44(7.2) | 96.35(12.8) | 94.35(10.3) | 90.34(8.7) | 89.35(7.4) | 88.49(4.7) | 85.34(3.5) |
| 328 | Cyprodinil | 73.19(5.4) | 77.48(3.5) | 81.49(5.2) | 81.49(3.4) | 84.79(6.4) | 87.19(7.5) | 93.55(6.4) | 91.47(5.7) | 88.67(4.3) | 105.69(7.0) | 102.57(4.4) | 98.37(2.6) |
| 329 | Fuberidazole | 70.28(4.6) | 73.49(3.8) | 75.19(6.6) | 83.49(3.4) | 85.47(6.5) | 86.49(8.1) | 88.65(7.3) | 86.39(5.5) | 82.37(2.5) | 86.35(8.6) | 85.47(7.2) | 82.49(5.4) |
| 330 | Isofenphos-oxon | 81.63(4.9) | 83.49(6.2) | 85.48(6.4) | 72.49(5.1) | 76.41(5.8) | 76.49(3.4) | 78.65(15.2) | 75.36(12.4) | 80.39(6.8) | 72.49(5.8) | 76.41(6.0) | 75.33(3.5) |
| 331 | Dicapthon | 63.15(20.5) | 68.49(17.6) | 70.19(5.5) | 63.87(5.5) | 68.49(14.5) | 64.89(6.9) | 87.35(9.4) | 86.39(7.5) | 82.69(6.3) | 80.36(16.9) | 76.58(14.6) | 74.89(8.3) |
| 332 | 2,2',4,5,5'-Pentachlorobiphenyl | 72.15(11.6) | 73.59(8.9) | 78.29(6.7) | 74.15(5.4) | 76.59(6.4) | 80.49(8.7) | 84.25(9.4) | 81.28(7.2) | 79.33(6.4) | 89.36(5.7) | 86.59(6.5) | 85.15(3.0) |
| 333 | MCPA-butoxyethyl ester | 74.52(6.3) | 78.49(7.8) | 80.16(4.8) | 88.15(4.9) | 90.18(6.7) | 91.74(8.6) | 73.55(11.3) | 73.59(8.4) | 76.39(8.6) | 97.58(6.5) | 96.37(4.3) | 94.28(4.2) |
| 334 | Isocarbophos | 92.48(5.4) | 88.19(7.9) | 85.49(6.3) | 93.48(5.9) | 96.81(7.8) | 100.26(9.5) | N.D | N.D | 72.36(15.0) | N.D | N.D | 83.26(15.5) |
| 335 | Phorate sulfone | 93.48(4.8) | 92.57(6.9) | 88.12(3.6) | 86.37(5.1) | 88.69(7.2) | 91.48(8.6) | 99.35(7.5) | 96.58(6.3) | 94.23(4.2) | 90.87(7.5) | 88.69(8.2) | 85.23(6.3) |
| 336 | Chlorfenethol | 71.49(5.9) | 75.16(6.4) | 77.19(8.1) | 85.12(3.9) | 87.19(6.4) | 91.74(9.6) | 85.65(11.5) | 83.26(8.7) | 80.23(4.1) | 89.65(8.4) | 87.19(6.4) | 85.74(5.2) |
| 337 | trans-Nonachlor | 65.32(9.4) | 68.31(4.9) | 72.49(8.2) | 88.31(6.2) | 92.48(7.2) | 95.49(6.4) | 89.25(8.3) | 87.39(6.3) | 86.39(4.3) | 98.26(4.3) | 96.57(7.3) | 95.49(4.3) |
| 338 | Dinobuton | 93.18(5.3) | 96.48(7.2) | 100.26(4.9) | 84.25(5.1) | 86.49(7.2) | 89.47(3.8) | 106.5(6.3) | 102.69(5.2) | 99.38(2.5) | 99.38(6.5) | 97.58(5.2) | 94.68(4.3) |
| 339 | Tribufos | 84.21(10.3) | 86.49(13.5) | 90.18(4.9) | 77.29(6.4) | 79.63(8.4) | 82.19(6.8) | 98.25(10.4) | 96.49(13.9) | 92.18(6.0) | 92.38(8.3) | 90.28(6.4) | 87.46(5.3) |
| 340 | Flurochloridone | 73.19(8.4) | 76.29(6.7) | 80.19(5.8) | 92.48(6.4) | 95.48(6.4) | 97.14(9.2) | N.D | 72.36(7.6) | 74.16(8.7) | N.D | 90.28(7.7) | 93.46(5.9) |
| 341 | Bromfenvinphos | 71.49(5.9) | 75.23(8.4) | 77.12(6.3) | 90.12(4.4) | 93.48(6.5) | 95.47(3.4) | 88.35(7.3) | 86.39(5.8) | 84.26(4.4) | 86.95(8.4) | 84.26(7.2) | 80.29(5.4) |
| 342 | Perthan | 87.21(6.3) | 89.46(7.2) | 90.85(3.4) | 92.78(6.8) | 96.31(7.6) | 98.17(5.4) | 98.65(4.3) | 96.35(5.7) | 92.76(2.6) | 99.37(7.5) | 97.63(5.5) | 95.38(6.1) |
| 343 | Ditalimfos | 70.23(11.5) | 72.64(8.1) | 75.36(5.4) | 119.63(4.8) | 125.4(8.2) | 126.8(6.9) | 93.65(8.7) | 90.58(7.3) | 88.36(5.5) | 78.65(8.2) | 75.62(5.3) | 74.59(4.2) |
| 344 | 2,3,4,4',5-Pentachlorobiphenyl | 67.29(12.3) | 70.25(8.2) | 73.15(10.6) | 74.25(3.2) | 75.12(6.3) | 77.16(8.2) | 87.65(7.1) | 89.66(6.4) | 85.63(4.2) | 80.25(13.9) | 77.63(9.9) | 75.39(6.5) |
| 345 | 4,4'-Dibromobenzophenone | 72.15(6.3) | 74.18(6.2) | 77.39(5.3) | 94.21(5.3) | 96.74(6.8) | 102.54(8.2) | 99.35(13.7) | 97.52(8.3) | 95.43(6.5) | 105.36(6.4) | 102.35(5.7) | 98.73(7.3) |
| 346 | Flutriafol | 81.75(12.3) | 85.49(6.4) | 88.52(3.6) | 87.45(2.7) | 95.43(7.4) | 92.48(7.9) | 97.55(6.5) | 95.48(5.3) | 93.47(2.2) | 97.26(8.3) | 95.43(7.5) | 92.48(3.7) |
| 347 | Mephosfolan | 81.25(10.6) | 83.49(7.2) | 85.49(6.2) | 82.39(4.7) | 85.64(6.8) | 88.49(11.3) | 86.35(6.4) | 85.37(5.5) | 82.16(4.2) | 93.68(7.2) | 90.25(4.7) | 88.49(5.3) |
| 348 | Athidathion | 89.34(5.2) | 92.85(6.7) | 96.48(7.8) | 85.74(13.2) | 88.46(9.7) | 91.48(6.7) | 97.65(6.4) | 95.28(7.2) | 94.58(2.7) | 96.85(6.4) | 94.28(4.4) | 89.36(2.5) |
| 349 | 2,2',4,4',5,5'-Hexachlorobiphenyl | N.D | 64.85(3.5) | 68.29(11.6) | N.D | 59.81(4.6) | 62.54(9.8) | N.D | N.D | 94.25(15.4) | N.D | N.D | 73.59(11.5) |
| 350 | Diclobutrazol | 77.25(8.4) | 79.64(8.5) | 83.49(11.3) | 91.46(2.4) | 93.47(5.2) | 95.49(6.4) | 105.5(6.4) | 102.48(9.3) | 98.74(5.4) | 99.32(6.4) | 96.54(4.2) | 95.49(3.7) |
| 351 | Disulfoton-sulfone | 81.75(12.3) | 85.49(6.4) | 88.52(3.6) | 94.21(3.3) | 96.18(2.4) | 99.15(8.2) | 92.55(8.6) | 89.25(5.2) | 87.19(2.6) | 98.25(7.5) | 96.18(6.4) | 94.75(8.6) |
| 352 | Hexythiazox | 74.28(6.3) | 76.92(7.5) | 80.54(6.5) | 74.25(6.9) | 77.95(8.7) | 80.31(5.6) | 95.85(11.2) | 94.75(7.6) | 90.25(2.8) | 88.67(9.3) | 86.57(6.6) | 84.71(3.3) |
| 353 | 2,2',3,4,4',5'-Hexachlorobiphenyl | N.D | N.D | 72.19(6.4) | N.D | N.D | 68.24(6.8) | N.D | N.D | N.D | N.D | N.D | N.D |
| 354 | Triamiphos | 90.25(6.4) | 92.48(7.5) | 95.48(9.3) | 88.17(4.3) | 91.58(5.7) | 93.18(9.4) | 95.35(7.3) | 92.48(8.4) | 88.16(4.2) | 78.69(13.6) | 76.59(10.3) | 75.42(3.5) |
| 355 | Resmethrin | 92.18(6.3) | 95.48(7.5) | 99.18(10.5) | 71.25(4.3) | 76.54(5.4) | 80.52(7.6) | 84.25(8.4) | 81.75(8.3) | 79.38(10.6) | 88.25(9.4) | 85.26(4.6) | 83.27(6.5) |
| 356 | Cyproconazole | 66.35(4.8) | 68.49(11.5) | 71.56(5.6) | 90.35(4.6) | 93.48(7.2) | 95.41(8.5) | 86.35(7.5) | 85.39(6.6) | 83.56(4.2) | 95.26(7.5) | 93.48(4.3) | 88.25(3.7) |
| 357 | Benzyl butyl phthalate | 74.15(6.6) | 78.25(6.7) | 80.25(6.9) | 95.14(4.2) | 97.18(6.3) | 98.25(7.7) | 82.55(8.6) | 78.25(5.3) | 76.58(8.1) | 92.38(6.6) | 90.28(4.2) | 87.56(6.4) |
| 358 | Clodinafop-propargyl | 71.25(6.4) | 75.36(4.9) | 77.38(6.3) | 84.25(3.5) | 86.39(4.8) | 88.96(8.3) | 96.35(9.4) | 94.26(7.2) | 93.48(5.2) | 82.57(11.5) | 83.59(8.5) | 86.37(4.3) |
| 359 | Fenthion sulfoxide | 86.35(4.8) | 88.39(10.2) | 90.48(6.3) | 82.49(5.5) | 85.64(7.2) | 88.49(9.2) | 87.55(6.4) | 88.39(8.7) | 84.29(5.5) | 98.25(7.2) | 95.38(6.3) | 93.12(6.5) |
| 360 | Fluotrimazole | 56.39(18.4) | 65.31(7.2) | 70.25(8.9) | 87.63(5.2) | 89.74(6.8) | 92.48(7.6) | 65.25(24.7) | 68.38(21.0) | 70.25(17.6) | 89.36(6.5) | 86.59(7.2) | 85.29(4.3) |
| 361 | Fluroxypyr-1-methylheptyl ester | 82.59(6.2) | 85.97(7.9) | 87.19(8.9) | 71.49(4.2) | 77.49(6.8) | 80.15(8.2) | 97.25(7.6) | 95.38(6.5) | 92.75(11.4) | 97.46(5.3) | 95.84(7.3) | 93.66(3.5) |
| 362 | Fenthion-sulfone | 73.59(8.5) | 75.49(6.9) | 78.45(3.9) | 92.58(6.2) | 93.49(7.5) | 95.84(7.5) | 92.55(12.7) | 89.63(8.7) | 87.42(5.0) | 92.58(8.5) | 93.49(4.4) | 88.67(9.3) |
| 363 | Triphenyl phosphate | 76.52(3.2) | 79.65(4.5) | 82.15(4.6) | 81.24(6.9) | 83.59(6.3) | 85.94(7.7) | 87.55(6.4) | 85.34(7.1) | 82.69(6.4) | 84.69(9.3) | 85.23(8.5) | 80.26(6.4) |
| 364 | Metamitron | 80.25(5.2) | 83.19(6.4) | 86.19(8.7) | 91.46(5.4) | 93.47(6.2) | 95.49(8.2) | 106.5(7.3) | 104.26(5.4) | 101.69(2.1) | 80.57(12.5) | 78.36(9.3) | 76.59(6.1) |
| 365 | 2,2',3,4,4',5,5'-Heptachlorobiphenyl | 92.54(5.6) | 93.48(7.4) | 96.78(6.8) | 82.49(3.9) | 85.39(5.7) | 78.39(10.2) | N.D | 88.63(14.5) | 87.26(12.7) | N.D | 72.46(13.3) | 73.41(14.2) |
| 366 | Tebufenpyrad | 92.58(4.8) | 95.48(6.7) | 99.48(8.3) | 98.47(6.4) | 99.84(6.7) | 102.36(6.8) | 97.25(5.4) | 95.48(6.8) | 91.28(2.8) | 96.35(8.2) | 99.84(6.8) | 92.57(4.2) |
| 367 | Cloquintocet-mexyl | 95.68(4.8) | 98.63(5.7) | 100.5(4.4) | 75.23(3.4) | 78.65(4.8) | 79.84(5.1) | 96.35(6.5) | 92.64(7.2) | 90.25(5.3) | 97.25(6.4) | 95.16(7.3) | 92.34(4.1) |
| 368 | Lenacil | 85.62(4.8) | 88.67(9.4) | 90.25(10.3) | 87.49(4.3) | 88.96(6.3) | 90.48(8.2) | 81.25(12.6) | 78.56(9.3) | 76.45(4.2) | 94.28(7.2) | 91.64(5.4) | 88.25(1.4) |
| 369 | Bromuconazole | 85.23(6.4) | 88.76(4.6) | 90.15(13.4) | 80.26(4.9) | 82.49(5.6) | 87.65(5.9) | 97.45(8.1) | 95.62(6.4) | 91.46(5.3) | 106.39(6.8) | 104.28(6.3) | 101.25(4.1) |
| 370 | Nitralin | 92.48(5.6) | 90.25(7.4) | 87.26(3.8) | 92.85(4.2) | 94.68(5.5) | 96.87(6.7) | 89.65(8.2) | 90.25(5.7) | 86.35(6.3) | 98.55(7.2) | 94.68(6.0) | 96.87(6.8) |
| 371 | Fenamiphos sulfoxide | 86.52(6.4) | 88.49(6.9) | 90.68(6.8) | 92.46(3.5) | 90.58(5.6) | 87.21(4.6) | 94.25(6.8) | 91.46(5.4) | 89.22(7.2) | 96.38(6.4) | 94.28(4.1) | 92.25(6.4) |
| 372 | Fenamiphos sulfone | 79.56(5.5) | 78.36(6.4) | 83.49(14.9) | 74.25(6.8) | 76.95(8.4) | 80.64(6.4) | 91.55(7.4) | 88.29(6.4) | 85.17(2.6) | 95.68(6.9) | 92.47(4.7) | 89.67(3.1) |
| 373 | Fenpiclonil | 94.21(5.5) | 96.87(7.6) | 102.56(6.4) | 93.48(5.4) | 96.87(5.9) | 99.54(6.7) | 102.5(6.4) | 99.47(7.6) | 96.51(10.3) | 100.25(8.7) | 96.87(5.3) | 94.75(3.3) |
| 374 | Fluquinconazole | 67.54(5.1) | 69.77(8.4) | 74.16(12.6) | 92.84(3.7) | 94.85(5.3) | 89.61(3.4) | 97.15(7.5) | 95.28(11.4) | 94.12(8.3) | 94.25(9.7) | 93.18(7.3) | 91.24(2.1) |
| 375 | Fenbuconazole | 86.25(6.3) | 88.59(7.8) | 90.18(9.4) | 85.74(4.2) | 87.49(6.4) | 90.57(7.8) | 75.25(9.4) | 77.23(8.2) | 75.33(5.5) | 95.62(4.2) | 93.22(5.2) | 90.57(6.0) |
| 376 | Ethylene brassylate | 84.15(4.2) | 87.96(6.8) | 90.36(6.8) | 85.21(6.3) | 88.49(7.5) | 90.87(8.3) | 90.25(12.4) | 87.96(8.6) | 85.46(5.1) | 88.75(12.4) | 85.74(8.3) | 86.59(4.2) |
| 377 | Propoxur | 81.45(3.6) | 83.49(5.9) | 86.37(9.8) | 91.25(5.6) | 94.76(5.8) | 95.28(8.4) | 99.65(6.6) | 94.25(5.4) | 92.45(3.5) | 97.68(5.5) | 95.27(3.5) | 92.48(3.2) |
| 378 | Isoprocarb | 76.35(5.6) | 79.64(5.8) | 82.49(13.2) | 84.25(1.3) | 88.49(7.8) | 90.15(4.8) | 96.35(8.3) | 92.67(6.5) | 90.58(4.3) | 90.87(5.5) | 88.49(4.7) | 85.23(2.5) |
| 379 | Methamidophos | 70.25(6.4) | 73.59(7.4) | 76.94(8.3) | 91.25(5.8) | 95.34(6.4) | 93.48(5.7) | 98.65(10.3) | 96.37(8.5) | 90.87(6.6) | 104.85(8.8) | 100.57(6.4) | 96.25(4.2) |
| 380 | Acenaphthene | 70.25(4.2) | 73.58(7.5) | 76.38(4.9) | 62.58(13.4) | 65.49(14.6) | 70.15(5.8) | 78.65(8.1) | 80.67(6.4) | 82.76(4.3) | 88.47(11.0) | 86.23(8.4) | 84.75(5.5) |
| 381 | Dibutyl succinate | 82.59(6.4) | 83.49(7.5) | 88.56(8.3) | 85.64(6.4) | 87.94(5.8) | 90.25(6.4) | 96.85(8.1) | 95.28(6.4) | 92.48(9.6) | 96.15(6.2) | 93.67(5.2) | 90.47(3.9) |
| 382 | Phthalimide | 91.58(4.1) | 93.48(6.5) | 96.48(7.5) | 82.59(4.2) | 85.76(6.3) | 88.64(7.2) | 99.65(5.2) | 97.58(6.3) | 95.28(4.2) | 88.57(7.2) | 85.49(6.7) | 83.54(4.2) |
| 383 | Chlorethoxyfos | 83.49(3.5) | 86.49(5.6) | 88.49(7.2) | 94.85(5.6) | 92.58(5.7) | 89.34(10.2) | 98.65(4.3) | 96.52(5.8) | 92.48(3.6) | 92.41(9.6) | 90.14(8.5) | 87.52(5.2) |
| 384 | Pencycuron | 72.58(6.4) | 75.89(7.3) | 77.65(8.7) | 88.34(4.2) | 90.35(5.8) | 95.28(8.4) | 82.65(8.5) | 80.27(6.4) | 77.84(4.1) | 94.78(6.3) | 92.67(4.2) | 89.52(3.5) |
| 385 | Tebuthiuron | 62.35(20.5) | 68.35(7.8) | 71.56(11.3) | 75.23(4.6) | 78.64(4.9) | 82.59(6.8) | 86.55(15.9) | 87.96(13.5) | 84.52(9.6) | 98.74(6.3) | 96.47(4.0) | 93.74(2.2) |
| 386 | Demeton-S-methyl | 76.35(5.4) | 80.39(4.7) | 81.49(11.5) | 94.68(5.9) | 98.64(3.8) | 100.3(7.2) | 86.45(8.6) | 82.67(7.2) | 79.68(5.1) | 105.24(9.8) | 101.64(6.4) | 98.24(5.1) |
| 387 | Cadusafos | 84.58(6.1) | 88.67(3.9) | 90.25(6.9) | 88.47(2.8) | 90.18(3.6) | 86.15(7.9) | 96.25(9.0) | 93.47(4.6) | 90.16(2.5) | 97.11(6.4) | 94.18(5.2) | 92.48(2.8) |
| 388 | Phenanthrene | 80.95(2.8) | 83.48(4.4) | 85.16(9.5) | 77.18(4.6) | 82.17(6.5) | 85.49(6.9) | 95.15(6.3) | 93.48(4.9) | 90.21(3.5) | 85.41(7.1) | 82.94(6.2) | 80.44(5.3) |
| 389 | Spiroxamine | 89.64(6.4) | 90.28(3.9) | 92.48(7.5) | 89.58(6.1) | 92.18(6.1) | 87.18(6.9) | 87.55(5.7) | 86.95(3.7) | 82.76(2.5) | 95.24(4.2) | 91.37(2.5) | 90.21(3.7) |
| 390 | Fenpyroximate | 81.74(3.6) | 85.49(6.2) | 88.49(7.5) | 71.04(2.9) | 76.18(3.9) | 60.18(18.5) | 94.75(6.8) | 91.42(4.3) | 88.76(3.2) | 90.25(4.1) | 88.75(5.2) | 86.57(7.2) |
| 391 | Tebupirimfos | 89.25(6.8) | 92.85(4.5) | 95.84(6.6) | 84.17(2.6) | 88.17(6.8) | 89.32(4.4) | 91.25(8.8) | 86.35(7.2) | 84.19(5.5) | 94.28(3.6) | 91.66(4.2) | 89.32(2.2) |
| 392 | Prohydrojasmon | 79.45(2.2) | 83.96(4.5) | 86.24(6.6) | 89.14(6.2) | 93.54(7.5) | 95.45(2.6) | 92.85(4.8) | 89.67(3.5) | 86.24(5.2) | 98.57(7.1) | 97.15(6.4) | 95.14(4.3) |
| 393 | Fenpropidin | 60.49(5.6) | 70.84(3.1) | 72.68(6.7) | 80.95(4.1) | 83.45(8.5) | 86.54(6.9) | 79.65(8.3) | 77.49(3.3) | 75.42(4.2) | 94.16(5.2) | 96.38(6.3) | 92.57(4.3) |
| 394 | Dicloran | 92.45(4.4) | 95.76(6.4) | 88.76(6.3) | 79.65(6.2) | 83.49(6.9) | 85.41(6.3) | 98.25(7.2) | 96.17(6.2) | 95.21(5.2) | 98.67(4.5) | 94.58(5.3) | 92.68(3.6) |
| 395 | Pyroquilon | 90.85(6.1) | 92.76(10.2) | 94.81(7.9) | 76.48(3.2) | 78.94(5.5) | 81.49(8.1) | 93.85(8.6) | 90.52(6.3) | 87.49(5.2) | 96.25(8.7) | 94.18(6.3) | 92.48(5.3) |
| 396 | Propyzamide | 80.96(4.8) | 83.25(5.6) | 85.71(9.4) | 90.18(3.2) | 93.18(7.1) | 89.64(2.2) | 94.75(6.6) | 92.74(4.6) | 89.22(2.4) | 96.54(7.2) | 94.16(6.3) | 91.48(4.2) |
| 397 | Pirimicarb | 63.84(16.4) | 69.84(6.4) | 71.74(6.4) | 92.45(3.9) | 93.54(5.4) | 96.87(6.5) | 94.65(13.7) | 91.48(9.9) | 88.62(10.4) | 92.68(6.4) | 89.64(4.2) | 86.37(3.0) |
| 398 | Phosphamidon | 81.04(3.8) | 85.97(4.1) | 88.54(3.8) | 85.64(4.8) | 88.54(4.5) | 90.54(5.4) | 95.85(5.4) | 92.76(6.4) | 89.64(4.2) | 90.15(5.2) | 87.49(3.7) | 85.24(6.4) |
| 399 | Benoxacor | 94.28(2.9) | 95.47(6.4) | 98.75(5.4) | 66.87(6.5) | 72.51(6.3) | 85.24(3.5) | 87.65(7.3) | 89.63(6.3) | 93.64(5.9) | 79.68(2.5) | 83.67(5.5) | 85.24(8.9) |
| 400 | Bromobutide | 92.54(5.4) | 83.64(3.5) | 79.54(6.7) | 71.25(3.8) | 78.65(3.9) | 85.61(5.8) | 83.65(6.6) | 85.24(7.2) | 88.69(4.2) | 85.26(7.4) | 87.62(6.4) | 92.68(8.5) |
| 401 | Acetochlor | 94.84(6.1) | 92.14(6.9) | 86.13(9.7) | 80.67(4.8) | 82.56(6.5) | 88.65(6.4) | 96.25(8.5) | 94.61(7.3) | 92.64(6.4) | 91.68(6.4) | 89.64(5.4) | 86.37(4.2) |
| 402 | Tridiphane | 98.45(5.8) | 95.48(6.9) | 93.42(4.8) | 71.68(2.9) | 85.48(6.2) | 90.25(9.1) | 101.5(7.3) | 98.64(5.2) | 95.24(6.7) | 96.84(7.6) | 93.67(5.2) | 90.58(4.7) |
| 403 | Terbucarb | 79.52(3.8) | 83.47(8.56) | 85.47(9.5) | 82.14(6.0) | 85.14(3.9) | 85.04(6.2) | 95.65(8.6) | 92.58(7.4) | 89.63(5.2) | 103.96(7.7) | 100.58(8.6) | 98.17(5.3) |
| 404 | Esprocarb | 95.87(6.8) | 87.47(7.1) | 83.86(8.4) | 74.15(4.2) | 77.09(6.2) | 71.05(3.8) | 98.65(7.0) | 96.34(5.3) | 93.68(4.6) | 85.67(6.8) | 83.54(5.3) | 79.54(5.0) |
| 405 | Fenfuram | 89.52(6.5) | 84.58(3.9) | 79.23(10.9) | 82.15(6.1) | 85.64(7.8) | 89.45(5.4) | 94.25(9.6) | 91.28(7.5) | 88.57(4.2) | 78.95(8.3) | 81.46(5.2) | 80.49(7.2) |
| 406 | Acibenzolar-S-methyl | 82.54(6.9) | 85.47(9.6) | 88.54(6.8) | 82.67(2.2) | 86.24(3.1) | 93.25(5.8) | N.D | N.D | 72.15(14.9) | N.D | N.D | 71.02(11.7) |
| 407 | Benfuresate | 108.52(6.3) | 93.65(4.8) | 92.51(2.6) | 76.15(3.2) | 73.85(4.8) | 77.68(9.6) | N.D | N.D | 79.64(10.2) | N.D | N.D | 75.42(8.6) |
| 408 | Dithiopyr | 72.48(3.6) | 75.62(5.6) | 78.69(3.4) | 83.41(6.3) | 85.67(6.8) | 88.62(6.5) | 96.05(8.2) | 93.15(4.3) | 89.47(2.2) | 88.14(8.0) | 86.34(4.2) | 82.54(5.6) |
| 409 | Metalaxyl-m | 80.25(5.1) | 85.54(4.2) | 91.54(6.3) | 90.25(3.5) | 92.85(4.6) | 93.87(3.9) | 74.15(6.3) | 76.85(10.5) | 79.64(6.3) | 93.64(7.2) | 90.45(7.0) | 88.23(4.6) |
| 410 | Malaoxon | 73.56(5.2) | 75.86(4.9) | 75.19(3.8) | 70.35(4.5) | 73.26(6.4) | 75.96(4.8) | 88.15(7.1) | 84.24(6.9) | 81.87(4.7) | 97.54(6.4) | 94.67(5.5) | 91.76(2.5) |
| 411 | Simeconazole | 81.45(6.7) | 84.59(8.6) | 87.15(4.9) | 81.59(6.2) | 84.69(6.1) | 88.74(7.2) | 95.65(10.2) | 92.76(8.5) | 89.67(2.2) | 79.46(8.0) | 77.14(7.7) | 75.30(5.4) |
| 412 | Chlorthal-dimethyl | 83.45(2.6) | 84.18(5.3) | 86.49(2.8) | 108.52(6.3) | 93.65(4.8) | 92.51(2.6) | 93.55(7.5) | 91.27(6.5) | 88.34(4.1) | 85.94(8.1) | 82.64(7.2) | 79.64(3.5) |
| 413 | Thiazopyr | 66.75(6.5) | 70.15(6.5) | 72.46(5.9) | 72.48(3.6) | 75.62(5.6) | 78.69(3.4) | 88.65(8.6) | 84.38(6.3) | 81.94(5.7) | 94.63(5.8) | 92.46(2.2) | 88.64(1.9) |
| 414 | Dimethylvinphos | 84.18(3.2) | 86.15(6.5) | 88.19(3.8) | 73.54(8.6) | 75.64(9.2) | 78.65(6.7) | 95.65(7.5) | 91.28(4.7) | 87.45(5.3) | 83.96(7.6) | 85.29(7.2) | 80.22(4.9) |
| 415 | Butralin | 82.54(3.6) | 84.98(5.5) | 87.49(3.8) | 74.12(3.6) | 76.48(6.) | 78.54(6.4) | 77.55(5.6) | 79.55(4.3) | 83.64(4.3) | 89.69(11.3) | 92.64(7.9) | 85.69(6.5) |
| 416 | Zoxamide | 74.65(3.1) | 78.95(6.2) | 80.65(6.4) | 91.58(7.5) | 88.54(6.5) | 83.49(7.6) | 88.65(5.8) | 90.14(4.4) | 92.58(8.0) | 94.78(9.6) | 92.55(6.4) | 89.51(4.1) |
| 417 | Pyrifenox | 82.54(3.2) | 85.19(5.1) | 88.59(7.1) | 91.58(5.4) | 95.24(7.6) | 99.68(9.4) | 85.65(8.0) | 86.45(6.4) | 81.49(2.5) | 96.78(10.3) | 93.67(6.3) | 88.73(2.3) |
| 418 | Allethrin | 86.21(1.2) | 83.17(9.5) | 79.61(15.3) | 102.5(4.6) | 98.54(5.8) | 95.42(6.3) | 82.65(5.2) | 80.49(3.5) | 77.64(1.4) | 94.56(2.1) | 91.68(2.9) | 88.34(2.0) |
| 419 | Dimethametryn | 82.59(15.9) | 80.49(6.4) | 76.52(3.9) | 95.24(3.6) | 98.64(5.6) | 100.26(6.3) | 86.55(5.3) | 84.97(3.0) | 81.49(5.8) | 88.49(8.3) | 85.22(3.6) | 81.45(3.0) |
| 420 | Quinoclamine | 92.48(3.8) | 95.68(4.9) | 99.64(8.5) | 87.63(5.4) | 90.25(4.8) | 92.48(6.4) | 104.5(5.4) | 100.48(2.1) | 98.54(3.9) | 95.67(4.4) | 92.31(3.7) | 90.28(7.6) |
| 421 | Methothrin | N.D | 78.25(4.6) | 82.59(6.4) | 91.85(7.2) | 93.48(8.5) | 95.89(10.8) | 82.45(10.3) | 80.47(8.1) | 77.65(4.0) | N.D | N.D | 72.16(5.2) |
| 422 | Flufenacet | 83.54(6.1) | 86.49(8.2) | 90.56(6.9) | 84.96(6.2) | 88.75(3.6) | 87.16(3.9) | 95.45(7.3) | 92.28(5.1) | 89.37(1.5) | 87.15(6.3) | 85.67(4.2) | 82.46(2.4) |
| 423 | Fenoxanil | 63.25(16.4) | 70.56(7.8) | 72.36(5.4) | 92.65(5.4) | 90.78(3.9) | 88.16(6.4) | 95.15(6.2) | 92.47(5.2) | 89.55(3.0) | 95.47(6.3) | 92.14(4.4) | 90.21(2.5) |
| 424 | Fthalide | 83.54(6.2) | 85.67(6.8) | 88.96(8.7) | 87.14(4.6) | 89.52(7.6) | 92.48(6.1) | 91.25(8.3) | 89.74(5.6) | 86.52(4.4) | 94.58(6.2) | 95.48(10.3) | 91.24(4.2) |
| 425 | Furalaxyl | 93.25(4.8) | 96.58(8.4) | 94.57(6.1) | 65.28(10.6) | 69.99(4.9) | 73.49(8.9) | 93.25(9.0) | 96.58(4.2) | 90.21(2.2) | 88.48(5.0) | 85.27(8.0) | 83.02(6.1) |
| 426 | Thiamethoxam | 84.15(6.2) | 85.78(4.2) | 88.91(6.4) | 84.75(5.3) | 86.54(9.8) | 90.25(6.8) | 80.95(13.7) | 77.49(10.2) | 75.49(7.0) | 88.16(7.0) | 86.54(6.2) | 83.15(4.0) |
| 427 | Mepanipyrim | 74.85(2.7) | 80.16(6.8) | 86.14(8.5) | 74.26(5.8) | 79.18(7.1) | 82.16(6.8) | 89.75(7.0) | 87.31(5.5) | 84.27(3.7) | 90.68(5.8) | 92.84(4.8) | 87.55(5.2) |
| 428 | Captan | 72.15(6.2) | 75.68(5.8) | 78.68(8.2) | 71.49(6.6) | 78.16(6.9) | 82.96(9.5) | 99.85(5.6) | 95.37(2.6) | 92.77(3.6) | 80.14(5.2) | 78.16(6.5) | 74.65(3.1) |
| 429 | Bromacil | 81.64(6.4) | 85.75(6.8) | 90.58(11.2) | 97.16(6.2) | 99.85(8.6) | 102.94(9.7) | 96.55(4.3) | 94.74(5.6) | 93.74(2.5) | 101.24(3.3) | 97.66(5.3) | 95.14(2.4) |
| 430 | Picoxystrobin | 74.58(5.1) | 78.69(6.7) | 81.59(6.3) | 85.49(6.9) | 88.95(6.9) | 90.25(7.5) | 94.25(7.3) | 91.24(5.4) | 89.45(3.5) | 97.88(6.3) | 94.15(7.7) | 90.25(5.0) |
| 431 | Butamifos | 77.65(2.6) | 82.65(5.6) | 88.65(5.3) | 94.58(5.6) | 91.28(7.2) | 88.15(11.2) | 85.95(8.2) | 82.65(4.6) | 79.25(1.3) | 78.84(8.0) | 74.54(6.3) | 73.15(5.2) |
| 432 | Imazamethabenz-methyl | 73.84(6.6) | 75.49(7.2) | 77.68(6.8) | 72.56(5.2) | 75.24(8.2) | 77.16(8.2) | 89.25(8.5) | 86.35(6.4) | 83.64(2.2) | 96.77(7.2) | 94.36(6.3) | 91.47(5.4) |
| 433 | (E)-Metominostrobin | 90.58(3.5) | 93.48(6.5) | 95.48(8.4) | 71.59(6.9) | 73.69(4.9) | 79.35(6.5) | 94.55(8.0) | 91.48(6.3) | 88.47(3.2) | 98.64(4.4) | 95.84(2.1) | 93.58(2.5) |
| 434 | TCMTB | 87.15(13.4) | 89.47(5.9) | 91.84(7.2) | 75.21(6.3) | 78.65(6.5) | 80.25(8.5) | 94.25(7.7) | 94.18(10.3) | 91.58(5.7) | 95.48(8.3) | 91.37(6.5) | 88.76(5.4) |
| 435 | Methiocarb sulfone | 74.18(6.1) | 80.15(6.4) | 85.17(6.2) | 95.18(3.3) | 91.08(6.1) | 97.48(5.8) | 83.65(7.5) | 80.31(8.6) | 79.48(6.5) | 115.69(14.7) | 116.79(10.3) | 108.76(8.5) |
| 436 | Imazalil | 74.16(5.4) | 76.89(7.9) | 82.59(8.6) | 91.54(5.5) | 88.26(6.4) | 84.69(6.8) | 74.15(13.5) | 77.15(8.5) | 79.48(5.6) | 105.45(6.3) | 101.23(4.1) | 98.03(2.4) |
| 437 | Isoprothiolane | 84.76(7.2) | 88.95(6.2) | 91.54(3.8) | 86.35(4.5) | 88.69(5.9) | 90.25(7.8) | 95.65(7.3) | 93.45(5.3) | 91.54(4.1) | 97.54(6.3) | 95.46(4.1) | 90.25(3.5) |
| 438 | Cyflufenamid | 81.54(2.1) | 84.75(6.5) | 87.63(9.7) | 91.84(6.3) | 95.48(5.2) | 94.12(6.3) | 97.15(3.5) | 95.22(3.5) | 92.15(4.9) | 103.45(6.2) | 99.67(5.5) | 95.45(3.2) |
| 439 | Pyriminobac-methyl | 86.54(6.2) | 88.94(8.5) | 91.87(10.5) | 81.48(1.9) | 85.64(7.2) | 88.94(8.3) | 78.55(6.3) | 80.25(5.6) | 77.15(3.4) | 125.48(11.3) | 122.47(8.9) | 116.39(4.6) |
| 440 | Isoxathion | 94.21(3.6) | 98.41(6.9) | 103.54(7.2) | 82.15(6.3) | 83.84(5.2) | 84.95(11.6) | 94.25(5.3) | 91.46(7.3) | 89.15(6.5) | 98.64(8.0) | 96.35(4.7) | 92.34(2.2) |
| 441 | (Z)-Metominostrobin | 96.12(4.7) | 92.15(3.8) | 88.25(8.5) | 80.35(6.4) | 83.49(7.6) | 85.96(7.9) | 91.25(7.5) | 89.66(5.2) | 86.49(3.5) | 92.63(7.2) | 90.28(4.6) | 87.41(3.6) |
| 442 | Diofenolan | 93.48(5.6) | 95.78(7.5) | 99.48(11.6) | 84.58(6.3) | 85.96(7.2) | 87.49(10.6) | 96.35(4.3) | 94.68(5.1) | 91.47(4.9) | 89.67(8.3) | 87.41(6.5) | 85.14(4.2) |
| 443 | Thifluzamide | 99.63(4.5) | 97.25(4.9) | 94.68(8.5) | 77.16(3.6) | 78.14(6.4) | 81.46(7.5) | 89.65(5.5) | 92.18(7.3) | 86.31(5.1) | 96.28(5.5) | 94.18(7.0) | 91.02(5.4) |
| 444 | Quinoxyfen | 87.15(3.6) | 88.29(4.3) | 93.48(8.5) | 74.21(5.4) | 77.49(8.4) | 73.58(8.4) | 95.35(9.0) | 91.28(6.4) | 89.47(4.3) | 78.96(7.2) | 81.25(8.4) | 76.29(8.6) |
| 445 | Chlorfenapyr | 88.25(4.6) | 91.25(8.5) | 94.18(11.6) | 80.13(4.2) | 83.49(6.4) | 88.76(4.6) | 74.55(5.7) | 76.54(2.2) | 73.58(4.1) | 91.57(8.6) | 88.74(6.4) | 84.97(5.6) |
| 446 | Trifloxystrobin | N.D | N.D | N.D | N.D | N.D | N.D | 85.45(9.3) | 81.24(6.4) | 78.26(5.5) | 98.64(5.2) | 96.35(3.5) | 92.58(4.7) |
| 447 | Imibenconazole-oxon-desbenzyl | 72.15(6.9) | 77.49(8.2) | 74.12(11.3) | 80.25(5.2) | 83.19(6.4) | 86.19(8.7) | 82.35(9.4) | 83.65(8.3) | 78.52(4.6) | 86.95(7.5) | 85.24(6.4) | 83.27(3.7) |
| 448 | Isoxadifen-ethyl | 75.24(3.5) | 78.47(5.4) | 81.58(6.4) | 92.54(5.6) | 93.48(7.4) | 96.78(6.8) | 94.25(7.2) | 91.57(6.3) | 88.23(4.2) | 91.57(9.1) | 93.48(7.5) | 88.15(6.8) |
| 449 | Fipronil | 84.26(5.1) | 86.39(8.2) | 91.58(7.5) | 92.58(4.8) | 95.48(6.7) | 99.48(8.3) | 93.65(5.2) | 90.24(2.8) | 88.27(3.5) | 89.67(7.4) | 87.59(6.3) | 86.25(5.2) |
| 450 | Imiprothrin | 93.48(5.4) | 95.24(10.8) | 96.24(7.6) | 85.68(4.8) | 88.63(5.7) | 90.5(8.6) | 95.25(6.5) | 92.54(4.3) | 89.36(2.1) | 93.64(5.8) | 91.28(3.5) | 88.26(6.3) |
| 451 | Carfentrazone-ethyl | 93.65(4.6) | 96.58(6.9) | 98.54(5.8) | 85.62(4.8) | 88.67(9.4) | 90.25(10.3) | 105.5(12.6) | 101.48(9.6) | 98.54(6.4) | 94.68(8.2) | 91.58(6.3) | 89.64(5.2) |
| 452 | Epoxiconazole | 86.37(4.2) | 88.47(8.4) | 92.58(5.9) | 85.23(6.4) | 88.76(4.6) | 90.15(13.4) | 88.65(7.2) | 85.39(6.6) | 82.45(4.3) | 89.45(7.3) | 88.76(6.1) | 86.35(5.3) |
| 453 | Pyraflufen-ethyl | 75.21(4.6) | 76.87(6.8) | 79.37(5.1) | 92.48(5.6) | 90.25(7.4) | 83.47(3.8) | 85.35(6.4) | 83.64(7.3) | 79.66(3.5) | 92.48(8.7) | 90.25(7.7) | 88.57(4.5) |
| 454 | Pyributicarb | 88.36(7.3) | 89.34(5.4) | 92.76(5.4) | 86.52(6.4) | 88.49(6.9) | 90.68(6.8) | 77.65(8.0) | 75.48(5.4) | 73.46(6.4) | 87.49(6.5) | 88.49(4.3) | 84.57(6.3) |
| 455 | Thenylchlor | 77.49(6.7) | 79.34(8.3) | 82.49(7.6) | 79.56(5.5) | 78.36(6.4) | 85.49(14.9) | 93.65(8.2) | 91.25(7.4) | 88.32(5.6) | 94.58(7.2) | 92.56(5.5) | 89.67(3.5) |
| 456 | Clethodim | 94.87(10.5) | 97.15(4.2) | 97.18(6.4) | 99.18(6.1) | 105.15(3.8) | 109.63(7.3) | 95.75(8.5) | 93.22(6.5) | 90.37(5.1) | 98.76(8.5) | 95.78(6.1) | 92.14(5.5) |
| 457 | Mefenpyr-diethyl | 90.35(4.6) | 93.48(7.2) | 95.41(8.5) | 96.87(6.5) | 99.78(6.4) | 100.52(9.7) | 89.35(8.6) | 84.69(6.4) | 85.41(5.3) | 96.35(7.5) | 95.28(6.3) | 92.58(7.3) |
| 458 | Famphur | 95.14(4.2) | 91.18(6.3) | 88.25(7.7) | 94.87(6.4) | 90.23(6.9) | 86.52(8.4) | 90.25(6.4) | 91.18(7.6) | 88.25(5.5) | 91.28(8.4) | 87.65(5.2) | 86.52(6.6) |
| 459 | Etoxazole | 84.25(3.5) | 86.39(4.8) | 88.96(8.3) | 87.45(6.4) | 91.56(5.6) | 82.19(6.4) | 94.25(7.3) | 91.38(5.2) | 88.96(4.3) | 87.45(8.6) | 85.69(6.3) | 82.19(2.4) |
| 460 | Pyriproxyfen | 82.49(5.5) | 78.32(5.4) | 75.68(6.7) | 65.48(5.9) | 70.59(3.85) | 74.12(6.9) | 87.35(6.6) | 85.38(5.4) | 83.49(4.1) | 88.96(6.1) | 86.35(5.2) | 84.27(7.9) |
| 461 | Picolinafen | 87.63(5.2) | 89.74(6.8) | 92.48(7.6) | 97.58(6.5) | 99.66(6.5) | 102.5(9.8) | 95.35(6.5) | 92.56(7.4) | 89.63(5.2) | 98.65(7.4) | 96.38(5.3) | 92.57(2.6) |
| 462 | Iprodione | 71.49(4.2) | 77.49(6.8) | 80.15(8.2) | 103.58(2.9) | 95.78(5.8) | 96.85(5.8) | 90.25(8.9) | 89.36(6.5) | 85.37(4.7) | 86.25(7.1) | 83.25(8.5) | 80.23(2.7) |
| 463 | Piperophos | 92.58(6.2) | 93.49(7.5) | 95.84(7.5) | 83.49(7.2) | 86.49(10.2) | 90.85(5.9) | 75.35(7.1) | 72.59(8.2) | 74.12(6.4) | 90.35(7.6) | 86.49(4.6) | 85.96(6.4) |
| 464 | Ofurace | 83.56(3.4) | 88.76(4.8) | 95.73(5.1) | 89.48(3.5) | 92.54(6.7) | 95.54(10.5) | 94.25(7.5) | 91.38(6.6) | 88.35(7.2) | 89.48(6.5) | 87.35(7.5) | 85.34(2.4) |
| 465 | Bifenazate | 80.12(1.6) | 85.45(3.2) | 88.61(7.85) | 77.58(5.8) | 79.58(7.2) | 82.54(3.6) | 88.35(6.4) | 86.35(7.2) | 85.29(4.2) | 93.68(7.5) | 90.28(5.4) | 88.35(4.2) |
| 466 | Endrin ketone | 95.21(2.8) | 97.25(3.8) | 100.7(8.9) | 96.78(5.8) | 90.48(6.8) | 91.48(9.7) | 93.65(6.4) | 90.28(7.8) | 88.63(4.3) | 96.78(6.4) | 95.28(4.3) | 93.58(6.9) |
| 467 | Clomeprop | 80.21(4.2) | 82.58(3.9) | 85.67(6.45) | 84.67(2.9) | 88.95(6.3) | 90.87(6.5) | 96.35(8.3) | 94.26(7.2) | 89.35(6.5) | 89.36(9.0) | 88.95(7.4) | 85.35(4.2) |
| 468 | Fenamidone | 73.17(6.3) | 76.21(3.8) | 77.95(4.8) | 94.85(5.7) | 98.73(6.4) | 100.34(6.4) | 73.65(8.3) | 76.21(6.3) | 77.95(8.6) | 88.39(5.7) | 86.32(5.5) | 82.35(6.2) |
| 469 | Naproanilide | 87.75(3.8) | 85.64(6.2) | 83.85(5.6) | 100.45(3.8) | 95.74(6.7) | 103.85(10.2) | 95.65(8.2) | 93.28(7.2) | 90.32(6.4) | 98.35(6.3) | 95.74(5.2) | 92.57(2.5) |
| 470 | Pyraclostrobin | 75.46(2.8) | 77.85(4.8) | 82.69(5.9) | 94.87(15.4) | 92.58(5.5) | 97.58(8.8) | 94.65(6.4) | 91.26(5.4) | 88.72(4.2) | 103.28(5.7) | 99.25(7.5) | 97.58(2.4) |
| 471 | Lactofen | 91.58(6.3) | 94.25(6.5) | 87.36(10.4) | 89.75(4.5) | 87.52(7.9) | 83.49(3.9) | 85.65(9.4) | 81.49(7.3) | 83.69(4.7) | 95.62(8.5) | 93.64(6.2) | 88.67(4.5) |
| 472 | Tralkoxydim | 78.64(6.8) | 80.54(5.6) | 84.59(7.9) | 108.26(6.1) | 95.48(5.9) | 88.47(6.8) | 77.25(8.5) | 79.85(6.1) | 82.39(8.5) | 99.45(7.5) | 95.37(6.1) | 93.18(4.0) |
| 473 | Pyraclofos | 92.26(4.6) | 95.68(7.3) | 97.46(6.4) | 96.74(3.3) | 98.46(5.8) | 102.46(4.7) | 103.5(11.4) | 99.67(8.6) | 97.46(5.5) | 96.74(8.3) | 94.37(6.4) | 91.24(3.7) |
| 474 | Dialifos | 84.35(10.3) | 86.47(4.6) | 90.51(7.6) | 88.38(6.7) | 91.48(7.7) | 98.15(8.6) | 96.35(8.4) | 95.36(5.7) | 91.48(2.6) | 98.68(5.7) | 95.36(6.6) | 93.58(4.2) |
| 475 | Spirodiclofen | 83.49(6.3) | 86.54(7.8) | 89.64(8.5) | 58.63(17.8) | 65.48(13.7) | 70.32(5.4) | 93.25(8.0) | 91.67(7.2) | 89.64(6.2) | 94.62(12.4) | 93.68(8.6) | 89.65(4.6) |
| 476 | Halfenprox | 94.58(6.3) | 92.18(7.7) | 89.36(5.6) | 81.59(8.2) | 84.75(7.2) | 88.19(9.6) | 94.55(6.4) | 92.18(7.9) | 89.36(5.3) | 96.35(9.0) | 94.28(8.1) | 91.38(6.5) |
| 477 | Flurtamone | 73.56(4.3) | 75.39(5.9) | 78.39(7.5) | 73.28(4.9) | 77.39(6.9) | 80.56(7.2) | 93.65(8.3) | 91.64(7.3) | 89.67(5.2) | 85.62(7.5) | 83.26(6.5) | 79.68(2.4) |
| 478 | Pyriftalid | 81.59(6.3) | 88.26(4.9) | 90.26(7.6) | 82.51(7.3) | 85.63(7.5) | 88.35(10.3) | 88.55(6.4) | 85.62(8.6) | 87.45(2.2) | 91.25(6.3) | 88.25(7.5) | 85.26(5.4) |
| 479 | Silafluofen | 73.26(5.4) | 75.86(6.8) | 80.24(7.8) | 87.42(2.3) | 89.64(7.1) | 93.45(7.4) | 97.65(6.4) | 95.68(5.3) | 96.35(8.3) | 93.68(6.5) | 91.28(7.9) | 89.62(4.1) |
| 480 | Pyrimidifen | 91.57(5.6) | 94.15(8.5) | 93.25(8.5) | 96.58(9.7) | 99.67(8.5) | 102.6(7.6) | 96.35(8.2) | 94.15(4.2) | 93.25(3.1) | 95.26(7.2) | 93.86(5.4) | 90.25(6.1) |
| 481 | Acetamiprid | 86.23(6.3) | 88.47(7.5) | 93.25(5.2) | 91.58(7.5) | 94.58(4.3) | 96.84(7.4) | 88.35(8.4) | 85.23(4.7) | 86.33(6.2) | 88.36(8.6) | 85.32(4.3) | 84.27(2.2) |
| 482 | Butafenacil | 87.49(5.9) | 89.15(6.3) | 92.48(6.3) | 91.48(3.9) | 93.45(6.7) | 96.47(8.5) | 92.65(7.3) | 90.25(6.4) | 88.46(2.1) | 88.39(7.3) | 85.32(5.2) | 83.69(6.3) |
| 483 | Cafenstrole | 80.26(6.9) | 83.51(7.4) | 86.24(8.6) | N.D | N.D | N.D | N.D | N.D | N.D | N.D | N.D | N.D |
| 484 | Fluridone | 79.84(2.9) | 85.48(6.4) | 90.41(8.6) | 91.56(3.9) | 93.48(6.6) | 95.31(9.8) | 88.35(9.4) | 85.48(4.6) | 83.26(4.3) | 95.26(7.2) | 93.48(5.3) | 90.16(6.4) |
| 485 | Heptachlor-2,3-exo-epoxide | 69.84(2.9) | 75.48(6.4) | 90.41(8.6) | 81.56(3.9) | 83.48(6.6) | 90.87(9.8) | 98.75(9.8) | 95.63(7.0) | 92.38(4.6) | 104.58(7.3) | 106.78(8.6) | 99.37(5.3) |
| 486 | Methamidophos | 73.58(2.8) | 82.98(6.7) | 91.25(6.8) | 92.85(6.2) | 93.48(8.9) | 95.48(6.9) | 91.55(7.5) | 88.23(6.3) | 85.74(4.8) | 99.67(10.2) | 97.46(8.3) | 96.27(6.3) |
| 487 | Carbofuran | 81.54(7.1) | 83.47(8.5) | 85.26(8.8) | 84.72(5.6) | 80.25(3.9) | 88.94(2.9) | 99.65(8.2) | 96.37(6.6) | 92.76(4.0) | 108.54(6.3) | 105.23(4.3) | 99.67(2.2) |
| 488 | Acetamiprid | 74.89(5.6) | 77.67(7.5) | 81.76(10.2) | 75.48(2.7) | 81.64(4.5) | 88.45(8.4) | 99.65(8.6) | 94.52(6.1) | 92.46(4.9) | 92.48(9.6) | 90.27(7.5) | 87.54(5.3) |
| 489 | Trichlorfon | 82.78(3.9) | 85.47(6.7) | 88.48(6.4) | 85.45(4.8) | 90.48(6.7) | 94.87(7.4) | 72.55(6.9) | 76.58(8.1) | 79.63(5.3) | 69.38(4.6) | 75.22(6.5) | 78.23(8.5) |
| 490 | Demeton | N.D | N.D | N.D | N.D | N.D | N.D | N.D | N.D | N.D | N.D | N.D | N.D |
| 491 | Phorate sulfoxide | N.D | 70.57(4.8) | 75.68(3.9) | N.D | 72.48(6.4) | 81.24(4.2) | N.D | 88.57(11.2) | 90.28(9.0) | N.D | 85.21(7.5) | 86.93(5.4) |
| 492 | Oxycarboxin | 100.4(2.4) | 95.45(4.5) | 90.34(5.8) | 94.85(3.7) | 96.84(8.4) | 93.18(8.4) | 99.25(8.5) | 97.51(6.3) | 94.78(3.3) | 97.48(9.4) | 95.28(6.2) | 92.58(4.2) |
| 493 | Phoxim | 67.85(2.2) | 74.38(4.9) | 76.48(5.5) | 78.49(6.4) | 81.46(7.2) | 85.47(3.8) | 98.55(6.4) | 95.87(6.0) | 93.48(4.2) | 105.49(7.5) | 102.94(5.3) | 99.58(3.1) |
| 494 | Methoxyfenozide | 93.45(6.6) | 91.85(4.8) | 95.18(7.9) | 109.18(3.6) | 102.74(8.5) | 98.17(11.5) | 90.55(8.8) | 88.54(7.3) | 85.63(6.5) | 81.57(6.4) | 79.63(4.3) | 76.25(4.0) |
| 495 | Diafenthiuron | 76.85(4.4) | 83.15(6.4) | 86.49(8.8) | 85.17(3.2) | 82.14(5.5) | 88.74(6.8) | 90.55(6.4) | 87.45(5.6) | 85.21(2.3) | 95.63(8.4) | 91.38(6.4) | 89.46(5.2) |
| 496 | Thifensulfuron-methyl | 70.45(1.7) | 72.64(3.6) | 77.58(9.9) | 104.85(6.7) | 101.8(5.4) | 99.78(4.9) | 95.35(4.8) | 93.48(6.4) | 91.44(2.5) | 89.67(7.4) | 86.32(8.2) | 82.45(5.2) |
| 497 | Ethoxysulfuron | 100.54(2.9) | 97.54(8.4) | 103.45(5.5) | 104.56(3.8) | 108.41(6.7) | 99.78(4.5) | 104.5(6.4) | 101.54(5.1) | 98.72(4.3) | 96.38(5.5) | 98.62(7.1) | 94.62(7.2) |
| 498 | Spinosad | 105.2(5.6) | 103.87(6.4) | 98.47(6.4) | 68.45(3.6) | 73.48(5.8) | 79.58(6.9) | 99.65(8.5) | 97.24(7.5) | 94.25(3.8) | 97.46(2.6) | 95.38(4.9) | 92.76(2.5) |
| 499 | Mepiquat chloride | 85.47(4.6) | 86.74(7.5) | 91.28(6.9) | N.D | N.D | 80.45(6.3) | 96.35(6.5) | 93.25(5.5) | 88.45(7.2) | N.D | N.D | 74.21(9.0) |
| 500 | Tricyclazole | 83.47(5.1) | 81.5(7.2) | 85.49(10.2) | 93.46(5.4) | 95.87(6.2) | 90.45(9.7) | 98.65(8.5) | 96.53(6.4) | 92.48(5.7) | 92.46(8.6) | 89.63(7.2) | 87.52(5.3) |
| 501 | Isoproturon | 77.19(3.2) | 79.63(5.4) | 81.57(6.5) | 99.84(4.1) | 102.48(3.4) | 105.67(2.9) | 83.55(5.7) | 85.32(6.5) | 89.63(7.2) | 90.32(5.3) | 85.34(6.4) | 82.49(4.2) |
| 502 | Pymetrozine | 83.76(2.4) | 85.79(7.8) | 89.37(5.9) | 107.52(4.1) | 99.84(4.8) | 90.38(4.6) | 96.55(7.3) | 94.82(5.4) | 92.48(7.3) | 82.54(6.4) | 78.52(7.2) | 80.25(8.2) |
| 503 | Flumetsulam | 91.58(2.2) | 88.75(5.8) | 92.85(4.6) | 97.25(6.5) | 99.48(7.6) | 103.15(8.2) | 97.45(6.1) | 95.22(4.5) | 92.48(3.1) | 86.45(7.3) | 84.56(5.9) | 82.19(3.1) |
| 504 | Thiodicarb | 77.54(3.2) | 80.27(6.4) | 83.49(6.6) | 93.45(5.2) | 95.47(8.6) | 97.46(8.7) | 89.65(8.1) | 86.54(5.6) | 87.49(5.9) | 93.58(10.3) | 90.45(6.4) | 86.49(5.3) |
| 505 | Cinosulfuron | 93.48(5.6) | 97.45(3.8) | 95.76(6.3) | 75.41(6.2) | 77.85(6.7) | 80.16(4.9) | 107.5(8.5) | 102.45(7.3) | 96.58(9.2) | 94.85(10.5) | 92.48(8.3) | 89.62(7.1) |
| 506 | Pyrazosulfuron-ethyl | 74.89(6.8) | 77.84(5.6) | 80.39(5.4) | 76.35(4.5) | 83.56(7.5) | 89.99(10.6) | 99.65(6.4) | 96.57(8.2) | 94.18(3.5) | 78.62(6.4) | 80.59(7.2) | 75.62(9.4) |
| 507 | Methomyl | 110.54(6.7) | 112.25(3.8) | 105.89(5.4) | 93.58(5.4) | 95.87(8.4) | 96.74(2.5) | 89.65(9.0) | 90.58(6.4) | 85.49(6.3) | N.D | 75.84(9.4) | 73.49(6.4) |
| 508 | Cymoxanil | 72.48(6.4) | 75.84(7.9) | 79.62(12.3) | 89.54(5.9) | 87.62(5.6) | 85.64(3.8) | 95.65(8.3) | 93.48(7.2) | 90.21(2.7) | 62.54(7.1) | 68.95(6.3) | 73.54(8.5) |
| 509 | Omethoate | 120.45(10.6) | 110.58(5.4) | 112.85(6.9) | 93.45(5.4) | 96.47(6.7) | 95.74(10.5) | 123.5(12.6) | 120.58(7.5) | 118.45(6.3) | 83.96(8.1) | 81.49(6.1) | 79.63(5.2) |
| 510 | Ethoxyquin | 85.74(5.2) | 88.46(3.9) | 92.48(3.7) | 74.59(6.9) | 79.63(6.8) | 82.69(6.9) | 99.65(7.3) | 98.74(6.4) | 95.28(3.0) | 95.14(10.5) | 93.68(8.6) | 90.58(6.3) |
| 511 | Aldoxycarb | 96.87(6.5) | 99.78(6.4) | 100.52(9.7) | 55.49(10.6) | 59.74(6.7) | 63.48(2.9) | 90.55(6.4) | 88.54(5.3) | 86.31(7.4) | 94.68(5.8) | 90.46(4.2) | 88.23(3.7) |
| 512 | Imazapic | 94.87(6.4) | 97.58(6.9) | 90.48(8.4) | 106.38(6.4) | 98.54(4.6) | 95.78(12.6) | 98.65(8.7) | 96.34(5.9) | 92.15(3.0) | 97.64(5.2) | 96.31(2.6) | 92.34(5.2) |
| 513 | Uniconazole | 87.45(6.4) | 91.56(5.6) | 92.58(6.4) | 93.48(5.2) | 90.48(6.4) | 84.68(10.5) | 80.55(6.4) | 77.62(5.5) | 75.68(8.5) | 97.84(6.4) | 99.67(8.5) | 94.18(7.3) |
| 514 | Clofentezine | 65.48(5.9) | 70.59(4.85) | 74.12(6.9) | 76.48(5.8) | 79.64(6.8) | 82.59(10.6) | 88.65(6.4) | 86.54(4.6) | 84.27(5.3) | 104.98(7.3) | 101.58(6.3) | 97.46(4.9) |
| 515 | Vamidothion sulfone | 97.58(6.5) | 94.87(6.5) | 95.78(9.8) | 79.48(3.5) | 83.54(6.7) | 84.54(10.5) | 105.5(8.7) | 103.69(6.4) | 98.76(5.5) | 128.49(7.3) | 125.63(8.3) | 119.87(5.2) |
| 516 | Terbufos sulfone | 103.58(2.9) | 95.78(5.8) | 96.85(5.8) | 77.58(5.8) | 79.58(7.2) | 82.54(3.6) | 98.65(4.9) | 96.48(5.2) | 93.44(3.6) | 90.52(6.3) | 85.23(7.3) | 83.64(4.1) |
| 517 | Cyazofamid | 124.85(6.7) | 115.48(7.4) | 116.87(8.9) | 96.78(5.8) | 90.48(6.8) | 91.48(9.7) | 92.65(8.6) | 90.48(7.1) | 87.16(6.3) | 87.95(10.6) | 85.29(8.6) | 82.17(5.3) |
| 518 | Florasulam | 96.57(3.8) | 93.85(6.7) | 90.78(4.5) | 84.67(2.9) | 88.95(6.3) | 90.87(6.5) | 89.65(7.5) | 87.15(3.3) | 84.79(6.4) | 96.58(7.5) | 94.18(6.1) | 91.62(4.3) |
| 519 | Benzoximate | 78.56(6.8) | 80.24(6.4) | 81.96(7.9) | 94.85(5.7) | 92.54(6.4) | 85.47(6.4) | 97.85(8.7) | 93.67(7.6) | 90.48(5.3) | 88.67(6.4) | 85.29(5.2) | 83.94(4.2) |
| 520 | Chlormequat chloride | 84.74(2.8) | 88.54(6.7) | 90.62(3.7) | 100.45(3.8) | 95.74(6.7) | 103.85(10.2) | 95.65(9.6) | 92.18(7.5) | 91.58(3.7) | 87.44(10.3) | 89.62(5.5) | 92.67(7.2) |
| 521 | Sethoxydim | 75.48(6.4) | 79.64(9.6) | 81.49(10.3) | 94.87(15.4) | 92.58(5.5) | 97.58(8.8) | 107.5(8.5) | 103.64(7.2) | 98.67(5.2) | 93.64(7.6) | 91.48(4.0) | 88.27(4.7) |
| 522 | Folpet | 94.85(5.9) | 90.58(3.9) | 87.59(10.2) | 89.75(4.5) | 91.85(7.9) | 85.47(3.9) | 92.65(9.3) | 91.48(7.6) | 89.61(8.4) | 93.87(6.4) | 90.58(7.6) | 88.25(3.0) |
| 523 | Methiocarb | 85.48(5.4) | 86.49(6.4) | 88.74(10.5) | 57.84(10.6) | 65.48(5.4) | 71.58(5.8) | 95.45(3.6) | 93.76(2.7) | 90.81(5.0) | 92.36(11.3) | 90.25(8.4) | 88.27(6.3) |
| 524 | Cartap hydrochloride | 84.67(7.8) | 88.96(7.5) | 90.25(7.8) | 101.58(9.7) | 94.58(7.5) | 96.84(6.7) | 96.35(8.6) | 94.72(8.1) | 91.68(5.3) | 97.14(2.6) | 95.33(6.4) | 92.46(5.1) |
| 525 | Propyl phosphate | 91.25(3.2) | 90.59(6.8) | 96.78(6.5) | 89.63(7.2) | 90.56(5.6) | 95.36(5.2) | 94.85(3.7) | 91.57(4.3) | 90.87(4.0) | 89.64(8.3) | 92.46(10.3) | 93.67(7.1) |
| 526 | Triisobutyl phosphate | 96.87(3.25) | 94.56(6.8) | 98.78(6.8) | 73.89(6.3) | 77.85(4.2) | 75.68(4.2) | 97.15(4.6) | 94.12(2.1) | 95.67(2.5) | 97.84(7.6) | 95.34(5.1) | 93.08(3.1) |
| 527 | Tris(2-butoxyethyl) phosphate | 90.58(3.2) | 88.75(5.6) | 91.28(2.1) | 78.67(3.1) | 81.45(5.8) | 87.87(5.18) | 88.65(8.9) | 86.24(4.7) | 84.37(2.6) | 95.77(9.6) | 93.74(7.0) | 90.01(4.0) |
| 528 | 2-Ethylhexyldiphenyl phosphate | 79.56(4.2) | 81.25(4.2) | 85.68(2.7) | 89.89(3.4) | 91.56(3.8) | 93.78(3.2) | 93.05(7.1) | 92.60(5.0) | 90.66(4.0) | 89.46(8.0) | 87.55(6.1) | 85.23(1.1) |
| 529 | Tris(2-ethylhexyl) Phosphate | 68.78(5.8) | 72.58(6.9) | 70.25(3.2) | 81.56(3.8) | 85.78(6.3) | 88.58(3.8) | 89.85(8.2) | 87.55(6.3) | 85.94(3.5) | 87.69(6.3) | 85.31(5.1) | 83.66(3.1) |
| 530 | Tris(3-methylphenyl) phosphate | 82.58(23.6) | 85.68(17.5) | 86.78(14.2) | 95.68(17.2) | 91.25(14.5) | 90.87(6.8) | 104.5(6.5) | 101.66(5.8) | 95.67(2.5) | 77.66(12.5) | 80.06(9.8) | 83.44(7.4) |
| 531 | Tri-p-tolyl phosphate | 105.87(3.8) | 98.78(6.9) | 92.58(5.8) | 85.67(4.2) | 88.78(4.8) | 90.85(3.7) | 97.85(7.9) | 95.21(6.4) | 93.88(4.1) | 89.77(7.5) | 87.25(6.3) | 85.94(5.0) |
| 532 | Tris(2-methylphenyl) phosphate | 90.28(4.7) | 89.34(8.2) | 95.7(9.7) | 79.56(7.4) | 82.98(6.9) | 85.62(11.4) | 99.55(5.7) | 97.69(6.4) | 95.22(2.6) | 95.67(7.0) | 93.46(5.3) | 90.12(4.1) |
| 533 | Tris(1,3-dichloroisopropyl) phosphate | 98.78(4.2) | 95.87(2.7) | 90.78(7.8) | 81.8(6.8) | 79.83(9.7) | 88.28(6.2) | N.D | 75.69(13.5) | 78.25(9.8) | 99.46(9.3) | 96.45(7.0) | 95.67(4.5) |
| 534 | Cresyl diphenyl phosphate | 108.98(9.8) | 109.78(9.7) | 95.78(4.2) | 106.84(6.7) | 105.78(2.7) | 94.87(6.2) | 82.35(6.0) | 85.46(7.7) | 88.96(4.7) | 97.88(8.5) | 95.35(7.2) | 93.88(4.3) |
| 535 | Phenyl phosphate | 93.87(6.2) | 95.8(6.9) | 89.63(2.6) | 88.65(7.2) | 95.42(6.3) | 97.88(6.5) | 75.25(5.6) | 78.55(7.6) | 82.49(8.5) | 77.55(5.3) | 80.19(6.4) | 82.55(4.1) |
| 536 | Tris(1-Chloro-2-Propyl) Phosphate | 95.38(7.4) | 92.65(4.82) | 98.67(5.8) | 93.4(5.8) | 91.68(4.8) | 90.52(6.45) | 74.55(7.8) | 70.54(6.1) | 65.38(8.7) | 81.54(6.3) | 83.67(7.4) | 85.96(8.2) |
| 537 | Tributyl phosphate | 85.36(4.68) | 80.39(2.37) | 88.56(6.2) | 105.36(4.3) | 101.85(2.7) | 95.78(7.5) | 74.55(6.3) | 77.89(7.9) | 79.66(5.0) | 90.22(5.2) | 93.67(6.5) | 95.24(3.5) |
| 538 | tris(2-chloroethyl) phosphate | 96.8(4.2) | 95.78(5.96) | 98.63(7.8) | 107.5(7.8) | 106.8(5.1) | 92.69(6.8) | 97.55(6.0) | 95.64(6.4) | 93.65(2.5) | 72.54(3.5) | 75.86(5.2) | 77.24(7.6) |
| 539 | Triethyl phosphate | 83.65(12.9) | 84.23(7.3) | 87.62(6.89) | 85.21(2.6) | 88.32(6.01) | 93.58(2.63) | 97.85(10.2) | 95.74(8.4) | 92.46(6.4) | 97.88(8.5) | 95.62(5.1) | 93.77(4.2) |
| 540 | Trimethyl phosphate | 98.2(6.9) | 96.52(3.1) | 95.78(6.9) | 100.63(3.9) | 103.85(6.8) | 106.87(4.5) | 90.85(8.5) | 91.45(6.5) | 87.34(4.2) | 106.88(5.7) | 102.47(3.0) | 97.45(3.9) |
| 541 | Benzyl butyl phthalate | 94.38(2.5) | 97.35(6.1) | 98.38(5.8) | 87.56(3.4) | 88.76(4.8) | 95.73(5.1) | 98.55(12.4) | 96.45(9.3) | 94.27(7.1) | 95.21(8.2) | 93.15(6.3) | 89.67(1.3) |
| 542 | [Diphenyl phthalate](https://www.chemsrc.com/en/cas/84-62-8_510543.html) | 74.25(5.2) | 76.25(6.7) | 82.54(6.2) | 80.12(1.6) | 85.45(3.2) | 88.61(7.8) | 93.75(9.2) | 91.02(6.3) | 88.65(4.2) | 92.68(6.4) | 89.37(6.4) | 86.34(5.2) |
| 543 | [β-Butoxyethyl phthalate](https://www.chemsrc.com/en/cas/117-83-9_750429.html) | 86.21(2.9) | 88.32(5.6) | 90.23(4.9) | 95.21(2.8) | 97.25(3.8) | 100.7(8.9) | 96.85(7.5) | 93.67(4.3) | 90.28(3.2) | 96.37(4.6) | 94.21(2.3) | 91.76(3.5) |
| 544 | [Dibutyl phthalate](https://www.chemsrc.com/en/cas/84-74-2_336203.html) | 90.22(4.1) | 88.25(6.7) | 91.28(6.7) | 80.21(4.2) | 82.58(3.9) | 85.67(6.45) | 92.45(10.4) | 90.27(9.6) | 86.37(6.4) | 92.78(6.4) | 89.67(5.2) | 87.15(2.1) |
| 545 | [Dicyclohexyl phthalate](https://www.chemsrc.com/en/cas/84-61-7_670264.html) | 102.54(3.8) | 98.47(2.5) | 105.38(4.2) | 83.17(5.14) | 86.21(3.8) | 87.95(4.8) | 96.85(9.6) | 94.16(7.5) | 91.25(5.0) | 90.28(8.8) | 88.45(6.4) | 85.93(6.1) |
| 546 | [Dimethyl phthalate](https://www.chemsrc.com/en/cas/131-11-3_28935.html) | 95.21(3.8) | 93.58(5.8) | 90.58(6.9) | 89.75(3.8) | 85.64(6.2) | 83.85(5.6) | 115.5(13.7) | 109.87(10.3) | 106.52(8.3) | 94.25(5.3) | 92.46(3.5) | 89.73(2.2) |
| 547 | dipentyl phthalate | 103.56(3.5) | 98.86(4.5) | 90.58(6.9) | 75.46(2.8) | 77.85(4.8) | 82.69(5.9) | 87.45(8.3) | 85.24(6.3) | 83.48(2.6) | 85.26(7.2) | 84.22(5.5) | 81.76(8.6) |
| 548 | [Diethyl phthalate](https://www.chemsrc.com/en/cas/84-66-2_401979.html) | 89.36(1.2) | 92.58(5.2) | 95.48(6.4) | 108.36(5.6) | 103.85(6.5) | 106.87(10.4) | N.D | 70.21(15.3) | 72.14(11.2) | N.D | 110.25(13.7) | 109.57(10.3) |
| 549 | Diisobutyl phthalate | 72.56(6.2) | 76.38(6.8) | 82.35(6.8) | 85.67(6.9) | 84.38(9.7) | 87.69(6.7) | N.D | 79.65(7.1) | 80.54(8.2) | N.D | 105.64(7.6) | 108.25(5.2) |
| 550 | Dihexyl phthalate | 101.85(3.8) | 95.84(6.2) | 93.15(6.9) | 96.57(5.2) | 97.15(6.8) | 93.25(5.2) | 86.55(10.6) | 85.15(7.3) | 83.16(4.2) | 95.67(9.3) | 92.48(8.1) | 89.63(7.1) |
| 551 | Dioctyl phthalate | 93.68(4.9) | 92.18(6.2) | 90.18(7.8) | 82.15(5.1) | 86.18(7.9) | 90.16(6.4) | 87.55(8.3) | 85.37(6.5) | 82.13(6.1) | 88.59(7.9) | 86.21(7.0) | 83.69(5.5) |
| 552 | Bis(2-methoxyethyl) phthalate | 94.18(3.6) | 97.15(4.8) | 90.58(5.9) | 85.24(5.4) | 83.74(6.1) | 80.57(6.5) | 86.55(7.2) | 88.39(8.5) | 83.69(4.2) | 94.26(4.8) | 92.18(8.3) | 90.28(6.4) |
| 553 | bis(2-ethoxyethyl) benzene-1,2-dicarboxylate | 91.25(5.1) | 95.28(6.7) | 94.18(4.8) | 92.87(5.1) | 95.84(3.8) | 88.56(6.5) | 98.25(9.1) | 96.37(8.1) | 92.58(6.0) | 91.58(7.1) | 89.37(6.3) | 87.58(5.2) |
| 554 | 1,2-Benzenedicarboxylic acid, 1,2-bis(1,3-dimethylbutyl) ester | 87.65(2.4) | 88.96(6.8) | 92.54(5.2) | 79.85(4.1) | 81.95(6.4) | 88.47(8.4) | 77.85(10.3) | 75.49(8.8) | 73.46(4.3) | 96.84(11.3) | 94.18(8.6) | 90.87(6.3) |
| 555 | bis(2-ethylhexyl) phthalate | 94.85(3.5) | 96.87(4.9) | 90.78(6.9) | 80.21(3.9) | 84.25(4.8) | 88.97(10.5) | 90.55(7.5) | 92.78(8.3) | 94.78(4.2) | 106.59(8.3) | 104.27(5.4) | 101.29(3.5) |
| 556 | Dinonyl phthalate | 74.89(5.6) | 77.67(7.5) | 81.76(10.2) | 93.85(4.4) | 96.84(6.5) | 90.87(6.9) | 90.55(6.4) | 95.34(8.2) | 98.75(9.4) | 91.84(8.6) | 89.67(7.2) | 85.24(6.0) |
| 557 | N-Sulfocarbamoylgonyautoxin-2 | 83.25(6.2) | 78.25(6.3) | 85.21(8.7) | 92.76(8.4) | 96.38(7.5) | 98.26(7.1) | 90.55(6.2) | 93.74(9.2) | 95.78(6.4) | 88.57(7.2) | 90.46(8.3) | 93.47(8.2) |
| 558 | N-Sulfocarbamoylgonyautoxin-3 | 85.24(6.3) | 88.45(9.7) | 93.48(12.7) | 93.85(4.6) | 96.84(6.7) | 94.58(7.5) | 88.25(9.1) | 92.48(7.2) | 93.47(6.4) | 95.48(8.2) | 100.52(7.5) | 102.48(3.5) |
| 559 | Decarbamovlgonyautoxin-2 | 89.2(14.2) | 93.55(7.6) | 87.21(6.1) | 72.35(3.4) | 76.87(3.8) | 80.59(7.1) | 82.55(3.9) | 89.45(7.2) | 93.41(7.1) | 82.56(8.7) | 88.12(4.9) | 93.68(7.2) |
| 560 | Decarbamovlgonyautoxin-3 | 79.62(4.1) | 83.64(7.1) | 92.54(6.3) | 86.34(8.2) | 90.47(5.3) | 93.48(4.5) | 87.25(6.4) | 89.67(3.4) | 93.87(6.3) | 92.48(3.6) | 95.47(6.8) | 91.45(5.3) |
| 561 | Decarbamoylneosaxitoxin dihydrochloride | 83.65(5.2) | 86.57(6.3) | 89.67(7.4) | 92.46(5.5) | 95.67(8.2) | 97.46(10.6) | 88.55(8.6) | 90.48(7.2) | 92.40(8.4) | 78.59(6.1) | 82.46(6.3) | 86.25(10.5) |
| 562 | Decarbamoylsaxitoxin | 86.32(5.8) | 87.24(6.4) | 92.15(9.3) | 88.75(3.6) | 84.12(4.1) | 89.36(5.6) | 93.15(7.5) | 96.32(6.9) | 99.58(8.2) | 91.25(8.6) | 92.54(7.1) | 96.35(8.7) |
| 563 | Gonyautoxin-1 | 88.63(5.4) | 91.35(6.4) | 95.87(8.2) | 82.46(6.1) | 87.35(5.4) | 90.78(4.1) | 88.25(11.3) | 92.74(8.7) | 95.48(7.2) | 97.25(6.4) | 93.74(7.4) | 96.83(6.4) |
| 564 | Gonyautoxin-4 | 77.25(6.3) | 88.25(6.3) | 96.25(6.2) | 88.76(8.4) | 85.14(6.3) | 81.75(4.2) | 96.35(6.5) | 98.74(5.2) | 91.58(4.2) | 88.62(6.3) | 84.57(8.1) | 92.68(6.3) |
| 565 | Gonyautoxin-2 | 87.12(6.3) | 96.58(7.4) | 92.58(6.3) | 88.36(6.7) | 90.56(5.4) | 94.26(8.9) | 78.65(8.6) | 82.54(6.6) | 88.65(7.3) | 94.68(6.3) | 98.74(8.2) | 94.18(4.3) |
| 566 | Gonyautoxin-3 | 73.58(8.2) | 82.98(9.3) | 91.25(4.2) | 86.32(6.2) | 88.66(7.6) | 93.48(8.3) | 95.45(9.2) | 88.23(6.3) | 85.74(4.6) | 93.12(8.8) | 95.28(7.2) | 93.64(6.5) |
| 567 | Gonyautoxin-6 | 88.62(6.3) | 92.87(7.4) | 95.84(8.3) | 88.67(6.2) | 95.74(8.1) | 97.68(9.6) | 89.55(7.3) | 90.14(9.6) | 84.71(6.3) | 92.54(7.4) | 87.49(6.6) | 83.21(6.4) |
| 568 | Neosaxitoxin | 75.26(6.1) | 78.95(8.2) | 81.54(5.2) | 82.15(7.2) | 86.93(4.5) | 89.67(6.3) | 93.65(6.4) | 95.17(8.3) | 92.48(7.1) | 96.84(8.1) | 94.28(6.3) | 90.58(7.1) |
| 569 | Saxitoxin dihydrochloride | 83.25(7.1) | 85.69(8.2) | 88.63(9.3) | 90.15(6.1) | 92.84(8.1) | 96.57(7.4) | 85.45(3.6) | 88.69(7.4) | 90.25(7.3) | 102.58(8.4) | 105.47(7.6) | 97.46(9.6) |
| 570 | Tetrodotoxin | 78.69(2.4) | 80.15(2.6) | 82.45(6.8) | 91.85(3.5) | 92.48(6.4) | 96.58(6.8) | 94.55(7.2) | 93.48(5.3) | 90.78(2.5) | 80.49(6.5) | 82.49(5.2) | 86.97(3.2) |
| 571 | Microcystin RR | 92.84(4.5) | 92.15(4.7) | 88.15(5.3) | 82.83(3.8) | 85.41(4.5) | 90.28(6.1) | 91.55(5.6) | 88.54(4.9) | 85.27(2.1) | 95.87(7.5) | 93.18(5.1) | 90.48(3.5) |
| 572 | Microcystin LR | 72.54(6.2) | 75.14(5.4) | 79.15(6.8) | 93.18(3.8) | 95.48(5.2) | 98.21(6.5) | 102.5(6.5) | 100.54(4.9) | 97.63(2.9) | 96.88(4.2) | 94.25(3.1) | 90.18(1.6) |
| 573 | Okadaic Acid | 93.15(3.9) | 95.87(5.2) | 97.65(7.2) | 81.96(5.2) | 85.48(6.8) | 87.97(8.8) | N.D | 79.63(10.5) | 77.52(6.5) | N.D | 110.58(3.1) | 115.47(1.9) |
| 574 | Nodularin | 75.25(14.9) | 78.49(16.2) | 85.47(6.8) | 81.96(6.2) | 84.65(6.8) | 88.96(8.6) | 88.35(5.5) | 86.35(4.8) | 84.16(2.8) | 97.28(7.2) | 94.52(5.3) | 92.46(3.1) |
| 575 | Sulfabenzamide | 92.56(2.1) | 89.36(7.8) | 85.16(2.6) | 78.64(8.2) | 95.31(7.9) | 98.64(8.1) | 110.5(5.9) | 105.27(4.2) | 99.78(2.6) | 98.45(6.3) | 94.78(6.5) | 90.75(5.1) |
| 576 | Sulfadiazine | 96.25(5.7) | 93.26(5.8) | 88.35(6.5) | 90.16(5.6) | 88.56(6.0) | 85.32(8.7) | 93.85(6.3) | 90.58(5.4) | 86.21(3.3) | 89.51(6.3) | 88.14(5.2) | 83.49(4.2) |
| 577 | Sulfadimethoxine | 98.56(4.1) | 96.34(3.7) | 88.76(7.2) | 87.52(3.8) | 90.23(6.5) | 84.36(5.8) | 87.15(7.0) | 85.24(5.9) | 80.17(3.8) | 85.21(8.6) | 82.19(7.6) | 78.51(6.9) |
| 578 | Sulfamethazine | 87.63(7.9) | 95.34(5.6) | 87.36(8.5) | 93.25(6.3) | 96.87(4.9) | 89.76(9.6) | 108.5(4.0) | 104.57(3.0) | 100.24(1.8) | 102.54(6.5) | 98.47(5.5) | 94.21(3.5) |
| 579 | Sulfadoxine | 86.37(6.7) | 90.78(7.8) | 87.26(7.8) | 105.89(6.8) | 105.96(10.2) | 103.02(6.8) | 105.5(6.5) | 100.24(5.5) | 94.18(4.6) | 97.45(8.2) | 95.48(6.2) | 93.15(5.2) |
| 580 | Sulfamerazine | 95.27(1.8) | 90.78(7.8) | 100.2(6.7) | 85.23(3.1) | 84.2(7.3) | 75.96(11.5) | 82.55(6.0) | 80.47(5.0) | 75.49(3.2) | 87.19(7.0) | 84.29(6.5) | 80.47(4.3) |
| 581 | Sulfamethizole | 88.46(5.2) | 93.26(6.1) | 88.35(6.7) | 88.36(4.5) | 90.56(2.8) | 94.26(3.5) | 103.5(7.0) | 100.58(6.9) | 98.57(5.4) | 97.54(3.6) | 95.21(5.4) | 90.24(6.0) |
| 582 | Sulfamethoxypyridazine | 75.49(5.8) | 80.25(7.2) | 83.49(6.1) | 76.59(5.2) | 72.56(7.3) | 78.69(6.4) | 98.55(4.4) | 97.18(2.7) | 90.54(6.9) | 93.65(10.3) | 90.57(8.6) | 84.72(4.2) |
| 583 | Sulfaphenazole | 76.35(1.4) | 80.36(3.7) | 84.57(8.6) | 94.89(3.8) | 96.85(7.2) | 103.84(9.6) | 115.5(4.9) | 110.87(6.0) | 107.65(3.7) | 107.89(7.0) | 103.48(8.6) | 100.57(5.3) |
| 584 | Sulfapyrazole | 78.65(6.8) | 80.35(7.2) | 87.12(9.2) | 94.36(1.6) | 92.8(6.1) | 87.24(4.1) | 86.25(7.0) | 85.17(5.6) | 82.79(4.6) | 102.54(5.3) | 100.25(7.9) | 85.16(3.7) |
| 585 | Sulfapyridine | 100.2(4.2) | 97.58(3.7) | 93.87(5.7) | 74.81(2.1) | 76.87(3.8) | 80.59(5.9) | 108.5(8.7) | 105.49(6.0) | 102.54(3.6) | 112.54(8.5) | 110.85(6.4) | 102.54(4.3) |
| 586 | Sulfaquinoxaline | 79.68(5.4) | 82.57(6.8) | 95.48(8.4) | 91.58(2.8) | 88.64(3.9) | 86.19(3.8) | 88.45(7.0) | 85.14(9.6) | 83.18(5.2) | 87.19(5.1) | 89.64(8.4) | 83.64(4.7) |
| 587 | Sulfathiazole | 94.87(5.1) | 90.24(6.5) | 88.75(10.2) | 70.28(4.6) | 73.85(6.4) | 80.21(12.6) | 108.5(8.0) | 105.24(4.9) | 98.62(7.5) | 88.64(4.8) | 85.47(6.0) | 82.19(2.3) |
| 588 | Sulfisomidine | 90.54(6.4) | 92.54(6.8) | 95.48(8.7) | 89.51(2.9) | 92.84(3.8) | 99.57(6.8) | 95.15(6.0) | 98.14(4.9) | 100.48(1.5) | 85.17(7.0) | 82.17(4.3) | 85.49(2.3) |
| 589 | Trimethoprim | 82.54(6.5) | 84.67(7.9) | 89.54(6.2) | 103.85(6.5) | 100.85(7.9) | 94.86(10.8) | 100.5(8.3) | 95.47(6.2) | 98.45(3.5) | 100.54(10.3) | 95.28(7.5) | 91.48(6.3) |
| 590 | Cinoxacin | 92.58(3.5) | 88.14(2.8) | 86.14(7.6) | 95.48(5.8) | 97.28(8.5) | 92.54(11.8) | 89.55(8.5) | 90.14(7.6) | 82.15(8.1) | 96.18(11.3) | 91.74(8.5) | 88.17(5.1) |
| 591 | Danofloxacin | 76.48(6.5) | 80.15(7.5) | 82.75(10.5) | 79.54(4.1) | 83.18(5.6) | 86.94(6.8) | 82.55(8.6) | 80.47(6.3) | 78.96(4.6) | 113.25(8.6) | 110.48(6.1) | 106.49(7.3) |
| 592 | Difloxacin | 110.8(3.8) | 105.87(4.9) | 99.87(8.6) | 110.58(3.6) | 100.58(6.9) | 95.48(7.9) | 113.5(9.0) | 110.69(7.6) | 106.28(5.3) | 97.84(6.5) | 95.16(3.5) | 91.87(2.5) |
| 593 | Enrofloxacin | 92.85(4.8) | 90.15(5.3) | 88.14(6.9) | 83.15(2.9) | 85.75(3.9) | 88.47(14.6) | 88.55(5.3) | 85.14(4.0) | 80.57(2.6) | 106.49(11.3) | 101.54(8.6) | 98.47(5.2) |
| 594 | Flumequine | 77.56(6.1) | 80.52(3.9) | 83.15(10.2) | 108.45(3.6) | 102.57(5.8) | 94.87(11.2) | 96.85(7.5) | 94.18(5.3) | 90.18(1.3) | 96.85(4.8) | 92.84(3.7) | 90.36(2.2) |
| 595 | Gatifloxacin | 80.35(2.9) | 88.37(6.4) | 94.28(6.9) | 95.18(5.6) | 96.47(6.4) | 90.18(8.8) | 83.45(6.2) | 81.54(7.7) | 78.36(5.0) | 102.58(9.7) | 97.48(5.5) | 95.17(3.2) |
| 596 | Lomefloxacin | 90.18(6.4) | 92.85(8.5) | 88.51(6.5) | 124.18(5.6) | 118.25(6.4) | 108.61(6.9) | 102.5(12.5) | 100.25(9.6) | 97.16(7.3) | 98.47(8.6) | 95.18(9.5) | 93.46(6.8) |
| 597 | Marbofloxacin | 66.28(7.5) | 72.18(4.6) | 75.48(6.4) | 89.56(3.9) | 92.48(7.7) | 95.18(13.5) | 111.5(6.8) | 108.49(6.1) | 111.02(5.3) | 104.59(9.6) | 102.58(8.6) | 98.16(7.3) |
| 598 | Moxifloxacin | 71.68(4.8) | 75.68(6.5) | 77.86(9.2) | 94.85(4.8) | 96.87(6.5) | 97.56(3.2) | 88.15(8.5) | 87.19(7.2) | 85.24(6.3) | 102.54(10.5) | 98.17(8.5) | 95.21(6.3) |
| 599 | Nalidixic acid | 81.46(5.4) | 82.59(4.4) | 87.59(8.2) | 103.48(4.4) | 105.68(8.9) | 95.48(13.5) | 102.5(9.7) | 98.49(6.3) | 95.12(5.2) | 97.49(8.1) | 95.42(7.6) | 90.68(3.3) |
| 600 | Ofloxacin | 76.85(6.9) | 79.65(4.5) | 81.57(8.6) | 93.86(4.5) | 95.87(7.2) | 95.85(13.8) | 103.5(6.2) | 100.54(5.9) | 95.17(2.0) | 100.25(7.5) | 94.28(6.2) | 95.17(4.2) |
| 601 | Orbifloxacin | 81.59(7.8) | 85.96(6.9) | 88.49(13.6) | 76.25(4.4) | 77.69(7.8) | 74.16(9.9) | 103.5(6.1) | 100.28(5.7) | 98.17(4.3) | 91.87(8.5) | 88.65(7.2) | 85.17(8.2) |
| 602 | Oxolinic acid | 94.21(7.5) | 93.58(6.2) | 90.18(6.8) | 102.58(7.7) | 106.58(6.9) | 96.28(8.6) | 85.95(4.2) | 82.47(6.2) | 80.16(3.5) | 96.27(6.3) | 92.78(7.7) | 90.18(4.9) |
| 603 | Sarafloxacin | 78.95(8.9) | 81.26(6.5) | 83.14(8.6) | 96.85(4.5) | 95.48(4.4) | 98.14(8.1) | 93.15(10.3) | 88.17(8.2) | 86.14(6.2) | 101.58(8.5) | 97.15(9.4) | 95.18(7.3) |
| 604 | Sparfloxacin | 63.54(2.9) | 65.48(7.1) | 68.94(8.4) | N.D | 60.58(9.8) | 62.74(10.8) | 88.65(8.7) | 85.24(4.7) | 83.21(2.7) | N.D | 60.58(9.8) | 62.74(10.8) |
| 605 | Tosufloxacin | 77.56(6.1) | 80.52(3.9) | 83.15(10.2) | 108.45(3.6) | 102.57(5.8) | 94.87(11.2) | 95.25(9.3) | 94.12(12.5) | 90.18(8.5) | 108.45(4.5) | 102.57(2.7) | 94.87(4.9) |
| 606 | Albendazole | 96.47(3.9) | 95.48(3.1) | 90.87(7.4) | 83.57(5.1) | 85.96(7.2) | 90.89(8.5) | 95.65(5.6) | 93.78(2.7) | 90.28(2.6) | 103.58(8.3) | 100.24(6.2) | 98.14(3.7) |
| 607 | Albendazole-2-aminosulfone | 77.56(6.1) | 80.52(3.9) | 83.15(10.2) | 108.45(3.6) | 102.57(5.8) | 94.87(11.2) | 81.55(7.3) | 82.94(6.4) | 79.36(4.1) | 103.58(7.2) | 99.58(6.5) | 96.23(5.2) |
| 608 | Albendazole sulfoxide | 124.58(5.4) | 126.47(8.9) | 130.45(6.7) | 94.67(5.9) | 96.85(8.5) | 90.85(12.6) | 116.5(7.3) | 114.28(5.4) | 109.48(7.3) | 99.25(7.5) | 97.16(6.3) | 95.21(5.3) |
| 609 | Mebendazole-amine | 83.14(2.2) | 85.94(6.5) | 88.45(9.8) | 74.56(4.1) | 77.26(6.3) | 79.15(8.9) | 89.65(8.3) | 87.14(7.3) | 85.21(6.3) | 87.54(10.3) | 85.26(9.4) | 83.19(7.2) |
| 610 | Thiabendazole | 74.19(6.5) | 76.98(8.2) | 83.15(10.2) | 72.59(8.2) | 75.49(6.3) | 77.68(9.5) | 80.25(7.6) | 78.25(6.4) | 75.26(6.0) | 86.25(9.4) | 84.19(7.3) | 82.49(5.3) |
| 611 | Dimetridazole | 92.58(6.5) | 95.84(11.2) | 99.58(6.8) | 84.57(2.6) | 87.19(5.6) | 90.28(6.8) | 93.55(7.6) | 90.58(6.2) | 88.16(4.2) | 97.58(6.6) | 94.16(6.3) | 92.87(4.3) |
| 612 | Fenbendazole | 87.49(6.5) | 88.59(2.5) | 91.49(3.6) | 72.58(10.3) | 79.65(6.4) | 82.54(7.9) | 92.85(6.4) | 93.48(7.3) | 89.18(4.6) | 86.25(9.7) | 84.12(7.3) | 83.19(3.5) |
| 613 | Flubendazole | 71.59(13.5) | 78.59(6.8) | 79.18(6.5) | 64.89(5.5) | 67.49(9.5) | 72.15(8.8) | 78.65(9.4) | 76.28(4.7) | 76.32(6.0) | 86.32(7.0) | 87.14(5.2) | 83.49(4.4) |
| 614 | Hydroxy ipronidazole | 74.85(3.1) | 77.86(5.2) | 80.91(4.9) | 79.81(3.9) | 82.45(6.2) | 90.65(8.9) | 88.25(10.4) | 85.47(8.2) | 83.16(6.4) | 92.58(7.7) | 90.25(6.4) | 88.54(5.3) |
| 615 | Ipronidazole | 89.75(4.4) | 92.84(5.5) | 93.79(6.2) | 64.57(2.2) | 68.94(8.5) | 74.96(12.3) | 93.55(11.3) | 91.46(8.6) | 88.26(7.1) | 78.96(9.7) | 77.25(8.3) | 74.21(6.3) |
| 616 | Mebendazole | 71.49(10.3) | 73.69(8.5) | 84.67(15.2) | 84.32(5.2) | 85.29(3.6) | 88.96(7.5) | 78.25(9.4) | 76.25(6.1) | 75.21(3.7) | 93.68(7.5) | 91.58(4.3) | 88.24(3.2) |
| 617 | Cambendazole | 80.26(4.4) | 83.65(4.6) | 88.74(5.6) | 71.56(4.3) | 77.86(6.8) | 79.23(10.5) | 85.15(8.3) | 82.15(7.4) | 80.11(4.3) | 78.62(11.4) | 76.21(8.3) | 74.11(5.6) |
| 618 | 5-Hydroxythiabendazole | 77.53(4.6) | 82.59(6.4) | 85.79(4.9) | 77.49(5.5) | 79.62(8.6) | 82.69(6.7) | 77.55(8.6) | 82.59(7.2) | 85.79(5.4) | 89.36(8.3) | 87.16(7.2) | 85.15(5.6) |
| 619 | Levamisole | 89.65(2.3) | 84.21(7.8) | 82.64(11.6) | 90.36(4.5) | 88.37(4.6) | 85.64(10.5) | 89.25(6.4) | 87.19(6.9) | 84.15(5.2) | 92.84(8.5) | 91.87(6.3) | 88.16(4.3) |
| 620 | Metronidazole | 71.46(3.8) | 72.49(5.5) | 78.63(4.8) | 74.59(5.5) | 82.59(6.6) | 88.14(4.6) | 77.25(6.6) | 78.26(6.3) | 80.59(5.7) | 87.19(7.6) | 85.21(8.4) | 86.22(3.3) |
| 621 | Oxfendazole | 56.32(10.8) | 65.23(7.8) | 68.25(7.5) | 90.64(7.2) | 94.56(6.2) | 102.56(5.9) | 89.25(6.3) | 87.25(6.3) | 84.16(3.0) | 91.58(8.3) | 88.25(4.7) | 86.24(4.0) |
| 622 | Oxibendazole | 64.28(2.9) | 66.49(5.9) | 71.24(5.4) | 100.25(4.1) | 105.6(6.5) | 107.2(8.4) | 74.25(6.4) | 73.26(6.0) | 70.68(7.3) | 87.49(6.4) | 85.24(5.6) | 81.29(2.6) |
| 623 | Ronidazole | 79.52(4.4) | 83.16(5.1) | 86.15(11.5) | 90.25(6.3) | 92.08(3.9) | 95.78(6.5) | 88.55(12.6) | 86.59(10.3) | 84.16(7.3) | 87.65(8.6) | 85.24(7.4) | 83.19(4.6) |
| 624 | Secnidazole | 89.16(4.5) | 90.25(5.6) | 92.84(7.6) | 92.84(7.8) | 94.68(6.9) | 93.48(8.4) | 94.55(13.3) | 92.58(10.4) | 88.74(8.6) | 96.25(8.7) | 94.18(7.3) | 93.16(5.3) |
| 625 | Tinidazole | 87.16(6.3) | 89.17(8.4) | 93.16(5.6) | 90.56(4.4) | 92.58(6.7) | 93.76(4.8) | 88.25(6.4) | 89.36(7.2) | 85.26(4.2) | 99.25(7.6) | 97.26(5.3) | 95.26(3.1) |
| 626 | Triclabendazole | 81.5(4.5) | 83.97(5.9) | 85.94(11.2) | 83.64(5.6) | 85.74(6.5) | 88.78(8.2) | 87.55(10.2) | 86.26(8.6) | 84.16(7.2) | 105.26(8.4) | 102.89(6.3) | 100.59(4.3) |
| 627 | Clindamycin | 85.62(3.2) | 87.36(5.4) | 90.31(5.9) | 90.85(4.4) | 93.58(4.6) | 96.31(8.1) | 96.25(13.6) | 94.28(8.5) | 91.78(6.3) | 83.47(9.6) | 81.25(7.6) | 79.85(4.9) |
| 628 | Doramectin | 89.63(4.5) | 92.58(8.2) | 94.15(10.5) | 74.25(5.2) | 75.68(8.5) | 77.16(9.3) | 98.55(12.6) | 95.47(9.2) | 92.18(8.3) | 88.25(9.0) | 85.24(6.4) | 83.49(5.3) |
| 629 | Eprinomectin | 76.25(4.1) | 79.65(6.4) | 82.54(7.2) | 71.54(8.5) | 74.36(6.9) | 77.15(8.5) | N.D | 70.58(9.6) | 73.84(6.4) | N.D | 75.29(8.6) | 77.19(4.6) |
| 630 | Ivermectin | 71.54(4.1) | 73.54(8.2) | 76.59(6.8) | 74.52(5.1) | 77.58(8.2) | 79.61(4.8) | 88.55(8.5) | 85.21(7.3) | 83.19(5.4) | N.D | 75.89(8.5) | 72.49(6.5) |
| 631 | Leucomycin A1 | 73.54(3.1) | 74.15(4.2) | 76.19(8.9) | 86.52(4.5) | 88.65(5.2) | 90.16(3.8) | 78.55(8.6) | 76.24(6.3) | 75.18(5.4) | 78.62(9.7) | 76.28(8.2) | 77.28(4.1) |
| 632 | Spiramycin | 90.58(4.2) | 92.54(5.5) | 95.68(8.1) | 89.57(4.7) | 92.54(6.3) | 95.87(4.6) | 106.5(5.2) | 102.87(4.2) | 98.57(3.0) | 92.85(8.8) | 89.62(7.5) | 87.14(5.2) |
| 633 | Tilmicosin | 84.52(4.1) | 88.75(5.4) | 85.63(8.4) | 93.58(8.3) | 95.48(6.7) | 88.64(4.9) | 98.25(3.2) | 96.52(7.4) | 94.18(6.3) | 98.25(7.5) | 96.28(6.4) | 94.18(5.7) |
| 634 | Tylosin | 91.68(3.1) | 95.64(7.2) | 97.48(8.1) | 101.25(5.4) | 105.64(6.4) | 107.48(8.1) | 88.55(9.7) | 87.19(8.3) | 84.19(5.3) | 102.54(8.3) | 98.47(7.2) | 94.28(6.4) |
| 635 | Virginiamycin M1 | 85.41(4.5) | 86.49(3.8) | 89.61(8.7) | 83.54(5.5) | 85.67(6.9) | 89.76(8.4) | 97.55(7.0) | 95.14(5.3) | 93.48(2.7) | 87.51(8.5) | 85.17(6.3) | 83.17(6.0) |
| 636 | Beclomethasone | 76.95(8.4) | 78.63(6.4) | 81.54(10.6) | 77.31(4.6) | 79.64(8.4) | 83.96(4.9) | 88.75(10.2) | 87.15(6.4) | 85.24(6.3) | 97.15(12.4) | 95.24(10.3) | 92.87(8.4) |
| 637 | Beclomethasone dipropionate | 87.63(5.1) | 89.76(6.4) | 91.78(12.5) | 84.67(8.1) | 86.94(9.3) | 90.48(6.4) | 88.55(7.5) | 86.32(5.0) | 84.19(2.7) | 106.58(9.0) | 102.84(7.4) | 98.75(6.2) |
| 638 | Betamethasone dipropionate | 75.12(3.2) | 77.49(8.1) | 80.49(4.9) | 86.35(10.2) | 87.49(8.6) | 90.38(11.5) | 85.25(7.3) | 83.97(6.3) | 81.75(4.3) | 98.54(11.4) | 96.28(8.4) | 94.17(5.3) |
| 639 | Betamethasone valerate | 93.58(6.4) | 90.48(7.4) | 89.65(5.3) | 90.25(6.1) | 92.48(6.4) | 94.25(6.1) | 95.85(7.6) | 93.47(6.4) | 91.48(2.0) | 88.96(11.5) | 86.29(9.1) | 84.75(8.2) |
| 640 | Chlormadinone acetate | 89.62(4.1) | 90.56(4.6) | 96.23(4.8) | 83.52(4.7) | 86.34(7.3) | 88.94(6.2) | 92.85(8.4) | 93.89(9.3) | 90.28(4.1) | 92.68(5.2) | 90.85(6.3) | 87.48(6.2) |
| 641 | Clobetasol 17- propionate | 83.59(3.1) | 85.94(6.8) | 88.63(8.1) | 73.46(2.3) | 75.86(5.7) | 78.64(8.6) | 89.45(8.2) | 87.14(6.3) | 85.19(7.5) | 70.29(6.8) | 74.81(6.2) | 76.98(3.3) |
| 642 | Clobetasone butyrate | 86.52(5.1) | 88.67(7.5) | 90.54(8.3) | 88.56(10.2) | 92.48(6.1) | 93.87(8.4) | 98.55(10.2) | 95.26(6.4) | 92.78(5.2) | 94.15(7.3) | 92.58(6.5) | 90.28(3.6) |
| 643 | Cortisone | 87.54(2.4) | 89.63(5.6) | 93.76(8.2) | 92.58(7.1) | 93.18(6.4) | 95.76(8.6) | 82.55(6.0) | 80.19(4.7) | 77.49(1.7) | 90.25(15.3) | 87.49(8.6) | 85.19(7.2) |
| 644 | Deflazacort | 86.35(4.2) | 88.64(5.3) | 90.78(11.3) | 82.59(11.4) | 85.67(4.3) | 86.94(10.5) | 94.85(5.2) | 91.28(4.3) | 90.18(2.2) | 80.96(11.9) | 82.59(9.2) | 77.19(1.9) |
| 645 | Dexamethasone | 75.32(8.8) | 81.24(6.9) | 86.94(7.8) | 86.35(6.2) | 87.49(8.3) | 90.65(8.9) | 113.5(7.6) | 108.49(6.3) | 102.84(3.7) | 95.48(8.4) | 93.38(7.2) | 90.29(6.4) |
| 646 | Diflorasone Diacetate | 84.71(3.2) | 86.59(7.4) | 88.59(8.1) | 84.21(3.5) | 86.49(6.7) | 89.65(8.6) | 93.15(8.2) | 92.18(7.1) | 89.62(4.2) | 87.49(9.4) | 85.19(7.3) | 83.27(5.1) |
| 647 | Epitestosterone | 89.64(5.5) | 92.48(6.2) | 93.48(2.3) | 75.26(4.9) | 77.68(8.4) | 80.21(6.2) | 88.25(4.9) | 85.23(7.6) | 83.59(8.7) | 82.59(8.5) | 80.51(6.3) | 77.26(4.1) |
| 648 | Fludrocortisone | 77.59(4.2) | 79.65(6.8) | 80.14(6.7) | 77.59(4.3) | 79.65(8.1) | 83.49(6.9) | 85.25(9.5) | 88.49(7.3) | 82.17(3.4) | 80.97(6.8) | 78.94(8.5) | 75.19(7.3) |
| 649 | Flumethasone | 82.49(8.1) | 86.14(9.3) | 89.49(8.5) | 74.85(5.2) | 76.83(7.6) | 79.65(11.6) | 93.45(13.3) | 90.57(11.3) | 88.29(5.3) | 73.58(9.3) | 75.89(8.1) | 71.26(5.2) |
| 650 | Flumethasone pivalate | 68.27(3.1) | 72.59(5.7) | 75.46(6.8) | 80.23(15.9) | 83.46(17.5) | 86.21(6.9) | 103.5(5.9) | 99.54(5.2) | 98.18(4.3) | 88.52(9.6) | 86.57(6.4) | 85.12(4.3) |
| 651 | Fluocinolone acetonide | 83.54(5.2) | 86.14(7.3) | 88.15(8.2) | 79.62(6.6) | 82.35(5.4) | 83.56(8.7) | 82.55(8.6) | 81.74(7.3) | 79.52(6.3) | 78.21(4.7) | 77.19(5.3) | 75.84(3.2) |
| 652 | Flurandrenolide | 87.21(3.2) | 89.63(5.2) | 91.46(6.8) | 84.24(6.2) | 86.54(7.7) | 88.95(7.9) | 87.15(13.7) | 85.29(9.9) | 83.29(7.1) | 89.52(9.2) | 87.15(8.3) | 86.29(7.0) |
| 653 | Fluoromethalone | 92.58(6.4) | 95.68(7.5) | 96.24(9.6) | 104.3(4.5) | 103.56(7.8) | 99.65(4.2) | 99.65(8.3) | 97.18(6.2) | 95.28(5.3) | 98.18(4.6) | 96.74(3.6) | 93.44(2.6) |
| 654 | Fluticasone propionate | 76.59(6.3) | 80.35(8.2) | 81.76(9.5) | 84.25(7.3) | 86.79(8.5) | 88.59(8.4) | 108.5(12.7) | 105.24(8.6) | 99.57(8.1) | 106.28(9.6) | 104.78(7.3) | 99.65(5.3) |
| 655 | Halcinonide | 72.58(4.6) | 75.86(1.3) | 79.63(12.5) | 87.32(4.5) | 89.59(7.5) | 92.78(4.8) | 85.15(7.6) | 88.96(5.2) | 90.51(3.3) | 91.87(8.3) | 89.47(6.2) | 86.59(4.7) |
| 656 | Hydrocortisone | 75.36(5.2) | 77.89(6.8) | 80.32(10.3) | 93.68(6.5) | 95.87(7.5) | 100.3(8.4) | 93.55(4.2) | 91.48(5.2) | 90.68(2.7) | 73.96(7.9) | 72.49(6.4) | 70.12(5.3) |
| 657 | Megestrol | 85.36(6.6) | 87.25(6.7) | 90.37(12.6) | 93.85(8.5) | 96.78(4.9) | 100.54(7.9) | 102.5(8.2) | 100.48(7.5) | 96.24(5.7) | 86.24(8.2) | 84.19(7.5) | 82.59(6.3) |
| 658 | Melengestrol acetate | 84.23(4.2) | 88.59(5.2) | 90.84(7.8) | 72.54(6.2) | 75.86(6.8) | 80.95(4.6) | 99.55(10.4) | 97.15(9.3) | 95.18(8.2) | 98.24(7.9) | 96.24(6.4) | 95.48(5.3) |
| 659 | Methylprednisolone | 92.58(4.8) | 95.68(10.3) | 97.68(9.5) | 75.89(4.4) | 77.89(6.5) | 79.63(4.8) | 89.65(12.6) | 87.15(9.4) | 85.27(8.5) | 101.54(10.3) | 98.57(7.3) | 95.47(6.4) |
| 660 | Mometasone Furoate | 91.58(3.6) | 92.68(4.8) | 95.86(7.9) | 71.89(6.4) | 75.68(4.6) | 80.59(7.8) | 96.55(14.4) | 94.18(10.3) | 91.48(8.2) | 71.49(6.4) | 73.84(6.3) | 67.19(5.3) |
| 661 | Prednicarbate | 88.32(4.1) | 89.67(4.9) | 93.48(8.4) | 78.59(6.3) | 81.49(6.7) | 83.64(8.8) | 89.75(9.2) | 87.48(8.3) | 86.35(4.8) | 87.51(8.4) | 84.59(7.5) | 83.49(6.5) |
| 662 | Testosterone | 84.96(3.4) | 86.59(7.4) | 89.64(8.7) | 96.48(2.3) | 92.84(7.8) | 90.26(5.8) | 113.5(10.5) | 107.49(8.6) | 105.29(6.3) | 93.54(7.5) | 90.58(6.3) | 88.47(5.2) |
| 663 | Triamcinolone acetonide | 76.52(10.3) | 78.59(8.4) | 82.54(6.3) | 95.24(7.3) | 93.48(4.5) | 90.12(6.6) | 87.25(10.3) | 85.14(7.5) | 83.49(6.5) | 98.14(8.5) | 95.46(7.1) | 93.48(4.2) |
| 664 | Bambuterol | 85.21(2.3) | 83.46(7.2) | 86.49(6.3) | 90.23(3.2) | 94.58(8.2) | 95.86(6.3) | 74.15(8.1) | 76.34(5.4) | 78.36(5.8) | 99.15(12.5) | 96.54(3.5) | 93.78(6.9) |
| 665 | Hydroxymethyl clenbuterol | 93.65(4.3) | 91.58(6.5) | 88.63(7.2) | 86.35(2.5) | 88.94(4.6) | 87.49(5.2) | 99.15(9.4) | 96.54(7.3) | 94.87(6.9) | 96.27(14.6) | 97.68(8.7) | 94.18(6.6) |
| 666 | Clencyclohexerol | 86.59(12.3) | 88.54(6.3) | 90.23(3.6) | 91.46(8.4) | 93.56(1.3) | 92.85(10.5) | 97.45(8.6) | 95.18(5.4) | 92.67(3.7) | 96.58(8.6) | 94.18(6.4) | 91.87(5.3) |
| 667 | Clenhexerol | 68.54(6.5) | 69.35(6.2) | 72.58(8.0) | 64.59(12.6) | 68.49(10.2) | 73.56(5.5) | 102.5(6.6) | 96.47(5.2) | 95.18(3.7) | 74.25(16.4) | 72.18(15.2) | 69.58(10.2) |
| 668 | Clenisopenterol | 81.46(3.4) | 83.96(6.4) | 88.76(7.8) | 83.65(6.5) | 85.96(4.2) | 88.56(12.6) | 107.5(10.4) | 103.69(8.5) | 98.57(6.4) | 82.68(8.4) | 80.69(7.5) | 77.35(6.5) |
| 669 | Clenproperol | 90.56(2.3) | 92.48(8.1) | 93.48(7.5) | 74.23(10.3) | 78.65(8.9) | 81.49(3.6) | 98.25(5.2) | 95.28(6.4) | 97.28(5.5) | 77.49(12.4) | 75.21(9.4) | 73.89(6.4) |
| 670 | Fenoterol | 77.49(3.5) | 79.65(5.4) | 82.65(7.8) | 83.59(4.5) | 88.67(6.9) | 90.36(6.4) | 86.35(7.0) | 83.74(5.3) | 81.87(4.3) | 98.57(8.7) | 96.28(6.4) | 94.28(3.7) |
| 671 | Formoterol | 71.52(5.5) | 75.68(6.9) | 78.96(6.8) | 90.35(6.5) | 92.86(8.4) | 96.35(6.4) | 88.95(8.5) | 85.27(8.0) | 83.19(5.0) | 97.58(10.3) | 95.21(8.7) | 93.16(6.4) |
| 672 | Pirbuterol | 81.56(3.6) | 83.64(7.2) | 86.95(8.2) | 70.31(4.8) | 72.56(6.8) | 75.68(10.3) | 99.65(8.7) | 96.58(6.4) | 95.18(4.2) | 81.98(11.3) | 79.65(9.4) | 77.15(7.3) |
| 673 | Ractopamine | 88.52(4.1) | 89.63(6.8) | 92.54(7.9) | 81.52(3.2) | 83.59(4.9) | 87.52(6.9) | 88.55(9.4) | 87.24(9.4) | 86.18(8.3) | 85.79(9.7) | 83.54(6.4) | 80.96(5.3) |
| 674 | Ritodrine | 102.58(4.9) | 99.65(4.8) | 96.32(1.7) | 82.51(6.1) | 86.32(7.8) | 85.26(9.6) | 82.55(10.4) | 80.26(8.6) | 78.64(6.4) | 94.87(8.4) | 92.54(7.4) | 90.38(6.5) |
| 675 | Salmeterol | 71.49(6.5) | 77.54(3.6) | 76.59(10.6) | 85.26(11.3) | 86.24(8.5) | 89.65(7.8) | 88.55(12.4) | 86.29(9.7) | 84.28(7.3) | 78.21(6.4) | 75.14(5.3) | 73.19(4.3) |
| 676 | Sotalol | 70.23(4.5) | 72.35(6.8) | 73.59(8.2) | 71.92(7.3) | 73.59(8.2) | 79.63(10.3) | 96.25(9.3) | 95.15(8.3) | 93.18(5.2) | 91.62(8.5) | 89.25(7.2) | 87.19(6.4) |
| 677 | Terbutaline | 74.51(2.3) | 75.84(6.3) | 78.65(10.2) | 83.26(10.2) | 85.96(7.8) | 88.52(4.6) | 85.45(9.4) | 83.19(5.7) | 81.47(4.0) | 99.58(9.4) | 97.15(7.3) | 96.18(6.1) |
| 678 | Tulobuterol | 84.12(10.2) | 86.52(4.9) | 88.76(8.2) | 75.28(6.9) | 76.59(10.2) | 79.65(6.3) | 90.25(6.4) | 87.19(5.2) | 88.76(4.6) | 84.96(9.3) | 81.49(8.6) | 78.34(5.0) |
| 679 | Clenpenterol | 114.3(10.2) | 118.65(11.9) | 122.5(8.9) | 85.64(7.3) | 86.95(9.3) | 88.15(9.6) | 113.5(7.3) | 116.27(8.4) | 119.48(6.4) | 86.92(8.6) | 85.19(6.9) | 83.17(5.3) |
| 680 | 4-Acetamidophenol | 71.56(5.2) | 73.98(6.5) | 77.95(8.3) | 91.54(6.2) | 93.58(7.3) | 95.68(6.5) | 88.25(7.6) | 86.29(5.3) | 85.16(4.3) | 78.62(9.3) | 76.29(8.3) | 75.19(5.3) |
| 681 | Chlorpromazine | 92.54(6.8) | 95.78(7.3) | 98.59(6.8) | 73.59(4.2) | 76.98(8.5) | 79.65(11.5) | 106.5(8.3) | 108.96(6.3) | 110.26(6.1) | 88.29(11.4) | 86.29(9.8) | 85.16(6.3) |
| 682 | Clopidol | 82.59(5.6) | 83.49(6.8) | 86.79(9.5) | 90.23(6.5) | 87.46(7.5) | 85.12(6.5) | 98.15(6.9) | 102.58(7.4) | 105.69(8.5) | 97.16(8.4) | 95.18(5.3) | 91.46(4.2) |
| 683 | Dapsone | 81.52(3.6) | 85.62(6.3) | 88.69(9.3) | 70.52(4.2) | 72.59(6.3) | 75.68(8.2) | 88.35(7.3) | 86.59(4.6) | 85.37(3.6) | 86.35(11.5) | 85.34(9.5) | 83.19(7.1) |
| 684 | Carbadox | 92.58(4.8) | 94.16(3.6) | 97.36(5.4) | 90.25(4.4) | 95.68(6.5) | 94.78(5.6) | 85.15(6.6) | 88.96(5.1) | 90.48(7.3) | 91.84(7.9) | 88.17(6.4) | 86.92(5.6) |
| 685 | Haloperidol | 86.52(5.2) | 90.24(6.8) | 91.78(8.4) | 72.58(5.4) | 74.96(10.2) | 76.85(8.2) | 85.15(7.1) | 87.59(8.7) | 88.96(9.1) | 93.48(12.6) | 90.25(9.5) | 88.52(7.3) |
| 686 | Azaperol | 90.89(4.5) | 92.58(7.4) | 93.85(6.7) | 81.58(4.8) | 83.59(4.9) | 87.49(12.6) | 93.65(9.1) | 91.58(7.5) | 89.25(5.5) | 96.48(8.6) | 94.68(6.3) | 92.48(5.2) |
| 687 | Azaperone | 86.52(4.5) | 88.63(7.4) | 90.54(9.8) | 84.52(7.4) | 86.75(9.4) | 90.45(6.8) | 84.55(6.3) | 83.64(5.1) | 81.94(3.6) | 88.59(8.7) | 86.75(9.6) | 84.21(7.2) |
| 688 | Propionylpromazine | 88.54(5.4) | 90.35(6.9) | 91.58(11.2) | 89.63(4.7) | 92.35(6.6) | 94.87(7.8) | 96.85(9.5) | 94.87(8.3) | 91.46(6.3) | 97.62(8.5) | 95.28(7.1) | 93.68(5.1) |
| 689 | Xylazine | 74.25(5.3) | 77.85(6.7) | 79.35(11.2) | 97.25(4.5) | 99.38(5.4) | 101.6(8.4) | 84.15(7.6) | 82.59(4.9) | 78.58(4.1) | 87.54(8.2) | 85.43(6.9) | 82.49(4.1) |
| 690 | Carbamazepine | 88.52(5.4) | 90.23(4.8) | 93.65(6.4) | 77.58(8.2) | 79.65(14.6) | 82.95(7.4) | 95.85(11.5) | 93.48(9.3) | 90.16(7.3) | 92.16(7.9) | 90.13(6.3) | 87.45(4.2) |
| 691 | Diphenhydramine | 91.58(3.1) | 88.54(7.9) | 86.59(8.5) | 72.59(5.4) | 76.93(8.2) | 80.56(9.5) | 93.75(6.4) | 91.46(3.6) | 88.65(2.1) | 87.49(9.6) | 85.12(7.3) | 82.46(6.4) |
| 692 | Imipramine | 86.52(3.5) | 88.65(4.9) | 91.58(7.8) | 81.59(6.3) | 83.54(8.2) | 86.52(9.3) | 92.55(6.0) | 88.65(5.4) | 84.63(3.3) | 93.58(7.4) | 91.46(6.4) | 88.34(5.3) |
| 693 | Sulpiride | 73.58(5.4) | 76.89(7.5) | 79.65(8.8) | 85.69(13.5) | 86.79(5.9) | 89.78(8.6) | 96.35(11.3) | 95.47(9.3) | 91.48(7.2) | 99.45(11.6) | 97.21(8.3) | 92.18(5.2) |
| 694 | Zolpidem | 74.51(2.3) | 75.84(6.3) | 80.59(6.8) | 88.76(4.6) | 92.86(6.5) | 93.58(4.7) | 88.95(8.5) | 85.21(7.2) | 83.49(2.6) | 86.35(9.5) | 85.26(8.5) | 82.16(7.2) |
| 695 | Fluoxetine | 70.25(8.5) | 72.59(10.6) | 77.59(8.9) | 92.58(6.9) | 93.48(8.5) | 95.84(9.5) | 94.85(9.5) | 92.48(8.2) | 85.16(5.5) | 93.48(11.5) | 91.47(8.1) | 87.15(7.1) |
| 696 | Coffeine | 91.25(5.7) | 95.78(7.1) | 103.02(6.2) | 92.56(2.2) | 95.34(7.9) | 100.56(2.6) | 95.85(8.3) | 93.19(6.1) | 90.19(3.1) | 90.16(8.9) | 88.06(8.2) | 85.15(7.1) |
| 697 | Codeine | 89.56(3.2) | 87.68(9.8) | 78.59(8.3) | 95.46(5.3) | 85.26(5.8) | 78.35(6.6) | 97.15(9.2) | 95.84(8.6) | 90.48(5.2) | 93.18(5.5) | 90.16(5.4) | 88.19(3.1) |
| 698 | 1,7-Dimethylxanthine | 90.58(4.7) | 95.71(4.4) | 100.25(3.6) | 98.56(4.2) | 96.34(3.8) | 88.76(7.6) | 107.5(8.2) | 105.96(5.5) | 100.26(5.2) | 96.48(7.5) | 94.28(4.7) | 91.02(5.2) |
| 699 | Chloroprocaine | 56.87(23.9) | 60.48(18.5) | 65.94(8.5) | 95.34(6.7) | 95.34(5.3) | 87.36(8.5) | 92.45(20.6) | 90.15(19.3) | 87.54(12.5) | 88.15(15.5) | 85.26(13.4) | 80.26(15.0) |
| 700 | Cinchocaine | 93.78(3.2) | 87.8(7.2) | 97.89(8.2) | 86.37(6.7) | 90.78(7.6) | 87.26(7.8) | 88.55(9.2) | 89.63(7.5) | 84.17(3.7) | 86.34(8.5) | 84.16(4.3) | 83.49(2.2) |
| 701 | Lidocaine | 98.76(2.6) | 91.58(2.9) | 94.87(7.9) | 95.27(1.6) | 90.78(7.8) | 91.85(6.7) | 113.5(15.9) | 110.35(12.6) | 105.49(10.6) | 106.24(16.4) | 104.78(13.5) | 101.25(9.8) |
| 702 | Procainamide | 91.25(5.7) | 95.78(7.2) | 103.02(6.3) | 88.46(5.3) | 93.26(5.9) | 88.35(6.6) | 103.5(4.9) | 100.26(5.2) | 95.17(3.2) | 98.15(9.7) | 95.24(8.5) | 93.58(7.0) |
| 703 | Procaine | 95.23(4.8) | 88.57(6.8) | 96.75(6.3) | 74.28(6.5) | 81.24(8.6) | 83.47(8.9) | 98.55(9.2) | 95.16(7.3) | 92.87(5.0) | 95.14(9.1) | 93.18(7.1) | 90.62(4.6) |
| 704 | Tetracaine | 90.25(6.3) | 88.75(8.3) | 92.87(6.0) | 75.94(1.4) | 78.47(3.9) | 82.56(8.4) | 99.45(8.6) | 97.15(7.2) | 94.18(6.5) | 93.48(6.5) | 95.68(8.2) | 90.13(6.2) |
| 705 | Brompheniramine | 90.78(4.7) | 95.67(3.9) | 99.63(8.4) | 78.65(6.8) | 80.35(7.2) | 87.12(8.8) | 98.15(8.1) | 95.12(5.5) | 93.08(2.6) | 95.48(7.2) | 93.15(5.5) | 91.02(2.5) |
| 706 | Cetirizine | 94.56(4.6) | 90.65(5.8) | 89.36(7.8) | 88.36(8.4) | 87.21(7.6) | 84.35(7.4) | 88.75(7.1) | 85.46(6.2) | 81.29(4.2) | 89.57(10.3) | 87.41(9.3) | 84.71(6.4) |
| 707 | Chlorpheniramine | 88.25(7.3) | 94.37(8.2) | 103.02(6.3) | 79.34(8.1) | 83.49(7.3) | 90.24(7.6) | 87.45(6.5) | 85.21(4.1) | 83.29(2.1) | 88.57(9.3) | 85.14(8.6) | 83.16(8.1) |
| 708 | Fluphenazine | 81.75(4.5) | 85.49(6.8) | 91.02(2.5) | 74.52(10.3) | 79.64(3.9) | 85.67(8.7) | 96.05(8.5) | 94.10(7.1) | 90.52(5.0) | 95.18(10.3) | 92.14(7.5) | 89.27(5.0) |
| 709 | Hydroxyzine | 77.68(2.4) | 80.52(5.3) | 84.73(7.1) | 81.38(4.9) | 86.35(8.7) | 90.54(5.7) | 90.25(9.0) | 92.87(7.1) | 85.21(6.4) | 92.58(8.5) | 90.87(6.3) | 87.54(5.0) |
| 710 | Promethazine | 95.68(4.6) | 90.21(4.2) | 89.47(6.5) | 80.23(7.6) | 79.87(7.0) | 85.63(10.2) | 87.45(7.0) | 89.75(8.7) | 84.19(3.7) | 88.95(8.5) | 85.16(7.5) | 81.95(5.1) |
| 711 | Terfenadine | 94.28(7.1) | 97.21(5.6) | 101.22(3.6) | 85.46(5.4) | 89.54(10.4) | 87.59(7.2) | 94.25(8.6) | 90.62(7.6) | 85.27(6.3) | 92.84(6.3) | 89.54(5.2) | 85.97(6.0) |
| 712 | Bifonazole | 76.38(8.7) | 81.36(8.1) | 88.36(10.6) | 102.36(3.4) | 110.89(6.9) | 98.34(12.4) | 95.85(8.7) | 93.15(6.1) | 88.36(5.1) | 92.15(8.5) | 88.25(6.4) | 85.74(5.0) |
| 713 | Econazole | 78.64(8.2) | 95.31(7.3) | 108.40(8.2) | 93.15(4.8) | 103.87(6.6) | 87.34(3.8) | 82.55(9.0) | 80.52(7.3) | 78.52(2.7) | 79.85(4.6) | 77.25(5.9) | 75.32(3.1) |
| 714 | Griseofulvin | 93.65(4.3) | 88.63(6.4) | 80.32(8.7) | 92.13(2.2) | 94.58(4.0) | 101.54(5.3) | 89.65(5.5) | 88.63(2.8) | 85.15(4.6) | 88.25(6.8) | 85.49(4.2) | 83.03(2.9) |
| 715 | Ketoconazole | 87.52(3.8) | 90.23(6.5) | 84.36(5.7) | 89.63(4.9) | 91.23(6.4) | 85.86(7.2) | 88.45(9.2) | 85.16(6.6) | 82.19(2.5) | 89.63(10.3) | 85.29(9.5) | 86.95(6.2) |
| 716 | Naftifine | 83.25(6.2) | 85.87(4.6) | 89.76(9.6) | 75.62(6.0) | 73.26(6.0) | 83.56(9.6) | 87.55(9.5) | 85.87(7.2) | 83.06(5.4) | 91.06(10.5) | 89.75(8.5) | 86.19(5.5) |
| 717 | Flunixin | 115.89(6.8) | 105.96(10.2) | 93.02(6.4) | 110.86(14.4) | 106.80(7.5) | 94.36(8.9) | 95.85(8.5) | 93.15(5.5) | 91.04(3.3) | 93.68(9.9) | 90.21(7.5) | 88.05(6.4) |
| 718 | Ketotifen | 85.23(3.1) | 84.2(7.3) | 75.96(10.2) | N.D | 73.26(6.8) | 81.56(6.2) | 98.55(9.6) | 95.02(7.1) | 93.18(5.2) | 95.84(8.6) | 92.15(6.2) | 90.63(5.1) |
| 719 | Lornoxicam | 93.65(4.5) | 90.56(4.6) | 87.26(3.5) | 102.50(4.8) | 110.35(6.9) | 95.80(5.0) | 92.85(8.2) | 90.56(4.6) | 87.26(4.0) | 89.57(9.0) | 87.52(7.6) | 85.24(7.0) |
| 720 | Melitracen | 76.59(14.8) | 79.36(6.4) | 82.54(7.2) | 94.23(7.2) | 92.36(6.5) | 97.80(4.2) | 95.15(9.9) | 92.15(7.5) | 89.14(5.2) | 94.23(7.2) | 92.36(6.5) | 87.14(4.2) |
| 721 | Oxaprozin | 94.89(3.9) | 96.85(7.2) | 103.84(8.8) | 74.56(5.5) | 72.89(6.9) | 80.56(7.9) | 92.25(12.8) | 89.67(9.2) | 87.59(7.3) | 89.63(11.2) | 87.12(9.4) | 85.25(7.9) |
| 722 | Antipyrine | 94.36(1.6) | 92.8(6.1) | 100.2(4.1) | 109.5(3.8) | 101.36(6.1) | 95.36(5.1) | 99.85(9.3) | 97.15(8.5) | 95.88(5.2) | 96.25(7.3) | 92.87(5.4) | 89.25(3.2) |
| 723 | Sulfaguanidine | 86.58(3.4) | 90.25(7.7) | 93.25(4.2) | 79.45(2.2) | 83.96(4.5) | 86.24(6.6) | 97.45(6.4) | 94.31(5.1) | 91.64(4.3) | 96.87(5.5) | 93.45(4.1) | 91.28(3.1) |
| 724 | Difloxacin hydrochloride | 71.52(5.5) | 75.52(7.3) | 81.45(6.4) | 60.49(5.6) | 70.84(3.1) | 72.68(6.7) | 89.65(7.3) | 84.57(6.2) | 82.49(4.3) | 87.26(5.5) | 89.45(6.4) | 91.25(3.5) |
| 725 | Fleroxacin | 81.49(3.5) | 83.96(7.5) | 88.76(5.6) | 92.45(4.4) | 95.76(6.4) | 88.76(6.3) | 87.55(4.7) | 85.23(3.7) | 83.57(5.4) | 99.68(8.8) | 94.58(6.3) | 95.63(4.3) |
| 726 | Enoxacin | 74.58(3.2) | 78.64(8.2) | 77.69(4.3) | 90.85(6.1) | 92.76(10.2) | 94.81(7.9) | 84.55(8.2) | 81.25(6.3) | 79.65(4.3) | 105.26(9.3) | 101.23(7.5) | 97.26(5.3) |
| 727 | Sarafloxacin hydrochloride | 81.69(3.1) | 85.74(6.5) | 90.85(6.7) | 80.96(4.8) | 83.25(5.6) | 85.71(9.4) | 93.65(6.3) | 91.28(7.5) | 88.54(4.2) | 88.54(6.3) | 87.59(5.3) | 86.31(2.7) |
| 728 | Nadifloxacin | 72.18(6.6) | 73.48(5.4) | 77.49(4.8) | N.D | 71.48(6.6) | 77.49(6.9) | 95.85(8.3) | 93.46(6.2) | 90.18(2.5) | 96.54(8.1) | 92.48(5.4) | 93.76(6.4) |
| 729 | Norfloxacin | 103.54(3.6) | 98.74(6.1) | 95.18(4.2) | 85.21(4.1) | 88.27(5.5) | 89.64(6.8) | 103.5(6.1) | 100.25(4.3) | 98.45(3.2) | 98.65(6.3) | 96.45(5.0) | 93.48(3.1) |
| 730 | Gemifloxacin mesylate | 90.54(6.2) | 93.75(7.5) | 91.45(2.6) | 77.54(2.8) | 80.28(4.6) | 85.47(6.3) | 76.55(8.5) | 74.65(7.2) | 72.16(4.2) | 90.48(6.3) | 88.26(5.1) | 86.54(3.2) |
| 731 | Glipizide | 85.14(6.2) | 86.54(7.5) | 91.45(2.6) | 81.75(5.6) | 85.79(4.6) | 87.94(6.3) | 93.45(8.1) | 90.15(6.6) | 88.32(3.2) | 88.95(8.7) | 85.79(4.6) | 83.46(3.8) |
| 732 | Repaglinide | 80.95(4.1) | 83.45(8.5) | 86.54(6.9) | 91.48(6.4) | 92.85(3.1) | 96.48(6.4) | 116.5(10.4) | 115.48(9.2) | 110.25(5.2) | 99.64(7.3) | 97.41(5.2) | 95.18(3.3) |
| 733 | Hygromycin B | 79.65(6.2) | 83.49(6.9) | 85.41(6.3) | N.D | 105.24(6.4) | 108.64(8.1) | 94.25(7.5) | 91.85(6.5) | 88.46(3.2) | 83.26(4.9) | 82.19(5.5) | 79.16(2.6) |
| 734 | Sulfisoxazole | 76.48(3.2) | 78.94(5.5) | 81.49(8.1) | 92.84(3.6) | 90.18(6.5) | 84.51(6.5) | 96.55(8.2) | 93.46(6.2) | 90.85(4.1) | 90.12(6.5) | 88.75(5.3) | 86.59(3.5) |
| 735 | Amikacin | 90.18(3.2) | 93.18(7.1) | 89.64(2.2) | 94.18(6.2) | 90.18(3.8) | 92.31(6.8) | 94.75(5.6) | 91.20(4.8) | 89.64(4.9) | 85.22(6.3) | 86.95(5.3) | 81.49(3.5) |
| 736 | Tolbutamide | 95.14(3.1) | 96.15(5.4) | 98.16(3.6) | 66.18(2.4) | 70.15(6.2) | 72.19(7.3) | 98.55(4.3) | 95.62(3.5) | 93.85(2.1) | 88.65(9.5) | 86.32(7.5) | 83.45(5.3) |
| 737 | Gliclazide | 88.47(2.8) | 90.18(3.6) | 86.15(7.9) | 90.25(3.8) | 92.54(6.1) | 95.36(4.9) | 87.65(4.9) | 85.32(4.2) | 82.97(3.0) | 96.24(5.3) | 94.58(3.6) | 90.63(2.7) |
| 738 | Gliquidone | 77.18(4.6) | 82.17(6.5) | 85.49(6.9) | 90.48(2.2) | 93.48(4.8) | 94.58(8.1) | 84.55(9.4) | 82.16(4.8) | 80.23(3.5) | 73.54(10.3) | 76.49(8.5) | 78.42(5.3) |
| 739 | Glyburide | 89.58(6.1) | 92.18(6.1) | 87.18(6.9) | 91.47(2.1) | 92.54(4.6) | 95.18(8.5) | 93.45(8.5) | 92.18(7.2) | 87.18(5.5) | 83.54(6.0) | 86.34(4.2) | 88.54(3.1) |
| 740 | 1-Aminohydantoin hydrochloride | 71.04(2.9) | 76.18(3.9) | 80.18(8.5) | 66.18(2.1) | 70.18(5.4) | 75.19(3.6) | N.D | N.D | 70.15(8.7) | N.D | 115.26(8.5) | 117.48(5.5) |
| 741 | Pioglitazone hydrochloride | 84.17(2.6) | 88.17(6.8) | 89.32(4.4) | 70.98(5.4) | 75.18(2.5) | 79.64(3.5) | 87.55(5.2) | 85.62(4.7) | 82.44(3.2) | 84.96(5.7) | 80.59(4.1) | 78.20(3.2) |
| 742 | Metformin hydrochloride | 89.54(4.8) | 92.54(5.7) | 93.67(4.6) | 81.95(7.2) | 84.59(6.4) | 88.76(7.3) | 94.55(8.8) | 90.25(6.1) | 87.15(2.7) | 74.25(10.3) | 77.58(9.2) | 80.12(6.3) |
| 743 | Glimepiride | 100.28(5.8) | 106.28(6.7) | 109.48(6.4) | 93.48(5.8) | 90.28(6.9) | 88.17(3.9) | 100.5(8.1) | 95.48(6.3) | 91.47(3.3) | 80.23(11.5) | 78.25(6.3) | 75.21(5.7) |
| 744 | Sulfamonomethoxine | 89.58(6.4) | 92.64(5.4) | 93.64(6.9) | 91.68(6.4) | 93.68(7.8) | 95.18(6.3) | 105.5(7.3) | 95.62(5.5) | 93.48(4.1) | 105.88(9.6) | 101.55(5.6) | 98.25(4.0) |
| 745 | Sulfacetamide | 87.48(6.9) | 90.68(2.4) | 93.64(7.8) | N.D | N.D | N.D | 98.65(8.3) | 95.24(6.3) | 93.47(4.2) | 98.67(8.5) | 95.48(7.3) | 92.88(6.1) |
| 746 | Sulfachlorpyridazine | 78.68(3.2) | 81.49(7.3) | 86.65(6.9) | 74.18(3.5) | 76.58(4.9) | 82.36(4.5) | 88.55(7.5) | 85.32(6.5) | 82.75(5.4) | 97.84(7.3) | 96.83(6.5) | 94.12(5.0) |
| 747 | Sulfamoxole | 86.12(8.3) | 88.46(3.7) | 92.74(6.7) | 84.58(6.1) | 88.67(3.9) | 90.25(6.9) | 90.55(5.3) | 87.59(3.5) | 84.16(2.2) | 92.84(9.6) | 90.55(8.3) | 87.23(4.6) |
| 748 | Sulfanitran | 76.58(6.4) | 78.96(9.6) | 82.14(6.4) | 80.95(2.8) | 83.48(4.4) | 86.49(9.5) | 96.35(7.5) | 94.16(5.4) | 92.48(4.3) | 88.45(7.2) | 85.32(5.7) | 83.59(4.2) |
| 749 | Sulfameter | 76.58(6.8) | 78.96(4.6) | 81.39(7.5) | 89.64(6.4) | 90.28(3.9) | 92.48(7.5) | 85.65(8.2) | 83.49(6.2) | 81.46(5.2) | 90.25(8.2) | 88.25(6.3) | 85.29(4.2) |
| 750 | Sulfamethoxazole | 90.48(3.2) | 85.49(6.4) | 82.18(9.4) | 81.74(3.6) | 85.49(6.2) | 88.49(7.5) | 90.25(10.3) | 88.44(8.3) | 85.29(7.2) | 95.24(8.3) | 93.48(6.3) | 90.22(4.1) |
| 751 | Buformin hydrochloride | 85.49(5.6) | 88.17(7.2) | 90.58(3.6) | 89.25(6.8) | 92.85(4.5) | 95.84(6.6) | 91.55(7.3) | 88.17(6.0) | 85.14(4.8) | 82.54(7.5) | 85.29(6.3) | 84.19(4.2) |
| 752 | Phenformin hydrochloride | 73.48(3.9) | 76.58(5.2) | 79.28(6.4) | 70.18(6.6) | 73.48(4.6) | 77.49(9.5) | 73.45(8.7) | 70.59(5.9) | 68.49(4.1) | 94.85(9.3) | 90.42(7.5) | 86.54(6.3) |
| 753 | Glibornuride | 75.20(6.9) | 78.49(6.8) | 81.49(9.2) | 84.15(6.3) | 88.54(3.6) | 90.28(6.3) | 78.45(11.3) | 76.23(9.2) | 73.45(8.2) | 89.77(7.5) | 86.23(6.5) | 85.98(4.1) |
| 754 | Ciprofloxacin | 89.54(5.5) | 86.18(4.6) | 72.58(4.8) | 85.49(3.6) | 88.74(3.2) | 90.58(5.5) | 95.85(8.5) | 92.46(6.2) | 88.55(4.2) | 82.49(2.4) | 80.30(2.0) | 77.15(1.2) |
| 755 | Kanamycin sulfate | N.D | N.D | N.D | N.D | N.D | N.D | N.D | N.D | 72.58(4.2) | N.D | N.D | 70.25(5.2) |
| 756 | Tobramycin | N.D | N.D | N.D | N.D | N.D | N.D | 95.85(8.2) | 93.12(6.0) | 88.16(3.5) | 85.49(6.0) | 82.16(4.5) | 78.26(3.5) |

**Table S5.** The linear ranges, correlation coefficients (r^2^) and matrix effects for the 756 database compounds in the four representative matrices.

| **No.** | **Compound name** | **Tilapia** | | | **Grouper** | | | **Oyster** | | | **Scallop** | | |
| --- | --- | --- | --- | --- | --- | --- | --- | --- | --- | --- | --- | --- | --- |
|  |  | **Linear range  (ng/mL)** | **r^2^** | **Matrix effect, %** | **Linear range  (ng/mL)** | **r^2^** | **Matrix effect, %** | **Linear range  (ng/mL)** | **r^2^** | **Matrix effect, %** | **Linear range  (ng/mL)** | **r^2^** | **Matrix effect, %** |
| 1 | Allidochlor | 10-250 | 0.9957 | -8.89% | 10-250 | 0.9982 | 15.60% | 10-250 | 0.9980 | -10.93% | 10-250 | 0.9926 | -13.74% |
| 2 | Dichlormid | 10-250 | 0.9969 | -25.96% | 10-250 | 0.9918 | -26.80% | 10-250 | 0.9967 | 4.23% | 10-250 | 0.9982 | 1.24% |
| 3 | Etridiazole | 10-250 | 0.9945 | 5.74% | 10-250 | 0.9989 | 16.28% | 10-250 | 0.9946 | -8.33% | 10-250 | 0.9928 | 2.38% |
| 4 | Chlormephos | 10-250 | 0.9978 | 27.11% | 10-250 | 0.9999 | 5.03% | 10-250 | 0.9942 | -6.86% | 10-250 | 0.9987 | 8.32% |
| 5 | Propham | N.D | N.D | N.D | N.D | N.D | N.D | 10-250 | 0.9978 | -9.90% | 10-250 | 0.9978 | -5.16% |
| 6 | Cycloate | 10-250 | 0.9945 | -7.07% | 10-250 | 0.9989 | 4.65% | 10-250 | 0.9998 | -8.69% | 10-250 | 0.9999 | -1.38% |
| 7 | Diphenylamine | 10-250 | 0.9920 | -5.84% | 10-250 | 0.9970 | 5.57% | 10-250 | 0.9986 | 6.75% | 10-250 | 0.9972 | -7.47% |
| 8 | Chlordimeform | 10-250 | 0.9995 | 16.05% | 10-250 | 0.9956 | -13.89% | 10-250 | 0.9986 | -4.06% | 10-250 | 0.9955 | 12.90% |
| 9 | Ethalfluralin | N.D | N.D | N.D | N.D | N.D | N.D | 20-250 | 0.9906 | -8.11% | 50-250 | 1.0000 | 31.75% |
| 10 | Phorate | 10-250 | 0.9988 | -6.69% | N.D | N.D | N.D | 10-250 | 0.9921 | -11.58% | 10-250 | 0.9944 | -6.02% |
| 11 | Thiometon | 10-250 | 0.9945 | 7.44% | 10-250 | 0.9989 | 2.66% | 10-250 | 0.9998 | 27.89% | 10-250 | 0.9999 | -10.40% |
| 12 | Quintozene | 10-250 | 0.9950 | -15.11% | 10-250 | 0.9927 | 12.42% | 10-250 | 0.9979 | -11.00% | 10-250 | 0.9977 | 19.20% |
| 13 | Atrazine-desethyl | 10-250 | 0.9958 | -25.67% | 10-250 | 0.9946 | -26.85% | 10-250 | 0.9947 | -3.98% | 10-250 | 0.9980 | -5.70% |
| 14 | Clomazone | 10-250 | 0.9950 | -7.23% | 10-250 | 0.9919 | -14.65% | 10-250 | 0.9981 | -5.22% | 10-250 | 0.9930 | 15.87% |
| 15 | Diazinon | 10-250 | 0.9988 | 20.82% | 10-250 | 0.9951 | -18.06% | 10-250 | 0.9924 | -8.57% | 10-250 | 0.9995 | -6.40% |
| 16 | Fonofos | 10-250 | 0.9934 | -3.72% | N.D | N.D | N.D | 20-250 | 0.9976 | 4.99% | 20-250 | 0.9999 | -2.03% |
| 17 | Etrimfos | 10-250 | 0.9914 | -19.09% | 10-250 | 0.9962 | -14.40% | 10-250 | 0.9989 | 2.49% | 10-250 | 1.0000 | 4.09% |
| 18 | Simazine | 10-250 | 0.9965 | -12.13% | 10-250 | 0.9956 | -9.14% | 10-250 | 0.9954 | 3.30% | 10-250 | 0.9999 | 0.92% |
| 19 | Propetamphos | 10-250 | 0.9998 | -15.93% | 10-250 | 0.9968 | -5.57% | 10-250 | 0.9974 | -10.02% | 10-250 | 0.9997 | 15.81% |
| 20 | Secbumeton | 10-250 | 0.9917 | -19.77% | 10-250 | 0.9973 | -9.50% | 10-250 | 0.9985 | -0.04% | 10-250 | 0.9954 | 29.50% |
| 21 | Dichlofenthion | 10-250 | 0.9963 | 0.75% | 10-250 | 0.9944 | 6.48% | 10-250 | 0.9976 | -4.04% | 10-250 | 0.9945 | -0.05% |
| 22 | Propyzamide | 10-250 | 0.9991 | 12.64% | 10-250 | 0.9904 | 2.55% | 10-250 | 1.0000 | -11.06% | 10-250 | 0.9918 | 6.99% |
| 23 | Mexacarbate | 10-250 | 0.9603 | 9.46% | 10-250 | 0.9912 | 10.15% | 10-250 | 0.9992 | 4.95% | 10-250 | 0.9965 | 7.74% |
| 24 | Aldrin | N.D | N.D | N.D | N.D | N.D | N.D | 10-250 | 0.9945 | -11.17% | 20-250 | 0.9971 | -16.10% |
| 25 | Dinitramine | 10-250 | 0.9917 | -1.18% | 10-250 | 0.9902 | -1.36% | 10-250 | 0.9958 | -1.40% | 10-250 | 0.9961 | 2.23% |
| 26 | Fenchlorphos | 10-250 | 0.9967 | -5.22% | 10-250 | 0.9981 | 7.78% | 10-250 | 0.9912 | 4.07% | 50-250 | 0.9995 | -33.14% |
| 27 | Prometryn | 10-250 | 0.9914 | -0.80% | 10-250 | 0.9924 | -12.10% | 10-250 | 0.9981 | -6.60% | 10-250 | 0.9999 | 5.06% |
| 28 | Cyprazine | 10-250 | 0.9940 | -19.89% | 10-250 | 0.9992 | -6.08% | 10-250 | 0.9989 | 14.16% | 10-250 | 1.0000 | 0.61% |
| 29 | Vinclozolin | 10-250 | 0.9907 | -4.28% | 10-250 | 0.9998 | 4.77% | 10-250 | 0.9909 | 2.12% | 10-250 | 0.9962 | -2.08% |
| 30 | β-HCH | N.D | N.D | N.D | N.D | N.D | N.D | 10-150 | 0.9939 | -1.80% | 10-150 | 0.9988 | 1.86% |
| 31 | Metalaxyl | 10-250 | 0.9934 | 2.60% | 10-250 | 0.9922 | 2.25% | 10-250 | 0.9986 | -4.87% | 10-250 | 0.9930 | -11.36% |
| 32 | Chlorpyrifos | 10-250 | 0.9810 | -6.95% | 10-250 | 0.9998 | -16.26% | 10-250 | 0.9986 | 2.28% | 10-250 | 0.9950 | 5.71% |
| 33 | Parathion-methyl | 10-250 | 0.9999 | 5.15% | 10-250 | 0.9915 | 4.24% | 10-250 | 0.9972 | 4.41% | 10-250 | 0.9967 | -4.91% |
| 34 | Anthraquinone | 10-250 | 0.9909 | -1.98% | 10-250 | 0.9967 | -5.39% | 10-250 | 0.9929 | -4.25% | 10-250 | 0.9941 | 0.20% |
| 35 | δ-HCH | 10-250 | 0.9906 | 4.97% | N.D | N.D | N.D | 10-250 | 0.9942 | -7.66% | 10-250 | 0.9972 | 17.64% |
| 36 | Fenthion | 10-250 | 0.9998 | -8.01% | 10-250 | 0.9931 | -14.71% | 10-250 | 0.9995 | -9.93% | 10-250 | 0.9999 | 13.93% |
| 37 | Malathion | 10-250 | 0.9976 | -15.94% | 10-250 | 0.9935 | -20.14% | 10-250 | 0.9953 | -8.86% | 10-250 | 0.9999 | -6.35% |
| 38 | Fenitrothion | 10-250 | 0.9905 | -7.26% | 10-250 | 0.9972 | -6.29% | 10-250 | 0.9999 | 3.26% | 10-250 | 0.9919 | 7.07% |
| 39 | Paraoxon-ethyl | 10-250 | 0.9935 | -14.89% | 10-250 | 0.9987 | -32.74% | 10-250 | 0.9962 | -13.45% | 10-250 | 0.9961 | -18.76% |
| 40 | Triadimefon | 10-250 | 0.9950 | 11.14% | 10-250 | 0.9927 | 47.19% | 10-250 | 0.9960 | 24.70% | 10-250 | 0.9966 | 4.09% |
| 41 | Parathion | 10-250 | 0.9990 | -2.72% | 10-250 | 0.9975 | -12.22% | 10-250 | 0.9994 | -6.17% | 10-250 | 0.9999 | 2.04% |
| 42 | Pendimethalin | N.D | N.D | N.D | 10-250 | 0.9974 | -20.36% | 10-250 | 0.9927 | 9.48% | 10-250 | 0.9955 | 12.69% |
| 43 | Linuron | 10-250 | 0.9929 | -21.03% | 10-250 | 0.9930 | -31.81% | 10-250 | 0.9973 | -12.56% | 10-250 | 0.9999 | -17.03% |
| 44 | Chlorbenside | N.D | N.D | N.D | N.D | N.D | N.D | 20-250 | 0.9944 | -11.85% | 20-250 | 0.9990 | 14.77% |
| 45 | Bromophos-ethyl | N.D | N.D | N.D | N.D | N.D | N.D | 20-250 | 0.9982 | 3.13% | 10-250 | 0.9993 | -4.52% |
| 46 | Quinalphos | 10-250 | 1.0000 | -1.63% | 10-250 | 0.9925 | -1.74% | 10-250 | 0.9950 | 5.03% | 10-250 | 0.9998 | -4.52% |
| 47 | trans-Chlordane (γ) | 10-250 | 0.9999 | -5.54% | 10-250 | 0.9964 | -9.10% | 10-250 | 0.9936 | 2.40% | 10-250 | 0.9988 | 6.93% |
| 48 | Phenthoate | N.D | N.D | N.D | N.D | N.D | N.D | 10-250 | 0.9991 | 0.36% | 10-250 | 0.9990 | 3.81% |
| 49 | Metazachlor | 10-250 | 0.9968 | -12.21% | 10-250 | 0.9966 | -22.74% | 10-250 | 0.9935 | 15.88% | 10-250 | 0.9988 | -4.62% |
| 50 | Fenothiocarb | 10-250 | 0.9990 | -22.63% | 10-250 | 0.9995 | -9.31% | 10-250 | 0.9943 | 15.35% | 10-250 | 0.9989 | -13.81% |
| 51 | Prothiophos | 10-250 | 0.9927 | -20.98% | 10-250 | 0.9946 | -9.69% | 10-250 | 0.9990 | -15.49% | 10-250 | 0.9987 | 12.72% |
| 52 | Chlorflurenol-methyl | N.D | N.D | N.D | N.D | N.D | N.D | 20-250 | 0.9993 | -22.91% | 20-250 | 0.9983 | 24.00% |
| 53 | Dieldrin | 10-250 | 0.9972 | -14.47% | 10-250 | 0.9923 | -12.89% | 10-250 | 0.9983 | 20.29% | 10-250 | 0.9980 | 3.89% |
| 54 | Procymidone | 10-250 | 0.9923 | -5.41% | 10-250 | 0.9919 | -14.57% | 10-250 | 0.9973 | 1.24% | 10-250 | 0.9985 | -3.21% |
| 55 | Methidathion | N.D | N.D | N.D | N.D | N.D | N.D | 10-250 | 0.9960 | -21.98% | 10-250 | 0.9934 | -21.57% |
| 56 | Cyanazine | 10-250 | 0.9954 | -9.32% | 10-250 | 0.9974 | -10.95% | 10-250 | 0.9981 | 19.23% | 10-250 | 0.9968 | 4.80% |
| 57 | Napropamide | 10-250 | 0.9946 | -7.49% | 10-250 | 0.9980 | -2.56% | 10-250 | 0.9961 | -18.69% | 10-250 | 0.9983 | 4.87% |
| 58 | Oxadiazon | N.D | N.D | N.D | N.D | N.D | N.D | 10-250 | 0.9944 | -5.73% | 10-250 | 1.0000 | 2.74% |
| 59 | Fenamiphos | 10-250 | 0.9959 | -19.10% | 10-250 | 0.9929 | -24.94% | 10-250 | 0.9997 | 17.21% | 10-250 | 0.9996 | 11.31% |
| 60 | Tetrasul | 10-250 | 0.9815 | -19.74% | 10-250 | 0.9957 | -7.47% | 10-250 | 0.9961 | -21.68% | 10-250 | 0.9992 | -10.48% |
| 61 | Aramite | 10-250 | 0.9928 | -0.98% | 10-250 | 0.9989 | 11.12% | 10-250 | 0.9984 | -3.34% | 10-250 | 0.9964 | 4.27% |
| 62 | Bupirimate | 10-250 | 0.9889 | 21.36% | 10-250 | 0.9936 | 30.67% | 10-250 | 0.9908 | -0.56% | 10-250 | 0.9930 | 11.69% |
| 63 | Carboxin | 10-250 | 0.9963 | 6.16% | 10-250 | 0.9985 | 3.97% | 10-250 | 0.9989 | -11.24% | 10-250 | 0.9950 | -0.48% |
| 64 | Flutolanil | 10-250 | 0.9974 | -5.51% | 10-250 | 0.9942 | -1.37% | 10-150 | 0.9934 | 32.14% | 10-250 | 0.9971 | 31.29% |
| 65 | 4,4'-DDD | 10-250 | 0.9947 | -2.71% | 10-250 | 0.9993 | -3.98% | 10-250 | 0.9968 | 6.88% | 10-250 | 0.9958 | 8.49% |
| 66 | Ethion | 10-250 | 0.9998 | -12.05% | 10-250 | 0.9995 | -13.11% | 10-250 | 0.9929 | -16.98% | 10-250 | 0.9987 | 1.69% |
| 67 | Sulprofos | 10-250 | 0.9987 | -26.30% | 10-250 | 0.9902 | -20.58% | 10-250 | 0.9998 | -21.80% | 10-250 | 0.9979 | -3.40% |
| 68 | Etaconazole | 10-250 | 0.9964 | 7.38% | 10-250 | 0.9981 | -2.53% | 10-250 | 0.9974 | -28.25% | 10-250 | 0.9982 | -8.34% |
| 69 | Myclobutanil | 10-250 | 0.9991 | -36.70% | 10-250 | 0.9962 | -19.83% | 10-250 | 0.9945 | 10.47% | 10-250 | 0.9969 | 9.62% |
| 70 | Diclofop-methyl | 10-250 | 0.9991 | 7.97% | N.D | N.D | N.D | 10-250 | 0.9954 | -4.49% | 10-250 | 0.9992 | -0.51% |
| 71 | Propiconazol | 10-250 | 0.9964 | 4.74% | 10-250 | 0.9986 | 10.97% | 10-250 | 0.9921 | -9.89% | 10-250 | 0.9986 | 4.24% |
| 72 | Fensulfothion | 10-250 | 0.9948 | -14.19% | 10-250 | 0.9931 | -11.46% | 10-250 | 0.9974 | -15.44% | 10-250 | 0.9956 | -22.54% |
| 73 | Bifenthrin | 10-250 | 0.9893 | 1.33% | 10-250 | 0.9983 | 15.09% | 10-250 | 0.9961 | -12.39% | 10-250 | 0.9959 | -16.22% |
| 74 | Mirex | 10-250 | 0.9774 | -3.45% | 10-250 | 0.9966 | 5.54% | 10-250 | 0.9951 | 1.07% | 10-250 | 0.9911 | 8.74% |
| 75 | Benodanil | 10-250 | 0.9973 | -19.77% | 10-250 | 0.9914 | -12.03% | 10-250 | 0.9976 | -1.65% | 10-250 | 0.9992 | -4.99% |
| 76 | Nuarimol | 10-250 | 0.9995 | -26.93% | 10-250 | 0.9973 | -9.60% | 10-250 | 0.9969 | 1.03% | 10-250 | 0.9948 | 1.55% |
| 77 | 4,4'-Methoxychlor | 10-250 | 0.9919 | -17.00% | 10-250 | 0.9945 | -6.45% | 10-250 | 0.9932 | -12.08% | 10-250 | 0.9998 | 11.64% |
| 78 | Oxadixyl | 10-250 | 0.9906 | -7.95% | 10-250 | 0.9986 | 10.68% | 10-250 | 0.9926 | -9.51% | 10-250 | 0.9959 | -12.69% |
| 79 | Tetramethrin | 10-250 | 0.9911 | -23.19% | 10-250 | 0.9809 | -20.15% | 10-250 | 0.9976 | -11.19% | 10-250 | 0.9913 | 7.07% |
| 80 | Tebuconazol | 10-250 | 0.9935 | -26.16% | 10-250 | 0.9934 | -33.46% | 10-250 | 0.9948 | 9.28% | 10-250 | 0.9943 | -17.22% |
| 81 | Norflurazon | 10-250 | 0.9991 | -4.96% | 10-250 | 0.9961 | -3.58% | 10-250 | 0.9992 | -13.81% | 10-250 | 0.9990 | -6.73% |
| 82 | Pyridaphenthion | 10-250 | 0.9963 | -31.94% | 10-250 | 0.9918 | -3.80% | 10-250 | 0.9953 | 12.48% | 10-250 | 0.9936 | 7.96% |
| 83 | Phosmet | 10-250 | 0.9925 | -5.24% | 10-250 | 0.9975 | -8.08% | 10-250 | 0.9973 | -4.99% | 10-250 | 0.9988 | -2.26% |
| 84 | Tetradifon | 10-250 | 0.9963 | -12.85% | 10-250 | 0.9913 | -15.68% | 10-250 | 0.9923 | -11.79% | 10-250 | 0.9979 | -19.31% |
| 85 | Oxycarboxin | 10-250 | 0.9930 | -7.99% | 10-250 | 0.9966 | -14.52% | 10-250 | 0.9934 | 17.24% | 10-250 | 0.9999 | 11.43% |
| 86 | cis-Permethrin | N.D | N.D | N.D | N.D | N.D | N.D | 20-250 | 0.9975 | -20.77% | 20-250 | 0.9974 | 3.43% |
| 87 | trans-Permethrin | N.D | N.D | N.D | N.D | N.D | N.D | 10-250 | 0.9907 | 12.59% | 10-250 | 0.9938 | 5.88% |
| 88 | Pyrazophos | 10-250 | 0.9985 | -10.16% | 10-250 | 0.9964 | -4.43% | 10-250 | 0.9986 | -11.88% | 10-250 | 0.9947 | 18.09% |
| 89 | Cypermethrin | 10-250 | 0.9936 | -22.85% | 10-250 | 0.9927 | -16.70% | 10-250 | 0.9979 | 1.03% | 10-150 | 0.9978 | 44.84% |
| 90 | Fenvalerate | 10-250 | 0.9909 | -9.31% | 10-250 | 0.9956 | -15.56% | 10-250 | 0.9974 | 9.10% | 10-250 | 0.9939 | -12.00% |
| 91 | Deltamethrin | 10-250 | 0.9965 | -24.09% | 10-250 | 0.9920 | -15.95% | 10-250 | 0.9981 | 0.21% | 10-250 | 0.9998 | -1.68% |
| 92 | EPTC | 10-250 | 0.9959 | -14.92% | 10-250 | 0.9972 | -13.47% | 10-250 | 0.9945 | -10.63% | 10-250 | 0.9988 | -17.33% |
| 93 | Butylate | 10-250 | 0.9942 | -24.82% | 10-250 | 0.9957 | -28.22% | 10-250 | 0.9955 | -6.64% | 10-250 | 0.9935 | -18.18% |
| 94 | Dichlobenil | N.D | N.D | N.D | N.D | N.D | N.D | 10-250 | 0.9677 | 3.94% | 20-250 | 0.9992 | 10.50% |
| 95 | Pebulate | 10-250 | 0.9974 | -17.51% | 10-250 | 0.9996 | -10.16% | 10-250 | 0.9442 | -6.39% | 10-250 | 0.9991 | 3.96% |
| 96 | Nitrapyrin | N.D | N.D | N.D | N.D | N.D | N.D | 10-250 | 0.9914 | -6.19% | 10-250 | 0.9974 | -3.50% |
| 97 | Mevinphos | 10-250 | 0.9996 | -4.60% | 10-250 | 0.9970 | 1.96% | 10-250 | 0.9986 | -5.53% | 10-250 | 0.9986 | -5.87% |
| 98 | Chloroneb | 10-250 | 0.9922 | -0.87% | 10-250 | 0.9931 | -6.75% | 10-250 | 0.9980 | -8.49% | 10-250 | 0.9928 | -11.13% |
| 99 | Tecnazene | N.D | N.D | N.D | N.D | N.D | N.D | 10-250 | 0.9924 | -6.72% | 10-250 | 0.9923 | -2.62% |
| 100 | Heptenophos | 10-250 | 0.9975 | -2.38% | 10-250 | 0.9915 | -3.25% | 10-250 | 0.9973 | -5.03% | 10-250 | 1.0000 | 6.82% |
| 101 | Hexachlorobenzene | N.D | N.D | N.D | N.D | N.D | N.D | 20-250 | 0.9937 | -6.67% | 20-250 | 0.9958 | -10.68% |
| 102 | Ethoprophos | 10-250 | 0.9982 | -7.99% | 10-250 | 0.9936 | -1.40% | 10-250 | 0.9966 | 3.31% | 10-250 | 1.0000 | -0.25% |
| 103 | Diallate | 10-250 | 0.9954 | -9.42% | 10-250 | 0.9987 | -6.15% | 10-250 | 0.9937 | -12.29% | 10-250 | 0.9970 | -7.95% |
| 104 | Propachlor | 10-250 | 0.9978 | -7.11% | 10-250 | 0.9940 | -8.67% | 10-250 | 0.9951 | -9.08% | 10-250 | 0.9989 | 2.76% |
| 105 | Triﬂuralin | 10-250 | 0.9965 | -10.61% | 10-250 | 1.0000 | -5.53% | 10-250 | 0.9994 | -7.92% | 10-250 | 0.9996 | -10.61% |
| 106 | Chlorpropham | 10-250 | 0.9925 | -4.46% | 10-250 | 0.9983 | -1.63% | 10-250 | 0.9974 | 8.85% | 10-250 | 0.9999 | -7.52% |
| 107 | Sulfotep | 10-250 | 0.9928 | -3.28% | 10-250 | 0.9923 | -7.33% | 10-250 | 0.9997 | -8.24% | 10-250 | 0.9992 | -6.48% |
| 108 | Sulfallate | 10-250 | 0.9997 | -4.71% | 10-250 | 0.9957 | -7.08% | 10-250 | 0.9993 | -1.57% | 10-250 | 0.9928 | -1.46% |
| 109 | α-BHC | 10-250 | 0.9951 | -13.47% | 10-250 | 0.9952 | -12.83% | 10-250 | 0.9971 | -3.65% | 10-250 | 0.9997 | -0.90% |
| 110 | Terbufos | 10-250 | 0.9992 | 5.18% | 10-250 | 0.9954 | 16.63% | 10-150 | 0.9986 | 23.43% | 10-150 | 0.9976 | 32.59% |
| 111 | Terbumeton | 10-250 | 0.9926 | -23.83% | 10-250 | 0.9903 | 4.75% | 10-150 | 0.9940 | -21.11% | 10-250 | 0.9960 | -9.13% |
| 112 | Profluralin | 10-250 | 0.9989 | -11.87% | 10-250 | 0.9907 | -12.24% | 10-250 | 0.9938 | 0.44% | 10-250 | 0.9982 | -9.96% |
| 113 | Dioxathion | 10-250 | 0.9927 | 9.41% | 10-250 | 0.9912 | 1.77% | 10-250 | 0.9983 | 0.49% | 10-250 | 0.9992 | 6.61% |
| 114 | Propazine | 10-250 | 0.9924 | 17.95% | 10-100 | 0.9922 | 54.02% | 10-250 | 0.9954 | 16.11% | 10-250 | 0.9964 | 18.64% |
| 115 | Chlorbufam | N.D | N.D | N.D | N.D | N.D | N.D | 10-250 | 0.9983 | 10.21% | 10-250 | 0.9928 | 8.77% |
| 116 | Dicloran | 10-250 | 0.9915 | 45.78% | 50-250 | 0.9938 | 69.78% | 20-250 | 0.9974 | -10.27% | 20-250 | 0.9914 | 19.77% |
| 117 | Terbuthylazine | 10-250 | 0.9905 | 30.17% | 10-250 | 0.9922 | 19.19% | 10-250 | 0.9949 | 14.45% | 10-250 | 0.9982 | -5.35% |
| 118 | Monolinuron | 10-250 | 0.9984 | -13.47% | 10-250 | 0.9922 | -2.33% | 10-250 | 0.9998 | -4.15% | 10-250 | 0.9995 | -0.31% |
| 119 | Flufenoxuron | 10-250 | 0.9951 | -5.79% | 10-250 | 0.9966 | -4.85% | 10-250 | 0.9959 | -5.83% | 10-250 | 0.9991 | 7.38% |
| 120 | Cyanophos | 10-250 | 0.9953 | -12.37% | 10-250 | 0.9992 | -3.40% | 10-250 | 0.9998 | 5.02% | 10-250 | 0.9915 | 4.92% |
| 121 | Chlorpyrifos-methyl | 10-250 | 0.9948 | -3.32% | 10-250 | 0.9968 | -0.52% | 10-250 | 0.9918 | -8.14% | 10-250 | 0.9904 | 5.77% |
| 122 | Desmetryn | 10-250 | 0.9971 | 17.55% | 10-250 | 0.9980 | 2.32% | 10-250 | 0.9926 | 10.55% | 10-250 | 0.9951 | 0.77% |
| 123 | Dimethachlor | 10-250 | 0.9940 | -4.20% | 10-250 | 0.9976 | -4.60% | 10-250 | 0.9969 | 3.73% | 10-250 | 1.0000 | 8.54% |
| 124 | Alachlor | 10-250 | 0.9990 | -17.70% | 10-250 | 0.9936 | -23.15% | 10-250 | 0.9938 | -18.67% | 10-250 | 1.0000 | -16.34% |
| 125 | Pirimiphos-methyl | 10-250 | 0.9972 | -16.93% | 10-250 | 0.9963 | -9.43% | 10-250 | 0.9966 | 16.23% | 10-250 | 0.9991 | -5.14% |
| 126 | Terbutryn | 10-250 | 0.9936 | -14.75% | 10-250 | 0.9946 | 6.95% | 10-250 | 0.9981 | 12.79% | 10-250 | 0.9957 | -20.45% |
| 127 | Thiobencarb | 10-250 | 0.9947 | -18.14% | 10-250 | 0.9900 | -24.65% | 10-250 | 0.9993 | 11.96% | 10-250 | 0.9994 | -2.79% |
| 128 | Aspon | 10-250 | 0.9948 | -8.64% | 10-250 | 0.9999 | -4.91% | 10-250 | 0.9953 | -5.61% | 10-250 | 0.9998 | -3.74% |
| 129 | Dicofol | 10-250 | 0.9925 | -0.14% | 10-250 | 0.9957 | -8.02% | 10-250 | 0.9901 | 10.71% | 10-250 | 0.9975 | -16.54% |
| 130 | Metolachlor | 10-250 | 0.9947 | 27.49% | 10-250 | 0.9969 | -3.42% | 10-250 | 0.9950 | 5.35% | 10-250 | 0.9968 | -7.82% |
| 131 | Oxychlordane | 10-250 | 0.9989 | -7.65% | 10-250 | 0.9958 | -9.07% | 10-250 | 0.9939 | -13.14% | 10-250 | 0.9963 | 17.79% |
| 132 | Pirimiphos-ethyl | 10-250 | 0.9915 | -1.32% | 10-250 | 0.9948 | 4.14% | 10-250 | 0.9983 | 0.98% | 10-250 | 0.9953 | 0.22% |
| 133 | Methoprene | 10-250 | 0.9961 | -1.63% | 10-250 | 0.9932 | -3.23% | 10-250 | 0.9905 | -9.89% | 10-250 | 1.0000 | -7.84% |
| 134 | Bromophos | 10-250 | 0.9957 | -15.28% | 10-250 | 0.9948 | -8.70% | 10-250 | 0.9956 | 16.98% | 10-250 | 0.9994 | -16.33% |
| 135 | Dichlofluanid | 10-250 | 0.9938 | -2.99% | 10-250 | 0.9976 | -9.64% | 10-250 | 0.9937 | 0.04% | 10-250 | 0.9997 | 3.69% |
| 136 | Ethofumesate | 10-250 | 0.9968 | -7.73% | 10-250 | 0.9996 | -13.99% | 10-250 | 0.9996 | 2.82% | 10-250 | 0.9945 | 12.19% |
| 137 | Isopropalin | 10-250 | 0.9966 | -14.10% | 10-250 | 0.9963 | -8.47% | 10-250 | 0.9971 | 1.26% | 10-250 | 0.9996 | 19.20% |
| 138 | α-Endosulfan | 10-250 | 0.9996 | 13.16% | 10-250 | 0.9947 | 6.82% | 50-250 | 0.9931 | -15.46% | 50-250 | 0.9959 | -8.61% |
| 139 | Propanil | 10-250 | 0.9990 | -4.97% | 10-250 | 0.9995 | 2.42% | 10-250 | 0.9997 | 4.76% | 10-250 | 0.9995 | 6.41% |
| 140 | Isofenphos | N.D | N.D | N.D | N.D | N.D | N.D | 10-250 | 0.9929 | 13.86% | 10-250 | 0.9914 | -1.77% |
| 141 | Crufomate | 10-250 | 0.9906 | -1.24% | 10-250 | 0.9957 | 12.37% | 10-250 | 0.9925 | 3.26% | 10-250 | 0.9904 | 9.18% |
| 142 | Chlorfenvinphos | 10-250 | 0.9961 | -7.67% | 10-250 | 0.9918 | -19.99% | 10-250 | 0.9966 | -9.17% | 10-250 | 0.9997 | 3.15% |
| 143 | cis-Chlordane (α) | 10-250 | 0.9986 | -8.22% | 10-250 | 0.9983 | -15.72% | 10-250 | 0.9937 | -10.84% | 10-250 | 0.9921 | 12.55% |
| 144 | Tolylfluanid | 10-250 | 0.9925 | -12.02% | 10-250 | 0.9959 | -10.25% | 50-250 | 0.9961 | 34.04% | 10-250 | 0.9993 | -15.48% |
| 145 | 4,4'-DDE | 10-250 | 0.9960 | -18.36% | 10-250 | 0.9913 | -9.79% | 10-250 | 0.9367 | -17.35% | 10-250 | 0.9995 | -24.98% |
| 146 | Butachlor | 10-250 | 0.9999 | -2.49% | 10-250 | 0.9957 | -4.65% | 10-250 | 0.9952 | -3.39% | 10-250 | 0.9958 | -9.09% |
| 147 | Chlozolinate | 10-250 | 0.9988 | -12.78% | 10-250 | 0.9994 | -3.32% | 10-250 | 0.9974 | -9.24% | 10-250 | 0.9986 | -13.00% |
| 148 | Crotoxyphos | 10-250 | 0.9923 | -2.64% | 10-250 | 0.9959 | 23.55% | 10-250 | 0.9944 | -12.51% | 50-250 | 0.9981 | -27.62% |
| 149 | Iodofenphos | 10-250 | 0.9996 | 4.13% | 10-250 | 0.9972 | -3.33% | 10-250 | 0.9969 | 9.59% | 10-250 | 0.9919 | 4.49% |
| 150 | Z-Tetrachlorvinphos | 10-250 | 0.9957 | 12.86% | 10-250 | 0.9948 | -7.01% | 10-250 | 0.9976 | -4.63% | 10-150 | 0.9978 | 30.74% |
| 151 | Chlorbromuron | 10-250 | 0.9982 | -9.34% | 10-250 | 0.9999 | 10.58% | 10-250 | 0.9986 | 12.61% | 10-250 | 0.9974 | 3.89% |
| 152 | Profenofos | 10-250 | 0.9909 | -10.94% | 10-250 | 0.9920 | -6.87% | 10-250 | 0.9979 | -14.70% | 10-250 | 0.9996 | -4.88% |
| 153 | Flurochloridone | 10-250 | 0.9869 | 0.61% | 10-250 | 0.9933 | -11.11% | 10-250 | 0.9908 | 0.72% | 10-250 | 0.9993 | 0.15% |
| 154 | Buprofezin | 10-250 | 0.9969 | -1.55% | 10-250 | 0.9968 | 0.96% | 10-250 | 0.9980 | 1.59% | 10-250 | 0.9991 | -0.87% |
| 155 | 2,4'-DDD | 10-250 | 0.9988 | -9.76% | 10-250 | 0.9973 | 1.10% | 50-250 | 0.9988 | 53.62% | 50-250 | 0.9924 | 29.71% |
| 156 | Endrin | 10-250 | 0.9935 | 2.50% | 10-250 | 0.9966 | -5.84% | 10-250 | 0.9985 | -14.05% | 10-250 | 0.9908 | -7.60% |
| 157 | Hexaconazole | 10-250 | 0.9957 | -2.15% | 10-250 | 0.9927 | 18.53% | 10-150 | 0.9917 | 32.40% | 10-250 | 0.9982 | -11.31% |
| 158 | Chlorfenson | N.D | N.D | N.D | N.D | N.D | N.D | 10-250 | 0.9972 | -6.64% | 10-250 | 0.9942 | -10.38% |
| 159 | 2,4'-DDT | 10-250 | 0.9935 | -2.48% | 10-250 | 0.9969 | -4.74% | 10-250 | 0.9922 | -3.51% | 10-250 | 0.9941 | -6.82% |
| 160 | Paclobutrazol | 10-250 | 0.9962 | -24.35% | 10-250 | 0.9904 | -2.65% | 10-250 | 0.9971 | 4.38% | 10-250 | 0.9987 | 1.61% |
| 161 | Methoprotryne | 10-250 | 0.9917 | -3.59% | 10-250 | 0.9985 | -7.02% | 10-250 | 0.9979 | 1.88% | 10-250 | 0.9950 | 13.07% |
| 162 | Erbon | 10-250 | 0.9995 | -7.81% | 10-250 | 0.9992 | -5.21% | 10-250 | 0.9976 | -5.23% | 10-250 | 0.9977 | -0.96% |
| 163 | Chloropropylate | 10-250 | 0.9948 | -5.46% | 10-250 | 0.9964 | -13.66% | 10-250 | 0.9979 | -2.18% | 10-250 | 0.9961 | -9.02% |
| 164 | Flamprop-methyl | 10-250 | 0.9981 | -8.55% | 10-250 | 0.9963 | -5.45% | 10-250 | 0.9962 | 2.02% | 10-250 | 0.9963 | 10.91% |
| 165 | Nitrofen | 10-250 | 0.9903 | -6.52% | 10-250 | 0.9986 | 2.13% | 10-250 | 0.9914 | -10.77% | 10-250 | 0.9957 | 19.15% |
| 166 | Oxyﬂuorfen | 10-250 | 0.9991 | -3.94% | 10-250 | 0.9987 | -5.12% | 10-250 | 0.9924 | 2.56% | 10-250 | 0.9969 | 5.83% |
| 167 | Chlorthiophos | 10-250 | 0.9996 | -7.78% | 10-250 | 0.9904 | -4.66% | 10-250 | 0.9988 | -8.56% | 10-250 | 0.9999 | -0.93% |
| 168 | β-Endosulfan | 10-250 | 0.9987 | -1.99% | 10-250 | 0.9911 | -7.24% | 10-250 | 0.9974 | -19.27% | 10-250 | 0.9950 | 16.67% |
| 169 | Flamprop-isopropyl | 10-250 | 0.9987 | -1.41% | 10-250 | 0.9948 | -4.56% | 10-250 | 0.9942 | -0.33% | 10-250 | 0.9990 | 12.23% |
| 170 | 4,4'-DDT | 10-250 | 0.9934 | -4.69% | 10-250 | 0.9973 | -4.18% | 10-250 | 0.9944 | 11.51% | 10-250 | 0.9979 | 1.30% |
| 171 | Carbophenothion | 10-250 | 0.9956 | -18.86% | 10-250 | 0.9953 | -5.80% | 10-250 | 0.9942 | -17.87% | 10-250 | 0.9952 | 12.53% |
| 172 | Benalaxyl | 10-250 | 0.9992 | -9.45% | 10-250 | 0.9969 | 9.43% | 20-250 | 0.9955 | 13.07% | 20-250 | 0.9948 | -2.60% |
| 173 | Edifenphos | 10-250 | 0.9506 | -2.13% | 10-250 | 0.9999 | -2.98% | 10-250 | 0.9913 | 8.01% | 10-250 | 0.9949 | -3.94% |
| 174 | Triazophos | 10-250 | 0.9998 | -0.47% | 10-250 | 0.9998 | 20.15% | 10-250 | 0.9971 | -15.55% | 10-250 | 1.0000 | -7.32% |
| 175 | Cyanofenphos | 10-250 | 0.9993 | -5.25% | 10-250 | 0.9902 | 12.79% | 10-250 | 0.9958 | 6.11% | 10-250 | 0.9944 | 14.02% |
| 176 | Chlorbenside sulfone | 10-250 | 0.9982 | -8.63% | 10-250 | 0.9901 | 21.50% | N.D | N.D | N.D | N.D | N.D | N.D |
| 177 | Endosulfan Sulfate | 10-250 | 0.9929 | -1.56% | 10-250 | 0.9987 | -6.05% | 10-250 | 0.9948 | -5.35% | 10-250 | 0.9961 | -2.56% |
| 178 | Bromopropylate | N.D | N.D | N.D | 10-250 | 0.9907 | -8.09% | N.D | N.D | N.D | 10-250 | 0.9922 | 3.69% |
| 179 | Benzoylprop-ethyl | 10-250 | 0.9993 | -10.88% | 10-250 | 0.9985 | -6.02% | 10-250 | 0.9956 | 1.59% | 10-250 | 0.9996 | 15.72% |
| 180 | Fenpropathrin | 10-250 | 0.9990 | -5.06% | 10-250 | 0.9915 | -2.93% | 10-250 | 0.9903 | -7.20% | 10-250 | 0.9922 | -1.17% |
| 181 | Leptophos | 10-250 | 0.9956 | -5.64% | 10-250 | 0.9988 | -4.30% | 10-250 | 0.9921 | -3.93% | 10-250 | 0.9990 | -4.58% |
| 182 | EPN | 10-250 | 0.9912 | -7.07% | 10-250 | 0.9965 | -10.78% | 10-250 | 0.9980 | -14.70% | 10-250 | 0.9954 | 10.83% |
| 183 | Hexazinone | 10-250 | 0.9962 | 8.26% | 10-250 | 0.9922 | -8.10% | 10-250 | 0.9941 | 7.37% | 10-250 | 0.9968 | -2.68% |
| 184 | Phosalone | 10-250 | 0.9997 | -4.69% | 10-250 | 0.9991 | -5.40% | 10-250 | 0.9977 | -8.38% | 10-250 | 0.9967 | 11.52% |
| 185 | Azinphos-methyl | 10-250 | 0.9928 | -1.50% | 10-250 | 0.9913 | -6.74% | 10-250 | 0.9974 | -6.50% | 10-250 | 0.9911 | 5.66% |
| 186 | Fenarimol | 10-250 | 0.9966 | -2.57% | 10-250 | 0.9926 | -1.42% | 10-250 | 0.9978 | -5.00% | 10-250 | 0.9993 | 6.42% |
| 187 | Azinphos-ethyl | 10-250 | 0.9916 | 8.17% | N.D | N.D | N.D | 20-250 | 0.9997 | -14.45% | 10-250 | 0.9975 | 2.05% |
| 188 | Prochloraz | 10-250 | 0.9945 | -8.73% | 10-250 | 0.9989 | -7.16% | 10-250 | 0.9963 | -1.03% | 10-250 | 0.9979 | 9.03% |
| 189 | Coumaphos | 10-250 | 0.9929 | -6.70% | 10-250 | 0.9996 | -11.72% | 10-250 | 0.9922 | 1.28% | 10-250 | 0.9971 | -12.63% |
| 190 | Cyfluthrin | N.D | N.D | N.D | N.D | N.D | N.D | 50-250 | 0.9926 | -1.32% | 50-250 | 0.9927 | -11.14% |
| 191 | tau-Fluvalinate | 20-250 | 0.9969 | -6.49% | 10-250 | 0.9997 | 1.19% | 10-250 | 0.9933 | -15.07% | 10-250 | 0.9987 | 19.46% |
| 192 | Dichlorvos | 10-250 | 0.9968 | -5.08% | 10-250 | 0.9990 | -4.62% | 10-250 | 0.9969 | -10.86% | 10-250 | 0.9921 | -24.86% |
| 193 | Biphenyl | 10-250 | 0.9987 | -5.39% | 10-250 | 0.9927 | -4.84% | 10-250 | 0.9992 | -1.20% | 10-250 | 0.9931 | 0.17% |
| 194 | Vernolate | 10-250 | 0.9958 | -6.29% | 10-250 | 0.9983 | 6.99% | 10-250 | 0.9906 | -15.99% | 10-250 | 0.9948 | 0.38% |
| 195 | 3,5-Dichloroaniline | N.D | N.D | N.D | 20-250 | 0.9957 | -9.06% | 20-250 | 0.9901 | 29.81% | 20-250 | 1.0000 | 23.23% |
| 196 | Molinate | 10-250 | 0.9941 | -20.19% | 10-250 | 0.9931 | -4.29% | 10-250 | 0.9913 | 4.17% | 10-250 | 1.0000 | 5.72% |
| 197 | E-Methacrifos | 10-250 | 0.9948 | -21.33% | 10-250 | 0.9955 | 13.69% | 10-250 | 0.9997 | -13.88% | 10-250 | 0.9967 | -12.72% |
| 198 | o-Phenylphenol | 10-250 | 0.9921 | -5.07% | 10-250 | 0.9987 | -15.45% | 10-250 | 0.9997 | -13.26% | 10-250 | 0.9901 | 1.39% |
| 199 | cis-1,2,3,6-Tetrahydrophthalimide | N.D | N.D | N.D | N.D | N.D | N.D | 10-250 | 0.9922 | -14.41% | 10-250 | 0.9974 | 14.67% |
| 200 | Fenobucarb | 10-250 | 0.9910 | 7.03% | 10-250 | 0.9990 | 14.13% | 10-250 | 0.9985 | 0.08% | 10-250 | 0.9953 | 5.06% |
| 201 | Benﬂuralin | 10-250 | 0.9912 | -1.87% | 10-250 | 0.9928 | -8.91% | 10-250 | 0.9985 | -1.59% | 10-250 | 0.9986 | 2.00% |
| 202 | Hexaflumuron | 10-250 | 0.9979 | -18.56% | 10-250 | 0.9964 | -19.99% | 10-250 | 0.9905 | -3.89% | 10-250 | 0.9934 | -1.38% |
| 203 | Prometon | 10-250 | 0.9970 | -10.34% | 10-250 | 0.9908 | 1.19% | 10-250 | 1.0000 | -9.87% | 10-250 | 0.9993 | -13.00% |
| 204 | Triallate | 10-250 | 0.9945 | -18.48% | 10-250 | 0.9921 | -10.68% | 10-250 | 0.9999 | 9.22% | 10-250 | 0.9969 | -8.62% |
| 205 | Pyrimethanil | 10-250 | 0.9918 | -2.20% | 10-250 | 0.9966 | -2.16% | 10-250 | 0.9973 | 4.01% | 10-250 | 0.9988 | 2.63% |
| 206 | γ-HCH | 10-250 | 0.9943 | -8.60% | 10-250 | 0.9912 | -2.00% | 10-250 | 0.9943 | -3.79% | 10-250 | 0.9928 | -4.66% |
| 207 | Disulfoton | 10-250 | 0.9965 | 5.45% | 10-250 | 0.9952 | 6.50% | 10-250 | 0.9929 | 18.99% | 10-150 | 0.9953 | 36.60% |
| 208 | Atrazine | 10-250 | 0.9962 | -26.48% | 10-250 | 0.9995 | -6.65% | 10-250 | 0.9999 | -20.97% | 10-250 | 0.9981 | -11.45% |
| 209 | Heptachlor | N.D | N.D | N.D | N.D | N.D | N.D | 10-250 | 0.9994 | -2.01% | 10-250 | 0.9999 | -9.03% |
| 210 | Iprobenfos | 10-250 | 0.9959 | -7.69% | 10-250 | 0.9960 | -1.19% | 10-250 | 0.9952 | -4.79% | 10-250 | 0.9995 | -4.52% |
| 211 | Isazofos | 10-250 | 0.9971 | -1.22% | 10-250 | 0.9974 | -5.98% | 10-250 | 0.9967 | -0.10% | 10-250 | 0.9917 | -1.70% |
| 212 | Plifenate | 10-250 | 0.9913 | -13.21% | 10-250 | 0.9989 | 17.53% | 20-250 | 0.9999 | 45.82% | 20-250 | 0.9984 | 21.82% |
| 213 | Fenpropimorph | 10-250 | 0.9959 | -19.37% | 10-250 | 0.9913 | -11.96% | 10-250 | 1.0000 | 8.44% | 10-250 | 0.9998 | -8.86% |
| 214 | Transfluthrin | 10-250 | 0.9956 | -9.48% | 10-250 | 0.9993 | -2.78% | 10-250 | 0.9989 | -13.23% | 10-250 | 0.9999 | 1.22% |
| 215 | Fluchloralin | 10-250 | 0.9984 | -0.44% | 10-250 | 0.9987 | 10.08% | 10-250 | 0.9987 | 5.02% | 10-250 | 0.9959 | 5.72% |
| 216 | Tolclofos-methyl | 10-250 | 0.9937 | -1.66% | 10-250 | 0.9931 | -1.02% | 10-250 | 0.9972 | -9.93% | 10-250 | 0.9938 | -8.67% |
| 217 | Propisochlor | 10-250 | 0.9882 | -9.61% | 10-250 | 0.9944 | 2.48% | 10-250 | 0.9992 | 6.31% | 10-150 | 0.9976 | 38.75% |
| 218 | Ametryn | 10-250 | 0.9999 | -5.38% | 10-250 | 0.9979 | 2.37% | 10-250 | 0.9995 | -8.25% | 10-250 | 0.9981 | -10.06% |
| 219 | Simetryn | 10-250 | 0.9902 | -10.88% | 10-250 | 0.9917 | 6.18% | 10-250 | 0.9971 | 6.80% | 10-250 | 0.9980 | -11.88% |
| 220 | Metobromuron | 10-250 | 0.9974 | -14.81% | 10-250 | 0.9992 | 14.60% | 10-250 | 0.9990 | -1.36% | 10-250 | 0.9955 | 5.04% |
| 221 | Metribuzin | 10-250 | 0.9964 | -15.09% | 10-250 | 0.9933 | 25.15% | 10-250 | 0.9992 | 19.42% | 10-250 | 0.9942 | -6.39% |
| 222 | Dimethipin | 10-250 | 0.9928 | -8.12% | 10-250 | 0.9922 | -1.64% | 10-250 | 0.9973 | 0.18% | 10-250 | 0.9984 | 4.60% |
| 223 | ε-HCH | 10-250 | 0.9938 | -22.71% | 10-250 | 0.9970 | 13.19% | 20-250 | 0.9995 | 27.95% | 20-250 | 0.9962 | -14.37% |
| 224 | Dipropetryn | 10-250 | 0.9962 | 1.51% | 10-250 | 0.9811 | 12.07% | 10-250 | 0.9993 | 2.63% | 10-250 | 0.9975 | -8.38% |
| 225 | Formothion | 10-250 | 0.9978 | 2.52% | 10-250 | 0.9961 | -12.04% | 10-250 | 0.9989 | 17.65% | 10-250 | 0.9921 | 23.99% |
| 226 | Diethofencarb | 10-250 | 0.9975 | -20.45% | 10-250 | 0.9979 | -12.45% | 10-250 | 0.9984 | -12.45% | 10-250 | 0.9940 | -16.76% |
| 227 | Dimepiperate | 10-250 | 0.9931 | -2.43% | 10-250 | 0.9996 | -3.18% | 10-250 | 0.9967 | -3.29% | 10-250 | 0.9982 | -8.44% |
| 228 | Bioallethrin | 10-250 | 0.9958 | -6.37% | 20-250 | 0.9914 | -0.62% | 10-250 | 0.9929 | -4.89% | 10-250 | 0.9924 | -3.93% |
| 229 | 2,4'-DDE | 10-250 | 0.9994 | -18.35% | 10-250 | 0.9949 | -8.08% | 10-250 | 0.9987 | -20.76% | 10-250 | 0.9953 | -12.84% |
| 230 | Fenson | 10-250 | 0.9975 | -1.82% | 10-250 | 0.9964 | 9.07% | 10-250 | 0.9979 | 0.36% | 10-250 | 0.9929 | -10.27% |
| 231 | Diphenamid | 10-250 | 0.9931 | 23.40% | 10-250 | 0.9923 | 5.50% | 10-250 | 0.9918 | -7.91% | 10-250 | 0.9934 | -14.26% |
| 232 | Chlorthion | 10-250 | 0.9964 | -11.86% | 10-250 | 0.9934 | -18.81% | 10-250 | 0.9988 | -21.03% | 10-250 | 0.9964 | -10.79% |
| 233 | Prallethrin | N.D | N.D | N.D | N.D | N.D | N.D | 10-250 | 0.9984 | 4.69% | 10-250 | 0.9922 | 10.43% |
| 234 | Penconazole | 10-250 | 0.9985 | -12.59% | 10-250 | 0.9973 | -3.48% | 10-250 | 0.9997 | -14.78% | 10-250 | 0.9974 | -5.45% |
| 235 | Mecarbam | 10-250 | 0.9941 | -22.16% | 10-250 | 0.9946 | -0.82% | 10-250 | 0.9977 | -18.44% | 10-250 | 0.9904 | -17.07% |
| 236 | Tetraconazole | 10-250 | 0.9906 | -5.78% | 10-250 | 0.9943 | -3.59% | 10-250 | 0.9984 | 10.11% | 10-250 | 0.9939 | -10.39% |
| 237 | Propaphos | 10-250 | 0.9972 | -5.04% | 10-250 | 0.9957 | 5.53% | 10-250 | 0.9975 | 3.15% | 10-250 | 0.9973 | 2.61% |
| 238 | Flumetralin | 10-250 | 0.9932 | -16.99% | 10-250 | 0.9927 | -7.53% | 10-250 | 0.9964 | 1.39% | 20-250 | 0.9987 | -15.32% |
| 239 | Triadimenol | 10-250 | 0.9917 | -5.14% | 10-250 | 0.9909 | -0.46% | 10-250 | 0.9960 | -5.18% | 10-250 | 0.9935 | 5.59% |
| 240 | Pretilachlor | 10-250 | 0.9951 | -2.35% | 10-250 | 0.9946 | -2.28% | 10-250 | 0.9997 | -1.50% | 10-250 | 0.9998 | -1.74% |
| 241 | Kresoxim-methyl | 10-250 | 0.9451 | -4.48% | 10-250 | 0.9931 | -6.37% | 10-250 | 0.9928 | 2.11% | 10-250 | 0.9996 | -5.23% |
| 242 | Fluazifop-butyl | 10-250 | 0.9925 | -19.07% | 10-250 | 0.9914 | -9.30% | 10-150 | 0.9968 | -11.33% | 10-150 | 0.9906 | -4.52% |
| 243 | Chlorfluazuron | 10-250 | 0.9950 | -15.04% | 10-250 | 0.9920 | -8.17% | 10-250 | 0.9984 | 6.73% | 10-250 | 0.9958 | -8.64% |
| 244 | Chlorobenzilate | N.D | N.D | N.D | N.D | N.D | N.D | 10-250 | 0.9986 | -6.93% | 10-250 | 0.9951 | -7.26% |
| 245 | Uniconazole | N.D | N.D | N.D | N.D | N.D | N.D | 10-250 | 0.9996 | -0.76% | 10-250 | 0.9995 | -9.36% |
| 246 | Flusilazole | 10-250 | 0.9984 | -14.67% | 10-250 | 0.9917 | -6.34% | 10-250 | 0.9978 | -12.19% | 10-250 | 0.9952 | -17.27% |
| 247 | Fluorodifen | N.D | N.D | N.D | N.D | N.D | N.D | 20-250 | 0.9928 | 4.77% | 20-250 | 0.9946 | 14.60% |
| 248 | Diniconazole | 10-250 | 0.9979 | -3.59% | 10-250 | 0.9982 | -7.26% | 10-250 | 0.9948 | 7.35% | 10-250 | 0.9987 | 5.18% |
| 249 | Piperonyl butoxide | 10-250 | 0.9925 | -8.27% | 10-250 | 0.9946 | -1.44% | 10-250 | 0.9988 | 4.67% | 10-250 | 0.9988 | 5.19% |
| 250 | Propargite | 10-250 | 0.9998 | -2.00% | 10-250 | 0.9988 | -1.14% | 10-250 | 0.9923 | 8.95% | 10-250 | 0.9990 | 6.89% |
| 251 | Mepronil | 10-250 | 0.9951 | -8.34% | 10-250 | 0.9998 | -2.64% | 10-250 | 0.9995 | 0.63% | 10-250 | 0.9995 | 2.76% |
| 252 | Dimefuron | 10-250 | 0.9918 | -8.65% | 10-250 | 0.9952 | 12.88% | 10-250 | 0.9994 | 9.40% | 10-250 | 0.9979 | 4.21% |
| 253 | Diﬂufenican | 10-250 | 0.9947 | -7.15% | 10-250 | 0.9908 | -4.84% | 10-250 | 0.9926 | -0.61% | 10-250 | 0.9937 | -2.61% |
| 254 | Fenazaquin | 10-250 | 0.9934 | -10.37% | 10-250 | 0.9953 | -8.42% | 10-250 | 0.9982 | -2.28% | 10-250 | 0.9951 | -8.88% |
| 255 | Phenothrin | 10-250 | 0.9935 | -13.88% | 10-250 | 0.9954 | -6.28% | 10-250 | 0.9953 | -15.55% | 10-250 | 0.9940 | -9.27% |
| 256 | Fludioxonil | N.D | N.D | N.D | N.D | N.D | N.D | N.D | N.D | N.D | N.D | N.D | N.D |
| 257 | Fenoxycarb | 10-250 | 0.9919 | -12.55% | 10-250 | 0.9903 | -4.99% | 10-250 | 0.9987 | 9.83% | 10-250 | 0.9926 | 4.59% |
| 258 | Sethoxydim | 10-250 | 0.9973 | -30.12% | 20-250 | 0.9923 | -10.78% | 10-250 | 0.9994 | -11.52% | 10-250 | 1.0000 | -16.88% |
| 259 | Anilofos | 10-250 | 0.9926 | -16.93% | 10-250 | 0.9983 | -14.26% | 10-250 | 0.9999 | 7.03% | 10-250 | 0.9969 | 22.55% |
| 260 | Acrinathrin | N.D | N.D | N.D | 10-250 | 0.9951 | -6.58% | 10-250 | 0.9962 | -17.82% | 10-250 | 0.9912 | -9.08% |
| 261 | λ-Cyhalothrin | N.D | N.D | N.D | N.D | N.D | N.D | 20-250 | 0.9934 | -5.43% | 20-250 | 0.9944 | -22.16% |
| 262 | Mefenacet | 10-250 | 0.9972 | -5.02% | 10-250 | 0.9934 | -1.18% | 10-250 | 0.9966 | -2.72% | 10-250 | 0.9991 | -10.52% |
| 263 | Permethrin | 10-250 | 0.9943 | -2.80% | 10-250 | 0.9918 | -1.34% | 10-250 | 0.9969 | 0.38% | 10-250 | 0.9932 | 1.48% |
| 264 | Pyridaben | 10-250 | 0.9946 | -12.30% | 10-250 | 0.9972 | 12.46% | 10-250 | 0.9990 | 6.70% | 10-250 | 0.9927 | -1.51% |
| 265 | Fluoroglycofen-ethyl | 10-250 | 0.9951 | -17.95% | 10-250 | 0.9935 | -13.30% | 10-250 | 0.9955 | -19.77% | 10-250 | 0.9987 | -10.59% |
| 266 | Bitertanol | 10-250 | 0.9939 | -10.99% | 10-250 | 0.9984 | 4.80% | 10-250 | 0.9998 | -7.10% | 10-250 | 0.9945 | -1.84% |
| 267 | Etofenprox | 10-250 | 0.9963 | -10.84% | 10-250 | 0.9979 | -4.77% | 10-250 | 0.9990 | -2.96% | 10-250 | 0.9986 | 2.69% |
| 268 | Cycloxydim | 10-250 | 0.9972 | -12.81% | 10-250 | 0.9932 | 19.14% | 10-250 | 0.9995 | 18.54% | 10-250 | 0.9997 | -3.77% |
| 269 | α-Cypermethrin | 10-250 | 0.9937 | 9.96% | 10-250 | 0.9933 | -2.45% | 10-250 | 0.9954 | -27.60% | 10-250 | 0.9902 | -8.21% |
| 270 | Flucythrinate | 10-250 | 0.9963 | -5.70% | 10-250 | 0.9984 | -0.44% | 10-250 | 0.9934 | 1.24% | 10-250 | 0.9955 | 4.92% |
| 271 | Esfenvalerate | 10-250 | 0.9972 | -7.15% | 10-250 | 0.9932 | -7.50% | 20-250 | 0.9961 | -5.60% | 20-250 | 0.9984 | -8.85% |
| 272 | Difenoconazole | 10-250 | 0.9987 | -5.67% | 10-250 | 0.9976 | 4.16% | 10-250 | 0.9994 | -1.51% | 10-250 | 0.9965 | 3.56% |
| 273 | Flumioxazin | 10-250 | 0.9933 | -17.50% | 10-250 | 0.9933 | -5.14% | 10-250 | 0.9904 | 6.82% | 10-250 | 0.9950 | 15.98% |
| 274 | Flumiclorac-pentyl | 10-250 | 0.9974 | -14.37% | 10-250 | 0.9915 | -7.86% | 10-250 | 0.9905 | -3.26% | 10-250 | 0.9999 | -5.74% |
| 275 | Dimefox | 10-250 | 0.9961 | -3.09% | 10-250 | 0.9942 | -2.87% | 10-250 | 0.9966 | 9.79% | 10-250 | 0.9914 | -7.12% |
| 276 | Disulfoton-sulfoxide | 10-250 | 0.9941 | -6.75% | 10-250 | 0.9949 | -4.42% | 10-250 | 1.0000 | 12.35% | 10-250 | 0.9981 | 12.20% |
| 277 | Pentachlorobenzene | 10-250 | 0.9925 | -4.95% | 10-250 | 0.9919 | -9.80% | 10-250 | 0.9976 | -2.71% | 10-250 | 0.9964 | -3.17% |
| 278 | Triisobutyl phosphate | 10-250 | 0.9931 | -14.07% | 10-250 | 0.9960 | -1.63% | 10-250 | 0.9991 | -12.63% | 10-250 | 0.9938 | 0.78% |
| 279 | Crimidine | 10-250 | 0.9938 | -5.91% | 10-250 | 0.9934 | -3.49% | 10-250 | 0.9995 | -2.49% | 10-250 | 0.9996 | -6.74% |
| 280 | BDMC-1 | 10-250 | 0.9931 | 0.31% | 10-250 | 0.9977 | 20.97% | 20-250 | 0.9908 | 13.50% | 20-250 | 0.9937 | 13.85% |
| 281 | Chlorfenprop-methyl | 10-250 | 0.9924 | -3.32% | 10-250 | 0.9924 | -2.52% | 10-250 | 0.9981 | -3.45% | 10-250 | 0.9965 | 3.45% |
| 282 | Thionazin | 10-250 | 0.9903 | -10.63% | 10-250 | 0.9910 | 7.13% | 10-250 | 0.9902 | -8.63% | 10-250 | 0.9934 | -14.29% |
| 283 | 2,3,5,6-tetrachloroaniline | 10-250 | 0.9973 | 13.92% | 10-250 | 0.9925 | 6.39% | 10-250 | 0.9910 | -16.24% | 10-250 | 0.9935 | -9.23% |
| 284 | Tributyl phosphate | 10-250 | 0.9962 | -18.40% | 10-250 | 0.8324 | -5.77% | 10-250 | 1.0000 | -8.69% | 10-250 | 0.9992 | -20.16% |
| 285 | 2,3,4,5-Tetrachloroanisole | 10-250 | 0.9949 | -4.06% | 10-250 | 0.9927 | -1.47% | 10-250 | 0.9961 | 7.66% | 10-250 | 0.9997 | 10.33% |
| 286 | Pentachloroanisole | 10-250 | 0.9967 | -2.86% | 10-250 | 0.9933 | -15.90% | 10-150 | 1.0000 | -5.66% | 10-150 | 1.0000 | -12.05% |
| 287 | Tebutam | 10-250 | 0.9974 | 7.27% | 10-250 | 0.9918 | -2.85% | 10-250 | 0.9958 | -4.60% | 10-250 | 0.9926 | -2.11% |
| 288 | Dioxabenzofos | 10-250 | 0.9987 | -0.33% | 10-250 | 0.9981 | -2.95% | 10-250 | 0.9972 | 3.25% | 10-250 | 0.9953 | 0.26% |
| 289 | Methabenzthiazuron | 10-250 | 0.9987 | -9.00% | 10-250 | 0.9907 | -4.45% | 10-150 | 1.0000 | 36.87% | 10-250 | 1.0000 | 12.46% |
| 290 | Simeton | 10-250 | 0.9927 | -0.13% | 10-250 | 0.9972 | -0.72% | 10-250 | 0.9996 | 5.51% | 10-250 | 0.9951 | 1.91% |
| 291 | Atratone | 10-250 | 0.9928 | -5.03% | 10-250 | 0.9976 | 5.24% | 10-250 | 0.9977 | -20.61% | 10-250 | 0.9956 | 0.62% |
| 292 | Atrazine-desisopropyl | 10-250 | 0.9964 | -0.74% | 10-250 | 0.9933 | 0.17% | 10-250 | 0.9969 | -0.01% | 10-250 | 0.9990 | 6.65% |
| 293 | Terbufos sulfone | 10-250 | 0.9959 | -24.80% | 10-250 | 0.9989 | -7.15% | 10-250 | 0.9993 | 6.59% | 10-250 | 0.9983 | -20.15% |
| 294 | Tefluthrin | 10-250 | 0.9938 | -3.48% | 10-250 | 0.9947 | 2.65% | 50-250 | 0.9999 | -21.93% | 50-250 | 0.9996 | -6.36% |
| 295 | Bromocylen | 10-250 | 0.9956 | -15.64% | 20-250 | 0.9985 | -2.67% | 10-250 | 0.9932 | -13.27% | 10-250 | 0.9926 | -24.97% |
| 296 | Trietazine | 10-250 | 0.9957 | -7.20% | 10-250 | 0.9986 | -3.89% | 10-250 | 0.9922 | -7.99% | 10-250 | 0.9904 | -4.89% |
| 297 | Cycluron | 10-250 | 0.9914 | -4.96% | 10-250 | 0.9969 | -7.84% | 10-250 | 0.9999 | -12.54% | 10-250 | 0.9997 | -1.79% |
| 298 | 2,6-Dichlorobenzamide | 10-250 | 0.9943 | -15.94% | 10-250 | 0.9942 | -2.94% | 10-250 | 0.9983 | 12.12% | 10-250 | 1.0000 | -3.97% |
| 299 | 2,4,4'-Trichlorobiphenyl | 10-250 | 0.9961 | -3.18% | 10-250 | 0.9972 | 6.00% | 10-250 | 0.9980 | -7.78% | 10-250 | 0.9917 | 5.66% |
| 300 | 2,4,5-Trichlorobiphenyl | 10-250 | 0.9928 | -6.23% | 10-250 | 0.9966 | -2.24% | 10-250 | 0.9949 | 3.52% | 10-250 | 0.9987 | 4.71% |
| 301 | Sebuthylazine-desethyl | 10-250 | 0.9916 | -12.29% | 10-250 | 0.9952 | -6.63% | 10-250 | 0.9995 | 10.59% | 10-250 | 0.9993 | -12.05% |
| 302 | 2,3,4,5-Tetrachloroaniline | 10-250 | 0.9916 | -16.41% | 10-250 | 0.9957 | -3.84% | 10-250 | 0.9984 | 5.99% | 10-250 | 0.9901 | -6.52% |
| 303 | musk ambrette | 10-250 | 0.9912 | -4.27% | 10-250 | 0.9968 | 0.09% | 10-250 | 0.9994 | -1.67% | 10-250 | 1.0000 | -10.99% |
| 304 | Musk xylene | 10-250 | 0.9928 | -13.92% | 10-250 | 0.9948 | -2.78% | 10-250 | 0.9997 | -9.42% | 10-250 | 0.9908 | -19.35% |
| 305 | Pentachloroaniline | 10-250 | 0.9945 | -6.33% | 10-250 | 0.9913 | -1.91% | 10-250 | 0.9966 | 1.74% | 10-250 | 0.9946 | 5.88% |
| 306 | Aziprotryne | 10-250 | 0.9952 | -1.65% | 10-250 | 0.9966 | -4.61% | 10-250 | 0.9995 | 2.15% | 10-250 | 0.9964 | -8.98% |
| 307 | Sebuthylazine | 10-250 | 0.9970 | -11.21% | 10-250 | 0.9915 | -5.66% | 10-250 | 0.9984 | 3.54% | 10-250 | 0.9958 | 10.10% |
| 308 | Isocarbamid | 10-250 | 0.9999 | -12.21% | 10-250 | 0.9946 | 9.60% | 10-250 | 0.9986 | 3.24% | 10-250 | 0.9935 | -0.52% |
| 309 | 2,2',5,5'-Tetrachlorobiphenyl | 10-250 | 0.9938 | -5.51% | 10-250 | 0.9944 | -1.66% | 10-250 | 0.9987 | -3.17% | 10-250 | 0.9936 | -4.39% |
| 310 | Musk moskene | 10-250 | 0.9937 | -17.90% | 10-250 | 0.9987 | -10.25% | 10-250 | 0.9926 | 10.75% | 10-250 | 0.9922 | -11.65% |
| 311 | Prosulfocarb | 10-250 | 0.9922 | -3.06% | 10-250 | 0.9959 | -1.58% | 10-250 | 1.0000 | 4.39% | 10-250 | 0.9971 | 2.34% |
| 312 | Dimethenamid | 10-250 | 0.9956 | -7.40% | 10-250 | 0.9923 | 15.61% | 10-250 | 0.9980 | -12.61% | 10-250 | 0.9917 | -13.72% |
| 313 | Fenchlorphos-oxon | 10-250 | 0.9912 | -6.66% | 10-250 | 0.9927 | -5.73% | 10-250 | 0.9988 | 0.69% | 10-250 | 0.9944 | -5.97% |
| 314 | Paraoxon-methyl | 10-250 | 0.9963 | -5.75% | 10-250 | 0.9974 | -1.95% | 10-250 | 0.9993 | 4.32% | 10-250 | 1.0000 | 2.77% |
| 315 | Monalide | 10-250 | 0.9989 | -11.63% | 10-250 | 0.9949 | -6.29% | 10-250 | 0.9995 | -2.86% | 10-250 | 0.9907 | -16.30% |
| 316 | Tibetene musk | 10-250 | 0.9973 | -2.41% | 10-250 | 0.9914 | -13.18% | 10-250 | 0.9903 | -5.70% | 10-250 | 0.9982 | -6.80% |
| 317 | Isobenzan | 10-250 | 0.9927 | -25.84% | 10-250 | 0.9942 | 9.84% | 10-250 | 0.9936 | -8.65% | 10-250 | 0.9923 | -4.75% |
| 318 | Octachlorostyrene | 10-250 | 0.9942 | -10.10% | 10-250 | 0.9963 | -2.50% | 20-250 | 0.9997 | -8.24% | 20-250 | 0.9968 | -3.25% |
| 319 | Pyrimitate | 10-250 | 0.9988 | 8.17% | 10-250 | 0.9950 | 5.96% | 10-250 | 0.9989 | 16.40% | 10-250 | 0.9997 | 8.08% |
| 320 | Isodrin | 10-250 | 0.9963 | -19.47% | 10-250 | 0.9927 | -12.63% | 10-250 | 0.9954 | 14.86% | 10-250 | 0.9968 | 5.64% |
| 321 | Isomethiozin | 10-250 | 0.9948 | -7.71% | 10-250 | 0.9974 | 0.71% | 10-250 | 0.9908 | -3.66% | 10-250 | 0.9987 | -9.14% |
| 322 | Trichloronate | 10-250 | 0.9963 | -11.77% | 10-250 | 0.9963 | -5.89% | 10-250 | 0.9976 | 5.84% | 10-250 | 0.9903 | 2.63% |
| 323 | Chlorthal-dimethyl | 10-250 | 0.9970 | -16.48% | 10-250 | 0.9921 | -6.95% | 10-250 | 0.9931 | 27.45% | 10-250 | 0.9994 | -19.58% |
| 324 | 4,4'-Dichlorobenzophenone | 10-250 | 0.9977 | -7.90% | 10-250 | 0.9953 | -10.95% | 10-250 | 1.0000 | -16.80% | 10-250 | 0.9983 | -7.84% |
| 325 | Nitrothal-isopropyl | 10-250 | 0.9968 | -18.12% | 10-250 | 0.9921 | -9.04% | 10-250 | 0.9986 | 33.97% | 10-250 | 0.9978 | -2.52% |
| 326 | Musk ketone | N.D | N.D | N.D | N.D | N.D | N.D | 20-250 | 0.9996 | 12.11% | 20-250 | 0.9982 | -10.42% |
| 327 | Rabenzazol | 10-250 | 0.9934 | -29.23% | 10-250 | 0.9973 | -6.33% | 10-250 | 0.9996 | 2.90% | 10-250 | 0.9991 | -17.87% |
| 328 | Cyprodinil | 10-250 | 0.9986 | -7.01% | 10-250 | 0.9989 | -17.22% | 10-250 | 0.9982 | 8.36% | 10-250 | 0.9959 | -0.03% |
| 329 | Fuberidazole | 10-250 | 0.9964 | -24.06% | 10-250 | 0.9959 | -7.95% | 10-250 | 0.9994 | -7.88% | 10-250 | 0.9951 | -18.91% |
| 330 | Isofenphos-oxon | 10-250 | 0.9965 | -8.01% | 10-250 | 0.9953 | -29.43% | 10-250 | 0.9968 | 26.17% | 10-250 | 0.9970 | -21.36% |
| 331 | Dicapthon | 10-250 | 0.9908 | -30.50% | 10-250 | 0.9936 | -25.03% | 10-250 | 0.9989 | -12.64% | 10-250 | 0.9996 | 26.98% |
| 332 | 2,2',4,5,5'-Pentachlorobiphenyl | 10-250 | 0.9918 | -17.75% | 10-250 | 0.9963 | -12.04% | 10-250 | 0.9986 | 13.63% | 10-250 | 0.9987 | 12.39% |
| 333 | MCPA-butoxyethyl ester | 10-250 | 0.9912 | -13.99% | 10-250 | 0.9957 | -3.79% | 10-250 | 0.9903 | 16.73% | 10-250 | 0.9999 | 1.33% |
| 334 | Isocarbophos | 10-250 | 0.9989 | -5.70% | 10-250 | 0.9977 | -1.53% | 50-250 | 0.9937 | 19.22% | 50-250 | 0.9948 | 15.91% |
| 335 | Phorate sulfone | 10-250 | 0.9987 | -4.02% | 10-250 | 0.9967 | -2.29% | 10-250 | 0.9959 | 3.31% | 10-250 | 0.9970 | 10.24% |
| 336 | Chlorfenethol | 10-250 | 0.9941 | -16.84% | 10-250 | 0.9938 | -11.61% | 10-250 | 0.9956 | -12.28% | 10-250 | 0.9973 | -14.49% |
| 337 | trans-Nonachlor | 10-250 | 0.9992 | -18.23% | 10-250 | 0.9923 | -4.53% | 10-250 | 0.9907 | -11.07% | 10-250 | 0.9942 | -2.50% |
| 338 | Dinobuton | 10-250 | 0.9965 | -2.19% | 10-250 | 0.9931 | -4.26% | 10-250 | 0.9986 | 0.33% | 10-250 | 0.9987 | -4.54% |
| 339 | Tribufos | 10-250 | 0.9956 | -4.68% | 10-250 | 0.9959 | 11.82% | 10-250 | 0.9994 | 14.05% | 10-250 | 0.9902 | 10.63% |
| 340 | Flurochloridone | 10-250 | 0.9948 | -14.76% | 10-250 | 0.9932 | -8.09% | 20-250 | 0.9977 | 10.59% | 20-250 | 0.9932 | 3.28% |
| 341 | Bromfenvinphos | 10-250 | 0.9923 | -10.23% | 10-250 | 0.9963 | 6.23% | 10-250 | 0.9973 | 17.10% | 10-250 | 0.9903 | 11.56% |
| 342 | Perthan | 10-250 | 0.9955 | -3.84% | 10-250 | 0.9934 | -0.73% | 10-250 | 0.9988 | 12.80% | 10-250 | 1.0000 | -4.62% |
| 343 | Ditalimfos | 10-250 | 0.9935 | -15.33% | 10-100 | 0.9974 | 38.43% | 10-250 | 0.9550 | 4.47% | 10-250 | 0.9956 | 17.53% |
| 344 | 2,3,4,4',5-Pentachlorobiphenyl | 10-250 | 0.9915 | -17.88% | 10-250 | 0.9946 | -11.48% | 10-250 | 0.9906 | -12.32% | 10-250 | 0.9914 | -22.07% |
| 345 | 4,4'-Dibromobenzophenone | 10-250 | 0.9958 | -16.70% | 10-250 | 0.9975 | -2.49% | 10-250 | 0.9979 | -4.67% | 10-250 | 0.9960 | -1.16% |
| 346 | Flutriafol | 10-250 | 0.9937 | -15.77% | 10-250 | 0.9921 | -3.70% | 10-250 | 0.9996 | 5.75% | 10-250 | 0.9991 | 3.52% |
| 347 | Mephosfolan | 10-250 | 0.9966 | -14.35% | 10-250 | 0.9967 | -4.29% | 10-250 | 0.9998 | 13.31% | 10-250 | 0.9992 | 7.07% |
| 348 | Athidathion | 10-250 | 0.9925 | -4.18% | 10-250 | 0.9921 | -0.66% | 10-250 | 0.9911 | -1.70% | 10-250 | 0.9994 | -2.52% |
| 349 | 2,2',4,4',5,5'-Hexachlorobiphenyl | 20-250 | 0.9953 | -10.59% | 20-250 | 0.9922 | -21.34% | 50-250 | 0.9976 | 6.04% | 50-250 | 0.9989 | 9.36% |
| 350 | Diclobutrazol | 10-250 | 0.9954 | -5.03% | 10-250 | 0.9912 | -1.34% | 10-250 | 0.9996 | 2.12% | 10-250 | 0.9980 | -1.21% |
| 351 | Disulfoton-sulfone | 10-250 | 0.9955 | -11.63% | 10-250 | 0.9934 | -4.13% | 10-250 | 0.9997 | -3.47% | 10-250 | 0.9911 | 1.86% |
| 352 | Hexythiazox | 10-250 | 0.9991 | 19.93% | 10-250 | 0.9944 | 7.31% | 10-250 | 0.9991 | -14.66% | 10-250 | 0.9966 | -7.39% |
| 353 | 2,2',3,4,4',5'-Hexachlorobiphenyl | 50-250 | 0.9973 | -33.23% | 50-250 | 0.9991 | 31.22% | 100-250 | 0.9996 | 43.03% | 100-250 | 0.9945 | -15.24% |
| 354 | Triamiphos | 10-250 | 0.9915 | -3.68% | 10-250 | 0.9941 | -9.54% | 10-250 | 0.9998 | 8.99% | 10-250 | 0.9997 | -10.68% |
| 355 | Resmethrin | 10-250 | 0.9992 | 1.08% | 10-250 | 0.9961 | -10.72% | 10-250 | 0.9914 | -15.29% | 10-250 | 0.9971 | -11.77% |
| 356 | Cyproconazole | 10-250 | 0.9992 | -14.79% | 10-250 | 0.9974 | -5.69% | 10-250 | 0.9998 | 10.50% | 10-250 | 0.9999 | 9.01% |
| 357 | Benzyl butyl phthalate | 10-250 | 0.9971 | -4.43% | 10-250 | 0.9905 | 13.61% | 10-250 | 0.9902 | -15.36% | 10-250 | 1.0000 | -22.50% |
| 358 | Clodinafop-propargyl | 10-250 | 0.9976 | -18.00% | 10-250 | 0.9918 | -7.06% | 10-250 | 0.9988 | 1.76% | 10-250 | 0.9953 | 4.79% |
| 359 | Fenthion sulfoxide | 10-250 | 0.9667 | -4.17% | 10-250 | 0.9998 | 8.50% | 10-250 | 0.9998 | -16.77% | 10-250 | 0.9997 | 1.52% |
| 360 | Fluotrimazole | 10-250 | 0.9971 | -28.65% | 10-250 | 0.9936 | -13.30% | 10-250 | 1.0000 | 32.92% | 10-250 | 0.9968 | 13.68% |
| 361 | Fluroxypyr-1-methylheptyl ester | 10-250 | 0.9942 | -19.01% | 10-250 | 0.9975 | -19.78% | 10-250 | 0.9990 | 16.38% | 10-250 | 0.9985 | -18.45% |
| 362 | Fenthion-sulfone | 10-250 | 0.9963 | -10.93% | 10-250 | 0.9952 | 3.04% | 10-250 | 0.9987 | 8.52% | 10-250 | 0.9919 | -14.33% |
| 363 | Triphenyl phosphate | 10-250 | 0.9996 | -13.27% | 10-250 | 0.9949 | -7.26% | 10-250 | 0.9986 | -6.17% | 10-250 | 0.9961 | -8.78% |
| 364 | Metamitron | 10-250 | 0.9968 | -26.13% | 10-250 | 0.9933 | -10.84% | 10-250 | 0.9921 | 0.10% | 10-250 | 0.9967 | -14.88% |
| 365 | 2,2',3,4,4',5,5'-Heptachlorobiphenyl | 10-250 | 0.9912 | -6.19% | 10-250 | 0.9907 | -11.89% | 20-250 | 0.9925 | 7.29% | 20-250 | 0.9909 | -9.65% |
| 366 | Tebufenpyrad | 10-250 | 0.9904 | -4.12% | 10-250 | 0.9955 | -2.18% | 10-250 | 0.9969 | -4.75% | 10-250 | 0.9997 | -4.99% |
| 367 | Cloquintocet-mexyl | 10-250 | 0.9935 | -5.69% | 10-250 | 0.9972 | 5.50% | 10-250 | 0.9986 | -6.18% | 10-250 | 0.9991 | -5.86% |
| 368 | Lenacil | 10-250 | 0.9986 | -7.56% | 10-250 | 0.9991 | 3.11% | 10-250 | 0.9930 | -5.91% | 10-250 | 0.9959 | -13.64% |
| 369 | Bromuconazole | 10-250 | 0.9869 | -13.51% | 10-250 | 0.9492 | -9.34% | 10-250 | 0.9907 | -8.43% | 10-250 | 0.9997 | -0.56% |
| 370 | Nitralin | 10-250 | 0.9912 | -4.05% | 10-250 | 0.9985 | 3.08% | 10-250 | 0.9928 | -4.49% | 10-250 | 0.9991 | -3.52% |
| 371 | Fenamiphos sulfoxide | 10-250 | 0.9909 | -6.36% | 10-250 | 0.9976 | -3.91% | 10-250 | 0.9936 | -8.10% | 10-250 | 0.9990 | -7.19% |
| 372 | Fenamiphos sulfone | 10-250 | 0.9948 | -11.15% | 10-250 | 0.9946 | -18.36% | 10-250 | 0.9954 | 2.96% | 10-250 | 0.9957 | 6.35% |
| 373 | Fenpiclonil | 10-250 | 0.9937 | -3.64% | 10-250 | 0.9957 | -1.50% | 10-250 | 0.9954 | 6.93% | 10-250 | 0.9983 | 1.89% |
| 374 | Fluquinconazole | 10-250 | 0.9994 | -12.39% | 10-250 | 0.9903 | -1.58% | 10-250 | 0.9987 | -5.96% | 10-250 | 0.9955 | -7.97% |
| 375 | Fenbuconazole | 10-250 | 0.9974 | -4.30% | 10-250 | 0.9926 | -2.09% | 10-250 | 0.9903 | 27.77% | 10-250 | 0.9951 | -2.52% |
| 376 | Ethylene brassylate | 10-250 | 0.9943 | -8.39% | 10-250 | 0.9913 | 3.71% | 10-250 | 0.9999 | -0.68% | 10-250 | 0.9985 | 10.88% |
| 377 | Propoxur | 10-250 | 0.9976 | -7.44% | 10-250 | 0.9922 | -4.04% | 10-250 | 0.9930 | 0.36% | 10-250 | 0.9934 | 5.49% |
| 378 | Isoprocarb | 10-250 | 0.9952 | -7.49% | 10-250 | 0.9975 | -11.69% | 10-250 | 0.9974 | 9.07% | 10-250 | 0.9960 | 5.95% |
| 379 | Methamidophos | 10-250 | 0.9979 | -9.05% | 10-250 | 0.9956 | -11.30% | 10-250 | 0.9987 | 8.18% | 10-250 | 0.9983 | 1.78% |
| 380 | Acenaphthene | 10-250 | 0.9934 | -22.93% | 10-250 | 0.9977 | -0.25% | 10-250 | 0.9979 | -16.28% | 10-250 | 0.9950 | -4.90% |
| 381 | Dibutyl succinate | 10-250 | 0.9924 | -6.29% | 10-250 | 0.9967 | -4.55% | 10-250 | 0.9952 | -1.17% | 10-250 | 0.9922 | -1.98% |
| 382 | Phthalimide | 10-250 | 0.9785 | -6.17% | 10-250 | 0.9950 | -8.92% | 10-250 | 0.9991 | -5.45% | 10-250 | 0.9984 | -6.72% |
| 383 | Chlorethoxyfos | 10-250 | 0.9904 | -7.13% | 10-250 | 0.9927 | -1.50% | 10-250 | 0.9938 | -1.99% | 10-250 | 0.9992 | 0.26% |
| 384 | Pencycuron | 10-250 | 0.9957 | -8.41% | 10-250 | 0.9963 | -7.26% | 10-250 | 0.9998 | 10.27% | 10-250 | 0.9947 | 7.69% |
| 385 | Tebuthiuron | 10-250 | 0.9927 | -23.09% | 10-250 | 0.9926 | -6.97% | 10-250 | 0.9998 | 15.09% | 10-250 | 0.9971 | -2.57% |
| 386 | Demeton-S-methyl | 10-250 | 0.9987 | -16.76% | 10-250 | 0.9938 | -0.08% | 10-250 | 0.9998 | 12.26% | 10-250 | 0.9996 | 13.45% |
| 387 | Cadusafos | 10-250 | 0.9978 | -6.45% | 10-250 | 0.9946 | -15.40% | 10-250 | 0.9983 | 11.10% | 10-250 | 0.9993 | 1.03% |
| 388 | Phenanthrene | 10-250 | 0.9952 | -15.55% | 10-250 | 0.9989 | -7.25% | 10-250 | 0.9968 | 13.00% | 10-250 | 0.9963 | 15.41% |
| 389 | Spiroxamine | 10-250 | 0.9947 | -0.12% | 10-250 | 0.9986 | -11.69% | 10-250 | 0.9978 | 4.84% | 10-250 | 0.9967 | -3.41% |
| 390 | Fenpyroximate | 10-250 | 0.9943 | -5.11% | 10-250 | 0.9913 | -29.03% | 10-250 | 0.9942 | 7.23% | 10-250 | 0.9994 | 6.09% |
| 391 | Tebupirimfos | 10-250 | 0.9924 | -11.03% | 10-250 | 0.9965 | -6.72% | 10-250 | 0.9985 | 6.66% | 10-250 | 0.9983 | -3.96% |
| 392 | Prohydrojasmon | 10-250 | 0.9933 | -2.15% | 10-250 | 0.9953 | 7.75% | 10-250 | 0.9986 | 6.21% | 10-250 | 0.9945 | -4.30% |
| 393 | Fenpropidin | 10-250 | 0.9933 | -13.31% | 10-250 | 0.9927 | -11.20% | 10-250 | 0.9995 | 3.73% | 10-250 | 0.9997 | 4.67% |
| 394 | Dicloran | 10-250 | 0.9962 | 6.31% | 10-250 | 0.9947 | -7.82% | 10-250 | 0.9976 | 3.51% | 10-250 | 0.9980 | -0.93% |
| 395 | Pyroquilon | 10-250 | 0.9982 | -4.34% | 10-250 | 0.9987 | -1.25% | 10-250 | 0.9977 | 2.45% | 10-250 | 0.9992 | -0.42% |
| 396 | Propyzamide | 10-250 | 0.9973 | 13.96% | 10-250 | 0.9965 | 5.33% | 10-250 | 0.9969 | -3.19% | 10-250 | 0.9995 | -4.73% |
| 397 | Pirimicarb | 10-250 | 0.9915 | -24.32% | 10-250 | 0.9944 | -13.79% | 10-250 | 0.9986 | -9.39% | 10-250 | 0.9998 | 9.37% |
| 398 | Phosphamidon | 10-250 | 0.9973 | -6.99% | 10-250 | 0.9952 | -1.19% | 10-250 | 0.9991 | 1.86% | 10-250 | 0.9923 | 4.54% |
| 399 | Benoxacor | 10-250 | 0.9964 | -8.12% | 10-250 | 0.9931 | -8.36% | 10-250 | 0.9910 | -9.53% | 10-250 | 0.9956 | -2.17% |
| 400 | Bromobutide | 10-250 | 0.9931 | -17.98% | 10-250 | 0.9989 | -2.76% | 10-250 | 0.9904 | -14.14% | 10-250 | 0.9964 | -6.72% |
| 401 | Acetochlor | 10-250 | 0.9986 | -12.49% | 10-250 | 0.9975 | -5.88% | 10-250 | 0.9941 | -0.90% | 10-250 | 0.9996 | 12.67% |
| 402 | Tridiphane | 10-250 | 0.9922 | -6.74% | 10-250 | 0.9988 | -8.36% | 10-250 | 0.9969 | -1.45% | 10-250 | 1.0000 | -5.32% |
| 403 | Terbucarb | 10-250 | 0.9981 | -2.36% | 10-250 | 0.9912 | -3.05% | 10-250 | 0.9955 | 7.43% | 10-250 | 0.9985 | 2.12% |
| 404 | Esprocarb | 10-250 | 0.9983 | -5.04% | 10-250 | 0.9944 | -1.69% | 10-250 | 0.9966 | -1.91% | 10-250 | 0.9951 | -7.09% |
| 405 | Fenfuram | 10-250 | 0.9928 | -8.40% | 10-250 | 0.9916 | -4.87% | 10-250 | 0.9913 | 5.98% | 10-250 | 0.9992 | 17.62% |
| 406 | Acibenzolar-S-methyl | 10-250 | 0.9928 | -10.13% | 10-250 | 0.9954 | -7.50% | 50-250 | 0.9994 | -8.95% | 50-250 | 1.0000 | -7.78% |
| 407 | Benfuresate | 10-250 | 0.9984 | -3.31% | 10-250 | 0.9956 | -1.14% | 50-250 | 0.9942 | -3.06% | 50-250 | 0.9961 | -1.35% |
| 408 | Dithiopyr | 10-250 | 0.9952 | -15.95% | 10-250 | 0.9927 | 1.83% | 10-250 | 0.9950 | -4.21% | 10-250 | 0.9912 | -8.56% |
| 409 | Metalaxyl-m | 10-250 | 0.9956 | -10.89% | 10-250 | 0.9982 | -4.77% | 10-250 | 0.9930 | -23.80% | 10-250 | 0.9997 | -6.16% |
| 410 | Malaoxon | 10-250 | 0.9981 | -0.59% | 10-250 | 0.9944 | -2.86% | 10-250 | 0.9947 | -5.55% | 10-250 | 0.9977 | -3.23% |
| 411 | Simeconazole | 10-250 | 0.9983 | -17.89% | 10-250 | 0.9972 | -5.86% | 10-250 | 0.9965 | -5.97% | 10-250 | 0.9958 | -16.18% |
| 412 | Chlorthal-dimethyl | 10-250 | 0.9967 | -3.31% | 10-250 | 0.9965 | -1.20% | 10-250 | 0.9949 | 11.12% | 10-250 | 0.9993 | -13.62% |
| 413 | Thiazopyr | 10-250 | 0.9968 | -12.99% | 10-250 | 0.9958 | -8.14% | 10-250 | 0.9966 | -5.13% | 10-250 | 0.9958 | 6.66% |
| 414 | Dimethylvinphos | 10-250 | 0.9921 | -5.76% | 10-250 | 0.9972 | -2.77% | 10-250 | 0.9973 | 13.83% | 10-250 | 0.9955 | 14.87% |
| 415 | Butralin | 10-250 | 0.9928 | -2.55% | 10-250 | 0.9935 | -5.59% | 10-250 | 0.9920 | 16.70% | 10-250 | 0.9994 | 16.57% |
| 416 | Zoxamide | 10-250 | 0.9953 | -9.88% | 10-250 | 0.9904 | -7.95% | 10-250 | 0.9946 | -3.42% | 10-250 | 0.9983 | -5.97% |
| 417 | Pyrifenox | 10-250 | 0.9982 | -3.37% | 10-250 | 0.9967 | -8.27% | 10-250 | 0.9982 | 12.10% | 10-250 | 0.9963 | 4.67% |
| 418 | Allethrin | 10-250 | 0.9914 | -9.48% | 10-250 | 0.9925 | -4.53% | 10-250 | 0.9977 | 15.76% | 10-250 | 0.9948 | 8.02% |
| 419 | Dimethametryn | 10-250 | 0.9926 | -23.57% | 10-250 | 0.9916 | 6.80% | 10-250 | 0.9947 | 8.05% | 10-250 | 0.9956 | 13.63% |
| 420 | Quinoclamine | 10-250 | 0.9913 | -3.54% | 10-250 | 0.9944 | -4.97% | 10-250 | 0.9934 | 2.93% | 10-250 | 1.0000 | 1.41% |
| 421 | Methothrin | 20-250 | 0.9944 | -18.00% | 10-250 | 0.9962 | -7.40% | 10-250 | 0.9948 | -14.72% | 50-250 | 0.9957 | -27.74% |
| 422 | Flufenacet | 10-250 | 0.9953 | -8.38% | 10-250 | 0.9963 | -4.70% | 10-250 | 0.9937 | -3.36% | 10-250 | 0.9910 | 4.78% |
| 423 | Fenoxanil | 10-250 | 0.9923 | -19.90% | 10-250 | 0.9921 | -12.72% | 10-250 | 0.9986 | -2.63% | 10-250 | 0.9950 | 9.70% |
| 424 | Fthalide | 10-250 | 0.9933 | -5.97% | 10-250 | 0.9811 | -8.24% | 10-250 | 0.9960 | 2.53% | 10-250 | 0.9973 | -4.35% |
| 425 | Furalaxyl | 10-250 | 0.9932 | -8.64% | 10-250 | 0.9905 | -1.18% | 10-250 | 0.9962 | 15.97% | 10-250 | 0.9971 | 13.14% |
| 426 | Thiamethoxam | 10-250 | 0.9931 | -7.63% | 10-250 | 0.9946 | -4.13% | 10-250 | 0.9992 | 21.14% | 10-250 | 0.9950 | -8.62% |
| 427 | Mepanipyrim | 10-250 | 0.9939 | -7.43% | 10-250 | 0.9932 | -6.44% | 10-250 | 0.9996 | 7.52% | 10-250 | 0.9968 | 1.01% |
| 428 | Captan | 10-250 | 0.9925 | -14.78% | 10-250 | 0.9928 | -8.42% | 10-250 | 0.9941 | 12.78% | 10-250 | 0.9975 | -11.85% |
| 429 | Bromacil | 10-250 | 0.9943 | -6.39% | 10-250 | 0.9966 | -0.87% | 10-250 | 0.9972 | 1.99% | 10-250 | 0.9979 | 1.74% |
| 430 | Picoxystrobin | 10-250 | 0.9912 | -10.85% | 10-250 | 0.9946 | -1.80% | 10-250 | 0.9989 | -13.32% | 10-250 | 0.9968 | -8.87% |
| 431 | Butamifos | 10-250 | 0.9969 | -8.64% | 10-250 | 0.9905 | -15.49% | 10-250 | 0.9995 | -17.88% | 10-250 | 0.9987 | -22.65% |
| 432 | Imazamethabenz-methyl | 10-250 | 0.9958 | -5.33% | 10-250 | 0.9973 | -1.02% | 10-250 | 0.9981 | 2.62% | 10-250 | 0.9988 | 4.38% |
| 433 | (E)-Metominostrobin | 10-250 | 0.9916 | -13.13% | 10-250 | 0.9942 | -2.84% | 10-250 | 0.9980 | 4.38% | 10-250 | 0.9983 | 0.57% |
| 434 | TCMTB | 10-250 | 0.9919 | -16.97% | 10-250 | 0.9902 | -11.24% | 10-250 | 0.9986 | 6.72% | 10-250 | 0.9998 | 2.81% |
| 435 | Methiocarb sulfone | 10-250 | 0.9942 | -6.10% | 10-250 | 0.9970 | -12.28% | 10-250 | 0.9950 | -17.88% | 10-250 | 0.9970 | -25.15% |
| 436 | Imazalil | 10-250 | 0.9938 | -23.69% | 10-250 | 0.9919 | -17.31% | 10-250 | 0.9982 | 13.48% | 10-250 | 0.9973 | 7.41% |
| 437 | Isoprothiolane | 10-250 | 0.9988 | -4.83% | 10-250 | 0.9979 | -5.45% | 10-250 | 0.9948 | 6.81% | 10-250 | 0.9987 | 4.54% |
| 438 | Cyflufenamid | 10-250 | 0.9945 | -6.04% | 10-250 | 0.9911 | -3.47% | 10-250 | 0.9968 | -2.28% | 10-250 | 0.9987 | -6.64% |
| 439 | Pyriminobac-methyl | 10-250 | 0.9982 | -8.55% | 10-250 | 0.9946 | -1.87% | 10-250 | 0.9971 | 20.59% | 10-150 | 0.9984 | 43.78% |
| 440 | Isoxathion | 10-250 | 0.9979 | -8.17% | 10-250 | 0.9972 | -1.32% | 10-250 | 0.9958 | -5.53% | 10-250 | 0.9987 | -3.60% |
| 441 | (Z)-Metominostrobin | 10-250 | 0.9932 | -12.53% | 10-250 | 0.9986 | -7.11% | 10-250 | 0.9958 | -6.09% | 10-250 | 0.9956 | -11.48% |
| 442 | Diofenolan | 10-250 | 0.9988 | -1.74% | 10-250 | 0.9953 | -0.88% | 10-250 | 0.9966 | -0.88% | 10-250 | 0.9984 | -2.79% |
| 443 | Thifluzamide | 10-250 | 0.9978 | -4.78% | 10-250 | 0.9967 | -2.06% | 10-250 | 0.9995 | 0.45% | 10-250 | 0.9904 | -9.57% |
| 444 | Quinoxyfen | 10-250 | 0.9901 | -13.18% | 10-250 | 0.9922 | -4.61% | 10-250 | 0.9983 | -10.24% | 10-250 | 0.9915 | -3.76% |
| 445 | Chlorfenapyr | 10-250 | 0.9995 | -5.20% | 10-250 | 0.9928 | -1.67% | 10-250 | 0.9913 | -5.98% | 10-250 | 0.9953 | 3.86% |
| 446 | Trifloxystrobin | N.D | N.D | N.D | N.D | N.D | N.D | 10-250 | 0.9918 | 5.58% | 10-250 | 0.9977 | 1.68% |
| 447 | Imibenconazole-oxon-desbenzyl | 10-250 | 0.9915 | -22.48% | 10-250 | 0.9966 | -14.40% | 10-250 | 0.9962 | -22.56% | 10-250 | 0.9902 | -12.61% |
| 448 | Isoxadifen-ethyl | 10-250 | 0.9977 | -9.05% | 10-250 | 0.9937 | -7.43% | 10-250 | 0.9999 | -8.46% | 10-250 | 0.9973 | -7.37% |
| 449 | Fipronil | 10-250 | 0.9974 | -7.46% | 10-250 | 0.9939 | -2.39% | 10-250 | 0.9993 | -8.27% | 10-250 | 0.9971 | -2.28% |
| 450 | Imiprothrin | 10-250 | 0.9936 | -12.50% | 10-250 | 0.9965 | -11.99% | 10-250 | 0.9933 | 5.29% | 10-250 | 0.9911 | -5.76% |
| 451 | Carfentrazone-ethyl | 10-250 | 0.9968 | 0.30% | 10-250 | 0.9969 | -0.97% | 10-250 | 0.9925 | 4.24% | 10-250 | 0.9964 | 21.86% |
| 452 | Epoxiconazole | 10-250 | 0.9981 | -9.59% | 10-250 | 0.9996 | -2.79% | 10-250 | 0.9976 | -3.41% | 10-250 | 0.9933 | -7.53% |
| 453 | Pyraflufen-ethyl | 10-250 | 0.9945 | -16.06% | 10-250 | 0.9987 | -13.56% | 10-250 | 0.9923 | -6.87% | 10-250 | 0.9985 | -0.13% |
| 454 | Pyributicarb | 10-250 | 0.9927 | -5.61% | 10-250 | 0.9909 | -2.36% | 10-250 | 0.9951 | -18.25% | 10-250 | 0.9992 | -18.69% |
| 455 | Thenylchlor | 10-250 | 0.9911 | -14.77% | 10-250 | 0.9988 | -24.96% | 10-250 | 0.9910 | -11.38% | 10-250 | 0.9912 | -9.27% |
| 456 | Clethodim | 10-250 | 0.9978 | -1.19% | 10-250 | 0.9998 | -0.51% | 10-250 | 0.9965 | -6.99% | 10-250 | 0.9961 | 2.59% |
| 457 | Mefenpyr-diethyl | 10-250 | 0.9947 | -15.59% | 10-250 | 0.9953 | -6.37% | 10-250 | 0.9985 | 7.53% | 10-250 | 0.9966 | -6.59% |
| 458 | Famphur | 10-250 | 0.9964 | -17.61% | 10-250 | 0.9925 | -6.69% | 10-250 | 0.9959 | 9.25% | 10-250 | 0.9975 | -11.17% |
| 459 | Etoxazole | 10-250 | 0.9933 | -2.34% | 10-250 | 0.9968 | -15.06% | 10-250 | 0.9958 | 9.11% | 10-250 | 0.9967 | 9.84% |
| 460 | Pyriproxyfen | 10-250 | 0.9977 | -17.17% | 10-250 | 0.9927 | -19.32% | 10-250 | 0.9999 | -6.30% | 10-250 | 0.9909 | 10.18% |
| 461 | Picolinafen | 10-250 | 0.9941 | -5.43% | 10-250 | 0.9956 | -0.72% | 10-250 | 0.9970 | -5.97% | 10-250 | 0.9987 | -2.95% |
| 462 | Iprodione | 10-250 | 0.9945 | -9.19% | 10-250 | 0.9924 | -5.58% | 10-250 | 0.9932 | 5.39% | 10-250 | 0.9956 | 10.85% |
| 463 | Piperophos | 10-250 | 0.9981 | -12.68% | 10-250 | 0.9946 | -8.66% | 10-250 | 0.9989 | -19.53% | 10-250 | 1.0000 | 2.45% |
| 464 | Ofurace | 10-250 | 0.9985 | -13.41% | 10-250 | 0.9972 | -6.69% | 10-250 | 0.9917 | 4.77% | 10-250 | 0.9975 | -1.31% |
| 465 | Bifenazate | 10-250 | 0.9908 | -9.37% | 10-250 | 0.9984 | -7.06% | 10-250 | 0.9977 | -5.36% | 10-250 | 0.9951 | -15.06% |
| 466 | Endrin ketone | 10-250 | 0.9946 | -0.87% | 10-250 | 0.9932 | -7.94% | 10-250 | 0.9961 | -9.83% | 10-250 | 0.9934 | 7.76% |
| 467 | Clomeprop | 10-250 | 0.9917 | -9.00% | 10-250 | 0.9988 | -6.99% | 10-250 | 0.9998 | 6.19% | 10-250 | 0.9942 | 9.80% |
| 468 | Fenamidone | 10-250 | 0.9939 | -16.29% | 10-250 | 0.9978 | -6.11% | 10-250 | 0.9939 | -16.42% | 10-250 | 0.9931 | -14.07% |
| 469 | Naproanilide | 10-250 | 0.9981 | -11.34% | 10-250 | 0.9974 | -3.88% | 10-250 | 0.9912 | 3.48% | 10-250 | 0.9960 | 9.81% |
| 470 | Pyraclostrobin | 10-250 | 0.9924 | 2.48% | 10-250 | 0.9914 | 3.50% | 10-250 | 0.9988 | 5.77% | 10-250 | 0.9921 | -3.41% |
| 471 | Lactofen | 10-250 | 0.9913 | -8.65% | 10-250 | 0.9964 | -15.51% | 10-250 | 0.9996 | -15.97% | 10-250 | 0.9977 | -8.77% |
| 472 | Tralkoxydim | 10-250 | 0.9999 | -17.86% | 10-250 | 0.9987 | -11.99% | 10-250 | 0.9932 | -11.03% | 10-250 | 0.9921 | 2.91% |
| 473 | Pyraclofos | 10-250 | 0.9951 | -2.02% | 10-250 | 0.9997 | -0.28% | 10-250 | 0.9926 | 20.23% | 10-250 | 0.9986 | 7.58% |
| 474 | Dialifos | 10-250 | 0.9938 | -4.52% | 10-250 | 0.9954 | -1.32% | 10-250 | 0.9998 | -8.22% | 10-250 | 0.9923 | -3.41% |
| 475 | Spirodiclofen | 10-250 | 0.9982 | -8.34% | 10-250 | 0.9967 | -19.40% | 10-250 | 0.9939 | -10.86% | 10-250 | 0.9982 | -8.50% |
| 476 | Halfenprox | 10-250 | 0.9947 | -2.93% | 10-250 | 0.9929 | -7.59% | 10-250 | 1.0000 | 5.59% | 10-250 | 0.9921 | 7.07% |
| 477 | Flurtamone | 10-250 | 0.9963 | -4.27% | 10-250 | 0.9961 | -4.16% | 10-250 | 0.9930 | 4.53% | 10-250 | 0.9943 | 2.68% |
| 478 | Pyriftalid | 10-250 | 0.9941 | -6.93% | 10-250 | 0.9976 | -4.59% | 10-250 | 0.9977 | 8.06% | 10-250 | 0.9927 | 12.49% |
| 479 | Silafluofen | 10-250 | 0.9931 | -14.52% | 10-250 | 0.9958 | -2.20% | 10-250 | 0.9964 | -0.23% | 10-250 | 0.9960 | 0.59% |
| 480 | Pyrimidifen | 10-250 | 0.9944 | -8.21% | 10-250 | 0.9904 | -9.03% | 10-250 | 0.9957 | -2.17% | 10-250 | 0.9979 | -4.42% |
| 481 | Acetamiprid | 10-250 | 0.9939 | -6.61% | 10-250 | 0.9988 | -3.55% | 10-250 | 0.9990 | 6.41% | 10-250 | 0.9993 | 4.08% |
| 482 | Butafenacil | 10-250 | 0.9919 | -5.82% | 10-250 | 0.9949 | -4.19% | 10-250 | 0.9922 | 3.79% | 10-250 | 0.9952 | 2.44% |
| 483 | Cafenstrole | 10-250 | 0.9913 | -9.97% | N.D | N.D | N.D | 100-250 | 0.9959 | -21.08% | 100-250 | 0.9970 | -24.14% |
| 484 | Fluridone | 10-250 | 0.9984 | -9.44% | 10-250 | 0.9995 | -2.63% | 10-250 | 0.9993 | 10.99% | 10-250 | 0.9958 | 4.71% |
| 485 | Heptachlor-2,3-exo-epoxide | 10-250 | 0.9935 | -5.13% | 10-250 | 0.9967 | -2.02% | 10-250 | 0.9987 | -0.30% | 10-250 | 0.9969 | -1.87% |
| 486 | Methamidophos | 10-250 | 0.9979 | -17.79% | 10-250 | 0.9963 | 7.55% | 10-250 | 0.9986 | 10.66% | 10-250 | 0.9988 | -4.23% |
| 487 | Carbofuran | 10-250 | 0.9980 | 18.51% | 10-250 | 0.9966 | 15.40% | 10-250 | 0.9983 | 3.21% | 10-250 | 0.9955 | -0.87% |
| 488 | Acetamiprid | 10-250 | 0.9984 | -9.75% | 10-250 | 0.9973 | 8.63% | 10-250 | 0.9966 | -3.11% | 10-250 | 0.9976 | 10.86% |
| 489 | Trichlorfon | 10-250 | 0.9982 | -12.70% | 10-250 | 0.9921 | -4.17% | 10-250 | 0.9959 | -21.67% | 10-250 | 0.9942 | -25.49% |
| 490 | Demeton | N.D | N.D | N.D | N.D | N.D | N.D | N.D | N.D | N.D | N.D | N.D | N.D |
| 491 | Phorate sulfoxide | 20-250 | 0.9979 | -32.34% | 20-250 | 0.9985 | -24.65% | 10-250 | 0.9942 | 9.80% | 10-250 | 0.9994 | -6.37% |
| 492 | Oxycarboxin | 10-250 | 0.9968 | -26.93% | 10-250 | 0.9971 | 0.09% | 10-250 | 0.9965 | -5.86% | 10-250 | 0.9966 | 11.72% |
| 493 | Phoxim | 10-250 | 0.9933 | -4.05% | 10-250 | 0.9933 | 29.59% | 10-250 | 0.9967 | -1.58% | 10-250 | 0.9943 | -2.37% |
| 494 | Methoxyfenozide | 10-250 | 0.9946 | -0.53% | 10-250 | 0.9997 | -4.99% | 10-250 | 0.9974 | 14.90% | 10-250 | 0.9970 | -10.99% |
| 495 | Diafenthiuron | 10-250 | 0.9977 | -6.88% | 10-250 | 0.9967 | -20.01% | 10-250 | 0.9993 | -6.51% | 10-250 | 0.9986 | -5.88% |
| 496 | Thifensulfuron-methyl | 10-250 | 0.9922 | 15.68% | 10-250 | 0.9999 | 18.28% | 10-250 | 0.9982 | 8.44% | 10-250 | 0.9997 | 7.04% |
| 497 | Ethoxysulfuron | 10-250 | 0.9999 | -0.85% | 10-250 | 0.9945 | 0.63% | 10-250 | 0.9964 | 4.56% | 10-250 | 0.9932 | 9.53% |
| 498 | Spinosad | 10-250 | 0.9978 | 18.59% | 10-250 | 0.9975 | -10.96% | 10-250 | 0.9953 | 1.33% | 10-250 | 0.9970 | -0.92% |
| 499 | Mepiquat chloride | 10-250 | 0.9943 | 21.58% | 50-250 | 0.9962 | 50.27% | 10-250 | 0.9933 | 2.44% | 50-250 | 0.9988 | 26.52% |
| 500 | Tricyclazole | 10-250 | 0.9971 | -16.96% | 10-250 | 0.9997 | 1.92% | 10-250 | 0.9977 | 1.49% | 10-250 | 0.9924 | 4.89% |
| 501 | Isoproturon | 10-250 | 0.9935 | -12.30% | 10-250 | 0.9984 | -14.43% | 10-250 | 0.9990 | -12.65% | 10-250 | 0.9996 | -10.83% |
| 502 | Pymetrozine | 10-250 | 0.9947 | -0.18% | 10-250 | 0.9931 | -16.00% | 10-250 | 0.9950 | -12.47% | 10-250 | 0.9982 | -5.97% |
| 503 | Flumetsulam | 10-250 | 0.9956 | 8.10% | 10-250 | 0.9967 | -8.04% | 10-250 | 0.9980 | -5.02% | 10-250 | 0.9938 | 3.59% |
| 504 | Thiodicarb | 10-250 | 0.9988 | -6.81% | 10-250 | 0.9994 | 10.53% | 10-250 | 0.9955 | 10.00% | 10-250 | 0.9959 | 10.41% |
| 505 | Cinosulfuron | 10-250 | 0.9963 | -0.19% | 10-250 | 0.9930 | 9.35% | 10-250 | 0.9937 | 1.50% | 10-250 | 0.9926 | -9.78% |
| 506 | Pyrazosulfuron-ethyl | 10-250 | 0.9977 | 7.73% | 10-250 | 0.9950 | 10.10% | 10-250 | 0.9970 | -0.68% | 10-250 | 0.9969 | 12.33% |
| 507 | Methomyl | 10-250 | 0.9930 | -5.12% | 10-250 | 0.9980 | 11.77% | 10-250 | 0.9965 | 17.63% | 20-250 | 0.9972 | 27.16% |
| 508 | Cymoxanil | 10-250 | 0.9964 | 0.43% | 10-250 | 0.9973 | 13.60% | 10-250 | 0.9985 | 9.59% | 10-250 | 0.9959 | 37.80% |
| 509 | Omethoate | 10-250 | 0.9993 | -14.80% | 10-250 | 0.9936 | -8.03% | 10-250 | 0.9968 | 37.26% | 10-250 | 0.9972 | -22.35% |
| 510 | Ethoxyquin | 10-250 | 0.9987 | -15.50% | 10-250 | 0.9931 | -10.58% | 10-250 | 0.9963 | 0.65% | 10-250 | 0.9987 | 7.48% |
| 511 | Aldoxycarb | 10-250 | 0.9960 | -3.48% | 10-250 | 0.9950 | 20.70% | 10-250 | 0.9970 | 12.09% | 10-250 | 0.9986 | -5.31% |
| 512 | Imazapic | 10-250 | 0.9947 | -11.93% | 10-250 | 0.9983 | 15.23% | 10-250 | 0.9995 | -3.07% | 10-250 | 0.9985 | 1.85% |
| 513 | Uniconazole | 10-250 | 0.9962 | -4.16% | 10-250 | 0.9995 | -3.21% | 10-250 | 0.9994 | 13.16% | 10-250 | 0.9975 | 13.43% |
| 514 | Clofentezine | 10-250 | 0.9954 | -10.13% | 10-250 | 0.9967 | -6.11% | 10-250 | 0.9985 | -10.23% | 10-250 | 0.9987 | -8.01% |
| 515 | Vamidothion sulfone | 10-250 | 0.9945 | 0.21% | 10-250 | 0.9983 | 16.96% | 10-250 | 0.9915 | 3.87% | 10-250 | 0.9993 | 39.38% |
| 516 | Terbufos sulfone | 10-250 | 0.9946 | 10.30% | 10-250 | 0.9997 | 2.37% | 10-250 | 0.9995 | -2.53% | 10-250 | 0.9947 | -7.54% |
| 517 | Cyazofamid | 10-250 | 0.9961 | -24.78% | 10-250 | 0.9882 | -0.97% | 10-250 | 0.9997 | 11.61% | 10-250 | 0.9988 | 14.29% |
| 518 | Florasulam | 10-250 | 0.9971 | -3.74% | 10-250 | 0.9946 | 2.27% | 10-250 | 0.9940 | -11.28% | 10-250 | 0.9972 | 0.14% |
| 519 | Benzoximate | 10-250 | 0.9952 | -1.98% | 10-250 | 0.9976 | -4.78% | 10-250 | 0.9930 | -4.89% | 10-250 | 0.9989 | 3.12% |
| 520 | Chlormequat chloride | 10-250 | 0.9972 | 4.56% | 10-250 | 0.9992 | 12.02% | 10-250 | 0.9971 | -11.99% | 10-250 | 0.9934 | 12.42% |
| 521 | Sethoxydim | 10-250 | 0.9945 | -8.55% | 10-250 | 0.9953 | -3.09% | 10-250 | 0.9930 | -2.19% | 10-250 | 0.9960 | 7.32% |
| 522 | Folpet | 10-250 | 0.9987 | -4.23% | 10-250 | 0.9999 | -4.80% | 10-250 | 0.9996 | -13.47% | 10-250 | 0.9987 | 10.59% |
| 523 | Methiocarb | 10-250 | 0.9984 | -7.76% | 10-250 | 0.9969 | -17.06% | 10-250 | 0.9977 | 5.77% | 10-250 | 0.9994 | -0.20% |
| 524 | Cartap hydrochloride | 10-250 | 0.9974 | -6.70% | 10-250 | 0.9960 | -6.32% | 10-250 | 0.9948 | -4.79% | 10-250 | 0.9993 | -11.86% |
| 525 | Propyl phosphate | 10-250 | 0.9974 | -26.04% | 10-250 | 0.9973 | -3.60% | 10-250 | 0.9928 | 3.14% | 10-250 | 0.9996 | 2.99% |
| 526 | Triisobutyl phosphate | 10-250 | 0.9987 | 3.94% | 10-250 | 0.9944 | 12.30% | 10-250 | 0.992 | -2.34% | 10-250 | 0.995 | -6.85% |
| 527 | Tris(2-butoxyethyl) phosphate | 10-250 | 0.9992 | 4.29% | 10-250 | 0.9993 | 17.34% | 10-250 | 0.9951 | 3.27% | 10-250 | 0.9937 | 1.90% |
| 528 | 2-Ethylhexyldiphenyl phosphate | 10-250 | 0.9944 | 25.07% | 10-250 | 0.9959 | 35.16% | 10-250 | 0.9937 | 6.93% | 10-250 | 0.9943 | -4.06% |
| 529 | Tris(2-ethylhexyl) Phosphate | 10-250 | 0.9986 | 31.75% | 10-250 | 0.9992 | 32.43% | 10-100 | 0.9985 | 4.64% | 10-100 | 0.9982 | 7.74% |
| 530 | Tris(3-methylphenyl) phosphate | 10-250 | 0.9987 | -68.23% | 10-250 | 0.9952 | -37.09% | 10-250 | 0.9997 | -5.94% | 10-250 | 0.9925 | -6.00% |
| 531 | Tri-p-tolyl phosphate | 10-250 | 0.9992 | -11.94% | 10-250 | 0.9974 | 16.96% | 10-250 | 0.9971 | 1.79% | 10-250 | 0.9947 | 1.82% |
| 532 | Tris(2-methylphenyl) phosphate | 10-250 | 0.9953 | -21.42% | 10-250 | 0.9984 | 10.79% | 10-250 | 0.9983 | 1.18% | 10-250 | 0.9962 | 3.58% |
| 533 | Tris(1,3-dichloroisopropyl) phosphate | 10-250 | 0.9954 | -3.33% | 10-250 | 0.9914 | -17.89% | 20-250 | 0.998 | -18.82% | 10-250 | 0.9967 | -0.70% |
| 534 | Cresyl diphenyl phosphate | 10-250 | 0.9985 | 27.75% | 10-250 | 0.9988 | 35.27% | 10-250 | 0.9985 | 11.66% | 10-250 | 0.9986 | -6.64% |
| 535 | Phenyl phosphate | 10-250 | 0.9936 | -9.28% | 10-250 | 0.9957 | -28.59% | 10-250 | 0.9973 | 17.86% | 10-250 | 0.9987 | 16.62% |
| 536 | Tris(1-Chloro-2-Propyl) Phosphate | 10-250 | 0.9989 | 6.35% | 10-250 | 0.9960 | -11.32% | 10-250 | 0.9987 | 28.68% | 10-250 | 0.9987 | 13.63% |
| 537 | Tributyl phosphate | 10-250 | 0.9997 | -18.42% | 10-250 | 0.9966 | -16.78% | 10-250 | 0.992 | 10.13% | 10-250 | 0.9941 | -12.88% |
| 538 | tris(2-chloroethyl) phosphate | 10-250 | 0.9961 | 0.70% | 10-250 | 0.9989 | -25.67% | 10-250 | 0.9984 | 4.50% | 10-250 | 0.9988 | 18.15% |
| 539 | Triethyl phosphate | 10-250 | 0.9923 | -36.35% | 10-250 | 0.9955 | -18.18% | 10-250 | 0.9968 | 3.94% | 10-250 | 0.9977 | -2.03% |
| 540 | Trimethyl phosphate | 10-250 | 0.9988 | 4.95% | 10-250 | 0.9931 | -1.93% | 10-250 | 0.994 | 9.56% | 10-250 | 0.9996 | 4.55% |
| 541 | Benzyl butyl phthalate | 10-250 | 0.9974 | 2.26% | 10-250 | 0.9989 | 21.33% | 10-250 | 0.9996 | -1.46% | 10-250 | 0.9962 | -5.71% |
| 542 | [Diphenyl phthalate](https://www.chemsrc.com/en/cas/84-62-8_510543.html) | 10-250 | 0.9991 | -29.01% | 10-250 | 0.9925 | -6.17% | 10-250 | 0.9982 | 5.94% | 10-250 | 0.9981 | -7.70% |
| 543 | [β-Butoxyethyl phthalate](https://www.chemsrc.com/en/cas/117-83-9_750429.html) | 10-250 | 0.9988 | 12.88% | 10-250 | 0.9970 | -2.22% | 10-250 | 0.9983 | 4.03% | 10-250 | 0.9939 | 4.97% |
| 544 | [Dibutyl phthalate](https://www.chemsrc.com/en/cas/84-74-2_336203.html) | 10-250 | 0.9942 | -6.80% | 10-250 | 0.9981 | 11.37% | 10-250 | 0.9953 | -5.58% | 10-250 | 0.9979 | -4.63% |
| 545 | [Dicyclohexyl phthalate](https://www.chemsrc.com/en/cas/84-61-7_670264.html) | 10-250 | 0.9977 | 26.44% | 10-250 | 0.9910 | 0.83% | 10-250 | 0.9982 | 6.70% | 10-250 | 0.9961 | -2.42% |
| 546 | [Dimethyl phthalate](https://www.chemsrc.com/en/cas/131-11-3_28935.html) | 10-250 | 0.9991 | 7.60% | 10-250 | 0.9991 | 23.69% | 10-250 | 0.9998 | -24.26% | 10-250 | 0.9981 | -6.50% |
| 547 | dipentyl phthalate | 10-250 | 0.9961 | -4.90% | 10-250 | 0.9988 | -19.81% | 10-250 | 0.9922 | -6.76% | 10-250 | 0.9935 | -8.70% |
| 548 | [Diethyl phthalate](https://www.chemsrc.com/en/cas/84-66-2_401979.html) | 10-250 | 1.0000 | -9.59% | 10-250 | 0.9921 | 0.01% | 20-250 | 0.996 | 25.27% | 20-250 | 0.996 | 11.47% |
| 549 | Diisobutyl phthalate | 10-250 | 0.9961 | -11.69% | 10-250 | 0.9977 | 17.07% | 20-250 | 0.9921 | 13.45% | 20-250 | 0.9952 | 17.76% |
| 550 | Dihexyl phthalate | 10-250 | 0.9975 | 3.44% | 10-250 | 0.9949 | -13.97% | 10-250 | 0.9999 | 17.26% | 10-250 | 0.9922 | 6.49% |
| 551 | Dioctyl phthalate | 10-250 | 0.9958 | -6.50% | 10-250 | 0.9926 | 5.01% | 10-250 | 0.998 | 9.21% | 10-250 | 0.9939 | 2.71% |
| 552 | Bis(2-methoxyethyl) phthalate | 10-250 | 0.9961 | 5.74% | 10-250 | 0.9991 | -8.07% | 10-250 | 0.9977 | 9.34% | 10-250 | 0.9976 | -5.79% |
| 553 | bis(2-ethoxyethyl) benzene-1,2-dicarboxylate | 10-250 | 0.9999 | -19.50% | 10-250 | 0.9927 | 0.84% | 10-250 | 0.9953 | 8.12% | 10-250 | 0.9963 | -13.36% |
| 554 | 1,2-Benzenedicarboxylic acid, 1,2-bis(1,3-dimethylbutyl) ester | 10-250 | 0.9913 | 8.66% | 10-250 | 0.9921 | 22.35% | 10-250 | 0.9999 | 22.86% | 10-250 | 0.9962 | 17.70% |
| 555 | bis(2-ethylhexyl) phthalate | 10-250 | 0.9990 | 7.36% | 10-250 | 0.9939 | -18.73% | 10-250 | 0.996 | 2.24% | 10-250 | 0.9918 | 6.46% |
| 556 | Dinonyl phthalate | 10-250 | 0.9988 | -11.48% | 10-250 | 0.9992 | -8.83% | 10-250 | 0.9932 | 6.29% | 10-250 | 0.9992 | 15.88% |
| 557 | N-Sulfocarbamoylgonyautoxin-2 | 10-250 | 0.9924 | -5.36% | 10-250 | 0.9947 | 6.33% | 10-250 | 0.9964 | -8.59% | 10-250 | 0.9986 | 11.32% |
| 558 | N-Sulfocarbamoylgonyautoxin-3 | 10-250 | 0.9935 | -7.26% | 10-250 | 0.9963 | 8.14% | 10-250 | 0.9978 | -3.25% | 10-250 | 0.9932 | 6.32% |
| 559 | Decarbamovlgonyautoxin-2 | 10-250 | 0.9954 | -6.93% | 10-250 | 0.9957 | -9.15% | 10-250 | 0.9964 | -6.39% | 10-250 | 0.9954 | -7.74% |
| 560 | Decarbamovlgonyautoxin-3 | 10-250 | 0.9973 | -4.63% | 10-250 | 0.9902 | -8.19% | 10-250 | 0.9957 | 2.15% | 10-250 | 0.9966 | -14.36% |
| 561 | Decarbamoylneosaxitoxin dihydrochloride | 10-250 | 0.9914 | -8.74% | 10-250 | 0.9947 | -6.28% | 10-250 | 0.9967 | 6.39% | 10-250 | 0.9988 | 9.69% |
| 562 | Decarbamoylsaxitoxin | 10-250 | 0.9921 | -6.34% | 10-250 | 0.9913 | -2.31% | 10-250 | 0.9948 | -5.15% | 10-250 | 0.9945 | 7.48% |
| 563 | Gonyautoxin-1 | 10-250 | 0.9963 | -8.74% | 10-250 | 0.9901 | -5.18% | 10-250 | 0.9947 | 8.62% | 10-250 | 0.9917 | -9.63% |
| 564 | Gonyautoxin-4 | 10-250 | 0.9975 | 6.31% | 10-250 | 0.9946 | -7.49% | 10-250 | 0.9964 | 8.30% | 10-250 | 0.9923 | -12.54% |
| 565 | Gonyautoxin-2 | 10-250 | 0.9943 | 9.74% | 10-250 | 0.9946 | -6.39% | 10-250 | 0.9953 | -7.59% | 10-250 | 0.9948 | 8.86% |
| 566 | Gonyautoxin-3 | 10-250 | 0.9928 | 8.62% | 10-250 | 0.9947 | -8.44% | 10-250 | 0.9961 | -6.11% | 10-250 | 0.9969 | 10.69% |
| 567 | Gonyautoxin-6 | 10-250 | 0.9993 | 7.49% | 10-250 | 0.9915 | -5.76% | 10-250 | 0.9973 | -3.96% | 10-250 | 0.9987 | -4.12% |
| 568 | Neosaxitoxin | 10-250 | 0.9962 | -8.47% | 10-250 | 0.9935 | -3.26% | 10-250 | 0.9934 | -8.57% | 10-250 | 0.9954 | -7.69% |
| 569 | Saxitoxin dihydrochloride | 10-250 | 0.9974 | -11.88% | 10-250 | 0.9946 | 8.87% | 10-250 | 0.9985 | 9.67% | 10-250 | 0.9938 | 8.62% |
| 570 | Tetrodotoxin | 10-250 | 0.9935 | 10.17% | 10-250 | 0.9949 | -7.14% | 10-250 | 0.9985 | 8.85% | 10-250 | 0.9984 | 18.71% |
| 571 | Microcystin RR | 10-250 | 0.9939 | -6.40% | 10-250 | 0.9937 | 10.78% | 10-250 | 0.9928 | -8.38% | 10-250 | 0.9988 | -4.19% |
| 572 | Microcystin LR | 10-250 | 0.9987 | -14.75% | 10-250 | 0.9970 | 5.42% | 10-250 | 0.9993 | -1.81% | 10-250 | 0.9995 | 3.02% |
| 573 | Okadaic Acid | 10-250 | 0.9975 | 6.64% | 10-250 | 0.9967 | -10.98% | 20-250 | 0.9995 | 7.28% | 20-250 | 0.9979 | 6.54% |
| 574 | Nodularin | 10-250 | 0.9970 | -27.56% | 10-250 | 0.9971 | -6.89% | 10-250 | 0.998 | -5.53% | 10-250 | 0.9982 | -0.08% |
| 575 | Sulfabenzamide | 10-250 | 0.9928 | -8.90% | 10-250 | 0.9987 | -12.13% | 10-250 | 0.9948 | -3.64% | 10-250 | 0.9921 | -10.82% |
| 576 | Sulfadiazine | 10-250 | 0.9977 | 1.21% | 10-250 | 0.9999 | -5.84% | 10-250 | 0.9956 | -4.91% | 10-250 | 0.9932 | -8.93% |
| 577 | Sulfadimethoxine | 10-250 | 0.9993 | 14.83% | 10-250 | 0.9947 | 2.23% | 10-250 | 0.9948 | 10.60% | 10-250 | 0.9966 | 21.93% |
| 578 | Sulfamethazine | 10-250 | 0.9980 | -7.42% | 10-250 | 0.9984 | -2.83% | 10-250 | 0.9946 | -3.59% | 10-250 | 0.9963 | -2.70% |
| 579 | Sulfadoxine | 10-250 | 0.9976 | 0.51% | 10-250 | 0.9961 | 4.63% | 10-250 | 0.9945 | -6.95% | 10-250 | 0.9962 | 0.65% |
| 580 | Sulfamerazine | 10-250 | 0.9992 | -9.84% | 10-250 | 0.9977 | -6.67% | 10-250 | 0.9950 | -27.31% | 10-250 | 0.9936 | -21.48% |
| 581 | Sulfamethizole | 10-250 | 0.9966 | -10.20% | 10-250 | 0.9997 | -12.37% | 10-250 | 0.9949 | -7.78% | 10-250 | 0.9966 | -5.43% |
| 582 | Sulfamethoxypyridazine | 10-250 | 0.9988 | 7.43% | 10-250 | 0.9984 | 0.34% | 10-250 | 0.9944 | -1.05% | 10-250 | 0.9929 | -3.14% |
| 583 | Sulfaphenazole | 10-250 | 0.9967 | 6.33% | 10-250 | 0.9957 | 5.85% | 10-250 | 0.9943 | -8.77% | 10-250 | 0.9972 | -6.43% |
| 584 | Sulfapyrazole | 10-250 | 0.9957 | 15.81% | 10-250 | 0.9985 | -3.46% | 10-250 | 0.9927 | 24.74% | 10-250 | 0.9971 | -11.56% |
| 585 | Sulfapyridine | 10-250 | 0.9986 | 7.57% | 10-250 | 0.9991 | -4.49% | 10-250 | 0.9959 | -2.07% | 10-250 | 0.9966 | -6.86% |
| 586 | Sulfaquinoxaline | 10-250 | 0.9960 | -7.68% | 10-250 | 0.9980 | -4.88% | 10-250 | 0.9940 | -14.88% | 10-250 | 0.9969 | -0.07% |
| 587 | Sulfathiazole | 10-250 | 0.9985 | 3.23% | 10-250 | 0.9972 | 1.87% | 10-250 | 0.9942 | -7.29% | 10-250 | 0.9971 | -2.89% |
| 588 | Sulfisomidine | 10-250 | 0.9985 | 6.09% | 10-250 | 0.9975 | 6.48% | 10-250 | 0.9985 | -1.73% | 10-250 | 0.9988 | -5.56% |
| 589 | Trimethoprim | 10-250 | 0.9935 | 3.33% | 10-250 | 0.9995 | -3.67% | 10-250 | 0.9990 | -8.43% | 10-250 | 0.9940 | -3.25% |
| 590 | Cinoxacin | 10-250 | 0.9985 | -21.63% | 10-250 | 0.9988 | -6.70% | 10-250 | 0.9959 | -12.39% | 10-250 | 0.9985 | -12.64% |
| 591 | Danofloxacin | 10-250 | 0.9937 | 28.55% | 10-250 | 0.9995 | -3.31% | 10-250 | 0.9930 | -3.87% | 10-250 | 0.9932 | -10.89% |
| 592 | Difloxacin | 10-250 | 0.9977 | 3.01% | 10-250 | 0.9998 | 1.15% | 10-250 | 0.9985 | -1.78% | 10-250 | 0.9947 | -2.22% |
| 593 | Enrofloxacin | 10-250 | 0.9953 | -4.07% | 10-250 | 0.9958 | 2.64% | 10-250 | 0.9932 | -4.21% | 10-250 | 0.9922 | -6.43% |
| 594 | Flumequine | 10-250 | 0.9981 | 3.43% | 10-250 | 0.9993 | -0.55% | 10-250 | 0.9954 | 1.97% | 10-250 | 0.9971 | -1.73% |
| 595 | Gatifloxacin | 10-250 | 0.9966 | -5.78% | 10-250 | 0.9994 | -2.55% | 10-250 | 0.9932 | -8.00% | 10-250 | 0.9949 | -3.55% |
| 596 | Lomefloxacin | 10-250 | 0.9999 | -5.18% | 10-250 | 0.9982 | 10.98% | 10-250 | 0.9938 | -1.89% | 10-250 | 0.9956 | -3.91% |
| 597 | Marbofloxacin | 10-250 | 0.9970 | 9.97% | 10-250 | 0.9999 | -5.13% | 10-250 | 0.9935 | -7.96% | 10-250 | 0.9950 | -0.68% |
| 598 | Moxifloxacin | 10-250 | 0.9966 | -5.23% | 10-250 | 0.9994 | -1.21% | 10-250 | 0.9973 | -2.41% | 10-250 | 0.9982 | -1.22% |
| 599 | Nalidixic acid | 10-250 | 0.9974 | -5.78% | 10-250 | 0.9986 | -4.95% | 10-250 | 0.9957 | -1.02% | 10-250 | 0.9980 | 1.86% |
| 600 | Ofloxacin | 10-250 | 0.9977 | 2.92% | 10-250 | 0.9990 | -6.74% | 10-250 | 0.9932 | -1.26% | 10-250 | 0.9937 | -3.88% |
| 601 | Orbifloxacin | 10-250 | 0.9968 | -0.35% | 10-250 | 0.9995 | -13.31% | 10-250 | 0.9972 | 0.94% | 10-250 | 0.9979 | -2.45% |
| 602 | Oxolinic acid | 10-250 | 0.9984 | 4.79% | 10-250 | 0.9997 | 0.21% | 10-250 | 0.9961 | -7.72% | 10-250 | 0.9976 | -8.84% |
| 603 | Sarafloxacin | 10-250 | 0.9973 | -7.05% | 10-250 | 0.9996 | -5.30% | 10-250 | 0.9985 | -3.48% | 10-250 | 0.9981 | -3.02% |
| 604 | Sparfloxacin | 10-250 | 0.9977 | -15.57% | 20-250 | 0.9996 | -22.33% | 10-250 | 0.9977 | -7.82% | 20-250 | 0.9989 | -13.75% |
| 605 | Tosufloxacin | 10-250 | 0.9948 | 7.50% | 10-250 | 0.9992 | 13.10% | 10-250 | 0.9924 | -1.54% | 10-250 | 0.9992 | -4.25% |
| 606 | Albendazole | 10-250 | 0.9974 | -3.89% | 10-250 | 0.9969 | -11.71% | 10-250 | 0.9935 | -1.49% | 10-250 | 0.9977 | -12.39% |
| 607 | Albendazole-2-aminosulfone | 10-250 | 0.9932 | -6.35% | 10-250 | 0.9967 | 1.17% | 10-250 | 0.9946 | 1.00% | 10-250 | 0.9949 | -11.39% |
| 608 | Albendazole sulfoxide | 10-250 | 0.9972 | 26.41% | 10-250 | 0.9979 | -15.61% | 10-250 | 0.9939 | -2.86% | 10-250 | 0.9961 | -4.67% |
| 609 | Mebendazole-amine | 10-250 | 0.9983 | 4.25% | 10-250 | 0.9991 | -11.79% | 10-250 | 0.9977 | -8.39% | 10-250 | 0.9927 | -20.38% |
| 610 | Thiabendazole | 10-250 | 0.9952 | 9.99% | 10-250 | 0.9914 | -11.20% | 10-250 | 0.9954 | -11.89% | 10-250 | 0.9962 | -12.35% |
| 611 | Dimetridazole | 10-250 | 0.9994 | 2.88% | 10-250 | 0.9953 | 4.60% | 10-250 | 0.9959 | -11.06% | 10-250 | 0.9962 | -15.86% |
| 612 | Fenbendazole | 10-250 | 0.9941 | -4.85% | 10-250 | 0.9974 | -12.84% | 10-250 | 0.9926 | -8.28% | 10-250 | 0.9970 | -13.55% |
| 613 | Flubendazole | 10-250 | 0.9942 | -16.80% | 10-250 | 0.9978 | -15.25% | 10-250 | 0.9988 | 1.87% | 10-250 | 0.9941 | -9.15% |
| 614 | Hydroxy ipronidazole | 10-250 | 0.9976 | 21.14% | 10-250 | 0.9974 | -11.17% | 10-250 | 0.9969 | -10.49% | 10-250 | 0.9957 | -13.78% |
| 615 | Ipronidazole | 10-250 | 0.9987 | 1.17% | 10-250 | 0.9976 | -15.35% | 10-250 | 0.9965 | -4.24% | 10-250 | 0.9945 | -34.04% |
| 616 | Mebendazole | 10-250 | 0.9961 | -21.97% | 10-250 | 0.9967 | -14.14% | 10-250 | 0.9983 | -9.81% | 10-250 | 0.9990 | -19.78% |
| 617 | Cambendazole | 10-250 | 0.9972 | 10.88% | 10-250 | 0.9971 | -10.25% | 10-250 | 0.9933 | -9.19% | 10-250 | 0.9978 | -20.22% |
| 618 | 5-Hydroxythiabendazole | 10-250 | 0.9979 | 13.12% | 10-250 | 0.9968 | 5.60% | 10-250 | 0.9959 | 7.33% | 10-250 | 0.9966 | -0.71% |
| 619 | Levamisole | 10-250 | 0.9976 | 5.28% | 10-250 | 0.9973 | 15.48% | 10-250 | 0.9965 | 8.66% | 10-250 | 0.9978 | 10.41% |
| 620 | Metronidazole | 10-250 | 0.9957 | 10.41% | 10-250 | 0.9970 | 0.03% | 10-250 | 0.9981 | -8.10% | 10-250 | 0.9984 | -13.55% |
| 621 | Oxfendazole | 10-250 | 0.9974 | 30.10% | 10-250 | 0.9954 | 2.75% | 10-250 | 0.9923 | -4.69% | 10-250 | 0.9941 | -2.99% |
| 622 | Oxibendazole | 10-250 | 0.9968 | -16.29% | 10-250 | 0.9993 | 1.61% | 10-250 | 0.9942 | -4.88% | 10-250 | 0.9984 | -8.47% |
| 623 | Ronidazole | 10-250 | 0.9979 | -12.77% | 10-250 | 0.9935 | -1.05% | 10-250 | 0.9993 | -1.51% | 10-250 | 0.9922 | -2.19% |
| 624 | Secnidazole | 10-250 | 0.9973 | 0.06% | 10-250 | 0.9976 | 3.27% | 10-250 | 0.9958 | -7.60% | 10-250 | 0.9957 | -1.22% |
| 625 | Tinidazole | 10-250 | 0.9952 | -5.79% | 10-250 | 0.9981 | -6.58% | 10-250 | 0.9963 | 1.18% | 10-250 | 0.9940 | -3.83% |
| 626 | Triclabendazole | 10-250 | 0.9947 | -10.83% | 10-250 | 0.9996 | -13.08% | 10-250 | 0.9984 | 0.54% | 10-250 | 0.9989 | -5.34% |
| 627 | Clindamycin | 10-250 | 0.9975 | -5.23% | 10-250 | 0.9976 | 1.17% | 10-250 | 0.9982 | -1.60% | 10-250 | 0.9942 | -6.19% |
| 628 | Doramectin | 10-250 | 0.9957 | -7.55% | 10-250 | 0.9984 | -14.06% | 10-250 | 0.9973 | -3.70% | 10-250 | 0.9944 | -6.33% |
| 629 | Eprinomectin | 10-250 | 0.9961 | 14.73% | 10-250 | 0.9953 | 21.86% | 20-250 | 0.9986 | 4.34% | 20-250 | 0.9925 | -24.54% |
| 630 | Ivermectin | 10-250 | 0.9950 | -2.23% | 10-250 | 0.9997 | 14.80% | 10-250 | 0.9988 | -5.84% | 20-250 | 0.9995 | 2.87% |
| 631 | Leucomycin A1 | 10-250 | 0.9966 | -11.67% | 10-250 | 0.9932 | 2.75% | 10-250 | 0.9988 | -7.39% | 10-250 | 0.9926 | -6.49% |
| 632 | Spiramycin | 10-250 | 0.9956 | -4.31% | 10-250 | 0.9943 | -1.63% | 10-250 | 0.9984 | 0.29% | 10-250 | 0.9989 | -3.88% |
| 633 | Tilmicosin | 10-250 | 0.9975 | 4.74% | 10-250 | 0.9973 | 1.98% | 10-250 | 0.9985 | -17.82% | 10-250 | 0.9978 | 16.91% |
| 634 | Tylosin | 10-250 | 0.9995 | 0.24% | 10-250 | 0.9975 | -2.90% | 10-250 | 0.9975 | 6.23% | 10-250 | 0.9930 | -10.32% |
| 635 | Virginiamycin M1 | 10-250 | 0.9936 | 4.81% | 10-250 | 0.9983 | 14.95% | 10-250 | 0.9985 | 5.81% | 10-250 | 0.9966 | -17.98% |
| 636 | Beclomethasone | 10-250 | 0.9973 | -3.64% | 10-250 | 0.9969 | -1.17% | 10-250 | 0.9987 | -2.52% | 10-250 | 0.9957 | -5.96% |
| 637 | Beclomethasone dipropionate | 10-250 | 0.9988 | 1.25% | 10-250 | 1.0000 | -4.98% | 10-250 | 0.9927 | -9.73% | 10-250 | 0.9994 | -11.28% |
| 638 | Betamethasone dipropionate | 10-250 | 0.9994 | 5.91% | 10-250 | 0.9998 | 5.79% | 10-250 | 0.9991 | -0.76% | 10-250 | 0.9991 | -2.88% |
| 639 | Betamethasone valerate | 10-250 | 0.9950 | -7.39% | 10-250 | 0.9929 | 5.26% | 10-250 | 0.9993 | -2.83% | 10-250 | 0.9935 | -6.61% |
| 640 | Chlormadinone acetate | 10-250 | 0.9990 | -8.42% | 10-250 | 0.9994 | 2.37% | 10-250 | 0.9986 | -3.03% | 10-250 | 0.9936 | 0.46% |
| 641 | Clobetasol 17- propionate | 10-250 | 0.9998 | -9.02% | 10-250 | 0.9968 | 11.59% | 10-250 | 0.9987 | 0.99% | 10-250 | 0.9933 | -8.19% |
| 642 | Clobetasone butyrate | 10-250 | 0.9990 | 3.06% | 10-250 | 0.9983 | -0.93% | 10-250 | 0.9985 | -6.08% | 10-250 | 0.9989 | 2.01% |
| 643 | Cortisone | 10-250 | 0.9946 | 4.45% | 10-250 | 0.9960 | 1.83% | 10-250 | 0.9928 | -13.06% | 10-250 | 0.9923 | -18.54% |
| 644 | Deflazacort | 10-250 | 0.9980 | 9.19% | 10-250 | 0.9942 | -3.75% | 10-250 | 0.9987 | -3.75% | 10-250 | 0.9932 | -2.63% |
| 645 | Dexamethasone | 10-250 | 0.9953 | 12.65% | 10-250 | 0.9937 | -6.30% | 10-250 | 0.9986 | -8.72% | 10-250 | 0.9966 | -3.16% |
| 646 | Diflorasone Diacetate | 10-250 | 0.9998 | 2.24% | 10-250 | 0.9968 | 6.21% | 10-250 | 0.9923 | -0.11% | 10-250 | 0.9946 | 0.39% |
| 647 | Epitestosterone | 10-250 | 0.9976 | 0.06% | 10-250 | 0.9968 | 9.13% | 10-250 | 0.9999 | 3.79% | 10-250 | 0.9946 | 4.78% |
| 648 | Fludrocortisone | 10-250 | 0.9977 | -11.63% | 10-250 | 0.9968 | -11.23% | 10-250 | 0.9925 | -8.02% | 10-250 | 0.9912 | -2.35% |
| 649 | Flumethasone | 10-250 | 0.9959 | 9.65% | 10-250 | 0.9948 | 13.55% | 10-250 | 0.9938 | -4.75% | 10-250 | 0.9924 | -18.23% |
| 650 | Flumethasone pivalate | 10-250 | 0.9993 | -13.17% | 10-250 | 0.9965 | -16.43% | 10-250 | 0.9933 | 7.98% | 10-250 | 0.9959 | -7.39% |
| 651 | Fluocinolone acetonide | 10-250 | 0.9999 | 7.43% | 10-250 | 0.9966 | -8.56% | 10-250 | 0.9933 | -6.18% | 10-250 | 0.9962 | -15.53% |
| 652 | Flurandrenolide | 10-250 | 0.9975 | 2.18% | 10-250 | 0.9994 | -0.56% | 10-250 | 0.9917 | -10.18% | 10-250 | 0.9967 | -12.81% |
| 653 | Fluoromethalone | 10-250 | 0.9941 | -2.22% | 10-250 | 0.9952 | -11.25% | 10-250 | 0.9984 | -3.91% | 10-250 | 0.9996 | -4.32% |
| 654 | Fluticasone propionate | 10-250 | 0.9995 | 8.11% | 10-250 | 0.9953 | 5.95% | 10-250 | 0.9987 | -0.39% | 10-250 | 0.9964 | -5.85% |
| 655 | Halcinonide | 10-250 | 0.9982 | 12.41% | 10-250 | 0.9967 | 2.48% | 10-250 | 0.9989 | -7.64% | 10-250 | 0.9930 | -5.55% |
| 656 | Hydrocortisone | 10-250 | 0.9952 | 10.52% | 10-250 | 0.9960 | 1.90% | 10-250 | 0.9961 | -6.04% | 10-250 | 0.9974 | -20.71% |
| 657 | Megestrol | 10-250 | 0.9996 | 1.31% | 10-250 | 0.9994 | 6.43% | 10-250 | 0.9930 | -6.58% | 10-250 | 0.9923 | -11.05% |
| 658 | Melengestrol acetate | 10-250 | 0.9972 | 9.79% | 10-250 | 0.9977 | 10.23% | 10-250 | 0.9929 | -9.19% | 10-250 | 0.9949 | -3.79% |
| 659 | Methylprednisolone | 10-250 | 0.9975 | 3.23% | 10-250 | 0.9970 | -8.43% | 10-250 | 0.9936 | -4.26% | 10-250 | 0.9913 | -6.29% |
| 660 | Mometasone Furoate | 10-250 | 0.9993 | 0.95% | 10-250 | 0.9938 | 16.48% | 10-250 | 0.9986 | -14.09% | 10-250 | 0.9960 | -27.04% |
| 661 | Prednicarbate | 10-250 | 0.9988 | 1.18% | 10-250 | 0.9923 | 6.16% | 10-250 | 0.9988 | -16.12% | 10-250 | 0.9951 | -10.10% |
| 662 | Testosterone | 10-250 | 0.9973 | -13.12% | 10-250 | 0.9943 | 1.91% | 10-250 | 0.9941 | -5.96% | 10-250 | 0.9931 | -8.28% |
| 663 | Triamcinolone acetonide | 10-250 | 0.9956 | 4.67% | 10-250 | 0.9971 | 9.86% | 10-250 | 0.9936 | 5.77% | 10-250 | 0.9952 | 5.51% |
| 664 | Bambuterol | 10-250 | 0.9971 | 12.79% | 10-250 | 0.9993 | -4.80% | 10-250 | 0.9917 | -4.08% | 10-250 | 0.9966 | -6.73% |
| 665 | Hydroxymethyl clenbuterol | 10-250 | 0.9996 | 4.88% | 10-250 | 0.9985 | -2.24% | 10-250 | 0.9939 | 1.42% | 10-250 | 0.9926 | 3.24% |
| 666 | Clencyclohexerol | 10-250 | 0.9981 | 7.55% | 10-250 | 0.9989 | -0.62% | 10-250 | 0.9931 | -3.13% | 10-250 | 0.9955 | -1.96% |
| 667 | Clenhexerol | 10-250 | 0.9976 | -21.64% | 10-250 | 0.9962 | -28.09% | 10-250 | 0.9936 | -3.41% | 10-250 | 0.9937 | -19.87% |
| 668 | Clenisopenterol | 10-250 | 0.9938 | -8.83% | 10-250 | 0.9981 | -9.90% | 10-250 | 0.9940 | -11.89% | 10-250 | 0.9928 | -12.73% |
| 669 | Clenproperol | 10-250 | 0.9970 | -6.06% | 10-250 | 0.9960 | -14.17% | 10-250 | 0.9953 | -1.59% | 10-250 | 0.9938 | -6.47% |
| 670 | Fenoterol | 10-250 | 0.9933 | -4.46% | 10-250 | 0.9922 | -8.38% | 10-250 | 0.9927 | -13.18% | 10-250 | 0.9956 | 10.53% |
| 671 | Formoterol | 10-250 | 1.0000 | -8.09% | 10-250 | 0.9989 | -3.50% | 10-250 | 0.9985 | -2.33% | 10-250 | 0.9922 | -4.91% |
| 672 | Pirbuterol | 10-250 | 0.9977 | -5.93% | 10-250 | 0.9992 | -24.22% | 10-250 | 0.9933 | 3.08% | 10-250 | 0.9965 | -9.66% |
| 673 | Ractopamine | 10-250 | 0.9958 | -5.05% | 10-250 | 0.9987 | -5.19% | 10-250 | 0.9930 | -3.53% | 10-250 | 0.9948 | -5.45% |
| 674 | Ritodrine | 10-250 | 0.9988 | -2.90% | 10-250 | 0.9981 | -6.90% | 10-250 | 0.9928 | 10.28% | 10-250 | 0.9952 | -2.92% |
| 675 | Salmeterol | 10-250 | 0.9942 | 20.15% | 10-250 | 0.9959 | 8.65% | 10-250 | 0.9984 | -18.68% | 10-250 | 0.9933 | -18.71% |
| 676 | Sotalol | 10-250 | 0.9981 | -13.72% | 10-250 | 0.9975 | -14.87% | 10-250 | 0.9930 | -6.25% | 10-250 | 0.9927 | -9.99% |
| 677 | Terbutaline | 10-250 | 0.9958 | -10.01% | 10-250 | 0.9967 | -13.30% | 10-250 | 0.9949 | 3.37% | 10-250 | 0.9976 | -3.17% |
| 678 | Tulobuterol | 10-250 | 1.0000 | 8.05% | 10-250 | 0.9968 | -12.63% | 10-250 | 0.9950 | -8.74% | 10-250 | 0.9958 | -10.35% |
| 679 | Clenpenterol | 10-100 | 1.0000 | 34.20% | 10-250 | 0.9979 | -14.13% | 10-100 | 0.9943 | -7.29% | 10-250 | 0.9943 | -10.96% |
| 680 | 4-Acetamidophenol | 10-250 | 0.9931 | -16.68% | 10-250 | 0.9996 | -1.79% | 10-250 | 0.9997 | -10.96% | 10-250 | 0.9993 | -24.36% |
| 681 | Chlorpromazine | 10-250 | 0.9979 | -1.05% | 10-250 | 0.9982 | -10.36% | 10-250 | 0.9939 | -6.43% | 10-250 | 0.9950 | -14.79% |
| 682 | Clopidol | 10-250 | 0.9946 | -5.69% | 10-250 | 0.9996 | 0.29% | 10-250 | 0.9948 | -1.64% | 10-250 | 0.9957 | -5.17% |
| 683 | Dapsone | 10-250 | 0.9994 | -15.08% | 10-250 | 0.9981 | -16.55% | 10-250 | 0.9954 | 6.38% | 10-250 | 0.9938 | -9.38% |
| 684 | Carbadox | 10-250 | 0.9997 | 4.97% | 10-250 | 0.9993 | -1.11% | 10-250 | 0.9972 | -13.77% | 10-250 | 0.9987 | -10.69% |
| 685 | Haloperidol | 10-250 | 0.9960 | -9.94% | 10-250 | 0.9971 | -21.10% | 10-250 | 0.9931 | -8.71% | 10-250 | 0.9946 | 2.08% |
| 686 | Azaperol | 10-250 | 0.9954 | -3.54% | 10-250 | 0.9987 | -4.64% | 10-250 | 0.9911 | -5.55% | 10-250 | 0.9952 | -5.22% |
| 687 | Azaperone | 10-250 | 0.9949 | -4.12% | 10-250 | 0.9983 | -3.60% | 10-250 | 0.9954 | -14.05% | 10-250 | 0.9961 | -9.02% |
| 688 | Propionylpromazine | 10-250 | 0.9935 | 3.65% | 10-250 | 0.9979 | -8.02% | 10-250 | 0.9921 | -3.92% | 10-250 | 0.9941 | -11.63% |
| 689 | Xylazine | 10-250 | 0.9996 | -14.07% | 10-250 | 0.9982 | 0.08% | 10-250 | 0.9955 | -7.09% | 10-250 | 0.9973 | -11.91% |
| 690 | Carbamazepine | 10-250 | 0.9990 | 5.96% | 10-250 | 0.9957 | 7.74% | 10-250 | 0.9950 | -15.53% | 10-250 | 0.9965 | -18.66% |
| 691 | Diphenhydramine | 10-250 | 0.9991 | -1.78% | 10-250 | 0.9993 | -11.03% | 10-250 | 0.9952 | -6.59% | 10-250 | 0.9959 | -21.78% |
| 692 | Imipramine | 10-250 | 0.9978 | 7.89% | 10-250 | 0.9993 | -10.71% | 10-250 | 0.9923 | -7.88% | 10-250 | 0.9957 | -12.63% |
| 693 | Sulpiride | 10-250 | 0.9982 | 5.48% | 10-250 | 0.9961 | -2.47% | 10-250 | 0.9986 | -5.99% | 10-250 | 0.9934 | -2.24% |
| 694 | Zolpidem | 10-250 | 0.9979 | 2.29% | 10-250 | 0.9988 | 3.33% | 10-250 | 0.9946 | -5.57% | 10-250 | 0.9944 | -8.32% |
| 695 | Fluoxetine | 10-250 | 0.9991 | -18.91% | 10-250 | 0.9985 | -10.88% | 10-250 | 0.9986 | -4.83% | 10-250 | 0.9922 | -9.36% |
| 696 | Coffeine | 10-250 | 0.9976 | 7.25% | 10-250 | 0.9940 | 4.65% | 10-250 | 0.9923 | 21.41% | 10-250 | 0.9990 | -7.89% |
| 697 | Codeine | 10-250 | 0.9935 | 9.19% | 10-250 | 0.9969 | -2.46% | 10-250 | 0.9912 | 3.71% | 10-250 | 0.9958 | -9.91% |
| 698 | 1,7-Dimethylxanthine | 10-250 | 0.9993 | 3.48% | 10-250 | 0.9993 | -17.56% | 10-250 | 0.9919 | -3.85% | 10-250 | 0.9972 | 6.15% |
| 699 | Chloroprocaine | 10-250 | 0.9994 | 42.74% | 10-250 | 0.9982 | -2.24% | 10-250 | 0.9972 | 4.65% | 10-250 | 0.9983 | -2.05% |
| 700 | Cinchocaine | 10-250 | 0.9992 | -2.60% | 10-250 | 0.9990 | -15.47% | 10-250 | 0.9974 | 2.58% | 10-250 | 0.9952 | -7.15% |
| 701 | Lidocaine | 10-250 | 0.9993 | 42.90% | 10-250 | 0.9974 | 13.64% | 10-250 | 0.9945 | 18.81% | 10-250 | 0.9985 | 30.95% |
| 702 | Procainamide | 10-250 | 0.9913 | -22.27% | 10-250 | 0.9983 | -1.39% | 10-250 | 0.9939 | -2.32% | 10-250 | 0.9989 | -2.99% |
| 703 | Procaine | 10-250 | 0.9971 | 9.66% | 10-250 | 0.9954 | 23.41% | 10-250 | 0.9986 | 0.39% | 10-250 | 0.9988 | -4.17% |
| 704 | Tetracaine | 10-250 | 0.9997 | -13.45% | 10-250 | 0.9973 | -22.24% | 10-250 | 0.9980 | -1.87% | 10-250 | 0.9973 | -12.73% |
| 705 | Brompheniramine | 10-250 | 0.9955 | -5.55% | 10-250 | 0.9987 | -14.21% | 10-250 | 0.9945 | 3.71% | 10-250 | 0.9938 | -3.57% |
| 706 | Cetirizine | 10-250 | 0.9976 | 0.66% | 10-250 | 0.9994 | -9.07% | 10-250 | 0.9938 | -1.02% | 10-250 | 0.9946 | -6.52% |
| 707 | Chlorpheniramine | 10-250 | 0.9964 | -7.72% | 10-250 | 0.9978 | -20.24% | 10-250 | 0.9991 | -4.58% | 10-250 | 0.9973 | 3.72% |
| 708 | Fluphenazine | 10-250 | 0.9976 | -9.37% | 10-250 | 0.9995 | -21.55% | 10-250 | 0.9973 | -1.66% | 10-250 | 0.9961 | 0.61% |
| 709 | Hydroxyzine | 10-250 | 0.9994 | -14.40% | 10-250 | 0.9998 | -28.36% | 10-250 | 0.9950 | -11.99% | 10-250 | 0.9963 | -14.21% |
| 710 | Promethazine | 10-250 | 0.9995 | -17.06% | 10-250 | 0.9984 | -21.24% | 10-250 | 0.9979 | -10.09% | 10-250 | 0.9968 | -6.86% |
| 711 | Terfenadine | 10-250 | 0.9976 | -0.48% | 10-250 | 0.9999 | -12.78% | 10-250 | 0.9967 | -1.94% | 10-250 | 0.9938 | -7.70% |
| 712 | Bifonazole | 10-250 | 0.9996 | -24.69% | 10-250 | 0.9970 | -29.73% | 10-250 | 0.9923 | -0.42% | 10-250 | 0.9951 | -5.61% |
| 713 | Econazole | 10-250 | 0.9977 | -22.58% | 10-250 | 0.9967 | -30.03% | 10-250 | 0.9937 | -2.63% | 10-250 | 0.9951 | -7.17% |
| 714 | Griseofulvin | 10-250 | 0.9988 | 36.26% | 10-250 | 0.9987 | 5.50% | 10-250 | 0.9981 | -4.57% | 10-250 | 0.9956 | 15.65% |
| 715 | Ketoconazole | 10-250 | 0.9995 | 6.85% | 10-250 | 0.9993 | -9.05% | 10-250 | 0.9964 | 13.88% | 10-250 | 0.9921 | 1.94% |
| 716 | Naftifine | 10-250 | 0.9989 | -11.58% | 10-250 | 0.9995 | -18.82% | 10-250 | 0.9957 | -7.94% | 10-250 | 0.9957 | -11.53% |
| 717 | Flunixin | 10-250 | 0.9974 | -22.95% | 10-250 | 0.9925 | -25.19% | 10-250 | 0.9978 | -4.34% | 10-250 | 0.9948 | -11.36% |
| 718 | Ketotifen | 10-250 | 0.9977 | -34.54% | 20-250 | 0.9921 | -44.24% | 10-250 | 0.9936 | -7.16% | 10-250 | 0.9945 | -14.22% |
| 719 | Lornoxicam | 10-250 | 0.9994 | -23.58% | 10-250 | 1.0000 | -28.54% | 10-250 | 0.9977 | -2.84% | 10-250 | 0.9958 | 2.15% |
| 720 | Melitracen | 10-250 | 0.9954 | 27.71% | 10-250 | 0.9938 | 4.08% | 10-250 | 0.9940 | -3.95% | 10-250 | 0.9954 | -14.24% |
| 721 | Oxaprozin | 10-250 | 0.9978 | -3.26% | 10-250 | 0.9993 | -17.60% | 10-250 | 0.9976 | -1.15% | 10-250 | 0.9942 | -2.08% |
| 722 | Antipyrine | 10-250 | 0.9994 | -5.22% | 10-250 | 0.9967 | -25.28% | 10-250 | 0.9967 | -1.75% | 10-250 | 0.9986 | 8.23% |
| 723 | Sulfaguanidine | 10-250 | 0.9941 | -7.50% | 10-250 | 0.9939 | 4.67% | 10-250 | 0.9987 | -4.49% | 10-250 | 0.9985 | 0.12% |
| 724 | Difloxacin hydrochloride | 10-250 | 0.9953 | 0.99% | 10-250 | 0.9967 | 5.16% | 10-250 | 0.9986 | -4.08% | 10-250 | 0.9948 | -2.80% |
| 725 | Fleroxacin | 10-250 | 0.9976 | 13.58% | 10-250 | 0.9986 | 1.14% | 10-250 | 0.9973 | 3.39% | 10-250 | 0.9963 | -0.93% |
| 726 | Enoxacin | 10-250 | 0.9989 | -4.50% | 10-250 | 0.9976 | -6.03% | 10-250 | 0.9980 | 15.10% | 10-250 | 0.9980 | -5.43% |
| 727 | Sarafloxacin hydrochloride | 10-250 | 0.9956 | 7.86% | 10-250 | 0.9983 | -6.88% | 10-250 | 0.9983 | 7.62% | 10-250 | 0.9950 | 6.95% |
| 728 | Nadifloxacin | 10-250 | 0.9990 | 7.30% | 20-250 | 0.9999 | -20.62% | 10-250 | 0.9950 | -7.55% | 10-250 | 0.9957 | -0.52% |
| 729 | Norfloxacin | 10-250 | 0.9985 | -11.08% | 10-250 | 0.9978 | 4.37% | 10-250 | 0.9969 | 6.17% | 10-250 | 0.9998 | -3.51% |
| 730 | Gemifloxacin mesylate | 10-250 | 0.9941 | 3.55% | 10-250 | 0.9988 | -8.50% | 10-250 | 0.9965 | 24.30% | 10-250 | 0.9960 | 9.97% |
| 731 | Glipizide | 10-250 | 0.9931 | 4.97% | 10-250 | 0.9991 | 10.10% | 10-250 | 0.9961 | -2.89% | 10-250 | 0.9986 | -14.29% |
| 732 | Repaglinide | 10-250 | 0.9979 | -12.62% | 10-250 | 0.9996 | -4.49% | 10-150 | 0.9986 | -3.16% | 10-150 | 0.9981 | -3.01% |
| 733 | Hygromycin B | 10-250 | 0.9956 | 11.26% | 20-250 | 0.9989 | 0.09% | 10-250 | 0.9983 | -1.35% | 10-250 | 0.9951 | -12.73% |
| 734 | Sulfisoxazole | 10-250 | 0.9989 | -10.27% | 10-250 | 0.9990 | -0.30% | 10-250 | 0.9972 | 2.27% | 10-250 | 0.9951 | -4.56% |
| 735 | Amikacin | 10-250 | 0.9983 | -2.12% | 10-250 | 0.9950 | 2.00% | 10-250 | 0.9984 | -8.42% | 10-250 | 0.9923 | -9.67% |
| 736 | Tolbutamide | 10-250 | 0.9964 | -4.66% | 10-250 | 0.9999 | 16.34% | 10-250 | 0.9983 | 3.93% | 10-250 | 0.9972 | -1.41% |
| 737 | Gliclazide | 10-250 | 0.9986 | -2.56% | 10-250 | 0.9961 | -1.12% | 10-250 | 0.9931 | 13.31% | 10-250 | 0.9988 | 7.49% |
| 738 | Gliquidone | 10-250 | 0.9997 | -9.65% | 10-250 | 0.9942 | -3.57% | 10-250 | 0.9920 | -7.93% | 10-150 | 0.9986 | -26.89% |
| 739 | Glyburide | 10-250 | 0.9967 | 3.11% | 10-250 | 0.9984 | -0.25% | 10-250 | 0.9960 | 9.38% | 10-250 | 0.9993 | 18.22% |
| 740 | 1-Aminohydantoin hydrochloride | 10-250 | 0.9968 | 10.35% | 10-250 | 0.9967 | 28.98% | 50-250 | 0.9976 | 37.04% | 20-250 | 0.9978 | -20.13% |
| 741 | Pioglitazone hydrochloride | 10-250 | 0.9984 | -5.55% | 10-250 | 0.9975 | -3.70% | 10-150 | 0.9986 | -9.40% | 10-150 | 0.9943 | 16.22% |
| 742 | Metformin hydrochloride | 10-250 | 0.9963 | 2.94% | 10-250 | 0.9903 | -8.74% | 10-250 | 0.9987 | 12.81% | 10-250 | 0.9986 | -18.56% |
| 743 | Glimepiride | 10-100 | 0.9943 | 31.19% | 10-250 | 0.9982 | -2.44% | 10-250 | 0.9986 | 10.44% | 10-250 | 0.9996 | 22.17% |
| 744 | Sulfamonomethoxine | 10-250 | 0.9940 | 3.08% | 10-250 | 0.9959 | -1.99% | 10-250 | 0.9983 | 3.33% | 10-250 | 0.9919 | 6.32% |
| 745 | Sulfacetamide | 10-250 | 0.9987 | -5.54% | N.D | N.D | N.D | 10-250 | 0.9922 | -3.89% | 10-250 | 0.9997 | 0.98% |
| 746 | Sulfachlorpyridazine | 10-250 | 0.9937 | -8.52% | 10-250 | 0.9981 | 10.27% | 10-250 | 0.9950 | 11.68% | 10-250 | 0.9945 | 1.79% |
| 747 | Sulfamoxole | 10-250 | 0.9985 | 5.57% | 10-250 | 0.9909 | 4.48% | 10-250 | 0.9995 | -0.10% | 10-250 | 1.0000 | 9.62% |
| 748 | Sulfanitran | 10-250 | 0.9958 | 11.71% | 10-250 | 0.9990 | -7.53% | 10-250 | 0.9963 | -4.04% | 10-250 | 0.9925 | 2.60% |
| 749 | Sulfameter | 10-250 | 0.9955 | -12.12% | 10-250 | 0.9967 | -6.35% | 10-250 | 0.9947 | 14.41% | 10-250 | 0.9969 | 4.50% |
| 750 | Sulfamethoxazole | 10-250 | 0.9966 | -4.16% | 10-250 | 0.9968 | 2.10% | 10-250 | 0.9948 | 4.68% | 10-250 | 0.9937 | -4.88% |
| 751 | Buformin hydrochloride | 10-250 | 0.9920 | -9.42% | 10-250 | 0.9964 | 2.51% | 10-250 | 0.9986 | 3.61% | 10-250 | 0.9986 | -14.99% |
| 752 | Phenformin hydrochloride | 10-250 | 0.9998 | -13.51% | 10-250 | 0.9989 | -11.97% | 10-250 | 0.9973 | 22.02% | 10-250 | 0.9947 | -9.13% |
| 753 | Glibornuride | 10-250 | 0.9974 | -13.71% | 10-250 | 0.9931 | 2.54% | 10-250 | 0.9987 | 17.47% | 10-250 | 0.9994 | 2.57% |
| 754 | Ciprofloxacin | 10-250 | 0.9989 | -3.76% | 10-250 | 0.9984 | -1.74% | 10-250 | 0.9969 | 5.85% | 10-250 | 0.9957 | -8.17% |
| 755 | Kanamycin sulfate | N.D | N.D | N.D | N.D | N.D | N.D | 50-250 | 0.9962 | 5.72% | 50-250 | 0.9984 | -3.11% |
| 756 | Tobramycin | N.D | N.D | N.D | N.D | N.D | N.D | 10-250 | 0.9925 | -7.40% | 10-250 | 0.9937 | -2.10% |

**Table S6.** Detailed information on the positive analytes in real-life samples.

| **Sample category** | **Number of samples** | **Sample source** | **Chemical group** | **Positive compound** | **Number of positive samples** | **Concentration** | **Maximum residue level** |
| --- | --- | --- | --- | --- | --- | --- | --- |
| **Tilapia** | 16 | Aquaculture farms, Guangdong Province, China | Pesticide | Fenpropidin | 2 | 0.03 mg/kg | 0.02 mg/kg in meats of swine, bovine, sheep, goat and poultry animals, European Union |
| **Grouper** | 16 | Retail markets, Shandong Province, China | Pesticide | Metazachlor | 1 | 0.04 mg/kg | 0.05 mg/kg in meats of swine, bovine, sheep, goat and poultry animals, European Union |
|  |  |  |  | Phorate sulfoxide | 1 | 0.005 mg/kg | 0.02 mg/kg in mammalian meats, China |
| **Oyster** | 16 | Retail markets, Shandong Province, China | Pesticide | Mefenpyr-diethyl | 1 | 0.005 mg/kg | 0.01 mg/kg in mammalian meats, Australia |
|  |  |  | Persistent Organic Pollutants | Dihexyl phthalate | 1 | 0.007 mg/kg | unavailable |
| **Scallop** | 16 | Retail markets, Shandong Province, China | Pesticide | Methoprene | 1 | 0.04 mg/kg | 0.10 mg/kg in meats of swine, bovine, sheep, and goat animals, United States |
